# Synergistic effects of LCN2 and TWEAK on the progression of psoriasis

**DOI:** 10.1038/s41423-025-01292-9

**Published:** 2025-05-15

**Authors:** Kaixuan Ren, Xueting Peng, Xudong Duan, Rongfang Feng, Christopher Cook, Mei Lu, Min Li, Hanjiang Gu, Xiaoyu Wang, Guorong Deng, Huiqun Ma, Yale Liu, Yumin Xia

**Affiliations:** 1 Department of Dermatology, The Second Affiliated Hospital of Xi’an Jiaotong University, Xi’an, China; 2 Department of Bone and Joint Surgery, The Second Affiliated Hospital of Xi’an Jiaotong University, Xi’an, China; 3 Department of Molecular and Cell Biology, University of California, Berkeley, Berkeley, CA USA; 4 Department of Dermatology, Jinling Hospital, Nanjing, China; 5 Department of Critical Care Medicine, The Second Affiliated Hospital of Xi’an Jiaotong University, Xi’an, China

**Keywords:** Psoriasis, Lipocalin 2, TWEAK, Fn14, synergistic, Psoriasis, Tumour-necrosis factors

## Abstract

Lipocalin 2 (LCN2) and the TWEAK/Fn14 signaling pathways are pivotal in psoriasis, influencing epidermal development, inflammatory cell chemotaxis, and inflammatory factor release. Despite their significant roles, the intricate relationship between LCN2 and TWEAK/Fn14 pathways remains unclear. Our study revealed the correlation between the expression of TWEAK, LCN2, and Fn14 in psoriatic lesions. We found that TWEAK is expressed by keratinocytes and macrophages, while LCN2 is expressed by keratinocytes and neutrophils. Surface plasmon resonance experiments demonstrated binding between LCN2 and Fn14, which was further validated by co-immunoprecipitation and cellular co-localization via immunofluorescence. In vitro, LCN2 promoted macrophage differentiation and TWEAK secretion, enhanced TWEAK and Fn14 expression in keratinocytes, and activated the MAPK signaling pathway. TWEAK upregulated LCN2 expression in neutrophils but not in keratinocytes. Bulk RNA-seq analysis revealed a synergistic effect of LCN2 and TWEAK in promoting inflammatory cytokine expression in keratinocytes, with enhanced MAPK pathway activation in the presence of M5 cytokines. Lcn2 knockout reduced Fn14 expression in skin lesions and serum TWEAK levels of imiquimod-induced murine psoriasis model, while Fn14 knockout attenuated the epidermal hyperplasia-promoting effects of TWEAK and LCN2. Overexpression of Fn14 in keratinocytes led to higher TWEAK expression upon LCN2 stimulation, suggesting a self-reinforcing loop among TWEAK, LCN2, and Fn14. We propose that LCN2 synergizes with TWEAK through Fn14 to drive psoriasis pathogenesis.

## One Sentence Summary

LCN2 and TWEAK synergistically interact with Fn14, leading to epidermal proliferation and inflammation in psoriasis through the MAPK signaling pathway; however, these effects can be reversed by LCN2 knockout.

## Introduction

Psoriasis is a chronic inflammatory skin disease affecting over 60 million individuals worldwide, including adults and children [1]. The primary pathogenic mechanisms underlying psoriasis include infection, trauma, aberrant keratinocyte proliferation, and activation of inflammatory cells [2]. These factors lead to the excessive proliferation and abnormal differentiation of keratinocytes and infiltration of various inflammatory cells. Noticeably, keratinocytes play a crucial role in psoriasis pathogenesis, recruiting neutrophils to the affected site through the release of chemotactic factors like CXCL1 and CXCL8 [3, 4]. Meanwhile, neutrophils trigger a vicious cycle of self-propagation in psoriasis through the formation and release of neutrophil extracellular traps (NETs), which in turn promote the development of chronic inflammation [5, 6]. Concurrently, a noticeable accumulation of CD68^+^ iNOS^+^ M1 macrophages is observed at the affected skin site in psoriasis patients, which leads to the release of TNF-α and further exacerbates disease progression [7]. These nuanced cell-to-cell interactions among keratinocytes, neutrophils, and macrophages collectively underpin the onset and advancement of psoriasis, yet the precise mechanisms governing their interplay remain unclear.

Lipocalin 2 (LCN2), a secretory cytokine derived from human neutrophils, liver cells, renal cells, bone marrow, adipose tissue, lungs, and macrophages, is involved in various biological processes, including antibacterial activity, cell differentiation and proliferation, and inflammation induction [8]. The human LCN2 protein exists in three distinct secreted forms: a monomeric 25 kDa structure, a 46 kDa homodimer, and a 135 kDa heterodimer complex formed with matrix metalloproteinase 9 (MMP9) [9]. So far, studies have revealed that LCN2 interacts with at least three functional receptors: 24p3R, megalin, and the melanocortin 4 receptor [10]. Elevated levels of LCN2 have been found in psoriasis, suggesting its potential involvement in the pathogenesis of psoriasis [8]. Studies have shown that LCN2 promotes the expression of pro-inflammatory cytokines in primary keratinocytes. Inhibition of the LCN2-specific receptor, 24p3R, has shown promising results in alleviating hyperkeratosis, infiltration of inflammatory cells, and excessive expression of inflammatory cytokines [11]. LCN2 is thought to both activate macrophages and inhibit M2 differentiation, thereby skewing macrophage differentiation towards the M1 subtype [12]. Apart from its macrophage activation role, LCN2 is implicated in prompting neutrophil infiltration and facilitating interactions between macrophages and neutrophils as a central mediator [13, 14]. Consequently, LCN2 is intricately linked to three fundamental cellular functions, significantly contributing to the progression of various diseases. However, the precise mechanisms through which LCN2 exerts these effects remain poorly clarified.

TWEAK (or TNFSF12) is a TNF superfamily member, produced by natural killer cells, macrophages, and dendritic cells [15]. TWEAK and its recognized receptor Fn14 (TNFRSF12A) are classified as members of the TNF and TNF receptor cytokine families, respectively. In recent years, CD163 has been identified as an additional scavenger receptor for TWEAK in many autoinflammatory diseases [16]. Fibroblast growth factor inducible-14 (Fn14, or TNFRSF12A) is essential for the development of psoriasis. When activated by TWEAK, Fn14 triggers signaling pathways that induce cell processes like proliferation, differentiation, migration, and apoptosis, as well as the expression of inflammatory mediators, leading to vasculogenesis and functional disturbance [17, 18]. Controlled or temporary activation of the TWEAK/Fn14 signaling pathway is involved in tissue restoration and rejuvenation, while uncontrolled or prolonged activation can cause detrimental inflammation and tissue impairment [19, 20]. TWEAK enhances the production of various factors, including VEGFA, IL-18, IL-23, HIF-1α, and Fn14, resulting in increased small blood vessels in the M5-cocktailed psoriatic cell model [17]. In murine models of colitis, the genetic knockout or neutralization of TWEAK leads to reduced infiltration of neutrophils and macrophages [21]. Conversely, TWEAK engagement with Fn14 upregulates CCL2, which promotes the recruitment of neutrophils and triggers an inflammatory response in a murine model of cerebral ischemic injury [22]. The activation of the TWEAK/Fn14 signaling pathway significantly contributes to the multifaceted progression of psoriasis [23], making it a promising target for therapeutic interventions to alleviate psoriasis.

Given that TWEAK is a soluble protein, it is primarily synthesized by immune cells and ubiquitously distributed in both serum and various tissues [24]. Similarly, LCN2, which exhibits a widespread presence in diverse tissue types, has been identified as a key player in facilitating breast cancer cell proliferation and migration via the nuclear factor of activated T-cells (NFAT1)-LCN2-TWEAK signaling cascade [25]. These findings suggest a potential synergistic relationship between LCN2 and TWEAK in modulating cellular proliferation and migration processes. Drawing upon the extensive tissue distribution patterns of both LCN2 and TWEAK, coupled with insights from our prior investigations, we propose that LCN2 collaborates with TWEAK to activate the Fn14 receptor, thereby fostering abnormal keratinocyte proliferation and triggering inflammatory responses.

In this study, we explored the intricate connections among LCN2, TWEAK, and Fn14. To achieve this, we utilized Lcn2 knockout (*Lcn2*^−/−^) and Fn14 knockout (*Fn14*^–/–^) mice, administered with recombinant LCN2 and TWEAK proteins, individually or in combination, to validate their biological interactions. Furthermore, utilizing RNA sequencing (RNA-seq) analysis of skin lesions from *Lcn2*^*-/-*^ mice treated with imiquimod (IMQ), we unveiled the role of LCN2 in the TWEAK/Fn14 signaling pathway and delineated downstream changes in genetic expression profiles. Our results point towards LCN2 amplifying TWEAK signaling through Fn14, with Lcn2 knockdown ameliorating the abnormal proliferation of keratinocytes and the inflammatory infiltration observed in IMQ-induced skin lesions, notably affecting neutrophils and macrophages. Likewise, Fn14 knockdown elicited a negative regulatory impact on LCN2 expression in the epidermis. These findings collectively suggest a collaborative synergy between LCN2 and TWEAK at the Fn14 receptor, propelling the advancement of psoriasis.

## Materials and methods

### Patients and samples collection

The research protocol for this study was ethically approved by the Medical and Ethics Committees of the Dermatology Department at the Second Affiliated Hospital of Xian Jiaotong University (No.2023499). Informed consent was obtained from all participants prior to their involvement. Skin samples (*n* = 7) and blood samples (*n* = 12) were collected from patients diagnosed with psoriasis vulgaris based on clinical and histological criteria at our hospital. These patients had not been treated with any systemic medicines within the past year. Additionally, normal control skin samples (*n* = 4) and sera (*n* = 10) were obtained from healthy volunteers who had undergone excision of pigmented nevus at our department of dermatological surgery.

### Animal models of psoriasis

All experiments conducted in this study followed the regulations set forth by the Ethics Committee of Xi’an Jiaotong University (Xi’an, China) (No.XJTUAE2023-2055). Female mice, aged 8-10 weeks, were used for the experiments. *Fn14*-deficient animals were bred on a Balb/c background, while *Lcn2*^–/–^ mice on a C57BL/6 background were purchased from the Jackson Laboratory. Corresponding WT littermates were used as control animals in all experiments. All experimental animals were housed in a specific pathogen-free environment. To induce a psoriasis-like disease model, the shaved backs of the mice were treated daily with 5% IMQ cream (Sichuan Mingxin Pharmaceuticals Co., China) or vehicle cream (Vaseline, Unilever Co., United States) for six consecutive days. In some experiments, animals were administered 8 ug (total volume 100 μL) recombinant mouse LCN2 (referred to as rmLCN2) via intraperitoneal injection three times weekly, or topically applied with 20 μg/mL (total volume 100 μL) recombinant mouse TWEAK (referred to as rmTWEAK) twice daily throughout the experiment. Prior to euthanizing the tissue, the mice were anesthetized with 50 mg/kg of sodium pentobarbital administered via intraperitoneal injection to alleviate pain.

### Bulk RNA-seq analysis

Bulk RNA-seq analysis was employed to investigate the synergistic effects of LCN2 and TWEAK in keratinocytes, as well as the role of *Lcn2* in *Lcn2*^–/–^ psoriasis mice models. In the synergistic effect experiments, keratinocytes were stimulated individually with LCN2 or TWEAK, or co-stimulated with both for 48 h, followed by RNA extraction for sequencing and analysis. The animal psoriasis models were established by administering IMQ to both WT mice and *Lcn2*^–/–^ mice. After a 6-day period, skin tissues were collected for analysis. In this study, transcriptome sequencing was performed using the Illumina sequencing platform. The Illumina PE library was constructed for 2 × 150 bp sequencing, and quality control was conducted on the obtained sequencing data. The input data consisted of Counts Per Million reads (CPM) values for each gene across all samples. To normalize the data, a *Z*-score was computed across all samples. The fold increase in gene expression (mean [AV] ± standard deviation) was calculated by comparing the RNA expression levels in the *Lcn2*^−/−^ mice group to the WT group. Differential gene expression analysis was performed using DESeq2 [26].

### Surface plasmon resonance (SPR)

The affinity between LCN2 and Fn14 was investigated using a Biacore T200 instrument (Biacore, Piscataway, NJ) for surface plasmon resonance analysis. A chip with a carboxymethyl dextran matrix coupler (Cytiva, USA) was used for immobilization of LCN2 on the sensor chip. LCN2 was incubated in PBS-P buffer at concentrations ranging from 0 to 2000 nM. The binding kinetic parameters, including the binding constant (K_a_) and dissociation constant (K_d_), were determined using a simplified Lanzivan model for data analysis.

### Cell culture and reagents

Human foreskin keratinocytes (HFKs) were cultured in Keratinocyte Medium (Sciencell, Carlsbad, CA). Human keratinocytes of the HaCaT cell line were cultured in RPMI 1640 medium (Gibco, Grand Island, USA) supplemented with 10% fetal bovine serum. Before stimulation assays, the keratinocytes underwent a 24-h serum-free starvation period. Subsequently, rmLCN2 (0–500 ng/mL; R&D Systems, Minneapolis, MN) and rmTWEAK (100 ng/mL; R&D Systems, Minneapolis, MN) were administered either separately or simultaneously, followed by stimulation with the M5 cocktail (including TNF-α, oncostatin M, IL-17A, IL-1α, IL-22 each at 10 ng/mL; ProteinTech, Wuhan, China) for 48 hours [17]. The macrophage J774A.1 cell line was obtained from Procell and maintained in DMEM medium (Procell Life Science & Technology Co, Wuhan, China). These cells were seeded in 6-well plates at a density of 2 × 10^5^ cells/mL and stimulated by adding rmLCN2 (0-50 ng/mL, R&D Systems) for 48 hours. Neutrophils were isolated from the femoral bone marrow of mice and suspended in RPMI 1640 medium (Gibco, Grand Island, USA). The cells were then seeded in 12-well plates at a density of 1×10^5^ cells/mL. Mouse rTWEAK (100 ng/mL, R&D Systems, Minneapolis, MN) was added to the culture for 24 h. Subsequently, the treated cells were harvested for RNA and protein extraction. Additionally, the cell supernatant collected for ELISA analysis.

### Proteomics

To identify changes in protein expression in keratinocytes after stimulation with TWEAK, the iTRAQ (isobaric tags for relative and absolute quantification) technology was employed following the methodology of Zhang et al. [27, 28]. The iTRAQ kit (CapitalBio Technology, Beijing, China) was used for this analysis. Protein samples from both untreated and TWEAK-treated keratinocytes underwent protein reduction, alkylation, and trypsin digestion. The samples were then labeled with iTRAQ reagents. After vacuum drying, the iTRAQ-labeled samples were subjected to further analysis using strong cation exchange chromatography. The peptide digest obtained from the fractionation using strong cation exchange (SCX) chromatography was analyzed using the Dionex Nano-UPLC System and Q Exactive Mass Spectrometer, both manufactured by Thermo Fisher Scientific.

### Histology and immunohistochemistry

Dorsal skin samples were obtained from the mice and subsequently subjected to formalin fixation. The fixed samples were then embedded in paraffin. Four-micrometer sections were prepared from the paraffin-embedded samples and subjected to standard hematoxylin and eosin (HE) staining protocols. Epidermal thickness was subsequently measured from the HE-stained sections. For immunohistochemistry (IHC) analysis, dewaxed tissue sections underwent a series of steps. Initially, they were immersed in boiling citrate buffer (0.01 M, pH 6.0) for 10 min, followed by cooling to room temperature. Afterwards, the sections were treated with 3% hydrogen peroxide for 15 min, blocked with 2% BSA diluted in PBS for 30 min, and then incubated with a diluted rabbit anti-LCN2 (or TWEAK, Fn14, KRT1, KRT5, KRT10, KRT14, KRT17, Involucrin) (2 µg/mL; Abcam, Cambridge, MA) or anti-Ly6G, CD45 (2 µg/mL; Servicebio, China) IgG overnight at 4 °C. On the following day, the DAKO REAL EnVision Detection System, Peroxidase/DAB + (DAB) kit (DAKO, Glostrup, Denmark) was used for further processing. Prior to dehydration and mounting, counterstaining with hematoxylin was performed. Microscopic images of the stained slides were acquired using a Nikon microscope (Tokyo, Japan). The integrated optical density of each image was measured and calculated using Image Pro Plus software version 6.0 (Media Cybernetics, Rockville, MD).

### Immunofluorescence staining

HFKs were cultured on coverslips and stimulated with either rmLCN2 or PBS, then fixed, permeabilized, and subjected to staining using TWEAK antibody sourced from Novus Biologicals (CO, USA). Neutrophils were isolated from mouse marrow and cultured on coverslips and stimulated by rmTWEAK. Then incubation was performed using LCN2 antibody which obtained from Abcam. To visualize TWEAK and LCN2 separately, anti-goat IgG secondary antibodies conjugated with CoraLite 594 PEX14 antibody from ProteinTech were used. Nuclei were counterstained with DAPI. Finally, the images were captured and analyzed using an inverted confocal microscope (Olympus, Tokyo, Japan).

### Multiplex immunohistochemical (mIHC)

Skin lesions from psoriasis patients were paraffin-embedded and sectioned as soon as possible. Paraffin-embedded sections of psoriatic skin lesions from patients were subjected to dewaxing and antigen retrieval, followed by incubation with primary antibodies against TWEAK (10 µg/mL; Abcam, Cambridge, MA), CD68 (1.8 µg/mL; Abcam, Cambridge, MA), CD66b (0.26 µg/mL; Abcam, Cambridge, MA), and LCN2 (10 µg/mL; Abcam, Cambridge, MA). Paraffin-embedded sections of skin lesions from mice were incubated with antibodies against TWEAK and F4/80 (0.5 µg/mL; Servicebio, China). After incubation with secondary antibody (biotin conjugated affiniPure goat anti-rabbit IgG, 1:1000, Boster, China), the sections were stained using tyramide signal amplification (TSA) dyes 620 and 555 (1:200, MCE, USA). Images were captured using a confocal microscope (Olympus, Tokyo, Japan), and subsequent data analysis was performed using ImageJ software.

### ELISA

The reagent kit used for measuring LCN2 level in human serum and IL-17A and TNF-α level in mice serum and skin was provided by ProteinTech (Wuhan, China). The levels of IL-23A in mouse serum and skin were measured using the reagent kit purchased from Elabscience (Wuhan, China). The reagent kits for detecting TWEAK and LCN2 levels in mouse serum and cell culture supernatants were respectively purchased from Fine Biotech (Wuhan, China) and YoBiBiotech (Shanghai, China).

### Western blotting

The cellular protein extracts were subjected to gel electrophoresis followed by transfer onto a polyvinylidene difluoride membrane (Millipore, Billerica, MA). Rabbit antibodies to KRT1, KRT5, KRT10, KRT14, KRT17, Loricrin, TWEAK and LCN2 (2 μg/mL) were purchased from Abcam. Rabbit antibodies to KRT6, KRT16, and Fn14 (200 μg/mL) were bought from Santa Cruz Biotechnology (Santa Cruz, CA). p-ERK1/2, ERK1/2 and, GAPDH (1:1000) were purchased from Cell Signaling Technology (CST, MA, USA). CXCL10 (1:1000) were bought from GeneTex (GeneTex, Shanghai, China). A secondary antibody, consisting of horseradish peroxidase-conjugated goat anti-rabbit IgG at a concentration of 0.2 μg/mL (Absin, Beijing, China), was employed. Signal detection of reactive proteins was accomplished through the utilization of an enhanced chemiluminescence kit (Merck Millipore, Billerica, MA). The protein band intensities were quantified using the ImageJ software. The primary antibodies used in the study can be found in Table S3.

### Quantitative PCR

Total RNA was isolated from tissues or cells using TRIzol reagent (Invitrogen, Carlsbad, CA). Subsequently, the total RNA was reverse transcribed into cDNA using a cDNA kit (Takara Bio, Kyoto, Japan), followed by PCR amplification and quantification using SYBR Green Master Mixes (Takara Bio, Kyoto, Japan). The PCR primers were purchased from Tsingke Biotechnology (Beijing, China). The primer sequences can be found in Table S4.

### Co-immunoprecipitation (Co-IP)

HaCaT cells were cultured in vitro and stimulated with rmLCN2 for 48 h to induce Fn14 protein overexpression. After cell collection, total protein was extracted and incubated with mouse anti-human Fn14 antibody (1:50, Santa Cruz, CA) conjugated to protein A/G magnetic beads (MCE, USA). The beads were washed, and the bound proteins were eluted for Western blotting analysis to detect LCN2 and Fn14 proteins. For Western blotting, a rabbit anti-human Fn14 antibody (1:1000, CST, MA, USA) was used as the primary antibody. At the same time, another method was used for verification. Fn14 was overexpressed in HaCaT cells using a lentiviral vector (LV-hTNFRSF12A-3FLAG-OE, Biopuxin, China). After LCN2 stimulation, cells were collected, and Fn14 protein was enriched using anti-Flag magnetic beads. The beads were eluted, and Western blotting was performed to detect LCN2 and Fn14 proteins.

### Statistical analysis

Statistical analyses were performed using GraphPad Prism 10 software (San Diego, CA). Error bars in the qPCR experiments represent the standard error of the mean (SEM) derived from three or more independent experiments. Student’s *t* test was used for comparison within two groups and one-way ANOVA were used for multiple comparisons. A significance level of *P* < 0.05 was considered statistically significant.

## Results

### LCN2 expression is positively correlated with TWEAK and Fn14

**Fig. 1:**
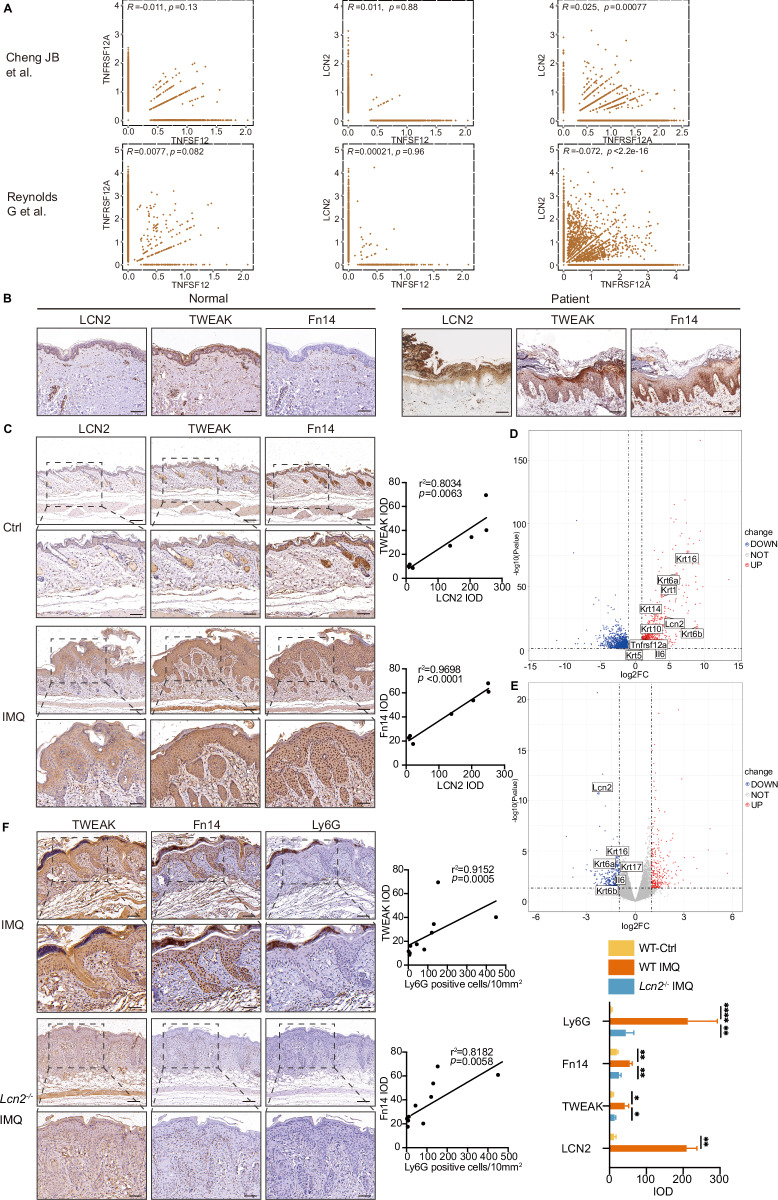
TWEAK, Fn14, and LCN2 are overexpressed in skin lesions of psoriasis patients and IMQ induced psoriatic mice model. **A** Single cell data from psoriasis was used for correlation analysis between *TWEAK, Fn14* and *LCN2*. **B** Immunostaining of LCN2, TWEAK and Fn14 in the skin of healthy control (HC) and patient with psoriasis (Brown). Normal control skin samples = 4, patient skin samples = 4. Nuclei were stained with hematoxylin (Bar = 200 μm). **C** Immunostaining of LCN2, TWEAK and Fn14 were performed on wild-type mouse skin paraffin sections. Stained epidermal region areas were quantitated by ImageJ software. The relationship between the IOD of TWEAK (or Fn14) and LCN2 staining was assessed by Spearman’s rank correlation. Number of control mice = 3, Number of IMQ induced WT mice = 4. Representative images are shown (Bar = 200 μm). **D** Volcano Plot showed the RNA-seq analysis of wild-type control mice and wild-type imiquimod model mice. **E** RNA-seq analysis of wild-type mice and *Lcn2*^–/–^ mice treated with imiquimod. **F** Immunostaining of TWEAK, Fn14 and Ly6G were performed on Lcn2 knockout mice skin paraffin sections. The relationship between the IOD of TWEAK (or Fn14) and number of Ly6G positive cell staining was assessed by Spearman’s rank correlation. Number of control mice = 3, Number of IMQ induced WT mice = 4, Number of IMQ induced *Lcn2*^–/–^ mice = 4. Representative images are shown (Bar = 200 μm)

To investigate the expression of TWEAK, Fn14, and LCN2 in psoriasis, we did the correlation analysis among them using two single cell RNA sequencing (scRNA-seq) data of psoriasis from Cheng et al. and Reynolds et al. [29, 30]. The data of Reynolds et al. showed that the expression level of *TNFSF12* (or *TWEAK)* in keratinocytes in psoriasis lesions was positively correlated with the expression of *TNFRSF12A* (or *Fn14)* (*R* = 0.0077, *P* = 0.082), which was consistent with the results of our previous study [19], while *TWEAK* was positively correlated with *LCN2* but the transcripts demonstrating Spearman correlation was not significant, and Fn14 was negatively correlated with the expression level of *LCN2* (*R* < 0.6, *P* < 2.1 3 10^–344^, 43/16,343 assessed genes). In the study of Cheng et al., the data showed *TWEAK* was negatively correlated with *Fn14* gene expression level (*R* = -0.011, *P* = 0.13), while *LCN2* was positively correlated with *TWEAK* (*R* = 0.011, *P* = 0.88) and *Fn14* (*R* = 0.025, *P* = 0.00077), respectively (Fig. 1A). From these, we can see that there are big differences in the studies analyzing the correlation among LCN2, TWEAK, and Fn14 in different data, so the relationship between the three needs to be further explored. We then performed IHC and found increased TWEAK, Fn14, and LCN2 in psoriatic skin lesions compared with healthy controls (HC) (Fig. 1B, Fig S1A). We therefore examined the lesions from IMQ-induced murine model to validate the spatial expression of TWEAK, Fn14, and LCN2. By IHC, we found that LCN2, TWEAK and Fn14 expression was significantly elevated in skin lesions of IMQ mice, while TWEAK and Fn14 were positively correlated with LCN2 expression levels (Fig. 1C), consistent with psoriasis patients (Fig. S1B).

We then performed bulk RNA-seq to assess the gene expression changes in the IMQ group compared to the control group. Our analysis revealed a broad transcriptional response in mice following IMQ induction, with 1395 genes upregulated and 1500 genes downregulated, with fold changes exceeding two fold (Fig. 1D). Notably, the genes associated with inflammation (*Lcn2*, *Il6*, *Tnfrsf12a* (*Fn14*)), marker of neutrophil (*Ly6g*), keratinocyte proliferation (*Krt5*, *Krt6*, *Krt14*, *Krt16*), and keratinocyte differentiation (*Krt1, Krt10*) were upregulated after IMQ treatment, suggesting the potential roles of LCN2 in these changes.

To further investigate the effects of LCN2 on psoriasis, we utilized *Lcn2*^–/–^ mice and subjected them to IMQ treatment for 6 days. Compared to the WT IMQ model, *Lcn2* gene knockout in the skin lesions resulted in the upregulation of 590 genes and the downregulation of 564 genes, with fold changes exceeding twofold (Fig. 1E). Among the downregulated genes were *Krt6, Krt16*, *Krt17*, *Il6* and *Lcn2*, same as human bulk-RNA results. While the expression of *Krt1*, *Krt5*, *Krt10*, *Krt14*, *Il23a*, *Tnfa* and *Tnfrsf12a* (*Fn14)* genes was downregulated, the differences were not statistically significant. Furthermore, the protein levels of TWEAK, Fn14, and Ly6G were significantly reduced in the epidermis of *Lcn2*^−/−^ mice compared to WT mice, and the expression levels of TWEAK and Fn14 were positively correlated with those of Ly6G (Fig. 1F). In summary, these experiments show that the expression of TWEAK and Fn14 are correlated with the level of LCN2 and the neutrophil marker Ly6G.

### Expression and localization of TWEAK and LCN2 and the interaction between LCN2 and Fn14

**Fig. 2:**
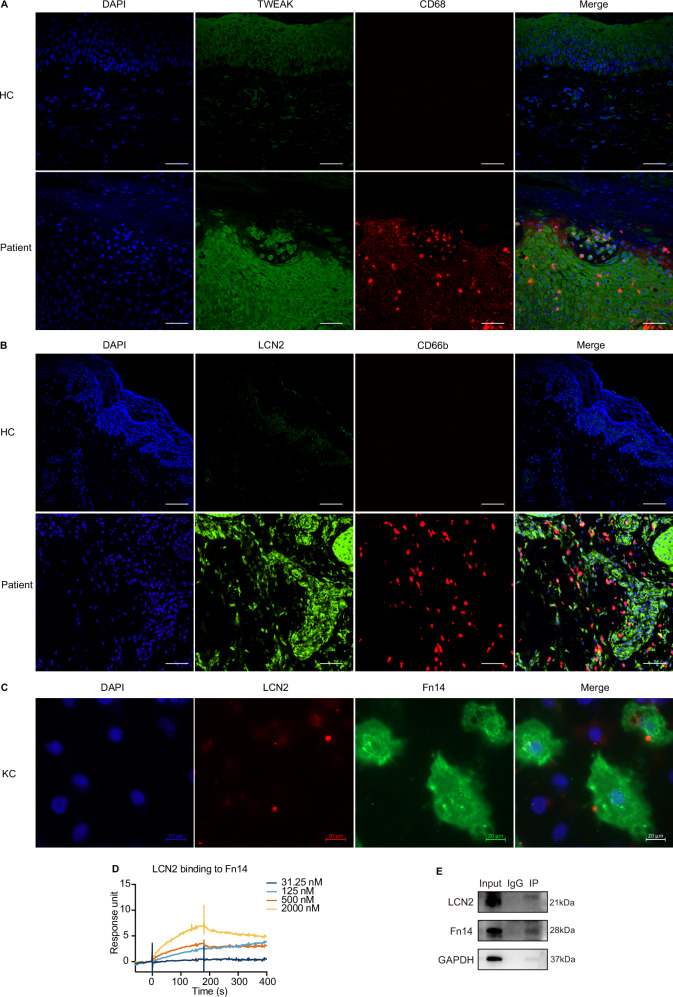
Expression localization of TWEAK and LCN2 and the interaction between LCN2 and Fn14. **A** Tissue mIHC results showing the cellular localization of TWEAK expression and its relationship with CD68-labeled macrophages; **B** tissue mIHC results showing the cellular localization of LCN2 expression and its relationship with CD66b-labeled neutrophils (Bar = 200 μm); **C** cellular immunofluorescence results showing the colocalization of LCN2 and Fn14; **D** the binding affinity of LCN2 conjugate to the Fn14 molecule was determined by SPR analysis. **E** Co-IP experiment detecting the interaction between LCN2 and Fn14 after stimulating Hacat cells cultured in vitro with LCN2

To further confirm the cellular expression of TWEAK and Fn14, we analyzed the expression patterns of TWEAK, LCN2 and Fn14 using scRNA-seq data from Cheng et al. and Reynolds et al. The results revealed that *TWEAK* gene is expressed in differentiated keratinocytes, fibroblasts and macrophages, while *LCN2* is mainly expressed in neutrophils, fibroblasts, and keratinocytes (Fig. S2). Additionally, *Fn14* gene is highly expressed in proliferating keratinocytes, undifferentiated keratinocytes, differentiated keratinocytes, macrophages, and fibroblasts (Fig. S2). mIHC was applied to investigate the cellular expression of TWEAK and LCN2 in tissue samples. We observed that, compared to normal controls, TWEAK was highly expressed in macrophages, LCN2 was highly expressed in neutrophils, and both TWEAK and LCN2 were highly expressed in keratinocytes within the lesional skin of psoriasis patients. (Fig. 2A, B).

Subsequently, we stimulated HaCaT cells overexpressing Fn14 (via lentiviral transfection) with recombinant human LCN2(rhLCN2) active protein and performed immunofluorescence staining to localize LCN2 and Fn14 proteins. We found that LCN2 protein co-localized with Fn14 on the cell membrane of keratinocytes, suggesting a potential interaction between LCN2 and Fn14 (Fig. 2C). To further investigate this interaction, we conducted SPR experiments, which demonstrated that LCN2 has binding affinity for Fn14 (Ka= 667003.7353 M^−1^S^−1^, Kd = 0.162054553 S^−1^, KD = 2.43 × 10^–7^ M) (Fig. 2D), but not for TWEAK (data not shown). To validate the SPR results, we performed Co-IP experiments, which confirmed that Fn14 can bind to LCN2 (Fig. 2E).

### LCN2 promotes macrophage differentiation and TWEAK secretion

**Fig. 3:**
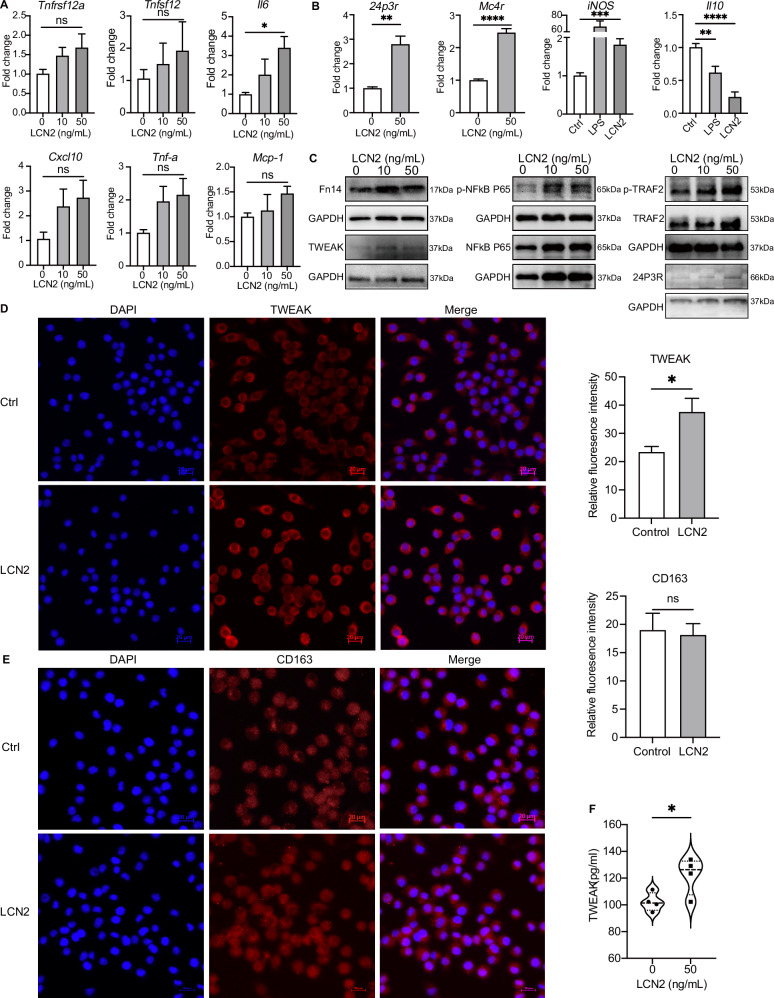
LCN2 induces the differentiation and secretion of TWEAK in macrophages. **A**, **B** *Tnfsf12*, *Tnfrsf12a*, and *Il6* were detected by RT-qPCR after treating with 0, 10, 50 ng/mL LCN2. *24p3r*, *Mc4r*, *inos*, and *Il10* mRNA in J774A.1 cell were detected by RT-qPCR after treated with 50 ng/mL LCN2. **C** The intracellular levels of Fn14, TWEAK, p-NFκB P65, p-TRAF2, and TRAF2 proteins were detected by Western blotting after stimulation of J774A.1 cell with LCN2 at concentrations of 0, 10, 50 ng/mL, respectively. **D**, **E** TWEAK and CD163 was detected by immunofluorescence in J774A.1 cell after LCN2 treatment. Bar = 100 μm. **F** Elisa was used to detect the concentration of TWEAK in cell supernatants 24 h after LCN2 stimulation of J774A.1. Data from immunofluorescence assays and Elisa assays are representative of three or more independent experiments are presented as the means ± SEM of triplicate wells. qPCR data are presented as the means ± SEM for triplicate wells from three or more independent experiments. ANOVA was used for comparison between groups: *P < 0.05, **P < 0.01, and ***P < 0.001. ns not significant

We then investigated the underlying pathogenic mechanisms of LCN2, TWEAK, and Fn14 involved in psoriasis. Since TWEAK is secreted by macrophages, we first explored the effects of LCN2 on macrophages. We stimulated mouse macrophage cell line J774A.1 with multiple concentration of recombinant mouse LCN2 (rmLCN2) in vitro. qPCR revealed the gene levels of *Tnfrsf12a*, *Tnfsf12*, *Il6* showed concentration-dependent responses to rmLCN2 stimulation, at doses ranging from 10 to 50 ng/mL (Fig. 3A). The expression of the LCN2 receptors *24p3r* and *Mc4r*, as well as the M1 macrophage marker *inos*, significantly increased after 50 ng/mL rmLCN2 stimulation, while the expression of the anti-inflammatory cytokine *Il10* significantly decreased (Fig. 3B). Additionally, the protein levels of Fn14, TWEAK, p-NFκB P65, p-TRAF2, and TRAF2 also showed significant elevation in rmLCN2 treated macrophages by Western blotting (Fig. 3C). Immunofluorescence results revealed an increase in TWEAK immunoreactivity (Fig. 3D) but unchanged CD163 expression in macrophages after rmLCN2 stimulation (Fig. 3E). However, the cell culture supernatant showed a significant increase in TWEAK expression via ELISA (Fig. 3F), indicating that LCN2 can promote M1 differentiation and the extracellular secretion of TWEAK, thereby exacerbating inflammation in psoriasis.

### LCN2 promotes TWEAK and Fn14 expression and activates MAPK pathway in keratinocytes

**Fig. 4:**
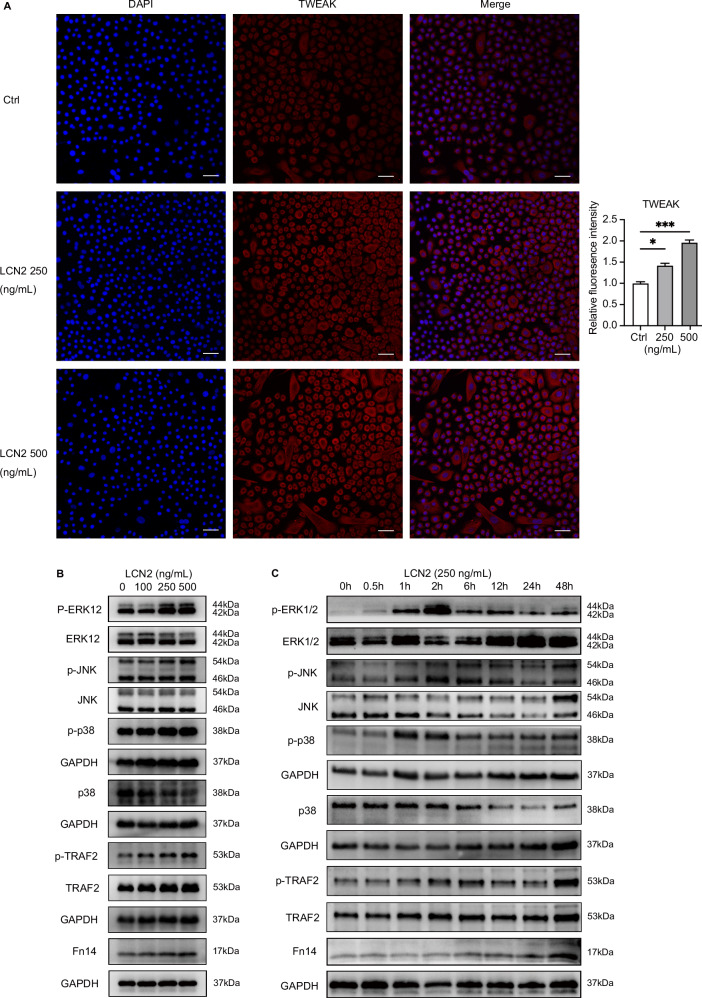
LCN2 regulates the expression of TWEAK and Fn14 and MAPK pathway in keratinocytes. **A** Immunofluorescence was used to determine TWEAK level after treatment with LCN2 in HFKs. Bar = 50 μm. **B** Western blotting was used to detect the expression of p-ERK1/2, p-JNK, p-p38, p-TRAF2 and Fn14 in primary keratinocytes after treating the cells with 0, 100, 250 and 500 ng/mL LCN2, respectively. **C** After the addition of 500 ng/mL LCN2 stimulation, keratinocytes were collected at 0, 0.5, 1, 2, 6, 12, 24 h for WB assay to detect p-ERK1/2, p-JNK, p-p38, p-TRAF2 and Fn14 expression over time. Data from immunofluorescence assays and immunoblot assays are representative of three or more independent experiments are presented as the means ± SEM of triplicate wells

Our previous studies found Fn14 is highly expressed in keratinocytes [17], we then looked into the roles of LCN2 in keratinocytes. HFKs were stimulated with rhLCN2 which promoted the expression of TWEAK in keratinocytes in a dose-dependent manner, when assayed by immunofluorescence (Fig. 4A). Moreover, we observed time and concentration-dependent increase in the protein levels of Fn14 following rhLCN2 stimulation, as shown in Fig. 4B, C. As our previous studies revealed that TWEAK can activate ERK to promote cell proliferation and inflammation [31], we then investigated MAPK pathway after rhLCN2 stimulation and found time and concentration-dependent increases of p-ERK1/2, p-JNK, p-p38, p-TRAF2 and Fn14 (Fig. 4B, C). Since TWEAK/Fn14 can activate ERK pathway [32], we therefore speculated that LCN2 may further activate the ERK1/2 pathway through the upregulation of TWEAK and Fn14 to promote the secretion of inflammatory factors and participate in the abnormal proliferation and differentiation of keratinocytes.

### TWEAK promotes neutrophil expression of LCN2 but enhances inflammation in keratinocytes by inducing MMP9 expression

**Fig. 5:**
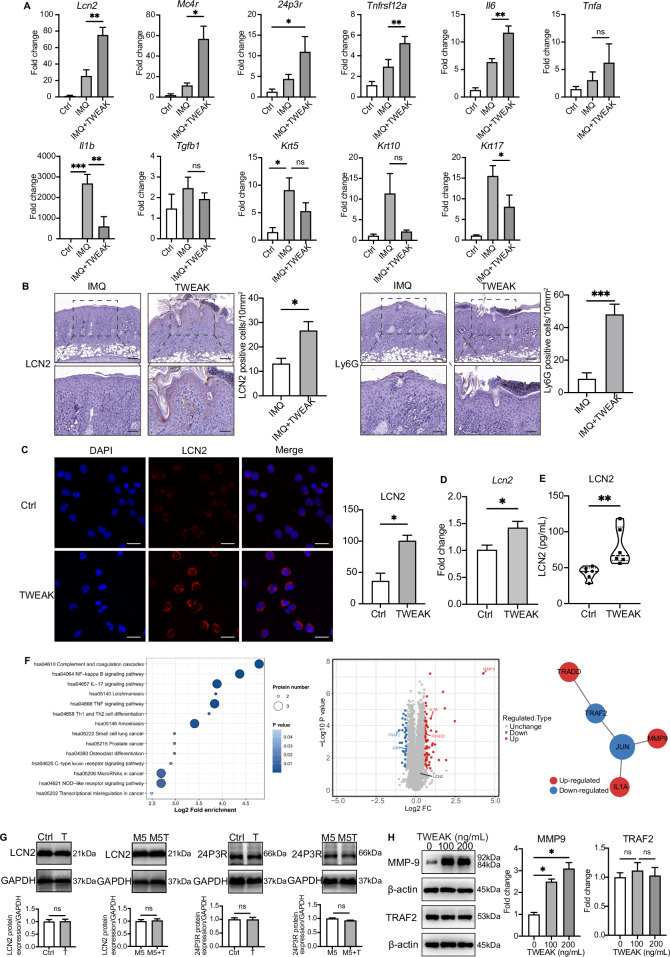
TWEAK promotes neutrophil expression of LCN2 but enhances inflammation in keratinocytes by inducing MMP9 expression. **A** Relative mRNA expression level of *Lcn2, Tnfrsf12a*, *Tgfb1, Il6, Tnfa, Il1b, Krt5, Krt10, Krt17, 24p3r, Mc4r* in mouse skin treated with TWEAK was detected by RT-qPCR. **B** Immunostaining of LCN2 and Ly6G were performed on paraffin sections of IMQ mice model with or without dorsally topical application of rmTWEAK (20 μg/mL, prepared in PBS). Unpaired two-tailed *t* test. **C** Immunofluorescence assays were used to detect LCN2 expression in neutrophils after TWEAK stimulation. Bar = 20 μm. **D** RT-qPCR was conducted to detect *Lcn2* mRNA level in neutrophils treated with TWEAK. **E** LCN2 levels in neutrophil supernatants after TWEAK stimulation for 24 h were measured by ELISA. **F** Proteomic analysis was used to further investigate the protein changes and possible pathways following TWEAK action on keratinocytes. **G** After treating with TWEAK on primary keratinocyte with or without M5 cytokines for 48 h, LCN2 and 24P3R protein levels in keratinocytes were detected by Western blotting. **H** After the TWEAK stimulus, proteins such as MMP9 and TRAF2 were detected by Western blotting. Data from immunofluorescence assays and ELISA assay are representative of three or more independent experiments are presented as the means ± SEM of triplicate wells. ANOVA was used for comparison between groups: *P < 0.05, **P < 0.01, and ***P < 0.001. ns not significant

Researchers have identified the impact of TWEAK on the infiltration of neutrophils and macrophages [21]. Given that LCN2 is primarily secreted by neutrophils, we sought to explore the interplay between TWEAK and LCN2 specifically within the context of neutrophil activity. To achieve this, we applied rmTWEAK to the skin of WT mice treated with IMQ. qPCR analysis revealed significant elevation in the mRNA levels of *Lcn2*, *Tnfrsf12a*, *24p3r*, *Mc4r*, and *Il6* in the mouse skin following rmTWEAK stimulation (Fig. 5A). There was a trend for *Tnfa* induction, though not significantly different. In contrast, *Il1b*, *Krt1*0, and *Krt17* mRNA levels significantly decreased, whereas *Krt5* and *Tgfb*1 expression remained unchanged (Fig. 5A). IHC results displayed increased neutrophil counts and LCN2 expression in the dermal tissue upon TWEAK stimulation compared to the imiquimod-induced mice model (Fig. 5B). To validate the effect of TWEAK on neutrophils in vitro, we isolated neutrophils from mouse bone marrow and stimulated them with 100 ng/mL rmTWEAK for 24 h. Through cell immunofluorescence, we observed a significant increase in intracellular LCN2 expression in mouse neutrophils following rmTWEAK stimulation (Fig. 5C). qPCR analysis also indicated a significant elevation in *Lcn2* mRNA levels in neutrophils (Fig. 5D). Further confirmation came from ELISA detection of LCN2 concentration in the cell supernatant and a notable increase in LCN2 after TWEAK stimulation was observed (Fig. 5E). Consequently, we conclude that TWEAK enhances neutrophil infiltration in skin lesions and LCN2 release from neutrophils.

We also examined whether TWEAK has an effect on LCN2 in epidermal keratinocytes. HFKs were cultured both under normal conditions and under a psoriatic M5 cytokine cocktail [33] plus TWEAK. Following Western blot analysis, our findings indicate that TWEAK failed to induce LCN2 and 24P3R expression in keratinocytes, irrespective of the presence of the M5 cytokine cocktail (Fig. 5G). To further study the role of TWEAK on keratinocytes, we conducted the proteomic analysis (Fig. 5F). The volcano plot demonstrated altered expression levels of 99 proteins, with increased expression in 66 proteins and decreased expression in 33 proteins but found no significant difference in LCN2 protein levels between the experimental groups (Table S1). Kyoto encyclopedia of genes and genomes (KEGG) analysis based on proteomic data from keratinocytes with TWEAK stimulation revealed enriched pathways, mainly including IL-17 and NF-κB pathways. This leads us to conclude that TWEAK stimulation on its own does not induce LCN2 and 24P3R expression in keratinocytes. Subsequently, we focused on the differentially expressed proteins associated with the TNF signaling pathway by activating MMP9, IL-1α and TRADD and decreasing the expression of TRAF2 and JUN proteins which lead to cell apoptosis (Fig. 5F). MMP9 can bind to LCN2 and participate in various inflammatory diseases [34]. Studies have found that LCN2 promotes the expression of MMP9 and maintains its stability by preventing degradation [35], indicating a close relationship between MMP9 and LCN2. Our Western blotting results demonstrate that TWEAK can enhance the expression of MMP9, but no significant difference was observed in the expression of TRAF2 (Fig. 5H). From these results we concluded that LCN2 can upregulate TWEAK levels in psoriatic lesion.

### The synergistic interaction between LCN2 and TWEAK in keratinocytes

**Fig. 6:**
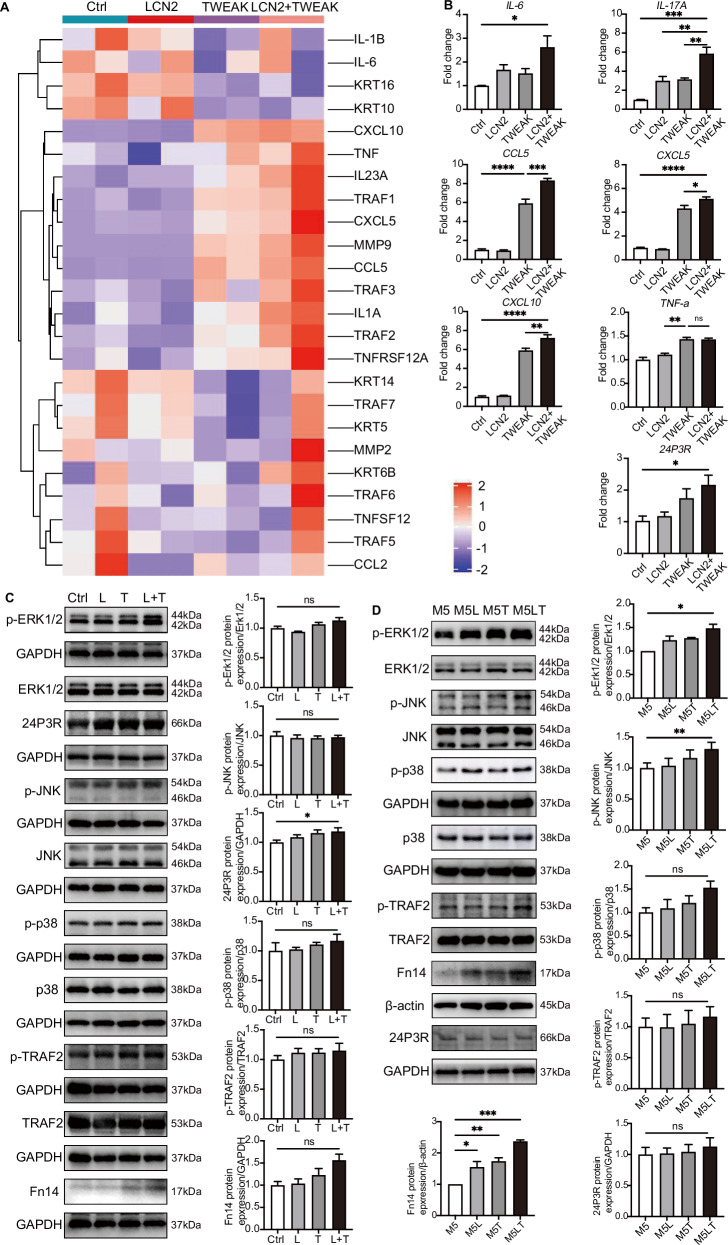
The synergistic interaction between LCN2 and TWEAK in keratinocytes. **A** Heatmap showing differential expressed genes after co-stimulating with TWEAK and LCN2 in keratinocytes. **B** Relative expression levels of *IL6*, *TNFa*, *IL17A, CCL5, CXCL5, CXCL10* and *24P3R* were detected from HFKs using RT-qPCR. **C**, **D** Western blot was used to detect p-ERK1/2, p-JNK, p-p38, p-TRAF2, 24P3R and Fn14 protein level in keratinocytes after stimulation with LCN2, TWEAK, or both. One-way ANOVA was used for comparison between groups: ns (*P* > 0.05); *P < 0.05; **P < 0.01; ***P < 0.001

To delve deeper, we introduced rhTWEAK and rhLCN2 independently, as well as in combination. RNA-seq results revealed that TWEAK and LCN2 exhibit synergistic effects in promoting the expression of inflammatory factors such as *IL23A*, chemokines including *CXCL5*, *CXCL10*, *CCL2*, and *CCL5*, as well as members of the *TRAF* family and *TNFRSF12A(Fn14)* in keratinocytes (Fig. 6A). qPCR result showed that *IL6*, *IL17A*, *CCL5*, *CXCL5*, *CXCL10*, *TNFa* and *24P3R* gene levels upregulated after co-stimulation of TWEAK and LCN2 (Fig. 6B). Furthermore, we conducted co-stimulation experiments based on with or without M5 cytokine cocktail activation. We observed that TWEAK facilitated the enhancement 24P3R, p-TRAF2 expression and triggered the activation of the MAPK signaling pathway in keratinocytes when coupled with M5 cytokines and LCN2 (Fig. 6C, D). Interestingly, the combined action of LCN2 and TWEAK resulted in a more pronounced upregulation of Fn14 (Fig. 6C, D), suggesting a synergistic effect between these factors in enhancing Fn14 expression in keratinocytes. Overall, these results demonstrate that LCN2 and TWEAK collaboratively promote Fn14 expression in keratinocytes, which together promotes the pathogenesis of psoriasis.

### *Lcn2* knockout alleviates psoriatic appearance in vivo

**Fig. 7:**
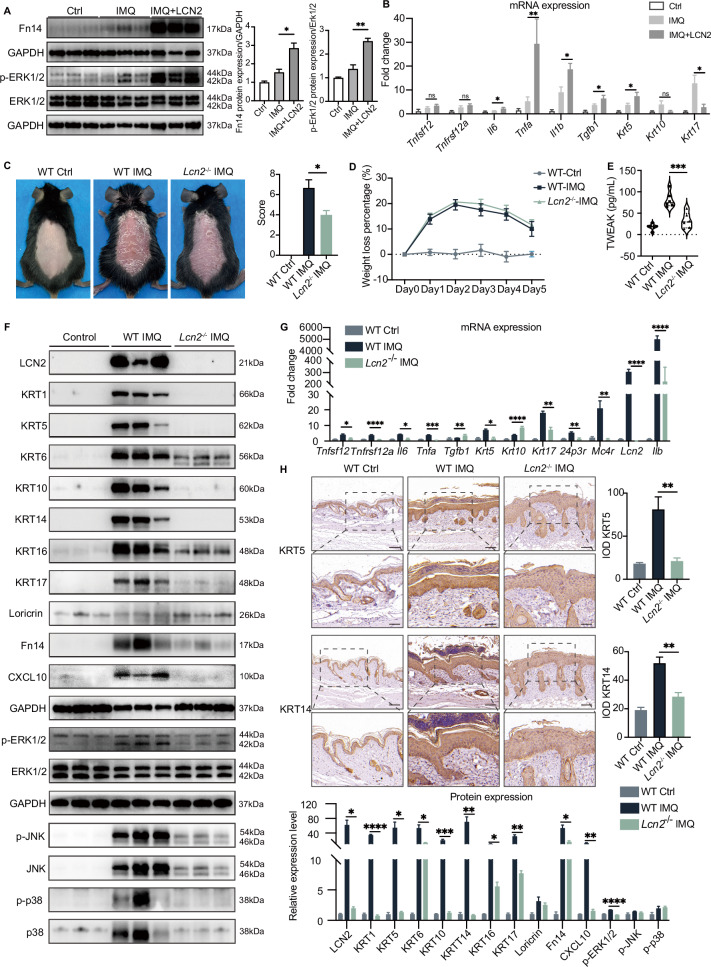
*Lcn*2^–/–^ mice attenuate the pro-proliferative and inflammatory effects of IMQ on the epidermis. **A** After being treated with imiquimod and LCN2, the expression levels of Fn14, p-ERK1/2 and in the skin of wild-type mice were analyzed by Western blotting (*n* = 3 per group). **B** Relative expression levels of *Tnfsf12(Tweak)*, *Tnfrsf12a(Fn14)*, *Il6*, *Tnfa*, *Il1b*, *Tgfb1*, *Krt5*, *Krt10*, and *Krt17* from mice skin were detected using RT-qPCR. **C**, **D** Photographs of skin lesions in mice and the mouse psoriasis severity score difference between wild-type imiquimod mice and *Lcn2*^–/–^ imiquimod model mice. Body weight changes in mice modeled with imiquimod for six consecutive days. **E** Mouse serum levels of TWEAK were assayed by ELISA. **F** Western blotting was used to detect KRT1, KRT5, KRT6, KRT10, KRT14, KRT16, KRT17, Loricrin, Fn14, CXCL10, LCN2, p-ERK1/2, p-JNK, p-p38 in wild-type and *Lcn2*^–/–^ mice after treated with imiquimod (*n* = 3 per group). **G** Relative mRNA expression levels of *Lcn2*, *Tnfsf12*, *Tnfrsf12a*, *Il6*, *Tnfa*, *Tgfb1*, *Krt5*, *Krt10*, *Krt17*, *24p3r*, *Mc4r*, *Il1b* were detected by RT-qPCR (*n* = 3–5 per group). **H** Immunostaining of KRT5 and KRT14 were performed on paraffin sections. Data are shown as mean ± SEM (*n* = 3–5 per group). ANOVA was used for comparison between groups: ns (*P* > 0.05); *P < 0.05; **P < 0.01; ***P < 0.001

The potential involvement of LCN2 in the regulation of inflammation, proliferation, and differentiation of keratinocytes has prompted us to investigate its role in vivo. To do this, we employed the IMQ psoriatic model and intraperitoneally administered 8 µg rmLCN2 to each mouse per day and compared these mice with those from the IMQ model intraperitoneally treated with PBS. Our findings reveal that LCN2 promotes the expression of Fn14 (*P* = 0.0148) in the skin of rmLCN2-administrated mice, consequently activating downstream ERK1/2 pathways (p-ERK1/2, *P* = 0.0051; Fig. 7A). qPCR results depicted significant upregulation of *Il6, Tnfa, Il1b, Tgfb1*, and *Krt5* gene levels following LCN2 stimulation (Fig. 7B), while *Tnfsf12* and *Tnfrsf12a* gene levels displayed an upward trend without statistical significance. Conversely, *Krt10* and *Krt17* gene levels were down-regulated (Fig. 7B). This finding suggests that LCN2 might facilitate inflammation and promote the keratinocytes differentiation within murine psoriatic lesions.

Furthermore, we conducted a comparative study on the effects of IMQ application on WT and *Lcn2*^–/–^ mice. Notably, *Lcn2*^–/–^ mice exhibited remarkable reductions in skin lesion thickness (Fig. S3), desquamation, and erythema, along with a decrease in the mouse psoriasis severity score (*P* = 0.0209, Fig. 7C, D) [36], without any impact on body weight (the detailed scoring criteria are provided in Table S5). To unveil the underlying mechanisms, we performed bulk RNA-seq analysis from the skin of IMQ-treated *Lcn2*^–/–^ and WT mice to assess differential gene expression (DEGs). The KEGG pathways from the upregulated DEGs revealed significant cytokine-cytokine receptor interaction and cell adhesion molecules in the IMQ-treated WT group (Fig. S4A), which were consistent with findings from human psoriasis results which we analyzed RNA-seq data uploaded by Rønholt et al. on skin lesions from normal subjects and patients with psoriasis (GSE161683, paper is not published) (Fig. S5). Furthermore, the gene ontology (GO) analysis identified terms associated with epidermal development, leukocyte migration, and cytokine activity, aligning well with the human psoriasis data. Subsequent analysis of IMQ-treated WT and *Lcn2*^–/–^ mice highlighted the significant involvement of *Lcn2* deletion in terms associated with leukocyte migration, cytokine and growth factor activation, and binding (Fig. S4B). Simultaneously, we observed that the number of CD45^+^ leukocytes in the epidermal and subcutaneous tissues of mice was significantly reduced after *Lcn2* knockout (Fig. S6). Consequently, the deletion of *Lcn2* appears to modulate leukocyte migration and have a regulatory effect on the release of inflammatory cytokines, leading to the alleviation of psoriasis-like skin lesions.

To validate the observed differences, TWEAK, IL-23A, IL-17A, and TNF-α levels in serum and skin lysate between IMQ-treated WT and *Lcn2*^–/–^ mice were evaluated by ELISA. The results demonstrated a significant decrease in TWEAK (*P* = 0.0002, Fig. 7E) levels in *Lcn2*^–/–^ mice serum compared to WT mice. Moreover, we observed a significant reduction in the expression of IL-17A in both the serum and skin lysate (Fig. S7A, B). However, no significant differences were observed in the expression levels of IL-23A and TNF-α in the serum and skin lysate of mice between the two groups (Fig. S7A, B). Western blotting further revealed decreased expression of KRT1, KRT5, KRT6, KRT10, KRT14, KRT16, KRT17, Fn14, and CXCL10 in IMQ-treated *Lcn2*^–/–^ mice (Fig. 7F). Additionally, the expression of p-ERK1/2 and p-JNK, key transcription factors involved in cell proliferation and differentiation, were also downregulated in *Lcn2*^–/–^ mice (Fig. 7F). qPCR results demonstrated a significant decrease in the expression of inflammatory factors (*Tnfrsf12a, Tnfsf12, Il6, Tnfa)*, keratinocyte proliferation marker genes *(Krt5* and *Krt17)*, and Lcn2 receptor genes *(24p3r* and *Mc4r)*, while the expression of differentiation genes (*Tgfb1* and *Krt10)* increased in the epidermis of *Lcn*2^–/–^ mice (Fig. 7G). Immunohistochemistry analysis further confirmed the decrease in proliferation marker (KRT5 and KRT14) expression in the epidermis of *Lcn2*^−/−^ mice (Fig. 7H). Therefore, *Lcn2* deletion attenuates IMQ-induced cell proliferation and inflammatory factor production in mouse skin lesions.

### TWEAK exacerbates skin lesions in the *Lcn2*^–/–^ murine psoriasis model

After learning that LCN2 can upregulate the TWEAK in keratinocytes, we then explored whether TWEAK exacerbates skin lesions without LCN2. We administrated *Lcn2*^–/–^ mice with 40 μg rmTWEAK per day for 6 consecutive days and collected the tissue protein for Western blotting analysis. The results revealed a significant upregulation of KRT5 (*P* = 0.0094) and KRT14 (*P* = 0.004) expression in the skin lesions of *Lcn2*^–/–^ mice when stimulated by rmTWEAK (Fig. S8A). Although the expression of Fn14 showed an increasing trend, the difference was not statistically significant (Fig. S8A). However, no significant differences were observed in the expression of KRT6, KRT16, KRT17, and CXCL10 (Fig. S8A). In addition, qPCR analysis of the *Lcn2*^–/–^ murine IMQ model revealed that additional rmTWEAK stimulation led to a significant decrease in the expression of *Tnfsf12, Tnfrsf12a, Il6*, and *24p3r* in the skin. However, there were no significant differences in the expression levels of *Il1b, Tnfa, Tgfb1, Krt5, Krt10, Krt17*, and *Mc4r* mRNA between these groups (Fig. S8B). IHC results further confirmed the upregulation of KRT5 (*P* = 0.0313) and KRT14 (*P* = 0.0063) expression in skin lesions following rmTWEAK stimulation (Fig. S8C). Based on these results, TWEAK was observed to counteract the protective impact of LCN2 knockdown on psoriatic lesions. This reaction subsequently promoted the expression of markers linked to a partial reduction in the differentiation of keratinocytes. Combined with the previous results, we posit that TWEAK secretion may be downstream of LCN2.

### LCN2 and TWEAK cooperate via Fn14 to promote psoriasis

**Fig. 8:**
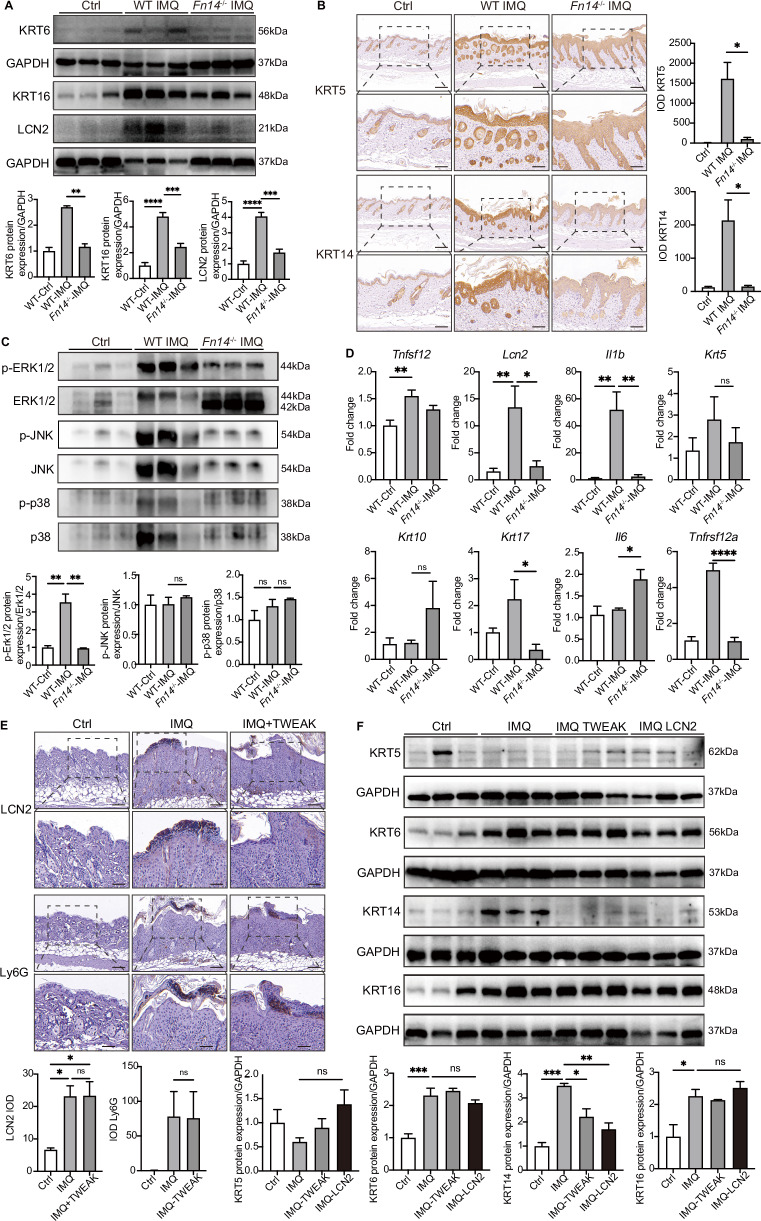
LCN2 acts on the downstream MAPK signaling pathway by binding to Fn14, and the deletion of *Fn14* can counteract the effect of TWEAK or LCN2. **A** KRT6, KRT16, LCN2 were detected by WB in the skin of wild-type mice as well as in the *Fn14*^–/–^ mouse imiquimod model. (*n* = 3 per group). **B** Immunohistochemistry was used to detect the expression of KRT5 and KRT14 in the skin of wild-type mice and *Fn14*^–/–^ mice (Brown). Nuclei were stained with hematoxylin (Bar = 200 μm). **C** WB detected p-ERK1/2, p-JNK and p-p38 expression in wild-type mice and *Fn14*^–/–^ mouse. (*n* = 3 per group). **D** *Tnfsf12*, *Tnfrsf12a*, *Lcn2*, *Il1b*, *Il6*, *Krt5*, *Krt16*, and *Krt17* mRNA expression levels were detected by using RT-qPCR methods. **E** Immunohistochemistry was used to detect the levels of LCN2 and Ly6G in the epidermis of *Fn14*^–/–^ mouse imiquimod model after administration of TWEAK. **F** After treated by LCN2 or TWEAK, Western blot was used to detect the expression of KRT5, KRT6, KRT14, and KRT16 in the *Fn14*^–/–^ mice skin tissue. (*n* = 3 per group) Data were pooled from three independent experiments. Data are shown as means ± SEM. *P < 0.05, **P < 0.01, and ***P < 0.001. SPR surface plasmon resonance

LCN2 promotes TWEAK in keratinocytes while TWEAK upregulated the LCN2 in neutrophils, we therefore checked whether both LCN2 and TWEAK can act synergistically on Fn14 to participate in the pathogenesis of psoriasis. To determine this, we used a mouse with global knockout of *Fn14* (*Fn14*^–/–^) and treated with rmLCN2 and rmTWEAK based on the IMQ-induced model. Skin proteins were extracted from the lesions and Western blotting was conducted to check the expression of KRT6, KRT16, and LCN2. The results revealed decreased expression under Fn14 knockout (Fig. 8A). Immunohistochemistry results of psoriatic lesions revealed significantly lower expression of KRT5 and KRT14 in the epidermis of *Fn14*^–/–^ mice after IMQ application (Fig. 8B). The expression levels of signal protein p-ERK1/2, p-JNK, and p-p38 were also detected by Western blotting, and the results demonstrated a significant decrease in p-ERK1/2 expression under Fn14 knockout (Fig. 8C). Furthermore, qPCR analysis showed a significant decrease in mRNA levels of *Tnfrsf12a, Lcn2, Il1b*, and *Krt17* in the epidermis of *Fn14*^–/–^ mice, while *Tnfsf12* and *Krt5* exhibited a decrease without significant differences. Conversely, *Il6* and *Krt10* mRNA levels were increased in *Fn14*^–/–^ mice (Fig. 8D).

In the *Fn14*^–/–^ IMQ-induced mouse models, the number of cells expressing LCN2 and Ly6G remained significantly increased compared to the normal control group. Surprisingly, additional stimulation with rmTWEAK did not lead to a significant increase in cell numbers (Fig. 8E). This suggests that even in the absence of Fn14, neutrophils can still infiltrate the psoriatic skin lesions. qPCR results revealed significantly increased expression of *Lcn2, Tnfsf12, Il1b, Krt5*, and *Krt10* mRNA in the skin of *Fn14*^–/–^ mice with IMQ-induced psoriasis (Fig. S9). Stimulation with rmLCN2 primarily led to increased expression of *Il1b* and *Il6*, while the levels of *Krt5, Krt10*, and *Krt17* mRNA were significantly decreased compared to the IMQ-induced model (Fig. S9). Our findings demonstrate that the number of LCN2 and Ly6G positive cells in the skin lesions of *Fn14*^–/–^ mice is significantly increased. Furthermore, Western blotting results indicated that stimulation with rmLCN2 does not significantly alter the expression levels of KRT5, KRT6, and KRT16 proteins in the skin of *Fn14*^−/−^ imiquimod-induced mice, while the expression of KRT14 protein displayed a decreased trend (Fig. 8F). Therefore, *Fn14* deficiency can negatively regulate the expression of the *Lcn2* gene, and additional stimulation with rmLCN2 cannot promote the expression of proteins related to epidermal proliferation.

To further validate that LCN2 promotes psoriasis development by interacting with Fn14, we stimulated Fn14-overexpressing keratinocytes with rhLCN2 and observed that these keratinocytes were more readily activated and exhibited higher expression levels of inflammatory factors. qPCR results revealed a significant upregulation in the gene levels of inflammatory cytokines such as *IL6*, *TNFa*, chemokines *CCL2*, *CCL5*, *CXCL5*, *CXCL10*, and the matrix metalloproteinase *MMP9*, while the expression of *MMP2* was significantly downregulated (Fig. S10A). Additionally, immunofluorescence staining demonstrated that the expression level of TWEAK in Fn14-overexpressing keratinocytes was higher after LCN2 stimulation (Fig. S10B). Therefore, we conclude that LCN2 participates in the inflammatory process in keratinocytes by binding to Fn14.

## Discussion

In this study, we initially discovered the increased expression of LCN2, TWEAK, and Fn14 in the lesions from patients with psoriasis, suggesting a potential correlation among the three. This observation was further supported in murine lesions where we found a significant decrease in the expression levels of TWEAK and Fn14 in mice lacking the *Lcn2* gene. Our investigation confirmed the involvement of the *Lcn2* gene in cytokine activation, as well as keratinocyte differentiation and proliferation. Specifically, we observed that LCN2 promotes the expression of TWEAK and the M1 marker *inos* by macrophages. Remarkably, global knockout of *Lcn2* in mice exhibited a protective impact on keratinocyte proliferation and differentiation in IMQ-induced psoriatic lesions. Notably, even in the absence of *Lcn2*, TWEAK continued to stimulate the expression of proteins associated with epidermal proliferation. We also discovered that TWEAK has the ability to induce the expression of LCN2 in neutrophils. Moreover, surface plasmon resonance analysis and Co-IP experiment confirmed the binding activity between LCN2 and Fn14, indicating that LCN2 may exert its biological functions through binding to Fn14. Fn14 deficiency was found to dampen the expression of LCN2 upstream, consequently mitigating the expression of proteins and genes linked to keratinocyte cell proliferation in psoriatic skin lesions. This study represents the first comprehensive investigation to elucidate the interplay among LCN2, TWEAK, and Fn14, underscoring the synergistic effect between LCN2 and TWEAK mediated via Fn14.

LCN2 is highly expressed in the lesions and serum of patients with psoriasis [37–39]. Recent studies have highlighted its involvement in promoting psoriasis development through various signaling pathways, such as the Th17, SREBP2-NLRC4, and TLR4/IL36R pathways [11, 40, 41]. As an inflammatory cytokine, LCN2’s pro-inflammatory effects have been extensively studied. Our observations reveal a positive correlation between LCN2 protein levels in lesional skin and the severity of erythema, indicating its involvement in the inflammatory response of psoriatic skin lesions. Similarly, Hau et al. found that LCN2 exacerbates erythema and scaling in psoriatic skin lesions [40], which aligns with our findings (Fig. S11). Furthermore, we demonstrated LCN2’s role in promoting the expression of TWEAK and Fn14 in keratinocytes and its synergistic effect with TWEAK in promoting the expression of inflammatory factors, cell proliferation, and differentiation. LCN2 and TWEAK can activate the MAPK signaling pathway under M5 cytokine conditions, suggesting their role in promoting keratinocyte proliferation in psoriatic skin lesions. In IMQ-induced psoriatic mouse models, *Lcn2* deficiency alleviated inflammatory tone and epidermal proliferation while reducing the protein levels of TWEAK and Fn14. Conversely, administering TWEAK worsened keratinocyte proliferation and differentiation. These results indicate that LCN2 acts as a stimulator of TWEAK and Fn14. SPR and Co-IP experiments confirmed the binding of LCN2 to Fn14. In *Fn14*^−/−^ IMQ-induced mice, the expression levels of LCN2, KRT5, KRT6, KRT14, and KRT16 proteins in the skin were decreased, while LCN2 administration did not significantly affect their expression, suggesting LCN2 primarily regulates keratinocyte proliferation through Fn14. These findings align with Gaudineau et al. ’s research, demonstrating that LCN2 not only participates in inflammatory reactions but also promotes TWEAK and Fn14 effects [25]. Additionally, we found that TWEAK upregulates the mRNA of LCN2 receptor *24p3r* and *Mc4r* in skin lesions of WT IMQ-induced mouse models. Although *Lcn2* deficiency inhibits its role in promoting proliferation-related proteins in keratinocytes, it still enhances *Il6, Il1b*, and *Lcn2* mRNA levels. Ma et al. propose that LCN2 may promote inflammation through the expression of IL-1β and other pro-inflammatory cytokines on the surface of keratinocytes via the 24P3R receptor [1]. We speculated that LCN2 promotes the expression of psoriasis-related inflammatory factors within keratinocytes through the 24P3R receptor, while facilitating keratinocyte proliferation and differentiation through the Fn14 receptor, but future research is needed to validate it.

Neutrophils play significant roles in the pathogenesis of psoriasis. Extensive research has demonstrated the chemotactic abilities of LCN2 and TWEAK, attracting neutrophils and macrophages to the site of inflammation [42, 43]. Shao et al. conducted an in vitro study revealing that LCN2 effectively attracts neutrophils to areas with high concentrations of LCN2 itself [44]. Conversely, TWEAK facilitates the expression of CCL2 within cells by interacting with Fn14, thereby luring neutrophils and macrophages to the site of inflammation [21, 22]. Our research investigated the IMQ mice model, indicating that TWEAK encourages the infiltration of neutrophils into the skin lesions while upregulating the expression of LCN2. In contrast, the chemotactic effect remains unimpaired in the psoriasis model of *Fn14*^–/–^ mice, suggesting direct participation of TWEAK in the process of neutrophil chemotaxis. That means TWEAK may directly or indirectly induce the infiltration of neutrophils to the site of inflammation by promoting the expression of LCN2 in neutrophils, forming a feed forward loop. Furthermore, our findings indicate that LCN2 upregulates the gene and protein levels of TWEAK and Fn14 in keratinocytes, thereby promoting the expression of inflammatory cytokines like IL-6 and IL-1β. Additionally, TWEAK enhances the expression and extracellular secretion of LCN2 protein in neutrophils. Our study has also revealed that LCN2 can promote the differentiation of macrophages towards the M1 phenotype, upregulate the expression of Fn14, and stimulate the expression and secretion of TWEAK by macrophages. The findings of Guo et al. and Nguyen et al. align with our conclusions, as they have also shown that LCN2 can induce macrophage polarization towards the M1 phenotype [12, 45]. Moreover, LCN2 is capable of enhancing the expression of its receptors such as 24P3R and MC4R in macrophages, suggesting that LCN2 may regulate macrophage differentiation and promote TWEAK secretion through those receptors.

Based on our findings and existing researches, we propose that the LCN2-TWEAK-Fn14 loop plays a critical role in the interactions among keratinocytes, neutrophils, and macrophages, with the following specific mechanisms: in psoriatic lesions, LCN2 secreted by infiltrating neutrophils and keratinocytes binds to the Fn14 receptor on the surface of keratinocytes. This binding promotes keratinocyte proliferation and the secretion of CCL2 and CXCL10, which recruit macrophages to the lesion site. Additionally, LCN2 induces the secretion of CXCL5 in keratinocytes, which further recruits neutrophils, while LCN2 itself also exhibits chemotactic activity for neutrophils. When macrophages migrate to the lesion site, LCN2 may binds to the 24P3R receptor on their surface, promoting their differentiation into the M1 phenotype and enhancing TWEAK expression. TWEAK, in turn, binds to the Fn14 receptor in keratinocytes and upregulates CCL2 expression, further recruiting macrophages and neutrophils to the site. Consequently, keratinocytes, neutrophils, and macrophages interact collaboratively via the LCN2-TWEAK-Fn14 signaling pathway, forming a complex network that collectively promotes the occurrence and progression of psoriasis.

We acknowledge that our study has certain limitations. Notably, we used Balb/c mice for *Fn14*^−/−^ strain and C57BL/6 mice for *Lcn2*^–/–^ strain, which may introduce variability due to the strain differences. Moving forward, conducting experiments with mice possessing dual deficiencies in *Lcn2* and *Fn14* within the same strain background could offer a more comprehensive understanding of the interactions among LCN2, TWEAK, and Fn14. While our study focused on only two genetically modified mouse strains, previous research by Sidler et al. has highlighted the protective impact of TWEAK deficiency in imiquimod-induced skin inflammation in C57BL/6 mice [18]. When combined with our research findings, this provides suggestive evidence that LCN2, TWEAK, and Fn14 interact synergistically and are highly relevant to the pathogenesis of psoriasis. Additionally, it is worth noting that LCN2 and TWEAK can directly regulate the levels of Fn14 in our study.

In summary, our study identifies LCN2 as a key regulator in the TWEAK/Fn14 signaling pathway, impacting psoriasis lesion development, inflammatory cell infiltration, and release of inflammatory factors. TWEAK operates as a positive feedback mechanism, heightening neutrophil infiltration and LCN2 expression, worsening skin lesions. Fn14, elevated by LCN2 stimulation, boosts keratinocyte proliferation. Consequently, a proposed synergistic biologic therapy targeting LCN2 and TWEAK emerges as a novel, effective approach for psoriasis treatment. This concept presents compelling prospects for future research in the field.

## Supplementary information

Supplementary figures

Supplementary tables

Supplementary data

Unprocessed images

## Data Availability

The bulk-RNA sequencing data were deposited under GSE242420. All the other data associated with this study are present in the paper.
