# Supplementary material for: Synergistic effects of LCN2 and TWEAK on the progression of psoriasis
Source: Cell Mol Immunol. 2025 May 15;22(7):760–75. doi: 10.1038/s41423-025-01292-9 (PMC12206918; doi:10.1038/s41423-025-01292-9)
Supplement: Supplementary file 1 — Supplementary figures [file 41423_2025_1292_MOESM1_ESM.pdf]

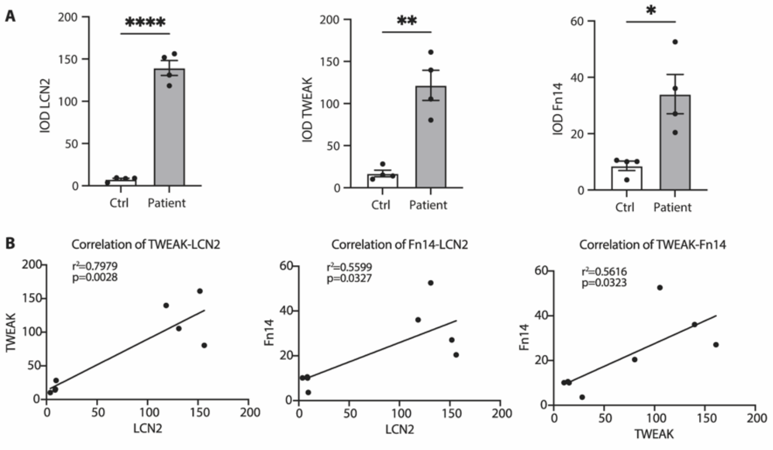

Fig. S1 High expression of LCN2, TWEAK and Fn14 in psoriatic lesions and their positive correlations. (A) IHC analysis showing the expression levels of LCN2, TWEAK, and Fn14 in psoriatic lesions (n = 3). (B) Correlation analysis among LCN2, TWEAK, and Fn14. Data are presented as mean ± SEM. *P < 0.05, **P < 0.01, and ***P < 0.001.

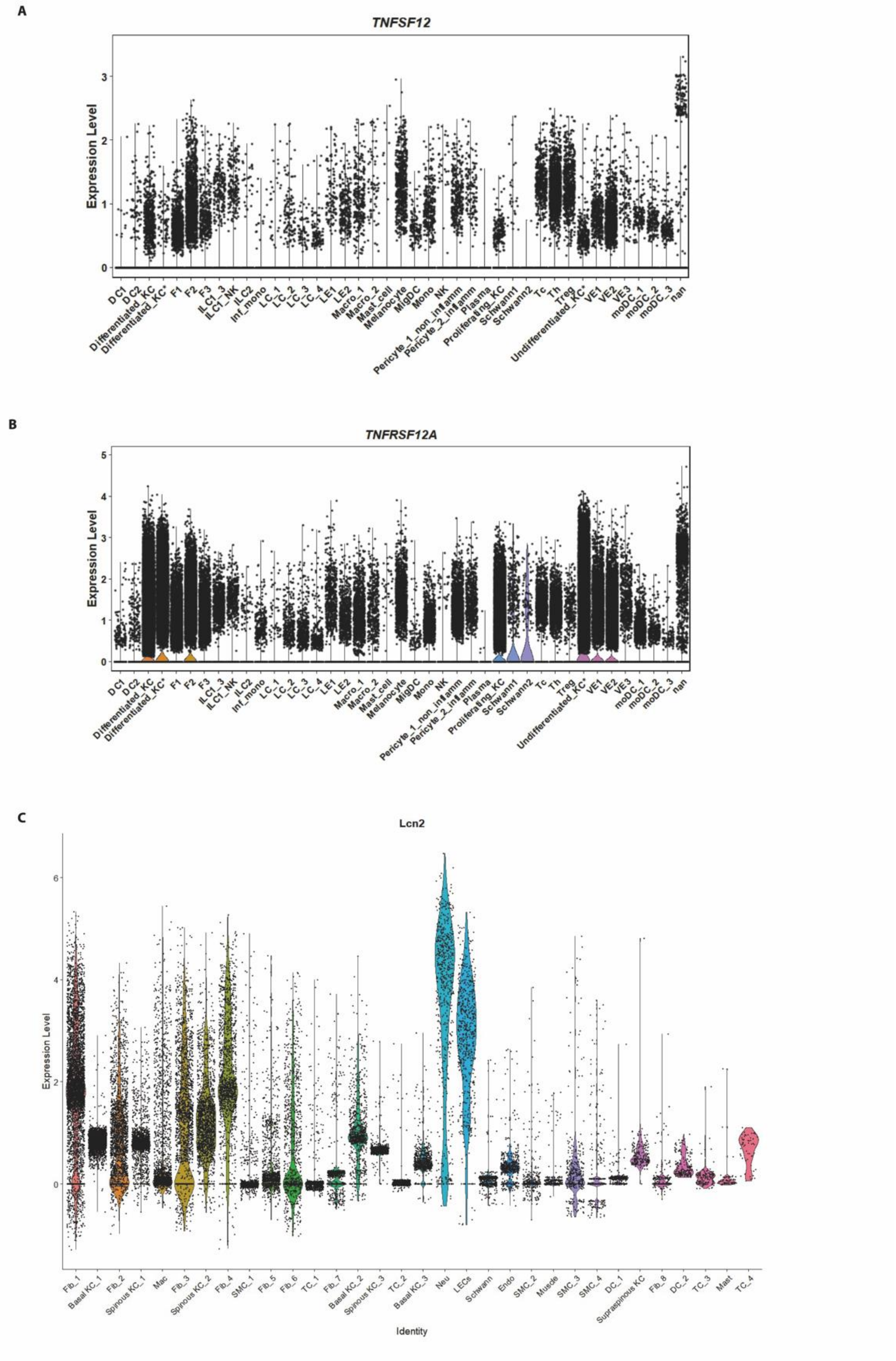

Fig. S2 Single-cell RNA sequencing (scRNA-seq) analysis of cell types expressing LCN2, TWEAK, and Fn14. Cell types expressing LCN2, TWEAK, and Fn14 were analyzed using two scRNA-seq datasets of psoriasis from Cheng JB et al. and Reynolds et al.

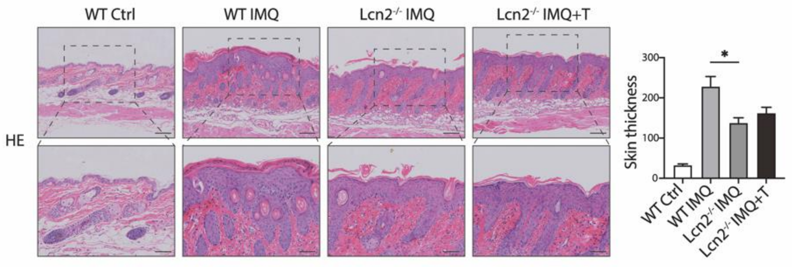

Fig. S3 HE staining in *Lcn2*$^{-/-}$ mice showed significant reduction in epidermal thickness. HE staining were performed on paraffin section and skin thickness were measured in all groups (n = 3-5). Bar=100μm. Data are presented as mean ± SEM. *P < 0.05, **P < 0.01, and ***P < 0.001

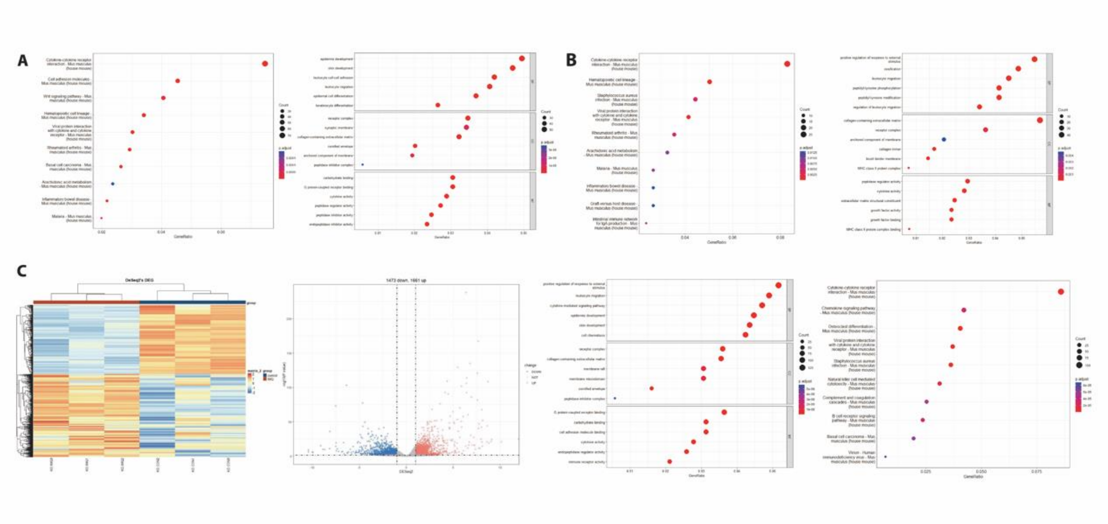

Fig. S4 KEGG and GO analysis of differences between wild-type and *Lcn2*$^{-/-}$ mice psoriatic models. (A) GO and KEGG analysis of wild-type control mice and wild-type imiquimod model mice. (B) GO and KEGG analysis of wild-type and *Lcn2*$^{-/-}$imiquimod mice model. (C) RNA-seq analysis of *Lcn2*$^{-/-}$control mice and *Lcn2*$^{-/-}$imiquimod mice model.

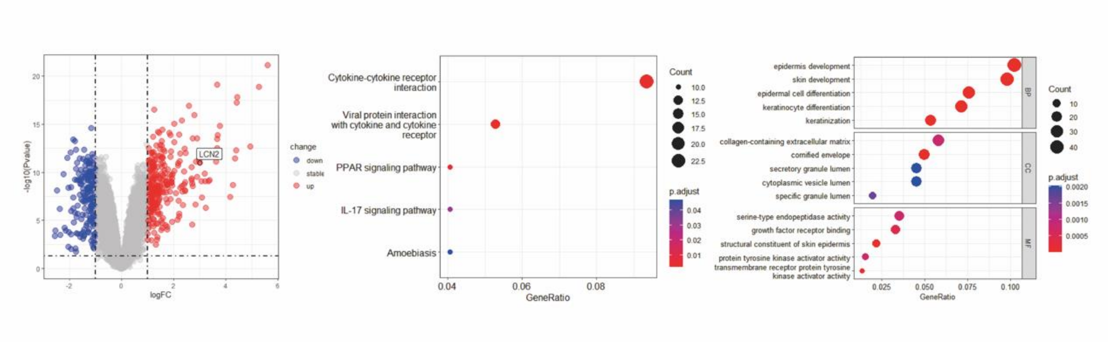

Fig. S5 Elevation of LCN2 expression in psoriatic lesions correlates with degree of erythema. RNA-seq analysis of normal human skin tissues and skin lesions from psoriasis patients (GSE161683). GO and KEGG analysis of health control and psoriasis patients.

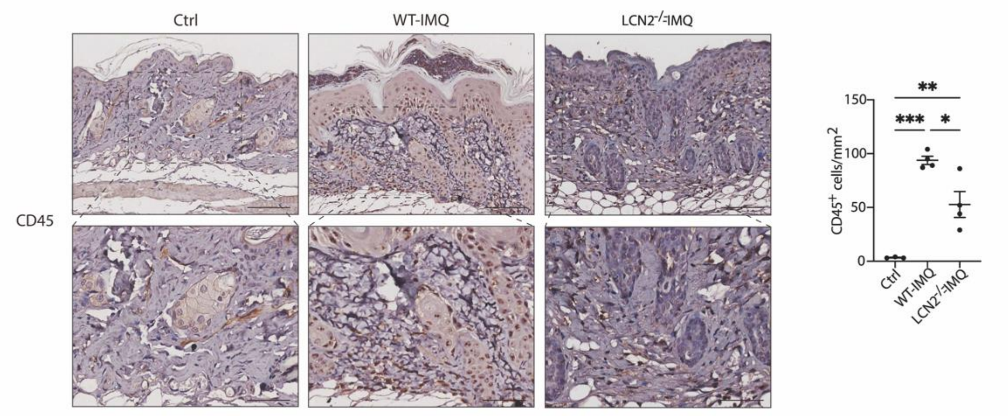

Fig. S6 Reduced number of $CD45^+$ leukocytes in skin lesions of *Lcn2*$^{-/-}$ mice. IHC staining showing the number of $CD45^+$ cells in skin lesions of mice (n = 3-4). Scale bar = 100 μm. Data are presented as mean ± SEM. *P < 0.05, **P < 0.01, and ***P < 0.001.

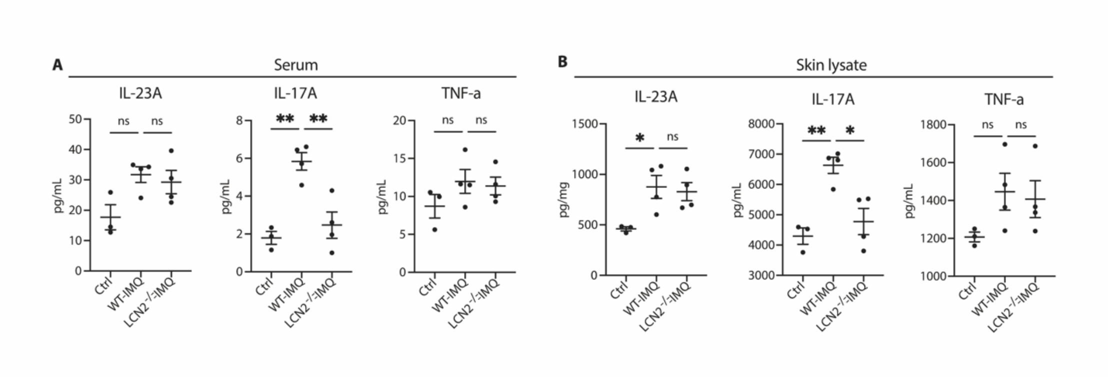

Fig. S7 Expression of IL-23A, IL-17A and TNF-α in serum and skin lesions of *Lcn2* knockout mice. (A, B) Concentrations of IL-23A, IL-17A, and TNF-α in mouse serum and skin lesion lysates were measured by ELISA (n = 3-4). Data are presented as mean ± SEM. *P < 0.05, **P < 0.01, and ***P < 0.001

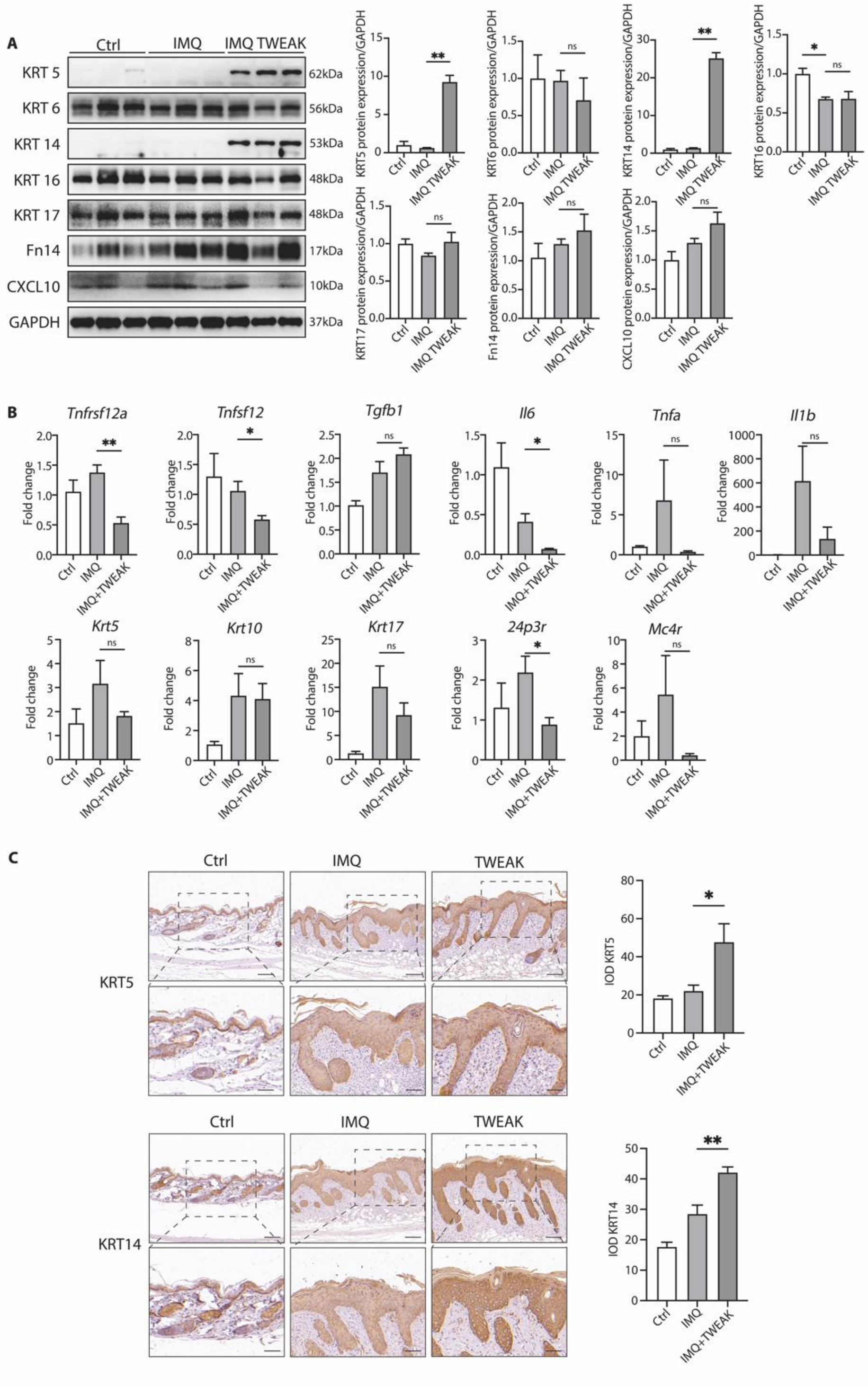

Figure S8. TWEAK is a downstream protein of LCN2 and promotes the expression of keratinocyte proliferation-related proteins.

(**A**) The expression levels of Keratin 5, Keratin6, Keratin14, Keratin17, Fn14, and CXCL10 proteins in the skin tissues of the control, IMQ-treated, and IMQ with TWEAK-treated groups in the

background of *Lcn2*$^{-/-}$ mice were detected by western blot (n = 3 per group). (**B**) Relative mRNA level of *Tnfrsf12a*, *Tnfsf12*, *Tgfβ1*, *Il6*, *Tnfα*, *Il1β*, *Krt5*, *Krt10*, *Krt17*, *24p3r*, *Mc4r* was detected by RT-qPCR. (**C**) Detection of KRT5 and KRT14 in mouse skin using immunohistochemical methods (Brown). Nuclei were stained with hematoxylin (Bar = 200μm). ANOVA was used for comparison between groups: **P* < 0.05, ***P* < 0.01, and ****P* < 0.001. ns, not significant.

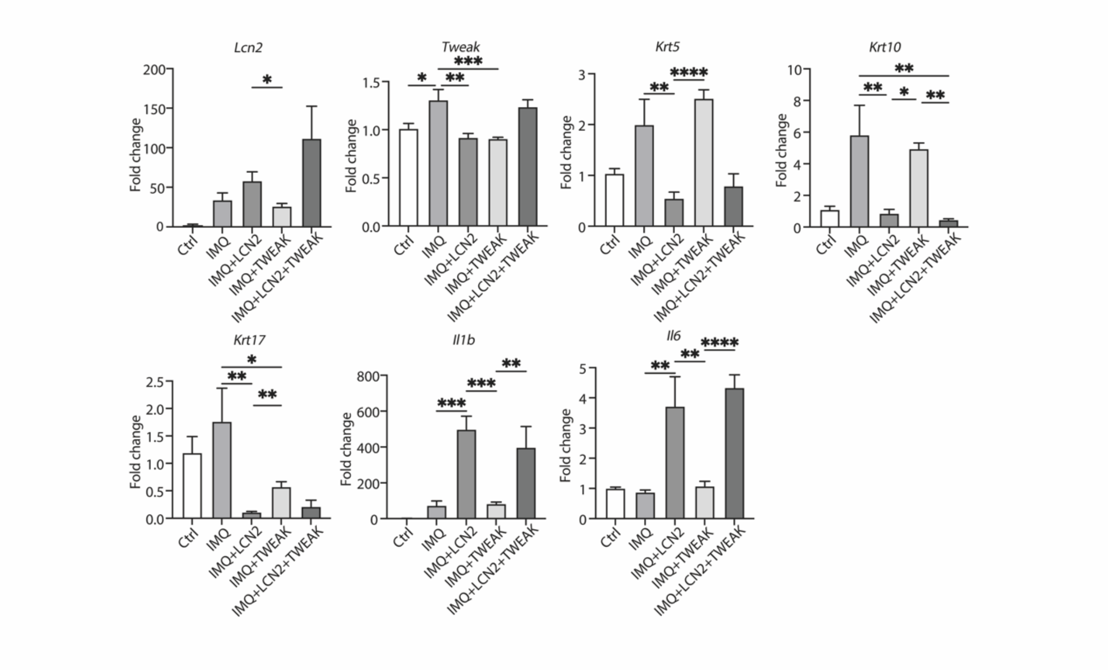

Fig. S9 Differential gene expression among groups of *Fn14*$^{-/-}$ mice treated with LCN2 or TWEAK stimulation in the IMQ model. Relative mRNA expression of *Tweak*, *Il6*, *Il1b*, *Krt5*, *Krt10* and *Krt17* in *Fn14*$^{-/-}$mice groups treated with IMQ, LCN2, TWEAK, combined or respectively, were detected by RT-qPCR. Data are presented as mean ± SEM. *P < 0.05, **P < 0.01, and ***P < 0.001

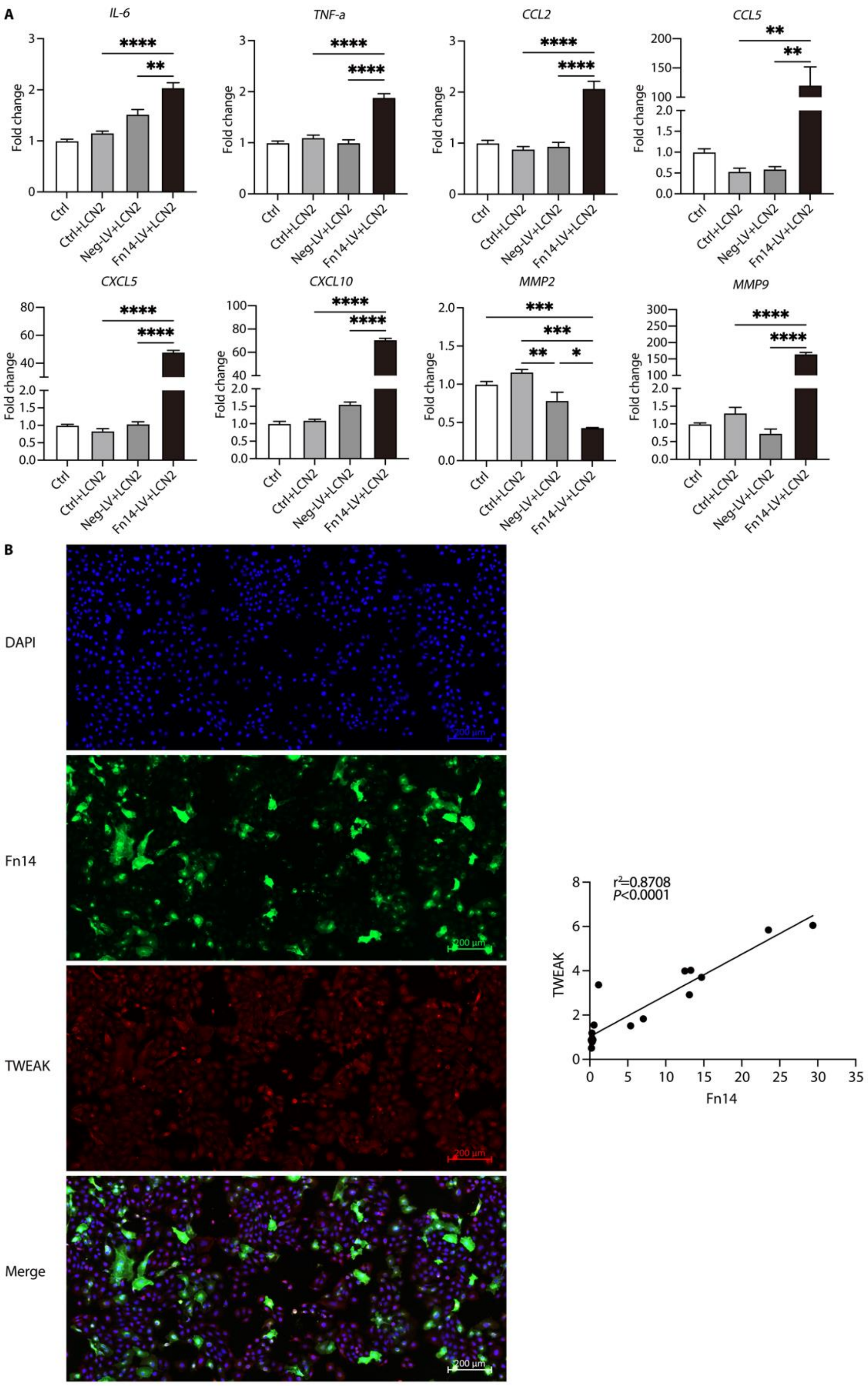

Figure S10. Overexpression of Fn14 enhances LCN2-induced inflammatory effects in keratinocytes and increases TWEAK expression

(**A**) Relative mRNA level of *IL-6*, *TNFα*, *CCL2*, *CCL5*, *CXCL5*, *CXCL10*, *MMP2*, *MMP9* was detected by RT-qPCR. (**B**) The expression level of TWEAK in cells with

Fn14 overexpression was assessed by cell immunofluorescence following LCN2 stimulation. Data were pooled from three independent experiments. Data are shown as means ± SEM. *P < 0.05, **P < 0.01, and ***P < 0.001.

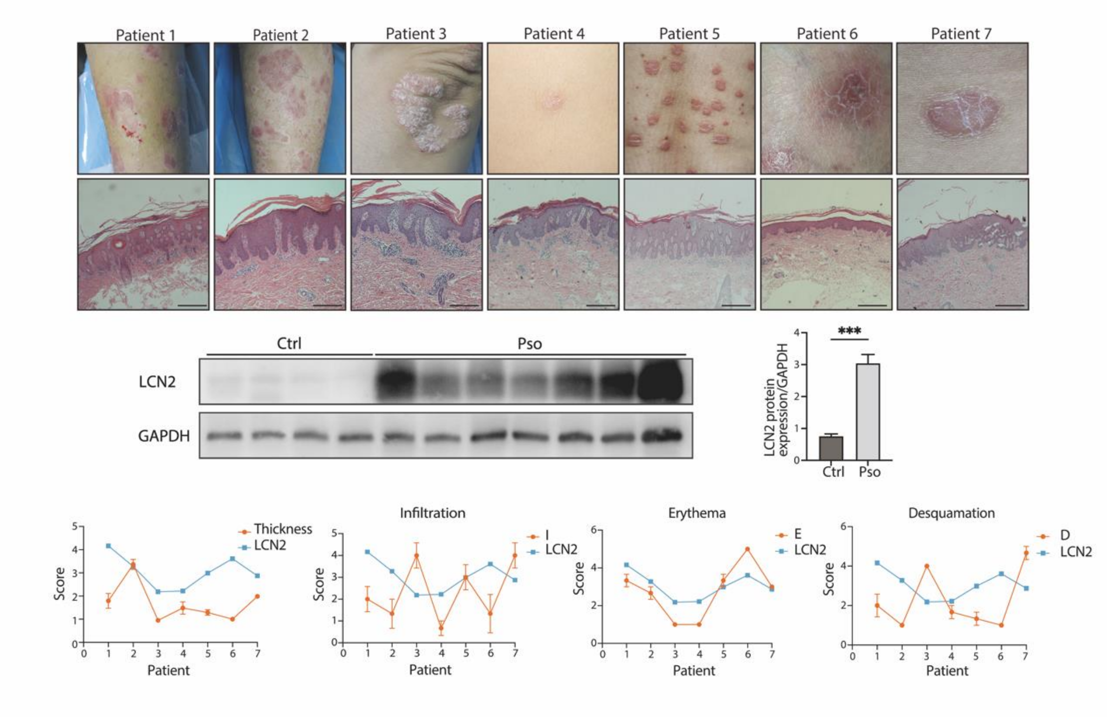

Fig. S11 Elevation of LCN2 expression in psoriatic lesions correlates with degree of erythema. Photographs of patients' skin lesions as well as HE-stained sections were analyzed for correlation of LCN2 protein levels with skin lesion scores and epidermal thickness. Detection of LCN2 protein content in skin lesions using Western blot assay. Data were obtained from three independent experiments (n=3). Data are presented as mean ± SEM. *P < 0.05, **P < 0.01, and ***P < 0.001.
