# Supplementary material for: Synergistic effects of LCN2 and TWEAK on the progression of psoriasis
Source: Cell Mol Immunol. 2025 May 15;22(7):760–75. doi: 10.1038/s41423-025-01292-9 (PMC12206918; doi:10.1038/s41423-025-01292-9)
Supplement: Supplementary file 2 — Supplementary tables [file 41423_2025_1292_MOESM2_ESM.docx]

**Table S1. Differential protein expression revealed by proteomic analysis**

| Accession | Protein description | Gene name | Regulated type |
| --- | --- | --- | --- |
| O14896 | Interferon regulatory factor 6 | IRF6 | Up |
| O15360 | Fanconi anemia group A protein | FANCA | Down |
| O15392 | Baculoviral IAP repeat-containing protein 5 | BIRC5 | Down |
| O43312 | Protein MTSS 1 | MTSS1 | Up |
| O43570 | Carbonic anhydrase 12 | CA12 | Down |
| O43909 | Exostosin-like 3 | EXTL3 | Down |
| O60701 | UDP-glucose 6-dehydrogenase | UGDH | Up |
| O60711 | Leupaxin | LPXN | Up |
| O75340 | Programmed cell death protein 6 | PDCD6 | Up |
| O75815 | Breast cancer anti-estrogen resistance protein 3 | BCAR3 | Down |
| O94817 | Ubiquitin-like protein ATG12 | ATG12 | Up |
| O95210 | Starch-binding domain-containing protein 1 | STBD1 | Up |
| O95235 | Kinesin-like protein KIF20A | KIF20A | Down |
| O95352 | Ubiquitin-like modifier-activating enzyme ATG7 | ATG7 | Up |
| O95361 | Tripartite motif-containing protein 16 | TRIM16 | Up |
| O95810 | Caveolae-associated protein 2 OS=Homo sapiens | CAVIN2 | Down |
| P01583 | Interleukin-1 alpha | IL1A | Up |
| P05120 | Plasminogen activator inhibitor 2 | SERPINB2 | Up |
| P05412 | Transcription factor Jun | JUN | Down |
| P07355 | Annexin A2 | ANXA2 | Up |
| P08243 | Asparagine synthetase [glutamine-hydrolyzing] | ASNS | Up |
| P08476 | Inhibin beta A chain | INHBA | Up |
| P14780 | Matrix metalloproteinase-9 | MMP9 | Up |
| P18510 | Interleukin-1 receptor antagonist protein | IL1RN | Up |
| P19971 | Thymidine phosphorylase | TYMP | Up |
| P21128 | Uridylate-specific endoribonuclease | ENDOU | Down |
| P24468 | COUP transcription factor 2 | NR2F2 | Down |
| P25116 | Proteinase-activated receptor 1 | F2R | Down |
| P30711 | Glutathione S-transferase theta-1 | GSTT1 | Up |
| P32004 | Neural cell adhesion molecule L1 | L1CAM | Up |
| P32321 | Deoxycytidylate deaminase | DCTD | Up |
| P37059 | 17-beta-hydroxysteroid dehydrogenase type 2 | HSD17B2 | Up |
| P40189 | Interleukin-6 receptor subunit beta | IL6ST | Up |
| P46531 | Neurogenic locus notch homolog protein 1 | NOTCH1 | Down |
| P46934 | E3 ubiquitin-protein ligase NEDD4 | NEDD4 | Up |
| P47895 | Aldehyde dehydrogenase family 1 member A3 | ALDH1A3 | Up |
| P49593 | Protein phosphatase 1F | PPM1F | Up |
| P50895 | Basal cell adhesion molecule | BCAM | Down |
| P51580 | Thiopurine S-methyltransferase | TPMT | Up |
| P55268 | Laminin subunit beta-2 | LAMB2 | Up |
| P57764 | Gasdermin-D | GSDMD | Up |
| P58107 | Epiplakin | EPPK1 | Up |
| P58317 | Zinc finger protein 121 | ZNF121 | Down |
| P60903 | Protein S100-A10 | S100A10 | Up |
| P61960 | Ubiquitin-fold modifier 1 | UFM1 | Up |
| P78325 | Disintegrin and metalloproteinase domain-containing protein 8 | ADAM8 | Up |
| Q00534 | Cyclin-dependent kinase 6 | CDK6 | Up |
| Q03426 | Mevalonate kinase | MVK | Up |
| Q04323 | UBX domain-containing protein 1 | UBXN1 | Up |
| Q12857 | Nuclear factor 1 A-type | NFIA | Down |
| Q12894 | Interferon-related developmental regulator 2 | IFRD2 | Up |
| Q12933 | TNF receptor-associated factor 2 | TRAF2 | Down |
| Q13501 | Sequestosome-1 | SQSTM1 | Up |
| Q14914 | Prostaglandin reductase 1 | PTGR1 | Up |
| Q15011 | Homocysteine-responsive endoplasmic reticulum-resident ubiquitin-like domain member 1 protein | HERPUD1 | Up |
| Q15404 | Ras suppressor protein 1 | RSU1 | Up |
| Q15628 | Tumor necrosis factor receptor type 1-associated DEATH domain protein | TRADD | Up |
| Q16719 | Kynureninase | KYNU | Up |
| Q24JP5 | Transmembrane protein 132A | TMEM132A | Up |
| Q2T9J0 | Peroxisomal leader peptide-processing protease | TYSND1 | Down |
| Q5T4B2 | Inactive glycosyltransferase 25 family member 3 | CERCAM | Down |
| Q5TZA2 | Rootletin | CROCC | Down |
| Q676U5 | Autophagy-related protein 16-1 | ATG16L1 | Up |
| Q69YH5 | Cell division cycle-associated protein 2 | CDCA2 | Down |
| Q6MZM0 | Ferroxidase HEPHL1 | HEPHL1 | Up |
| Q6N022 | Teneurin-4 | TENM4 | Down |
| Q6ZUT6 | Coiled-coil domain-containing protein 9B | CCDC9B | Up |
| Q6ZV73 | FYVE, RhoGEF and PH domain-containing protein 6 | FGD6 | Up |
| Q8N3Y3 | Xylosyl- and glucuronyltransferase LARGE2 | LARGE2 | Down |
| Q8N3Y7 | Epidermal retinol dehydrogenase 2 | SDR16C5 | Up |
| Q8N543 | Prolyl 3-hydroxylase OGFOD1 | OGFOD1 | Up |
| Q8N884 | Cyclic GMP-AMP synthase | CGAS | Up |
| Q8N8Z6 | Discoidin, CUB and LCCL domain-containing protein 1 GN=DCBLD1 PE=1 SV=2 | DCBLD1 | Down |
| Q8WWM9 | Cytoglobin | CYGB | Up |
| Q92870 | Amyloid beta precursor protein binding family B member 2 | APBB2 | Down |
| Q92990 | Glomulin | GLMN | Up |
| Q96AH8 | Ras-related protein Rab-7b | RAB7B | Down |
| Q96C90 | Protein phosphatase 1 regulatory subunit 14B | PPP1R14B | Up |
| Q96DU7 | Inositol-trisphosphate 3-kinase C | ITPKC | Up |
| Q96E29 | Transcription termination factor 3, mitochondrial | MTERF3 | Down |
| Q96EQ0 | Small glutamine-rich tetratricopeptide repeat-containing protein beta | SGTB | Up |
| Q96FN4 | Copine-2 | CPNE2 | Up |
| Q9BRK4 | Leucine zipper putative tumor suppressor 2 | LZTS2 | Down |
| Q9BXS6 | Nucleolar and spindle-associated protein 1 | NUSAP1 | Down |
| Q9BYX4 | Interferon-induced helicase C domain-containing protein 1 | IFIH1 | Up |
| Q9GZT4 | Serine racemase | SRR | Up |
| Q9GZX9 | Twisted gastrulation protein homolog 1 | TWSG1 | Down |
| Q9H4H8 | Protein FAM83D | FAM83D | Down |
| Q9H788 | SH2 domain-containing protein 4A | SH2D4A | Up |
| Q9H910 | Jupiter microtubule associated homolog 2 | JPT2 | Up |
| Q9NV35 | Nucleotide triphosphate diphosphatase NUDT15 | NUDT15 | Up |
| Q9UBP4 | Dickkopf-related protein 3 | DKK3 | Down |
| Q9UGK3 | Signal-transducing adaptor protein 2 | STAP2 | Up |
| Q9UH17 | DNA dC->dU-editing enzyme APOBEC-3B | APOBEC3B | Up |
| Q9UI42 | Carboxypeptidase A4 | CPA4 | Down |
| Q9UKY7 | Protein CDV3 homolog | CDV3 | Up |
| Q9Y376 | Calcium-binding protein 39 | CAB39 | Up |
| Q9Y617 | Phosphoserine aminotransferase | PSAT1 | Up |
| Q9Y6I4 | Ubiquitin carboxyl-terminal hydrolase 3 | USP3 | Down |

**Table S2. Clinical patient information and scores**

| Patient no. | Sex | Anatomic site | Erythema | Induration | Desquamation | Barker score | Patient PASI | Epidermal thickness |
| --- | --- | --- | --- | --- | --- | --- | --- | --- |
| Pso1 | M | Lower leg | 3 | 2 | 3 | 3 | 11 | 390.54 |
| Pso 2 | M | Lower leg | 3 | 1 | 2 | 2 | 13 | 170.52 |
| Pso 3 | F | Elbow | 1 | 4 | 3 | 3 | 6 | 111.46 |
| Pso 4 | F | Back | 1 | 1 | 1 | 1 | 2.9 | 148.90 |
| Pso 5 | F | Back | 3 | 3 | 3 | 3 | 2 | 205.84 |
| Pso 6 | F | Lower leg | 5 | 1 | 3 | 3 | 6.5 | 116.35 |
| Pso 7 | M | Elbow | 3 | 4 | 4 | 4 | 3 | 230.42 |

**Table S3. The primary antibodies used in the study**

| Antibodies | Dilution | Company | Cat# |
| --- | --- | --- | --- |
| Anti-GAPDH | 1:1000 | CST | 2118 |
| Anti-Fn14 | 1:1000 | CST | 4403 |
| Anti-Fn14 | 1:200 | Santa Cruz | 56250 |
| Anti-TNF-a | 1:1000 | CST | 3707S |
| Anti-TWEAK | 1:1000 | Abcam | 37170 |
| Anti-TWEAK | 1:1000 | NBP1 | 76695 |
| Anti-p-ERK1/2 | 1:1000 | CST | 4370s |
| Anti-ERK1/2 | 1:1000 | CST | 4695 |
| Anti-LCN2 | 1:1000 | Abcam | 125075 |
| Anti-LCN2 | 1:1000 | Abcam | 216462 |
| Anti-p-p38 | 1:1000 | Abcam | 178867 |
| Anti-p38 | 1:1000 | Abcam | 170099 |
| Anti-Ip10 | 1:1000 | GeneTex | gtx31179 |
| Anti-Involucrin | 1:150 | Abcam | 53112 |
| Anti-Loricrin | 1:200 | Abcam | 85679 |
| Anti-KRT1 | 1:20000 | Abcam | 185628 |
| Anti-KRT5 | 1:10000 | Abcam | 52635 |
| Anti-KRT6 | 1:200 | Santa Cruz | 53260 |
| Anti-KRT10 | 1:10000 | Abcam | 76318 |
| Anti-KRT14 | 1:20000 | Abcam | 181595 |
| Anti-KRT16 | 1:125 | Santa Cruz | 53255 |
| Anti-KRT17 | 1:1000 | Abcam | 109725 |
| Anti-p-JNK | 1:1000 | Abmart | T40074 |
| Anti-JNK | 1:1000 | Abmart | T40073 |
| Anti-Ly6G | 1:200 | Servicebio | GB11229-100 |
| Anti-CD68 | 1:100 | Abcam | 125047 |
| Anti-CD66b | 1:2000 | Abcam | 300122 |
| Anti-F4/80 | 1:2000 | Servicebio | GB113373-100 |
| Anti-p-TRAF2 | 1:1000 | Abcam | E2B6L |
| Anti-TRAF2 | 1:1000 | CST | 4712 |

**Table S4. The primer sets used for mRNA detection**

| Genes | Forward (5'-- 3') | Reverse (5'-- 3') |
| --- | --- | --- |
| Human GAPDH | GGTGTGAACCATGAGAAGTATGA | GAGTCCTTCCACGATACCAAAG |
| Human TWEAK | GTACTCTGTGGGCAAGGATG | GGAGGAGCTTGTCTTGTTTCT |
| Human Fn14 | CCTTCCAAGGTGTCTGGTT | CAAATGCTGCAGTTCCTTAGTC |
| Human Lcn2 | CCCAGCCCCACCTCTGA | CTTCCCCTGGAATTGGTTGTC |
| Human 24p3R | TCCTGGGCTTCACCAACTTC | AATGCCGGTAAGGGTCATGG |
| Human Krt14 | CAGGAGATGATTGGCAGCGT | CAACACTGAGCTGGAGGTGA |
| Human Krt17 | CTCAAACTTGGTGCGGAAGT | GGCCGTCCAAATAGATCCCC |
| Human loricrin | GGCCGTCCAAATAGATCCCC | TGCAAACCTCGGGTAGCATC |
| Human involucrin | CAACTGAAGCATCTGGAGCA | AGGTGCTTTGGCTGTCCTAC |
| Mouse Il-1β | GCAACTGTTCCTGAACTCAACT | ATCTTTTGGGGTCCGTCAACT |
| Mouse iNOS | CCAAGCCCTCACCTACTTCC | CTCTGAGGGCTGACACAAGG |
| Mouse Il-10 | TTCTTTCAAACAAAGGACCAGC | GCAACCCAAGTAACCCTTAAAG |
| Mouse Il-6 | TAGTCCTTCCTACCCCAATTTCC | TTGGTCCTTAGCCACTCCTTC |
| Mouse Ip-10 | ATTTCCACGATTTCCCAGAG | AGGAGCCCTTTTAGACCTTTTT |
| Mouse Lcn2 | GGCCCTGAGTGTCATGTGTC | TTCTGATCCAGTAGCGACAGC |
| Mouse 24p3r | TTTGGCCGTCGTGGGATTG | GGCGCATCAGGTAGACACC |
| Mouse Mc4r | CCCGGACGGAGGATGCTAT | TCGCCACGATCACTAGAATGT |
| Mouse Tnfrsf12a | CTAGTTTCCTGGTCTGGAGAAGATG | CCCTCTCCACCAGTCTCCTCTA |
| Mouse Tnfsf12 | CGAGCTATTGCAGCCCATTAT | ACCTGCTTGTGCTCCATCCT |
| Mouse Tnf-a | TGAGCACAGAAAGCATGATCC | GCCATTTGGGAACTTCTCATC |
| Mouse Krt5 | CAGAGCTGAGGAACATGCAG | CATTCTCAGCCGTGGTACG |
| Mouse Krt10 | CAGCTGGCCCTGAAACAATC | AGTTGTTGGTACTCGGCGTT |
| Mouse Krt17 | TACCTGGACAAGGTGCGTG | GCTCTGTCTCAAACTTGGTACG |
| Mouse Tgfb1 | TGCGCTTGCAGAGATTAAAA | GCTGAATCGAAAGCCCTGTA |

**Table S5. Scoring criteria for mouse psoriasis model**

| Erythema | Scaling | Lesion thickness |
| --- | --- | --- |
| 0: No erythema | 0: No visible scaling | 0: Lesion is level with the normal skin |
| 1: Light red erythema | 1: Fine scaling, partially covering the lesion surface | 1: Lesion is slightly raised compared to normal skin |
| 2: Red erythema | 2: Moderate scaling, covering most of the lesion surface completely or incompletely | 2: Lesion is moderately raised, with rounded or sloped edges |
| 3: Dark red erythema | 3: Severe scaling, covering almost the entire lesion surface | 3: Lesion is thickened and significantly raised |
| 4: Very dark red erythema | 4: Very thick, layered scaling, covering the entire lesion surface | 4: Lesion is highly thickened and prominently raised |
