# Supplementary material for: Synergistic effects of LCN2 and TWEAK on the progression of psoriasis
Source: Cell Mol Immunol. 2025 May 15;22(7):760–75. doi: 10.1038/s41423-025-01292-9 (PMC12206918; doi:10.1038/s41423-025-01292-9)

Source Fig.2 LCN2

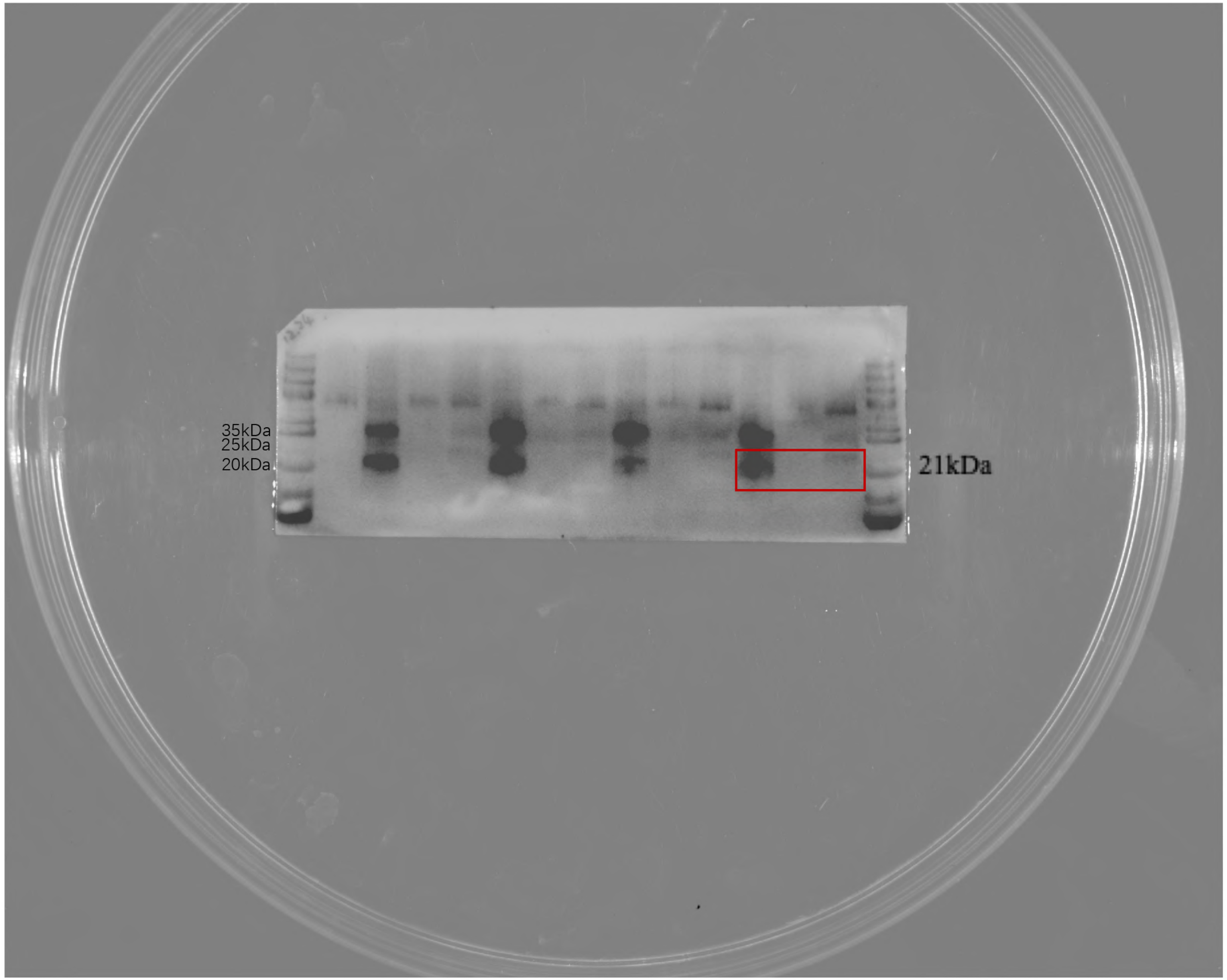

Source Fig.2 Fn14

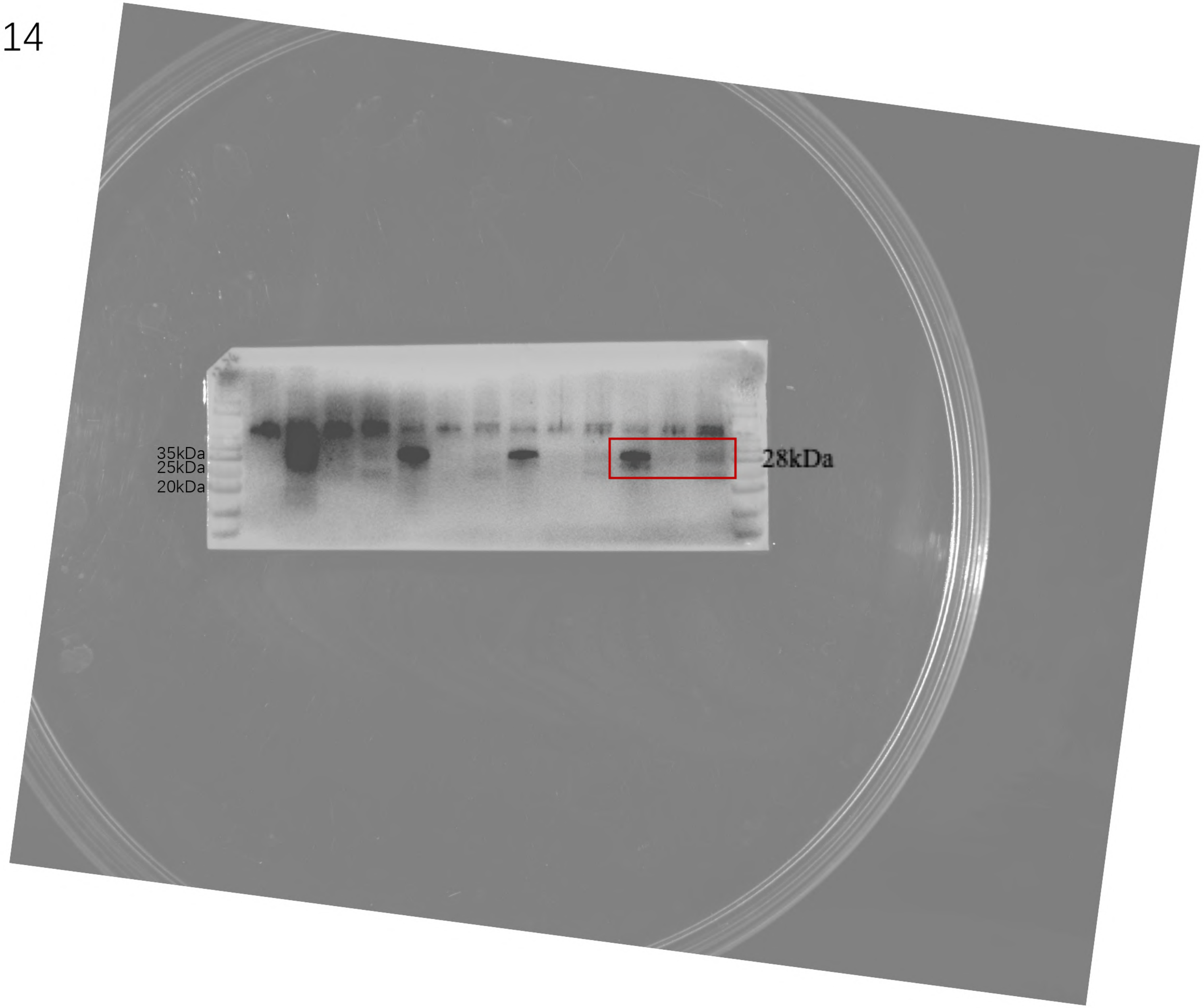

Source Fig.2 GAPDH

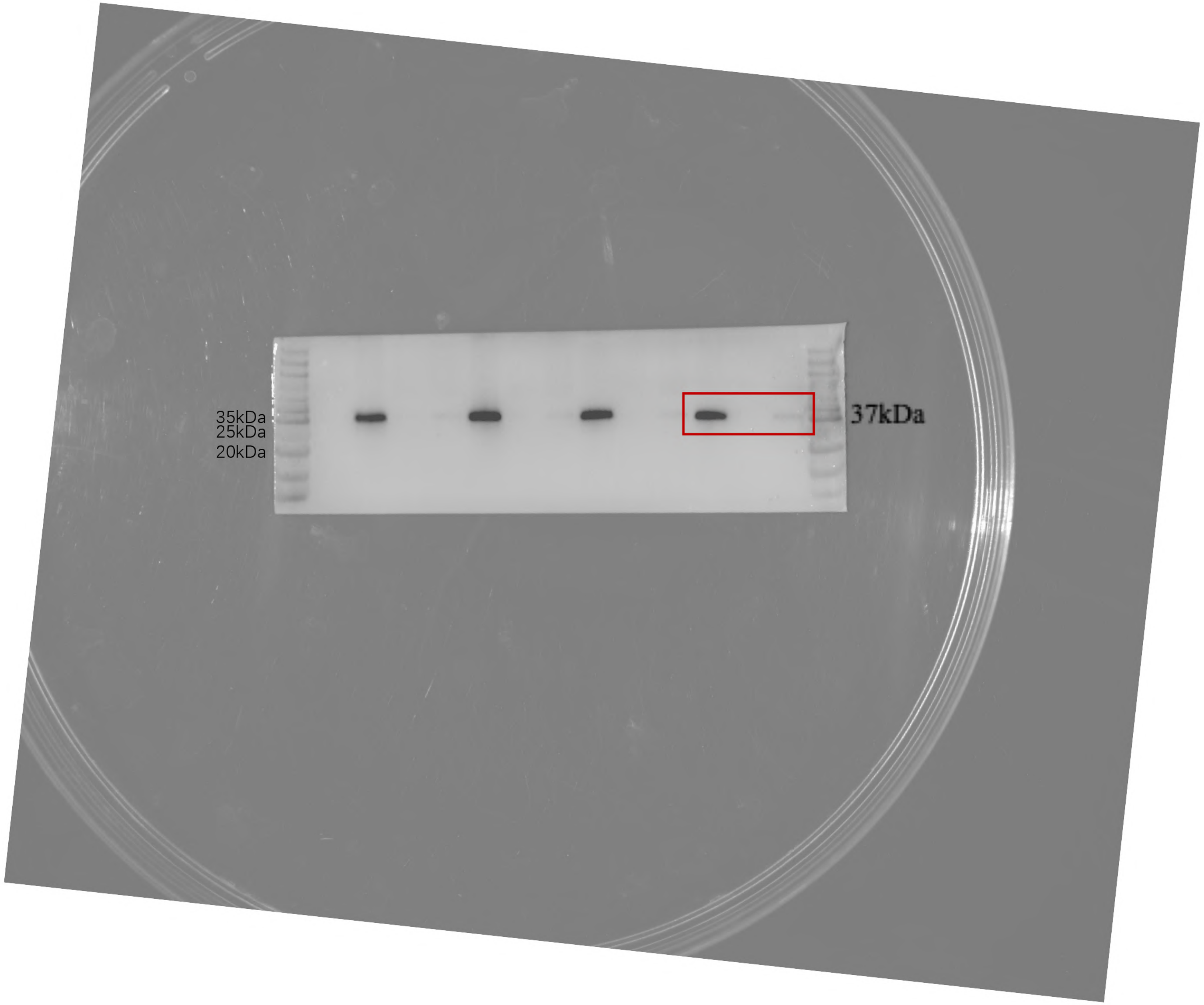

Source Fig.3C Fn14

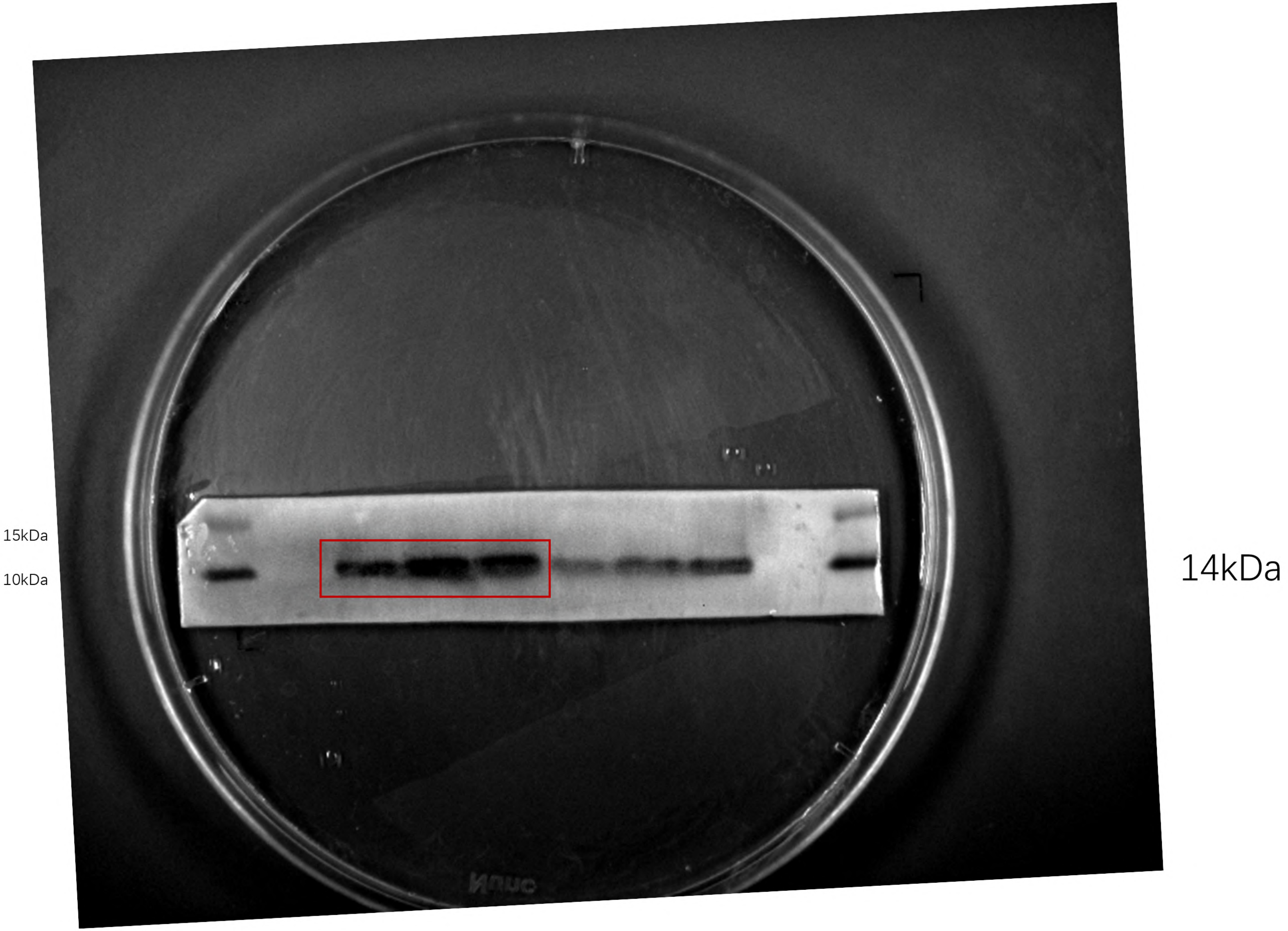

Source Fig.3C GAPDH

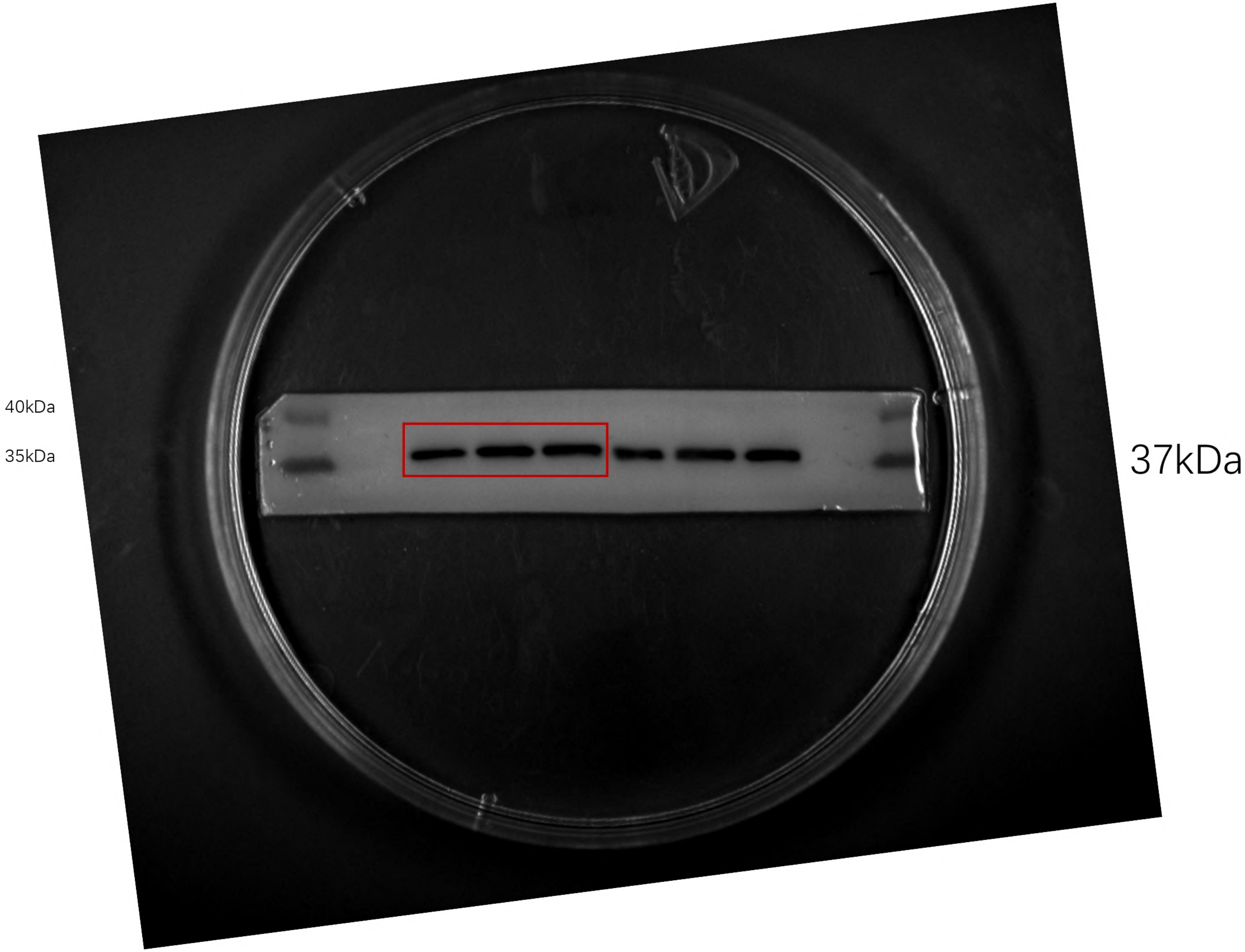

Source Fig.3C TWEAK

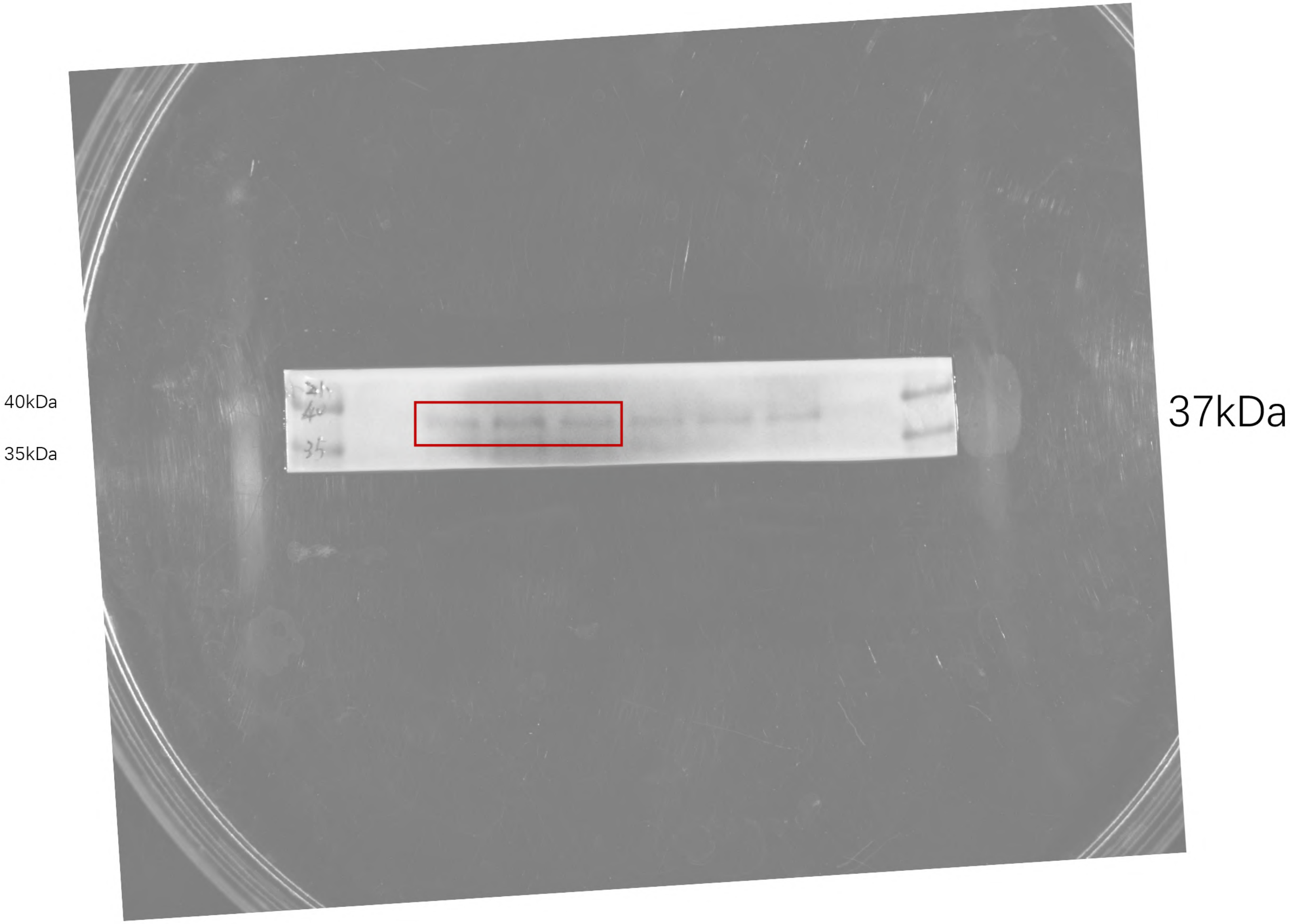

Source Fig.3C GAPDH

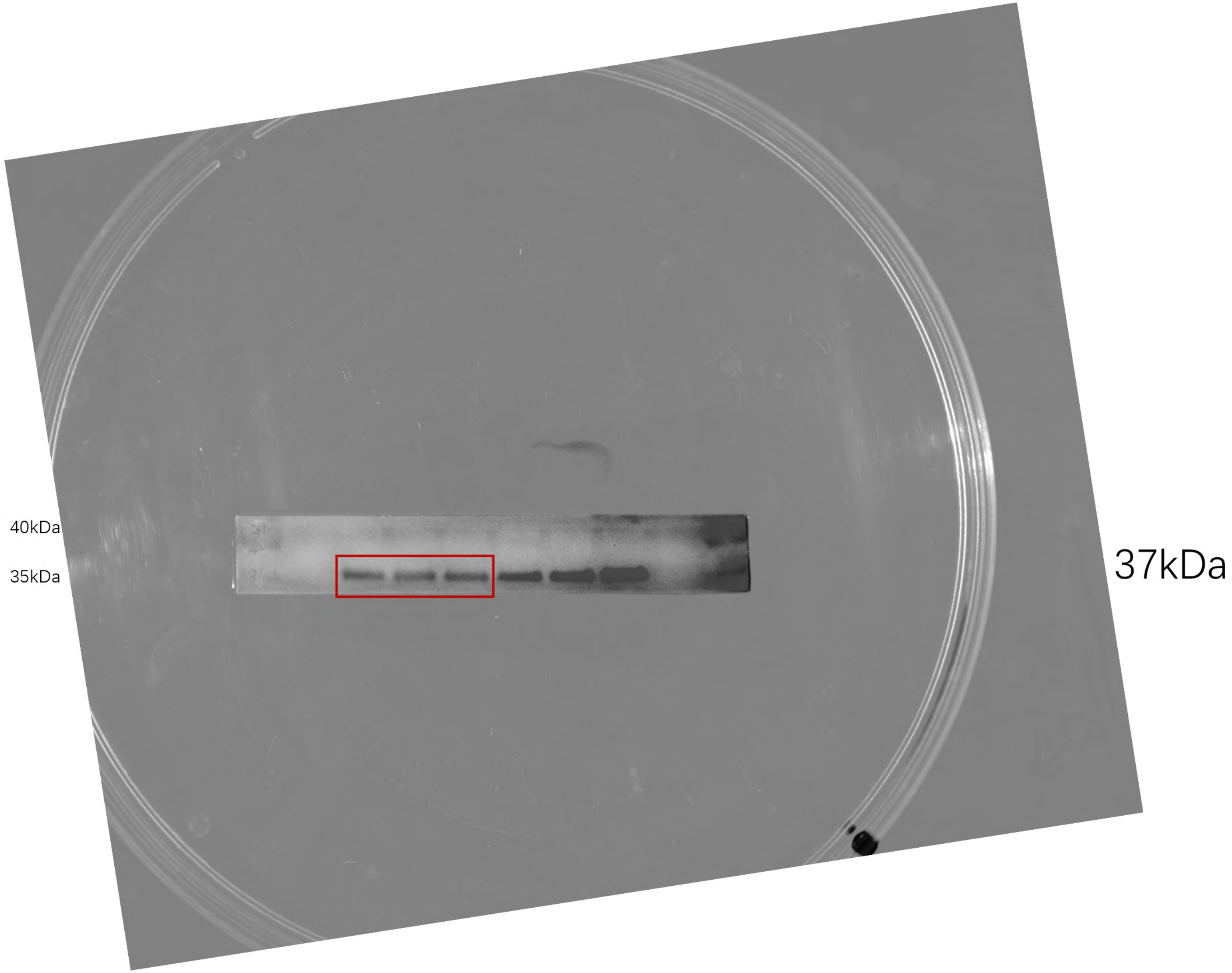

Source Fig.3C p-NFkB p65

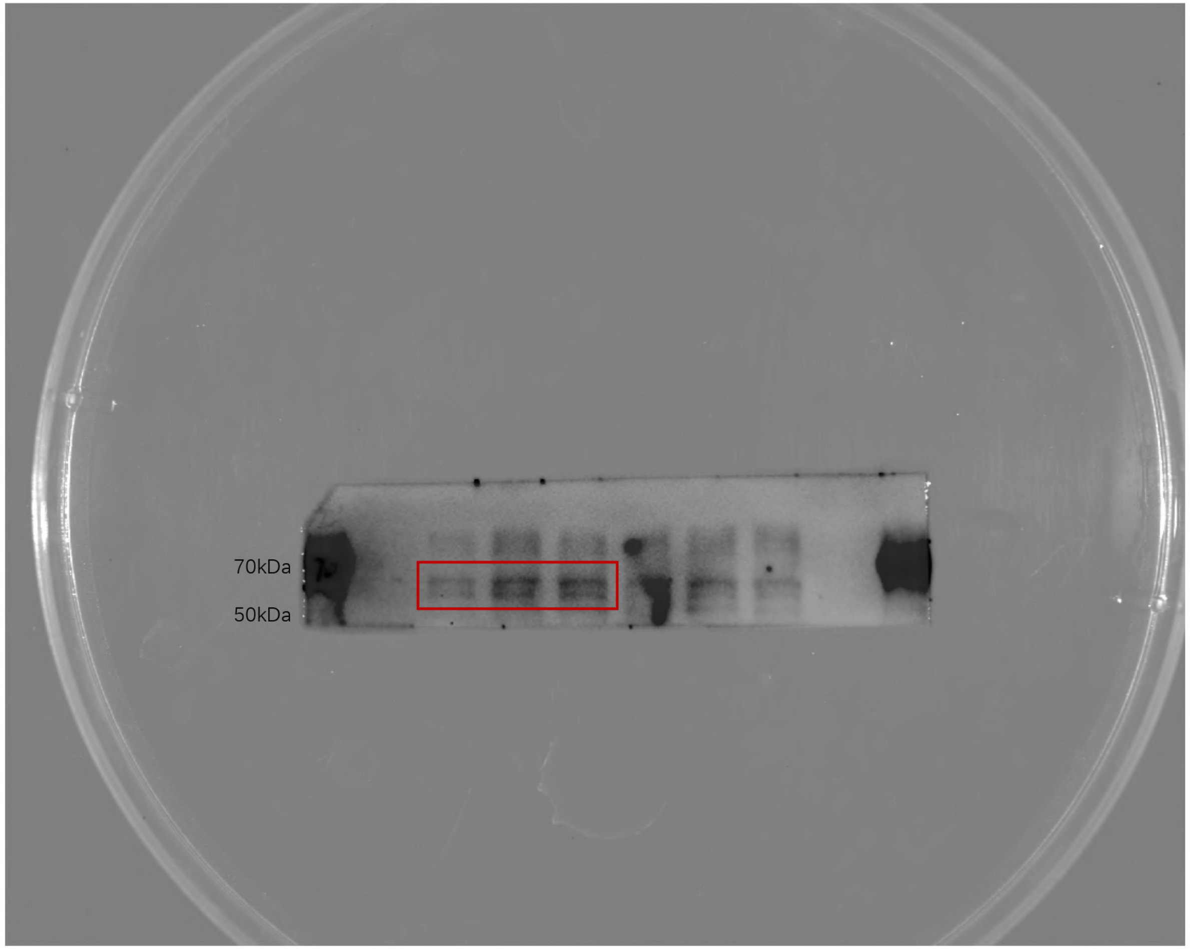

65kDa

Source Fig.3C GAPDH

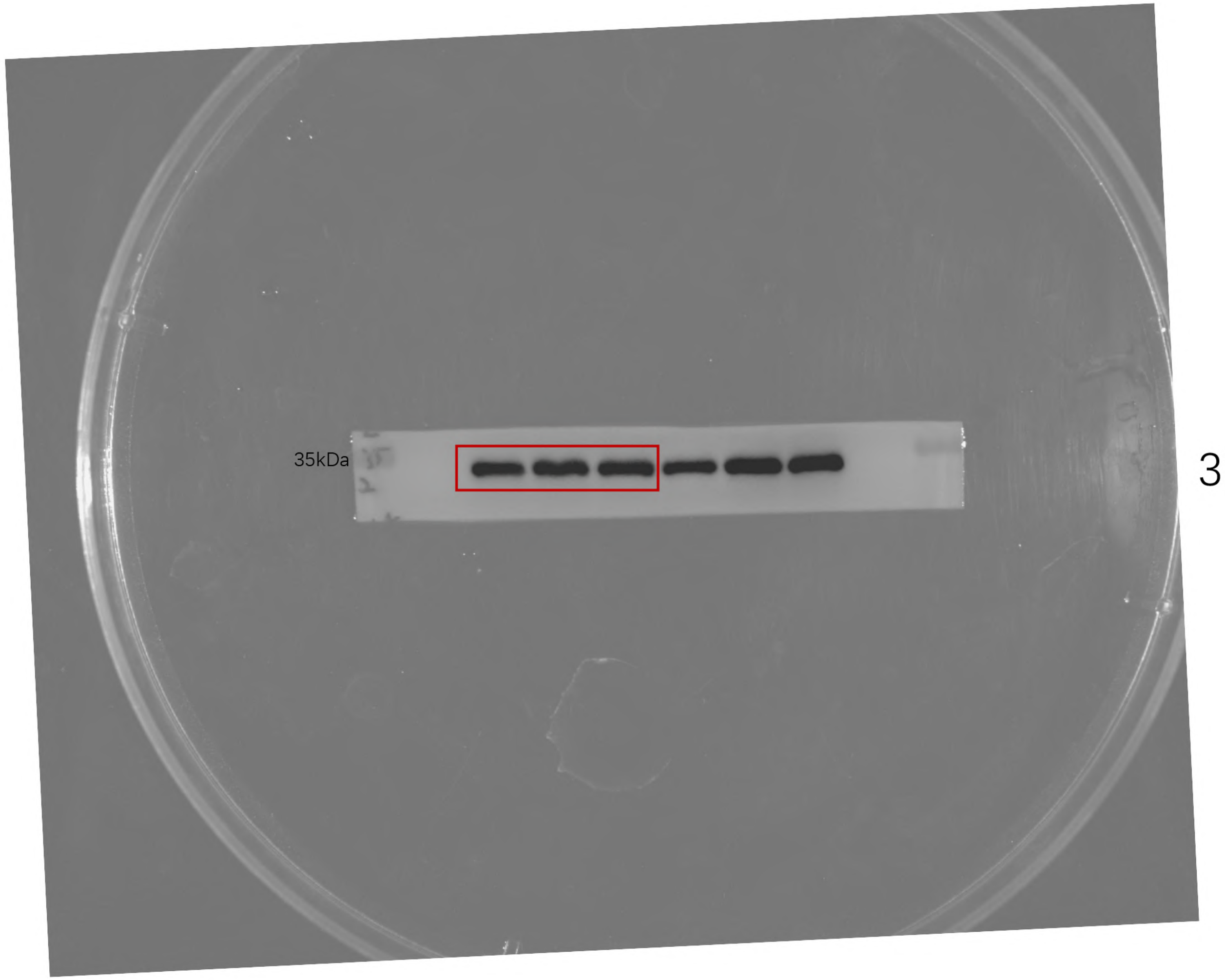

Source Fig.3C NFkB p65

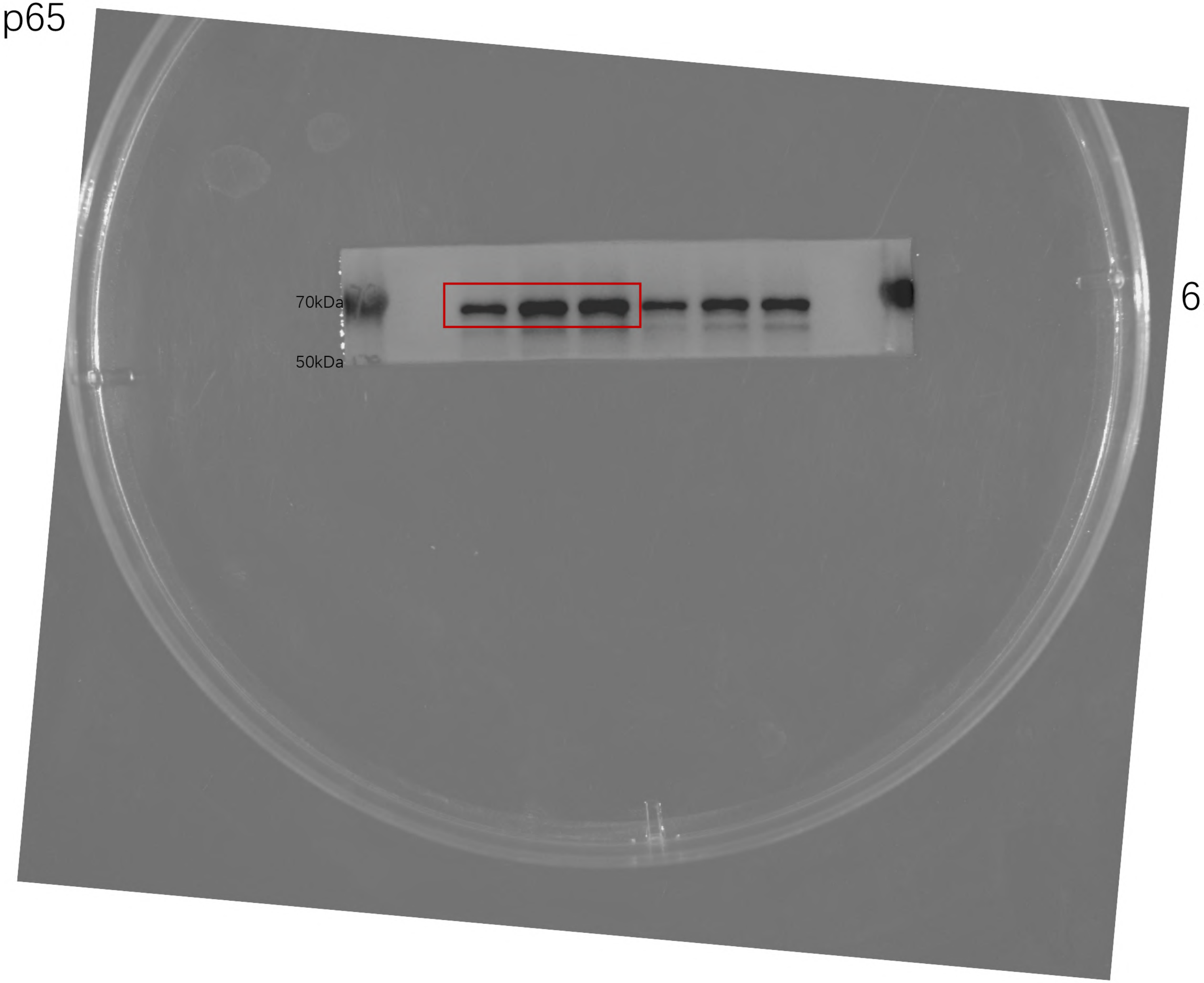

65kDa

Source Fig.3C GAPDH

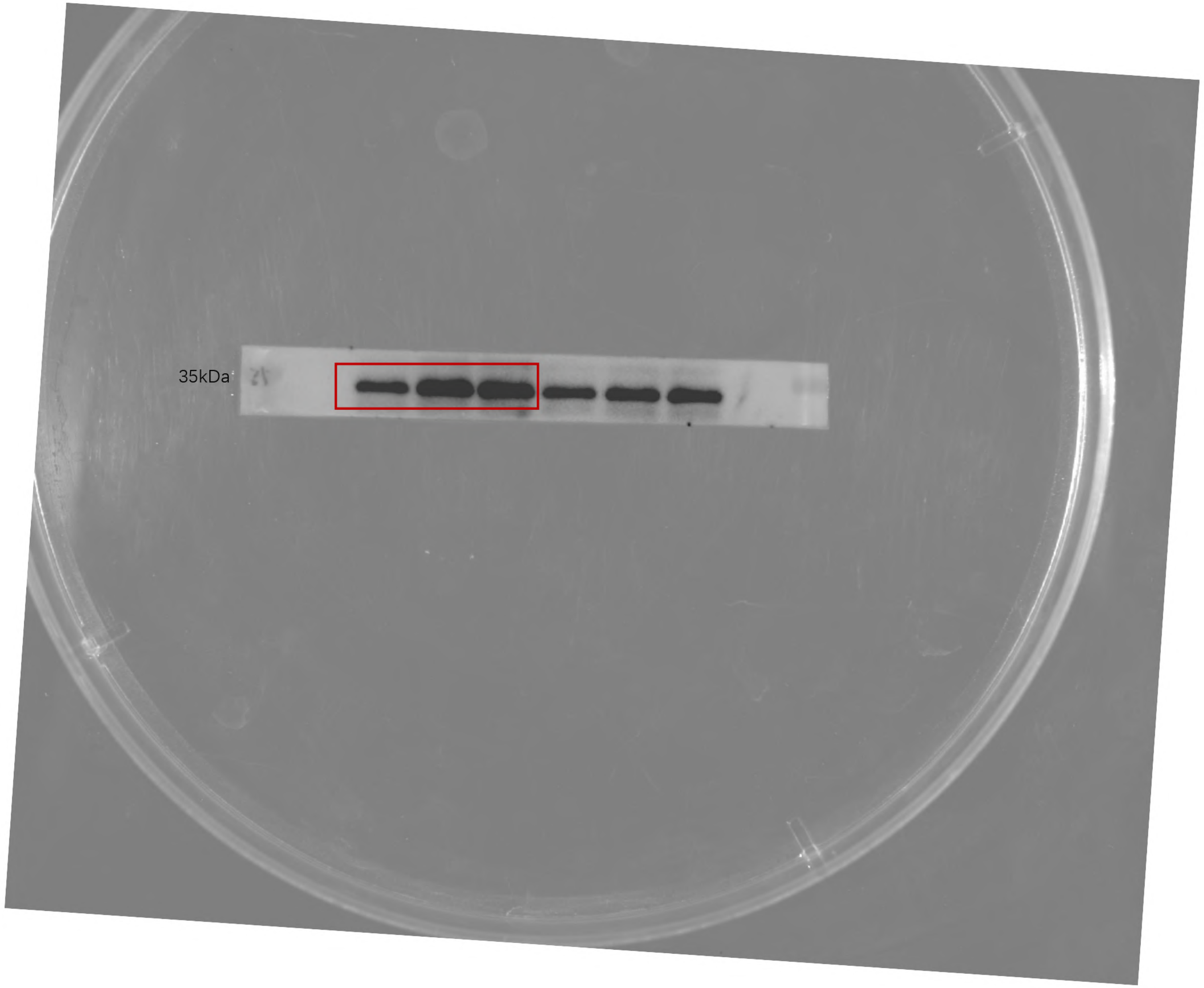

37kDa

Source Fig.3C p-TRAF2

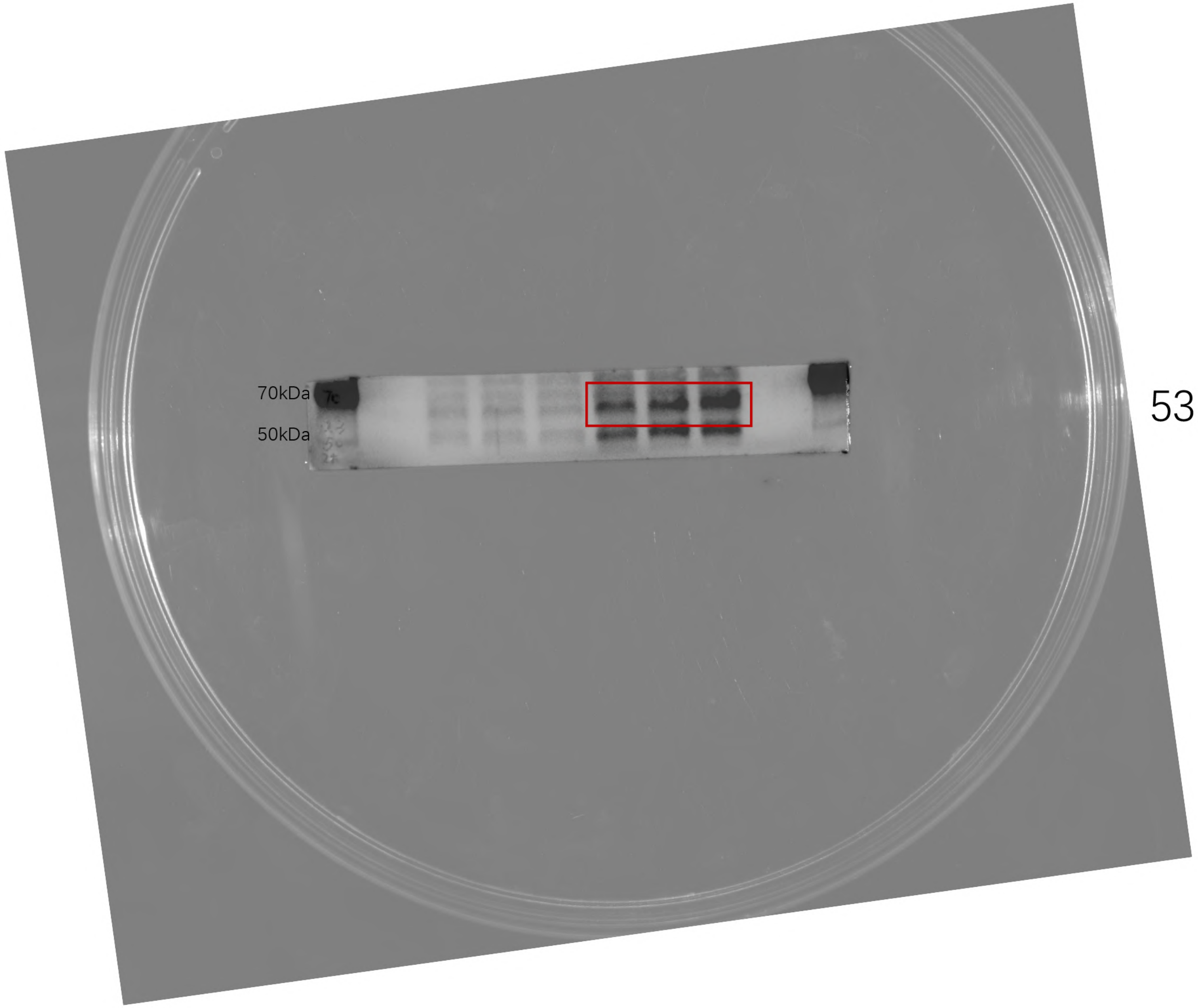

Source Fig.3C TRAF2

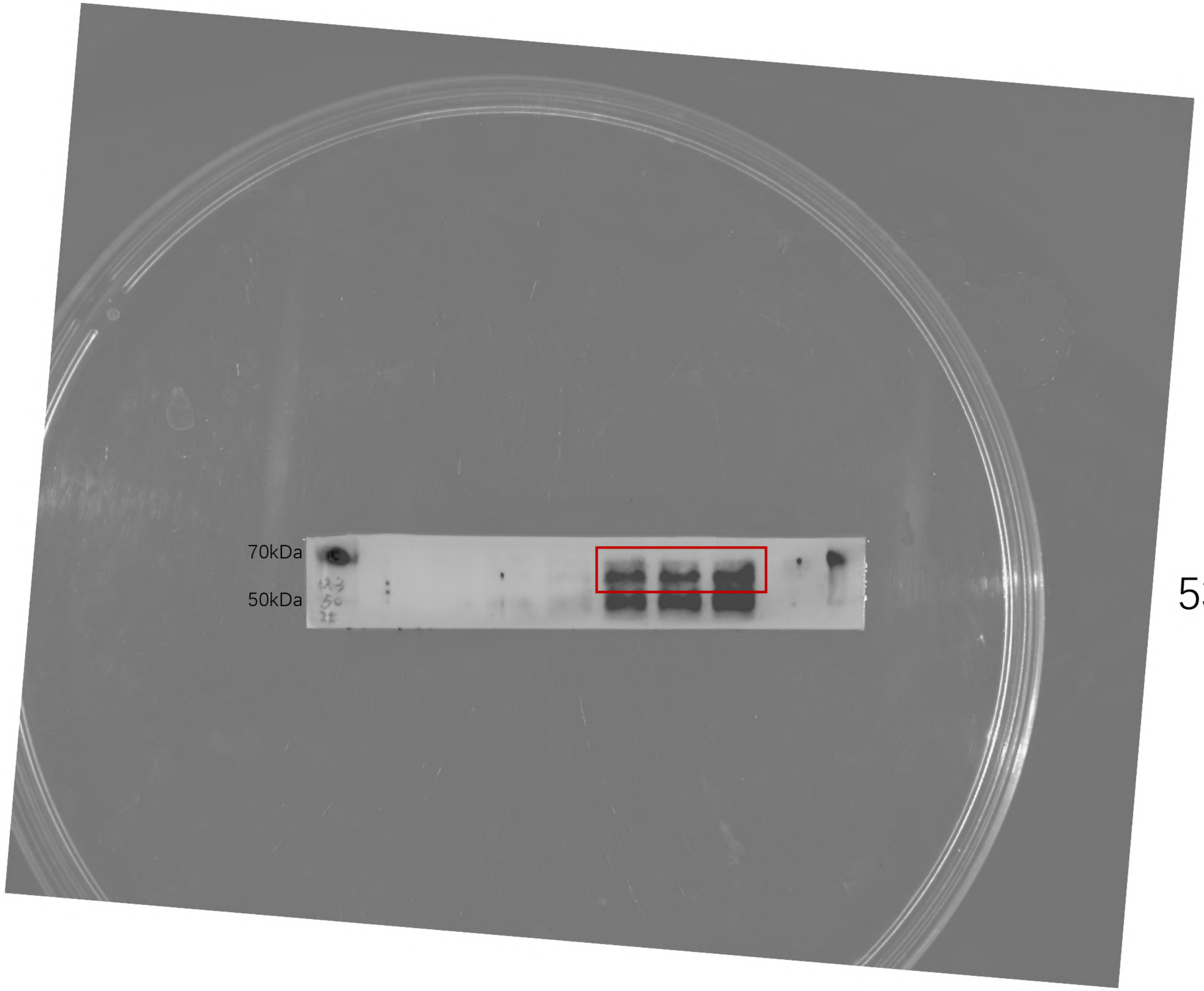

Source Fig.3C GAPDH

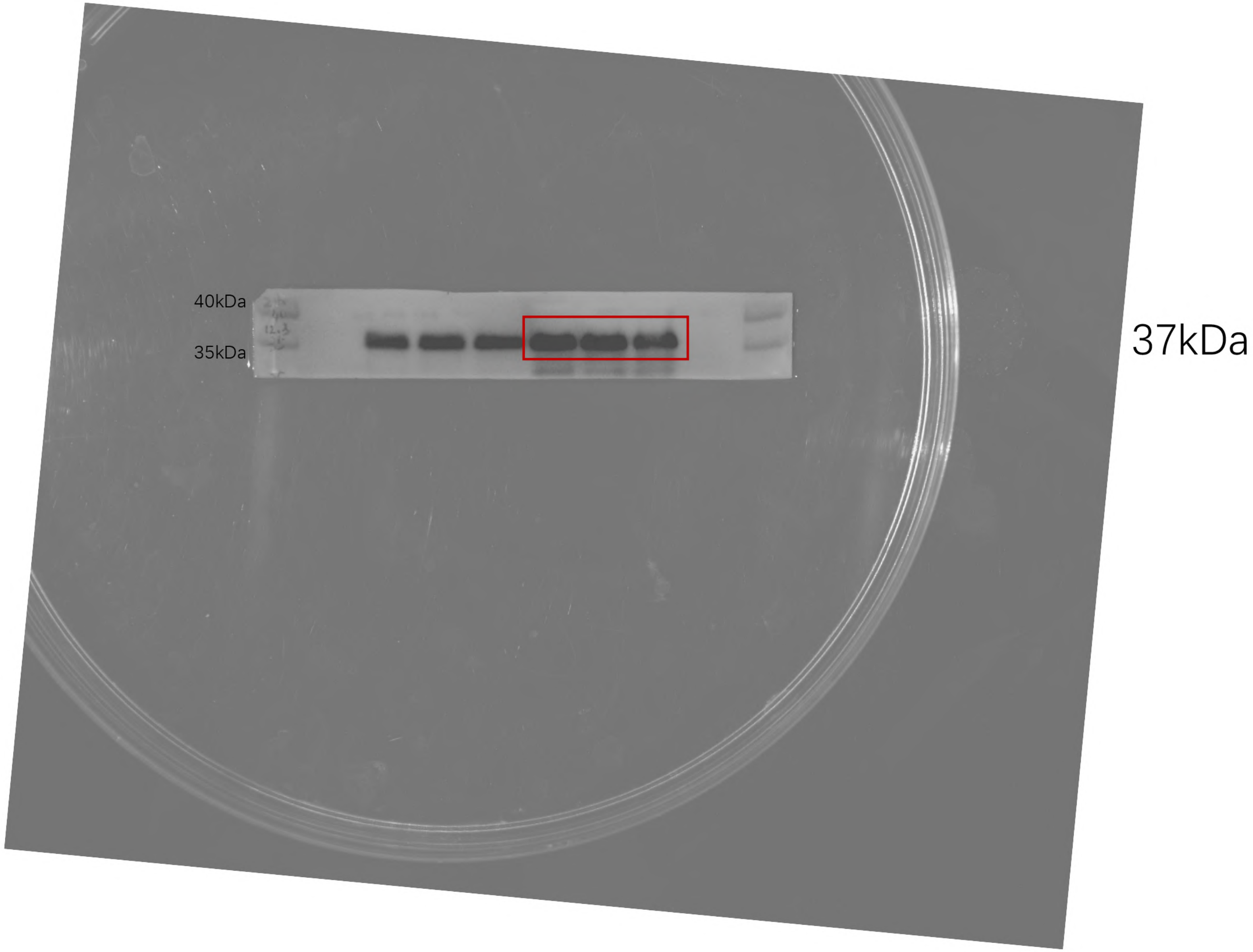

Source Fig.3C 24P3R

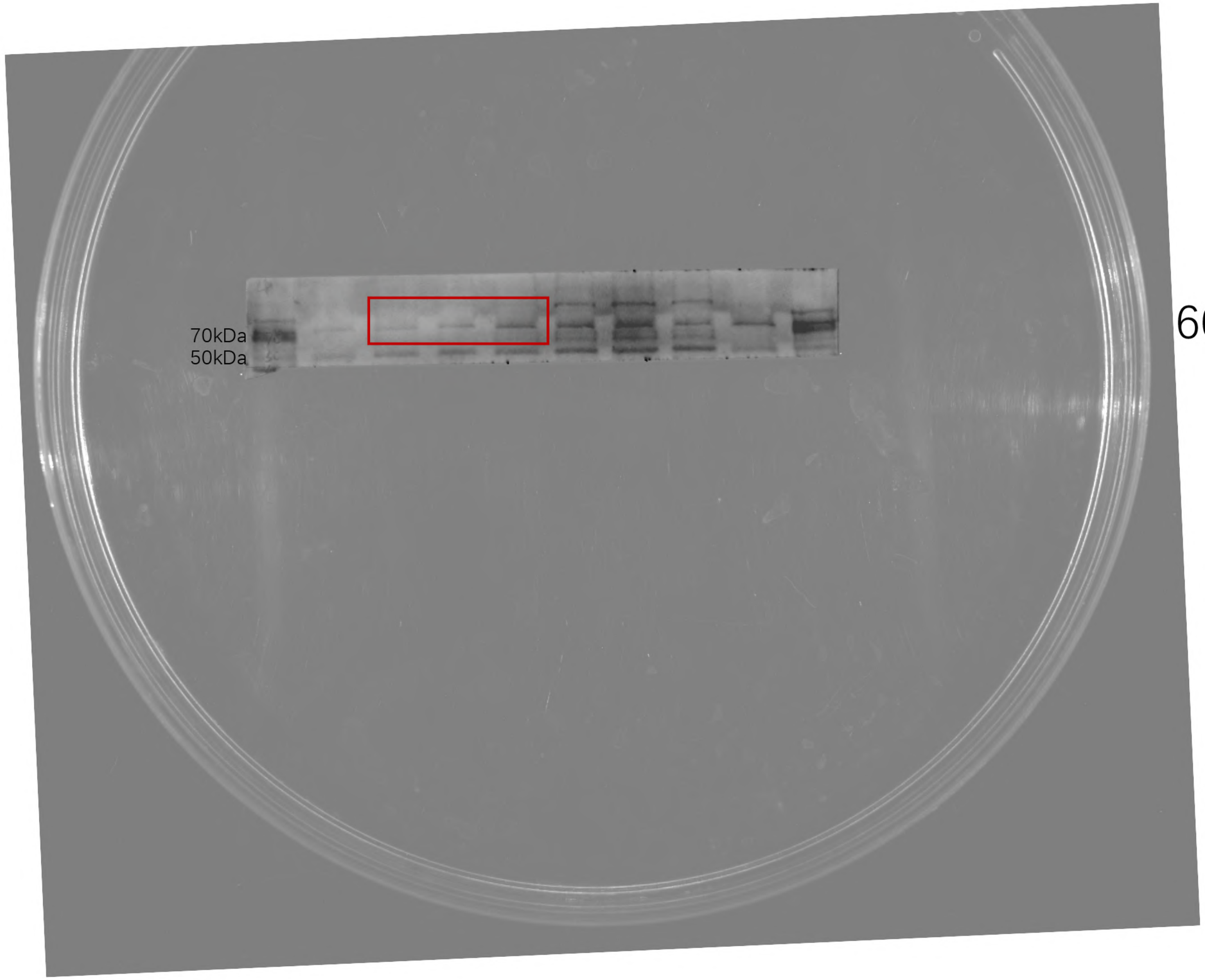

Source Fig.3C GAPDH

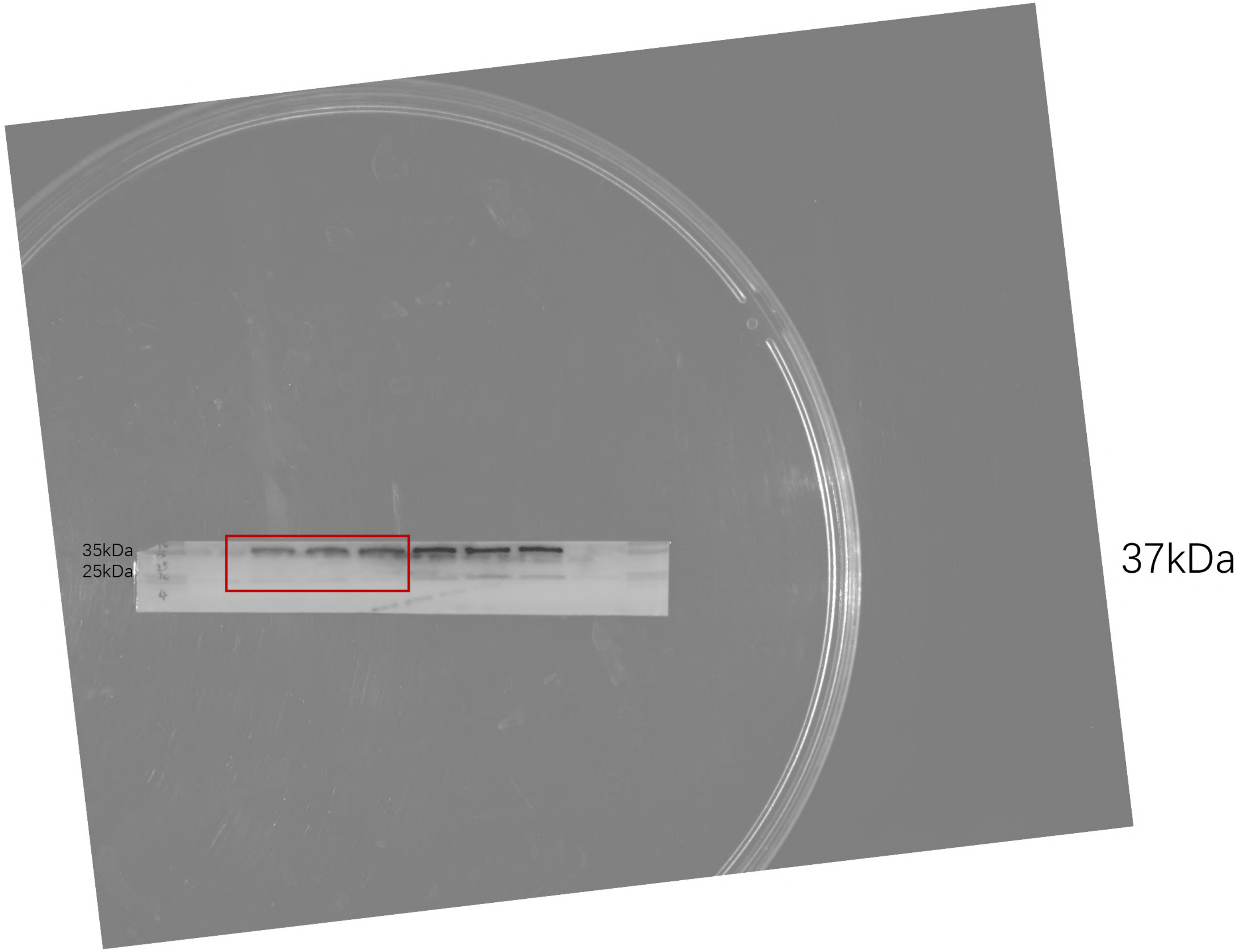

Source Fig.4B p-ERK1/2

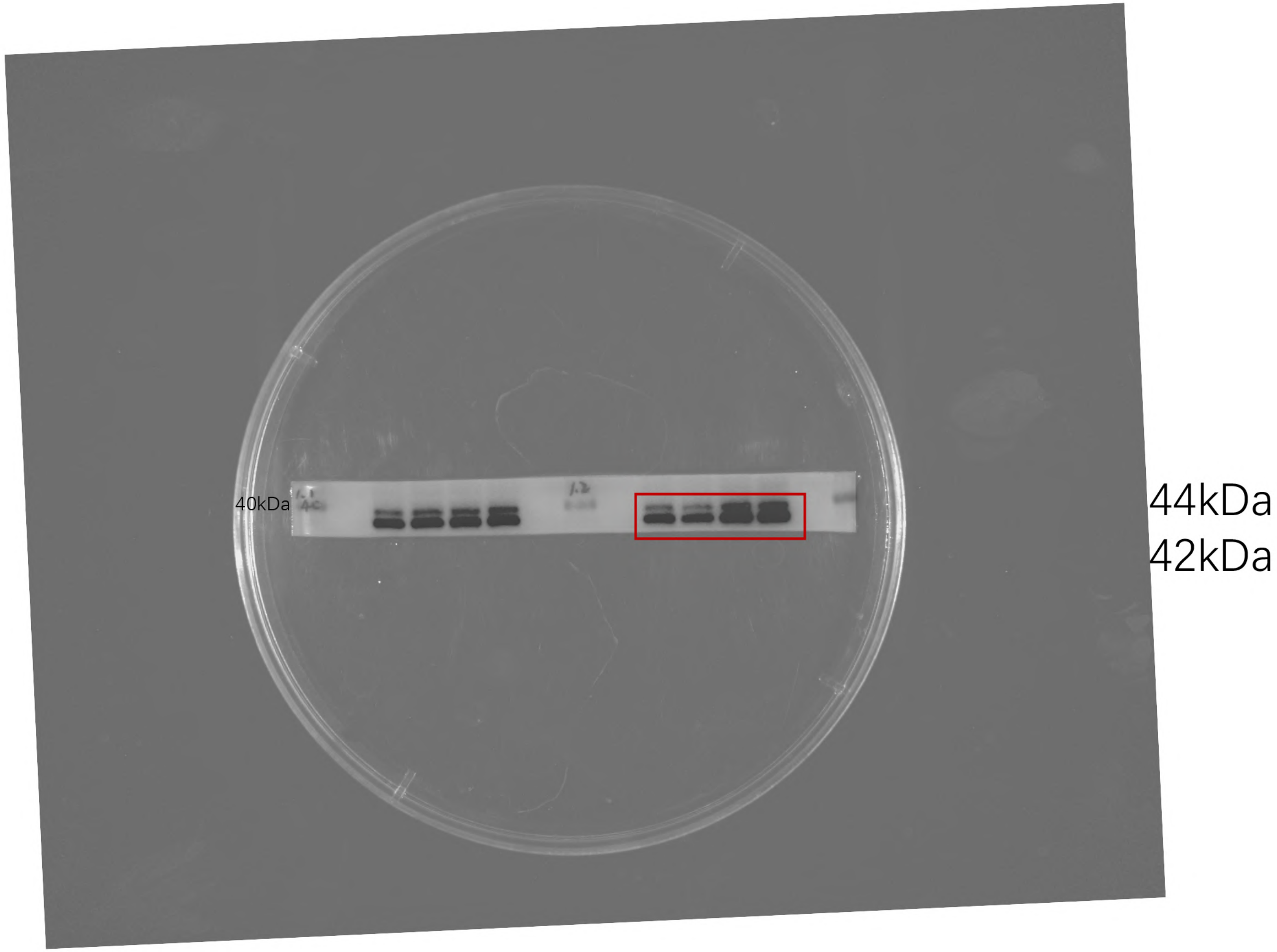

Source Fig.4B ERK1/2

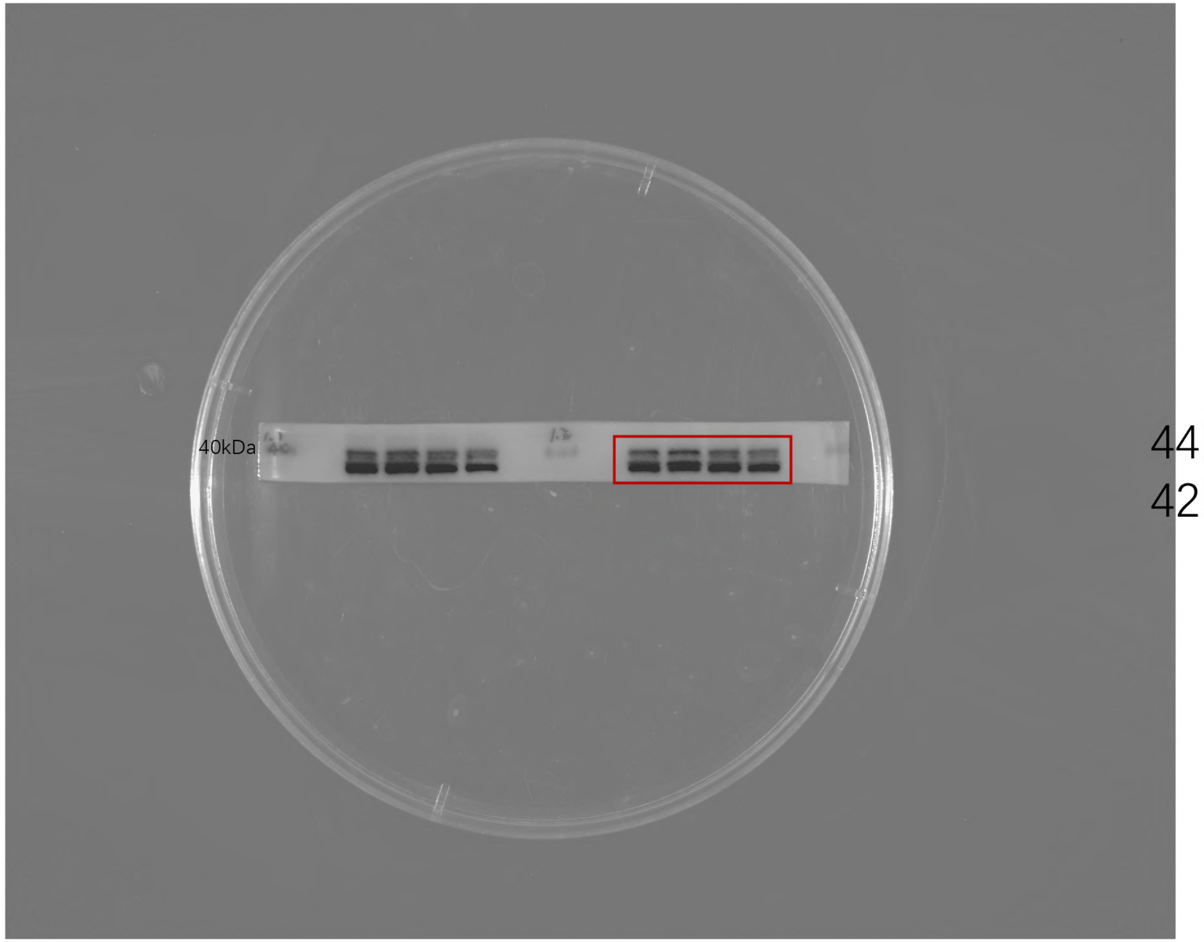

Source Fig.4B p-JNK

70kDa
50kDa
40kDa

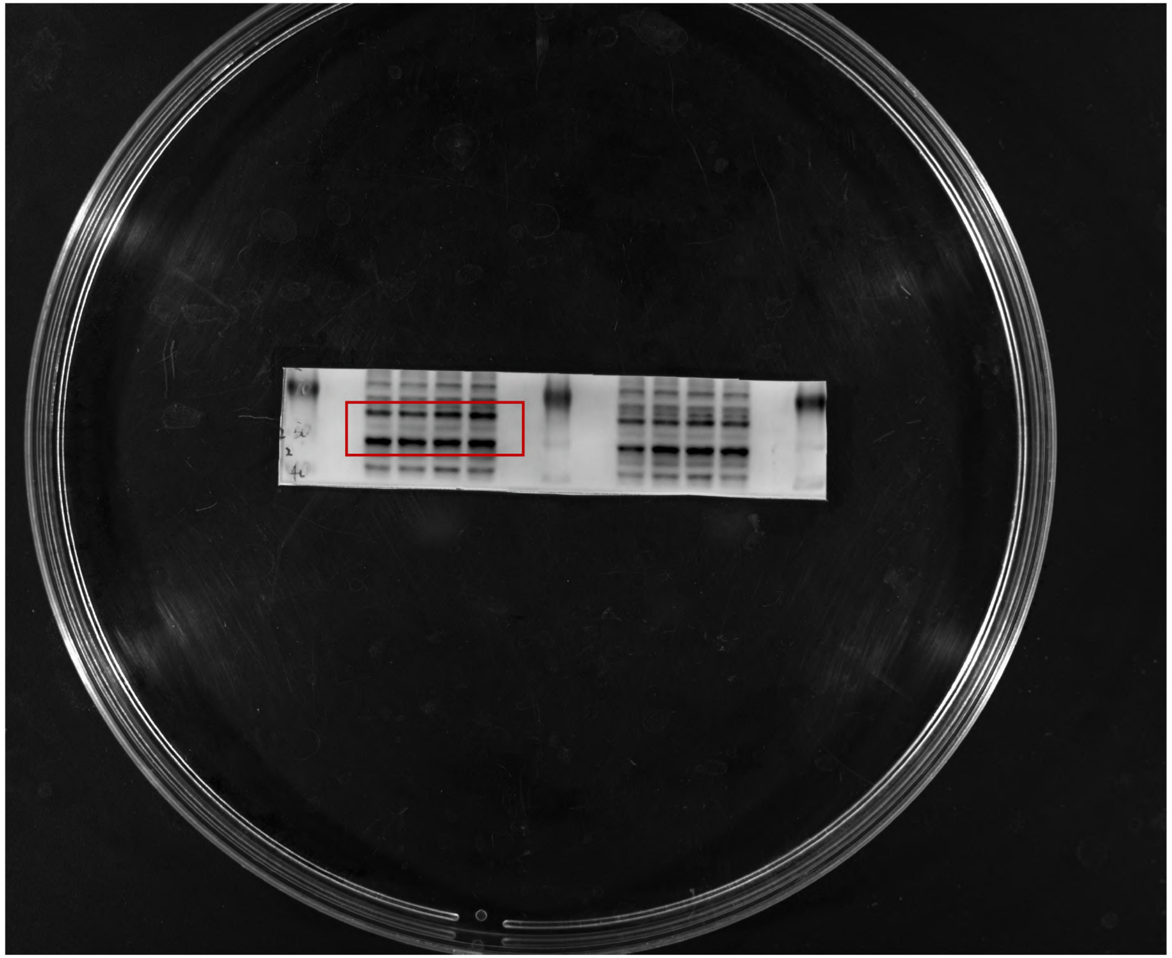

54kDa
46kDa

Source Fig.4B JNK

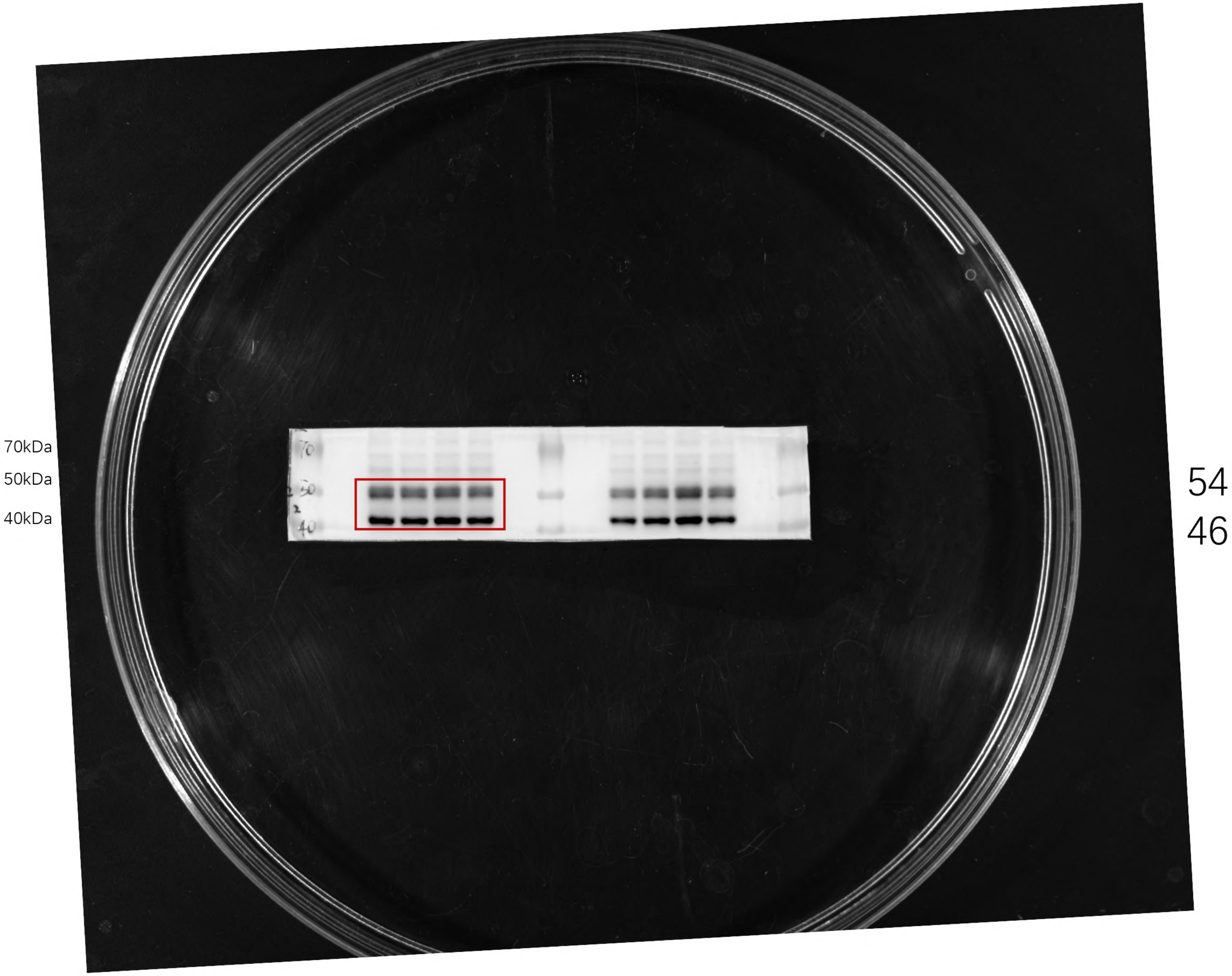

Source Fig.4B p-p38

40kDa
35kDa
25kDa

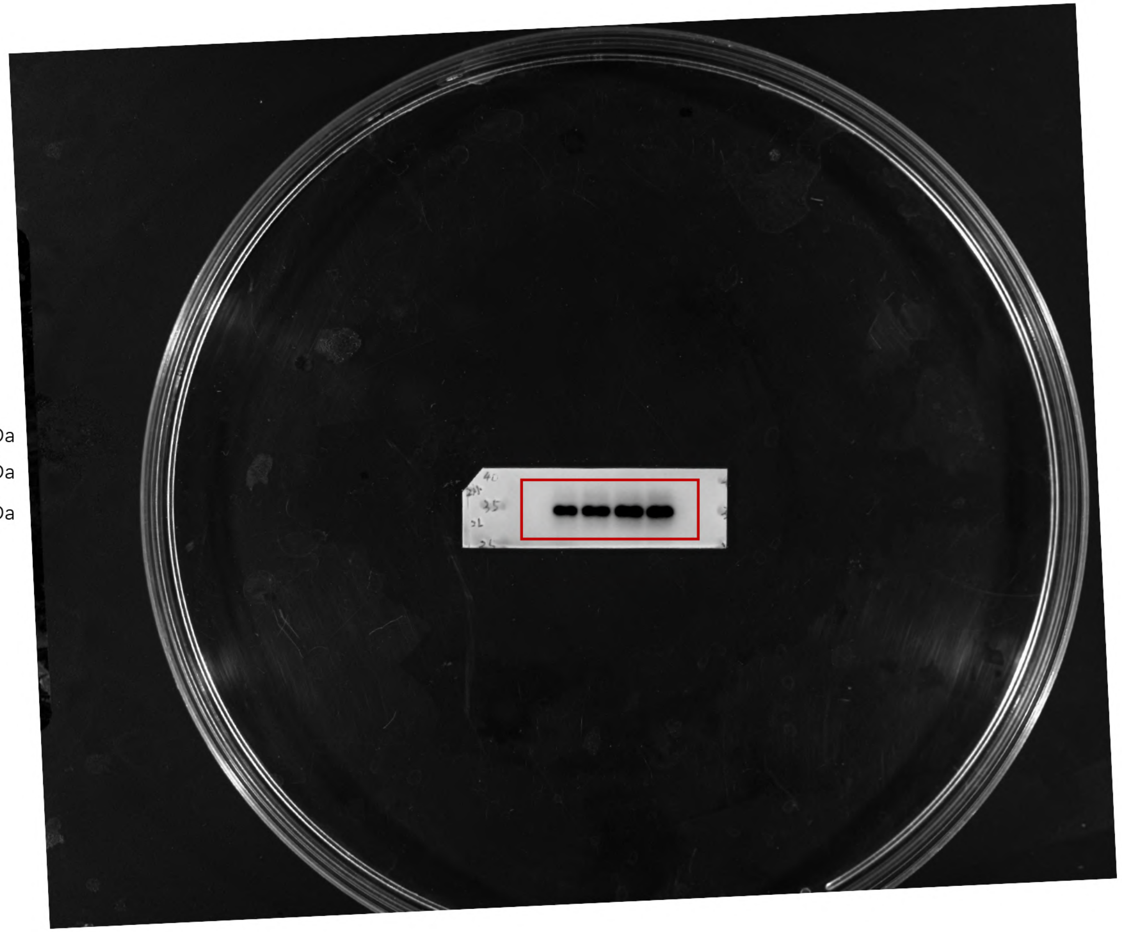

38kDa

Source Fig.4B GAPDH

40kDa
35kDa
25kDa

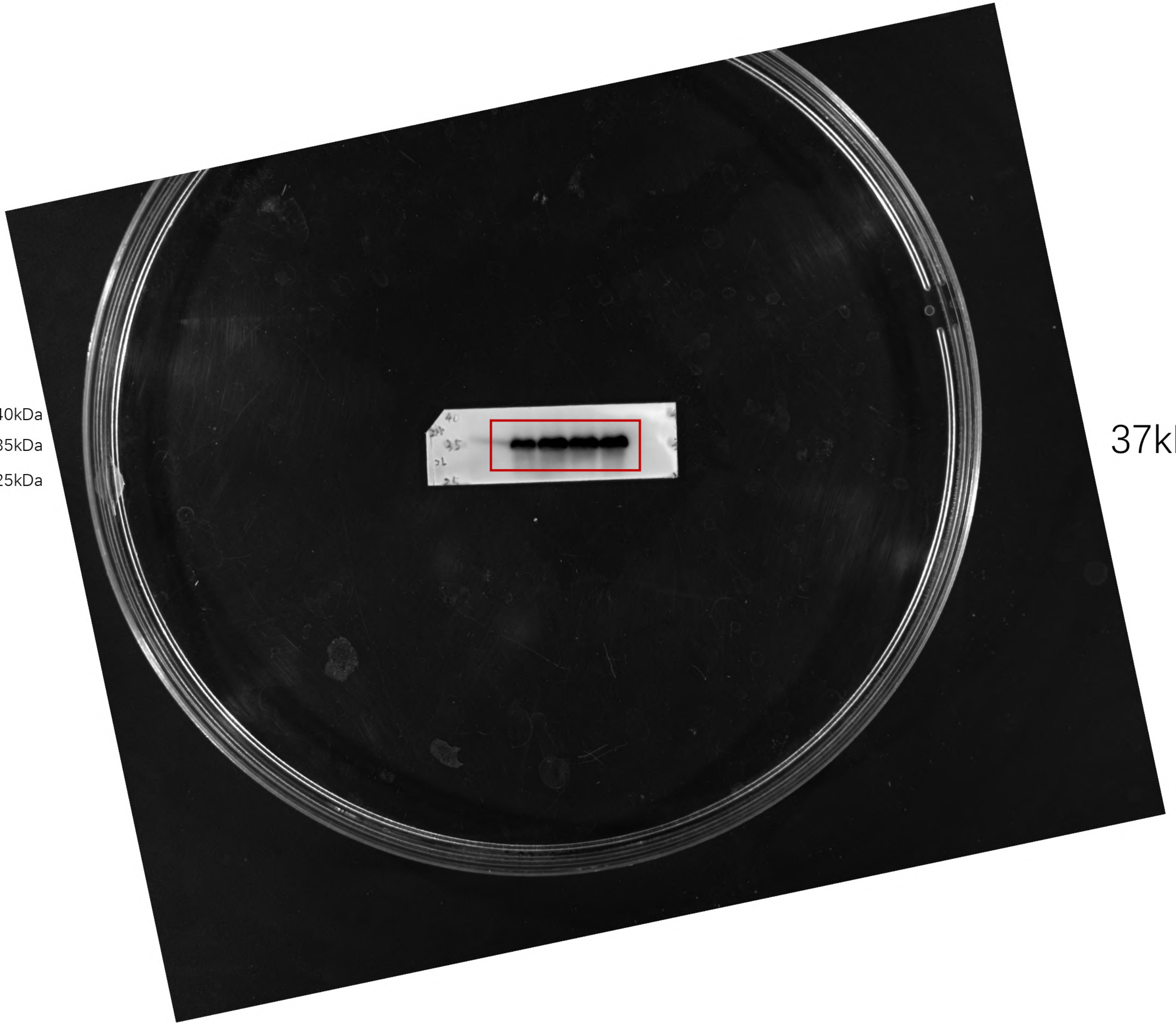

37kDa

Source Fig.4B p38

40kDa
35kDa
25kDa

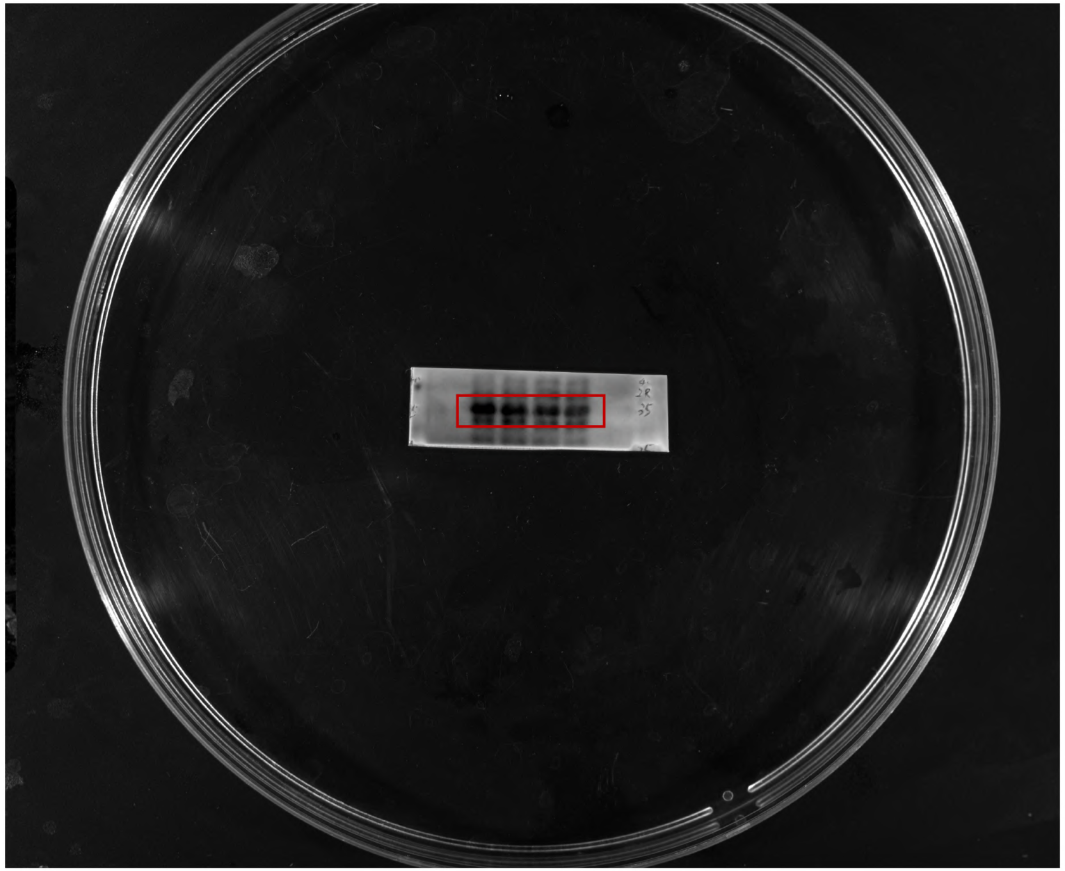

38kDa

Source Fig.4B GAPDH

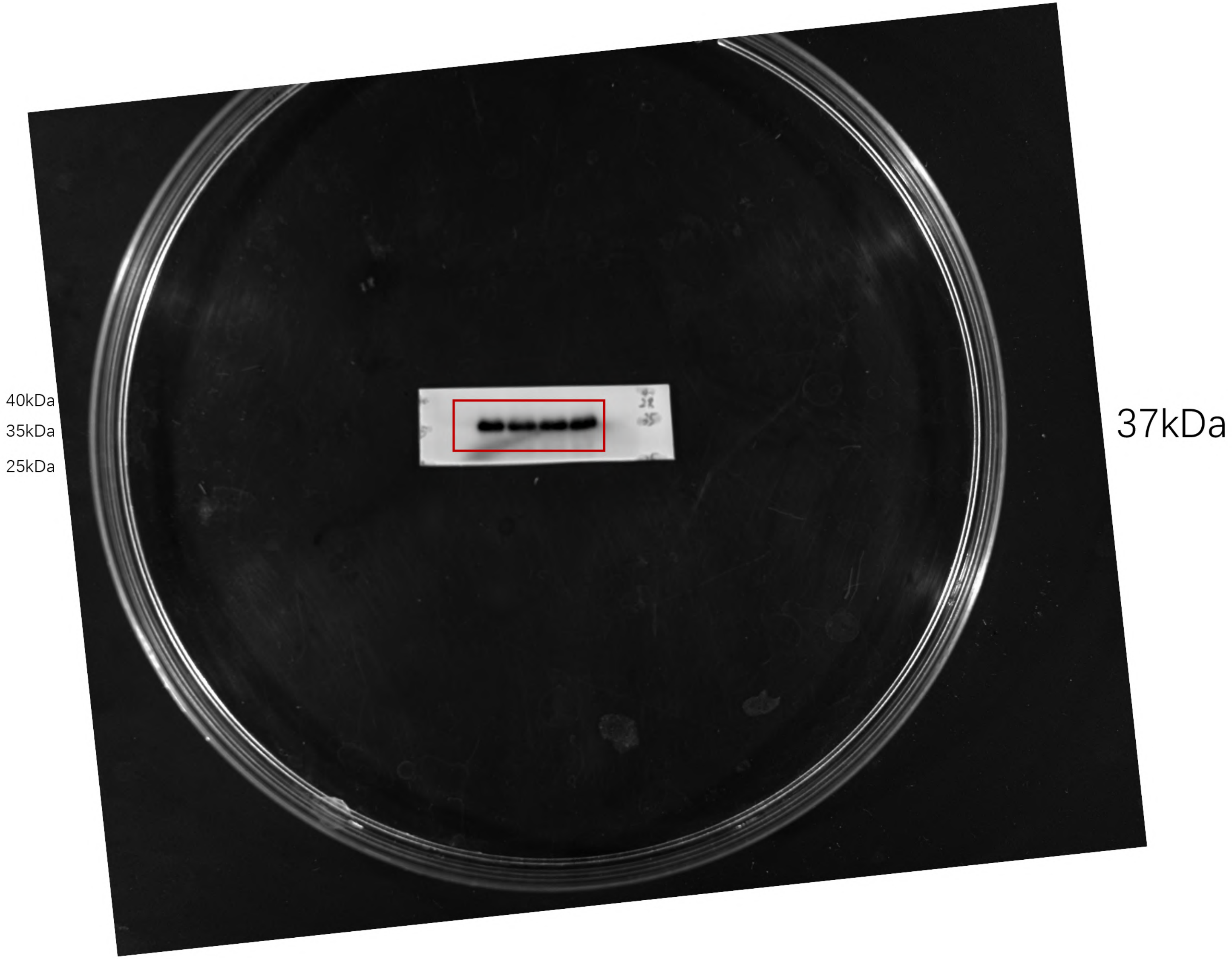

Source Fig.4B p-TRAF2

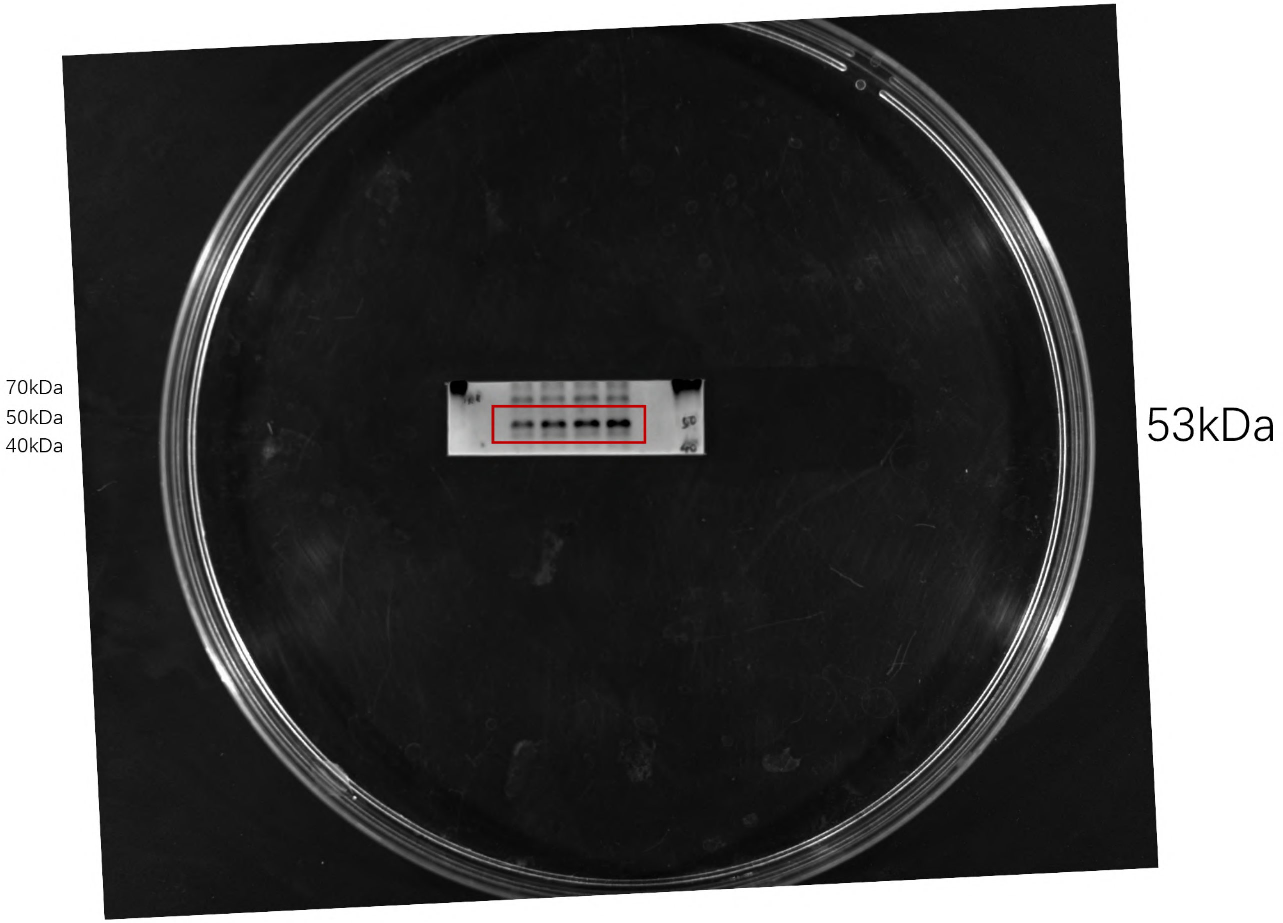

Source Fig.4B TRAF2

70kDa
50kDa
40kDa

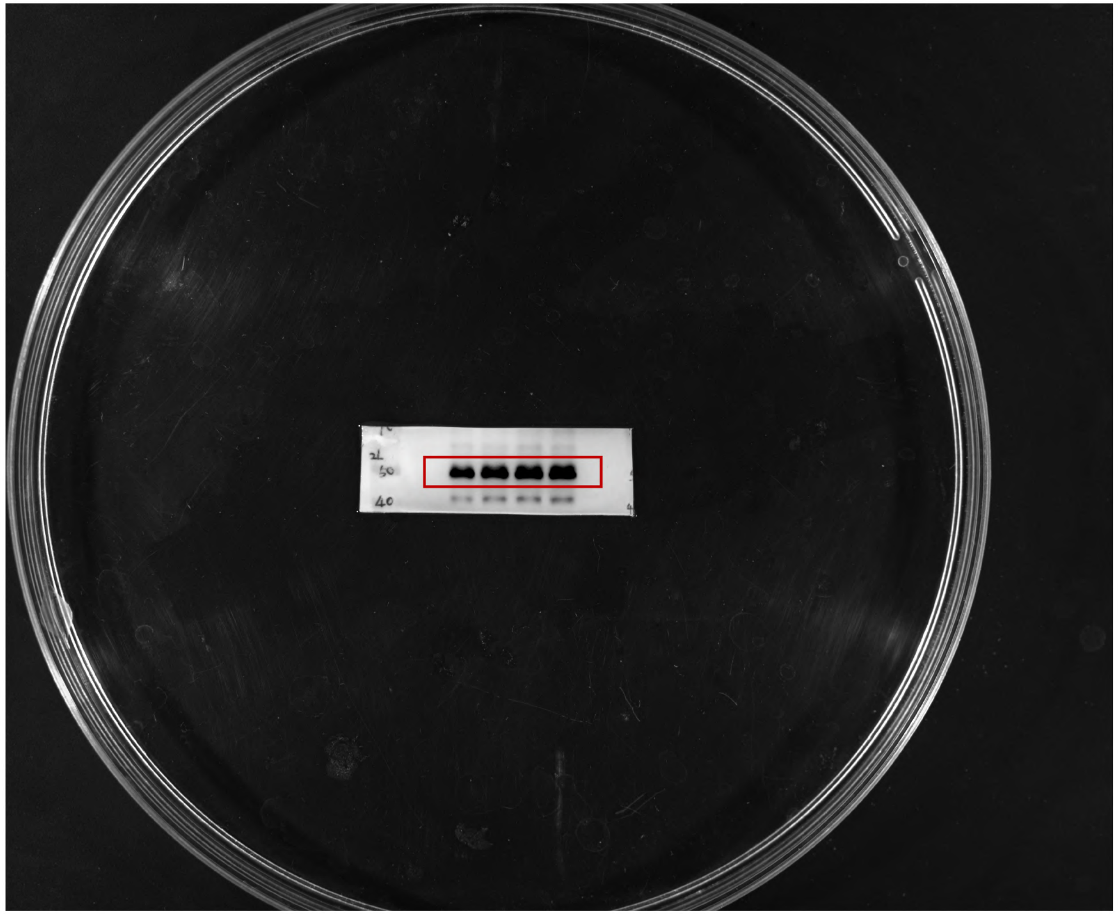

53kDa

Source Fig.4B GAPDH

40kDa
35kDa

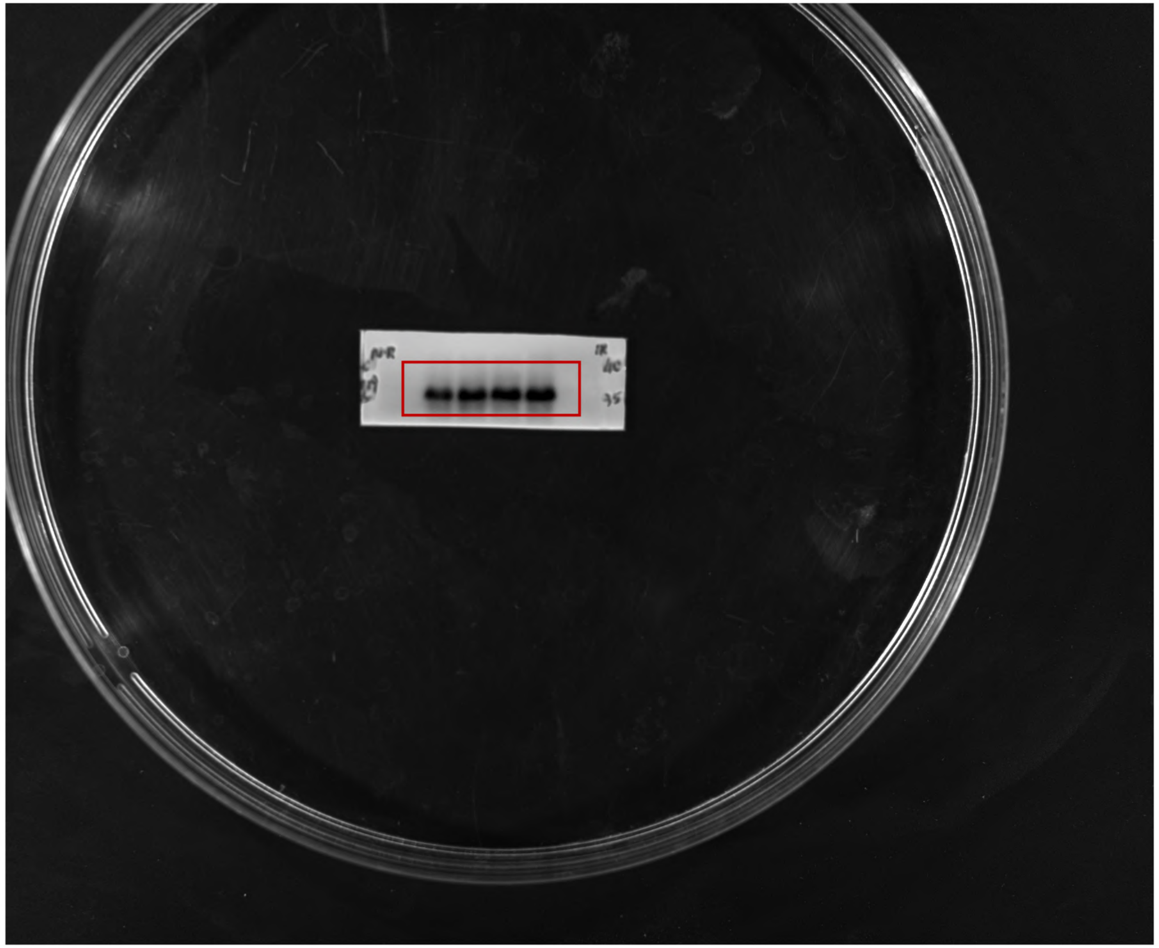

37kDa

Source Fig.4B Fn14

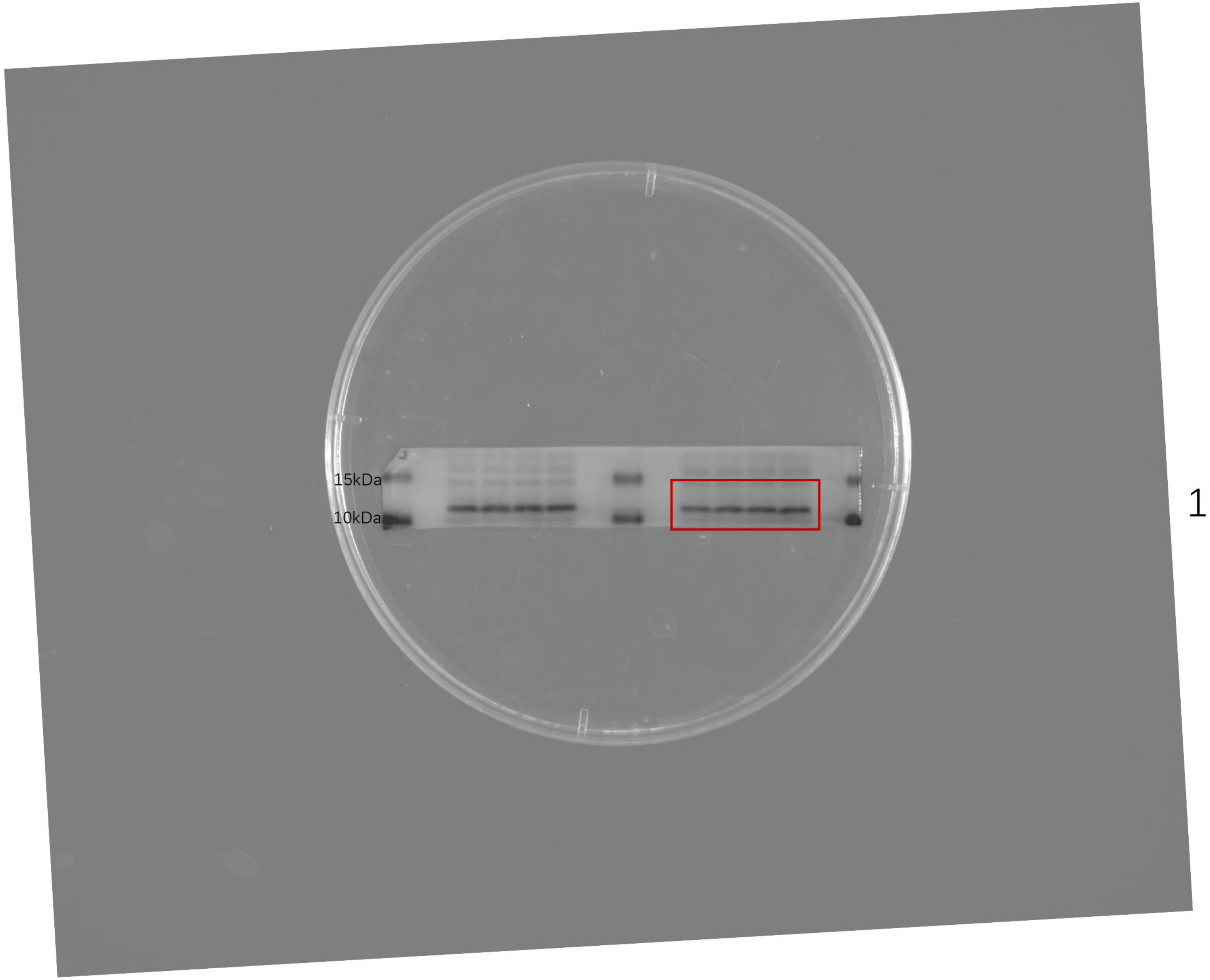

17kDa

Source Fig.4B GAPDH

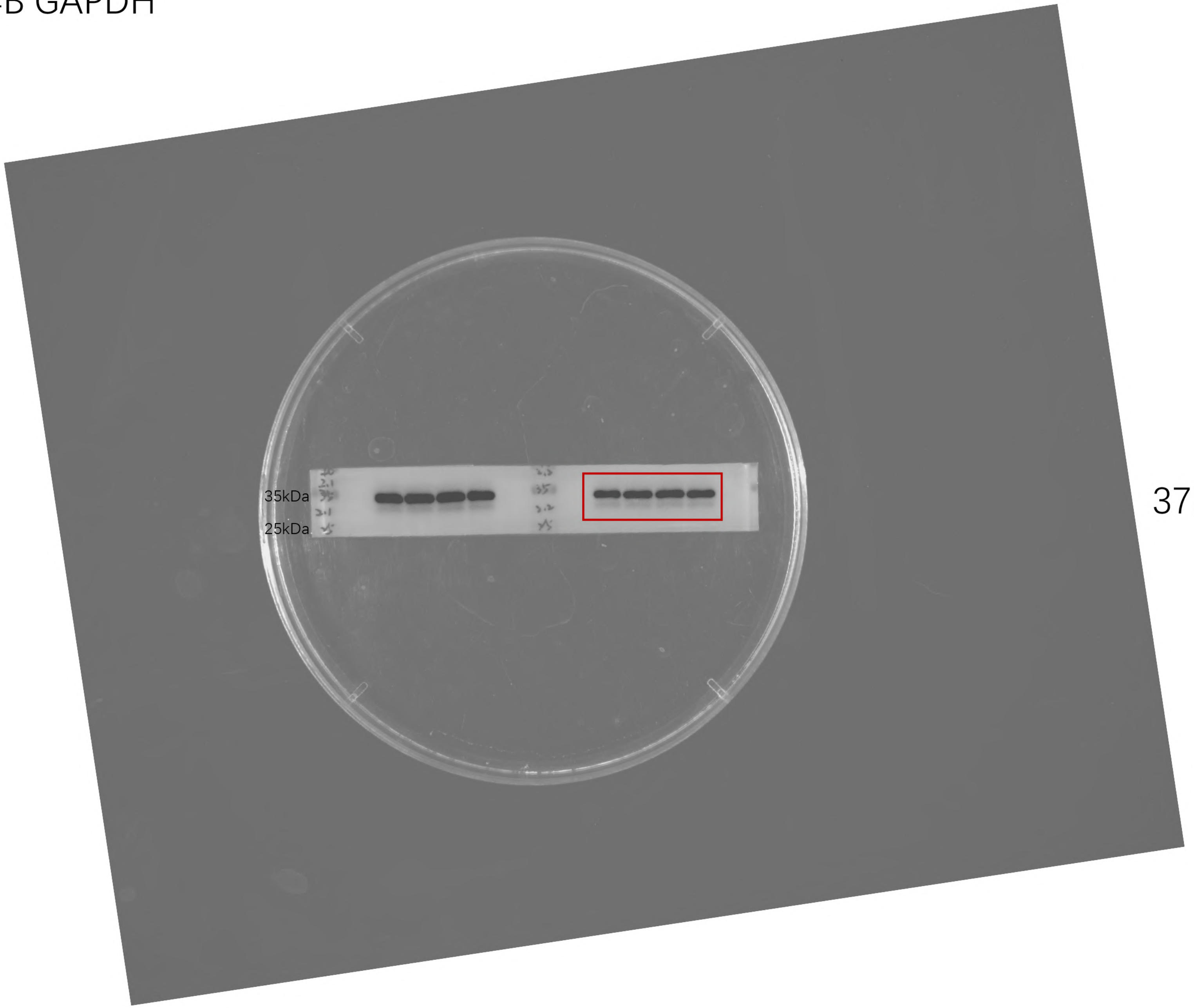

37kDa

Source Fig.4C p-ERK1/2

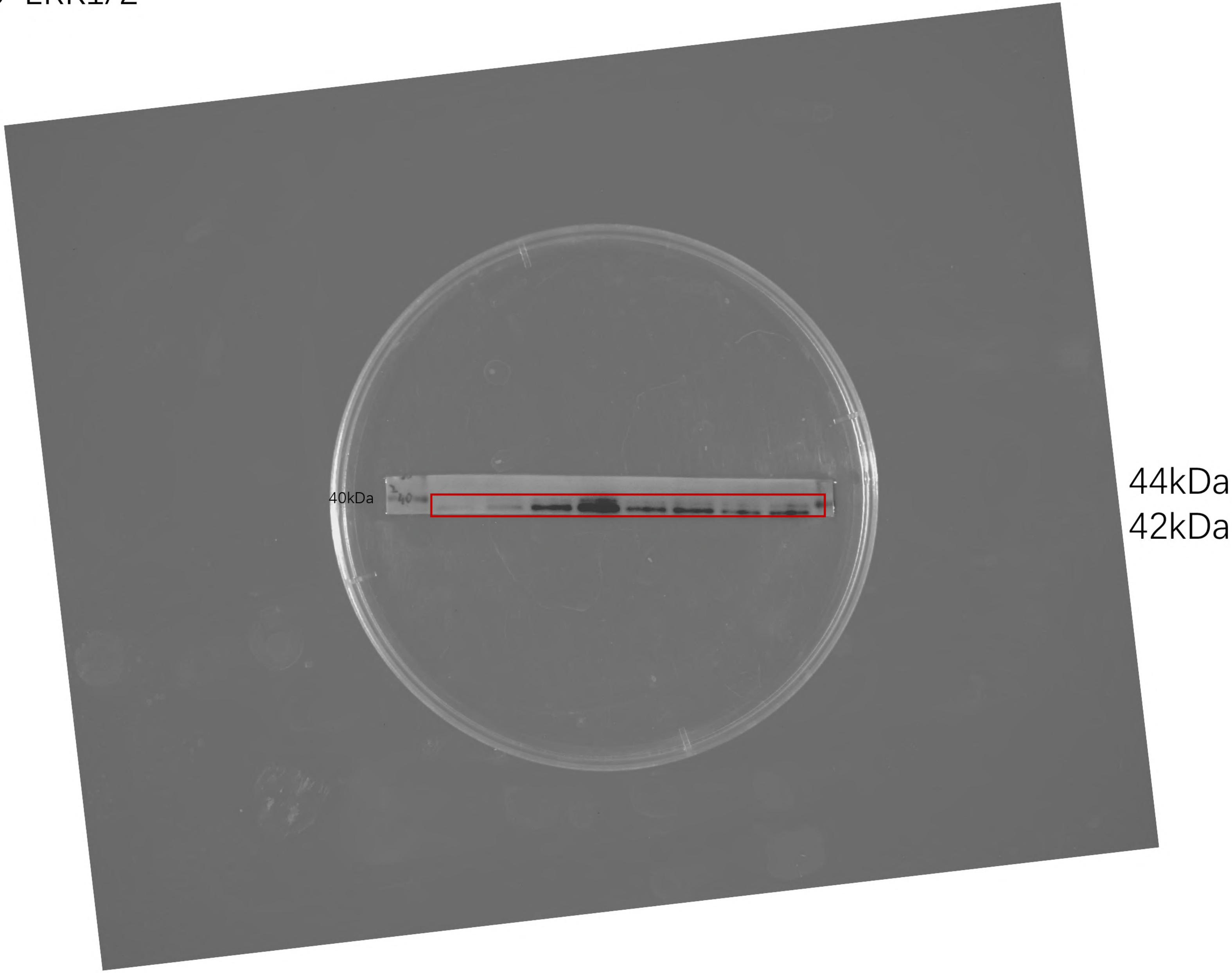

Source Fig.4C ERK1/2

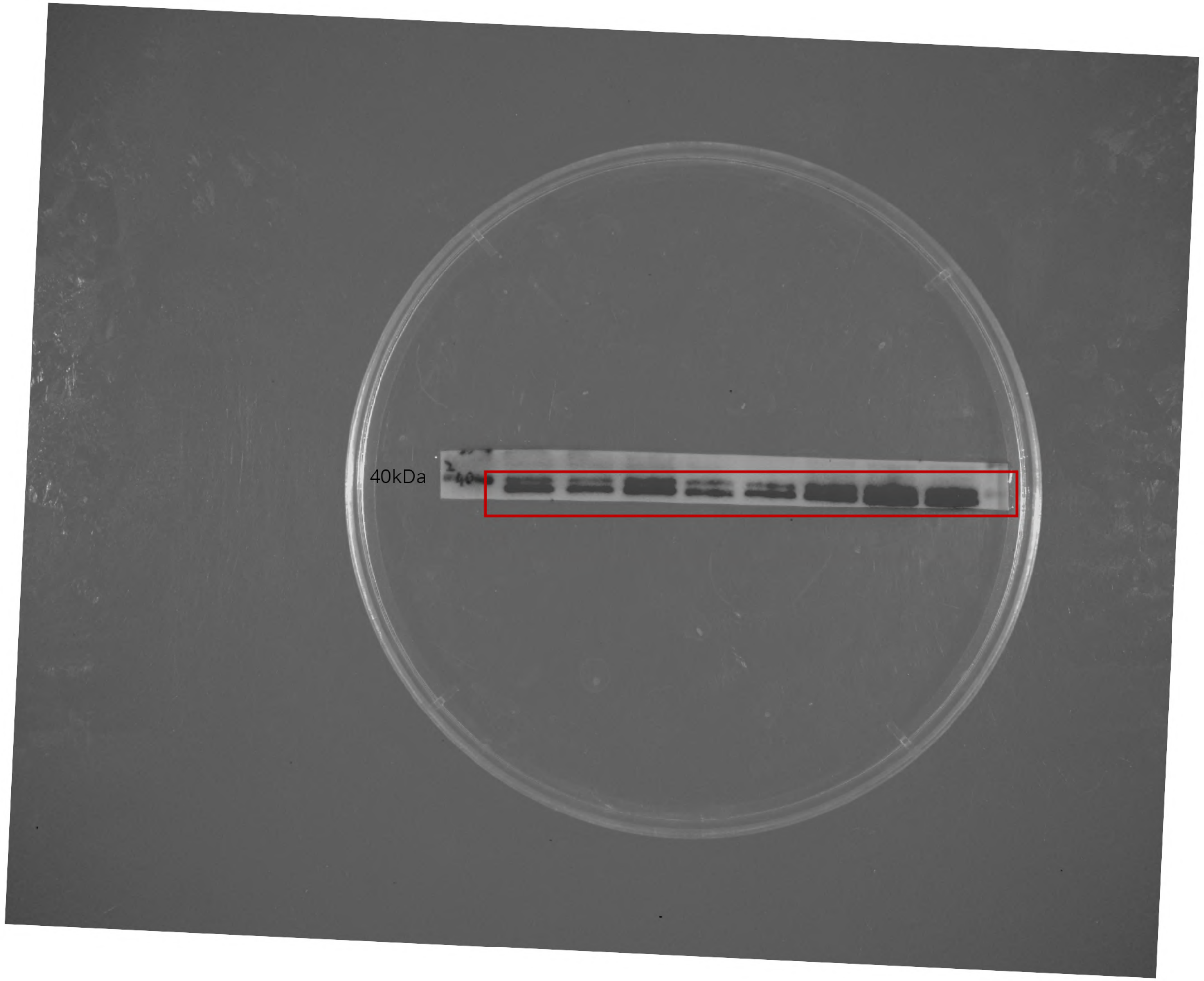

44kDa
42kDa

Source Fig.4C p-JNK

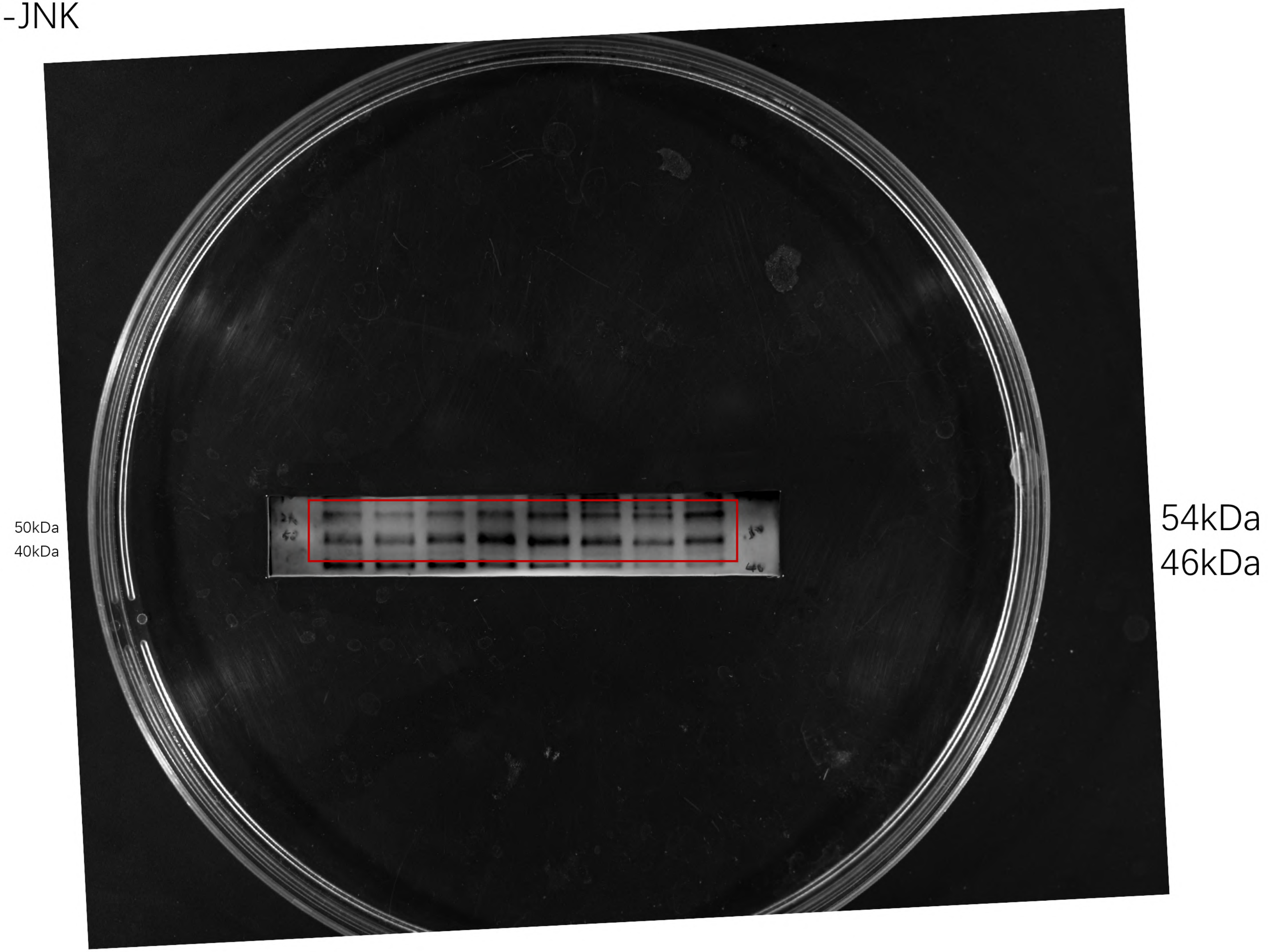

Source Fig.4C JNK

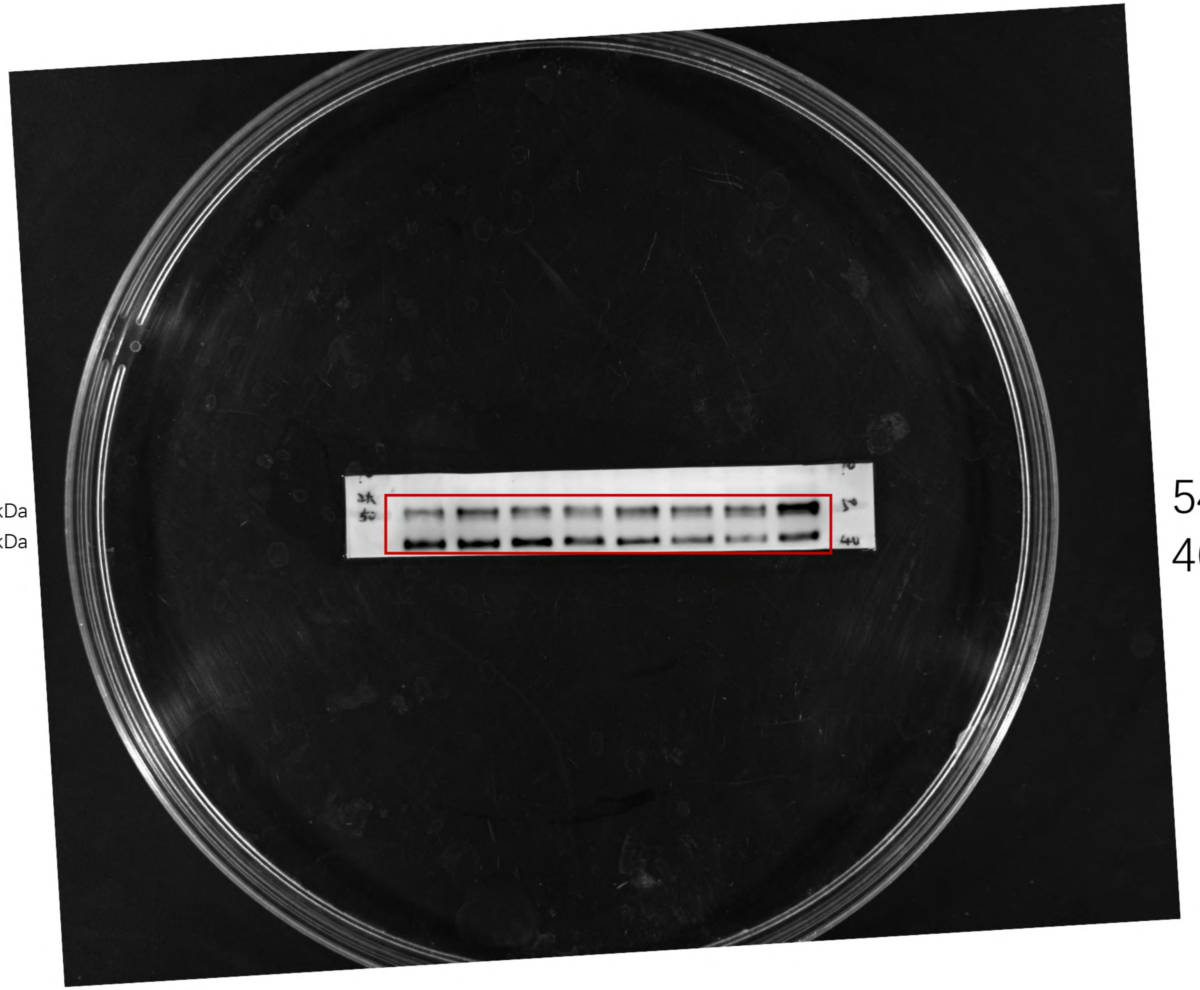

Source Fig.4C p-p38

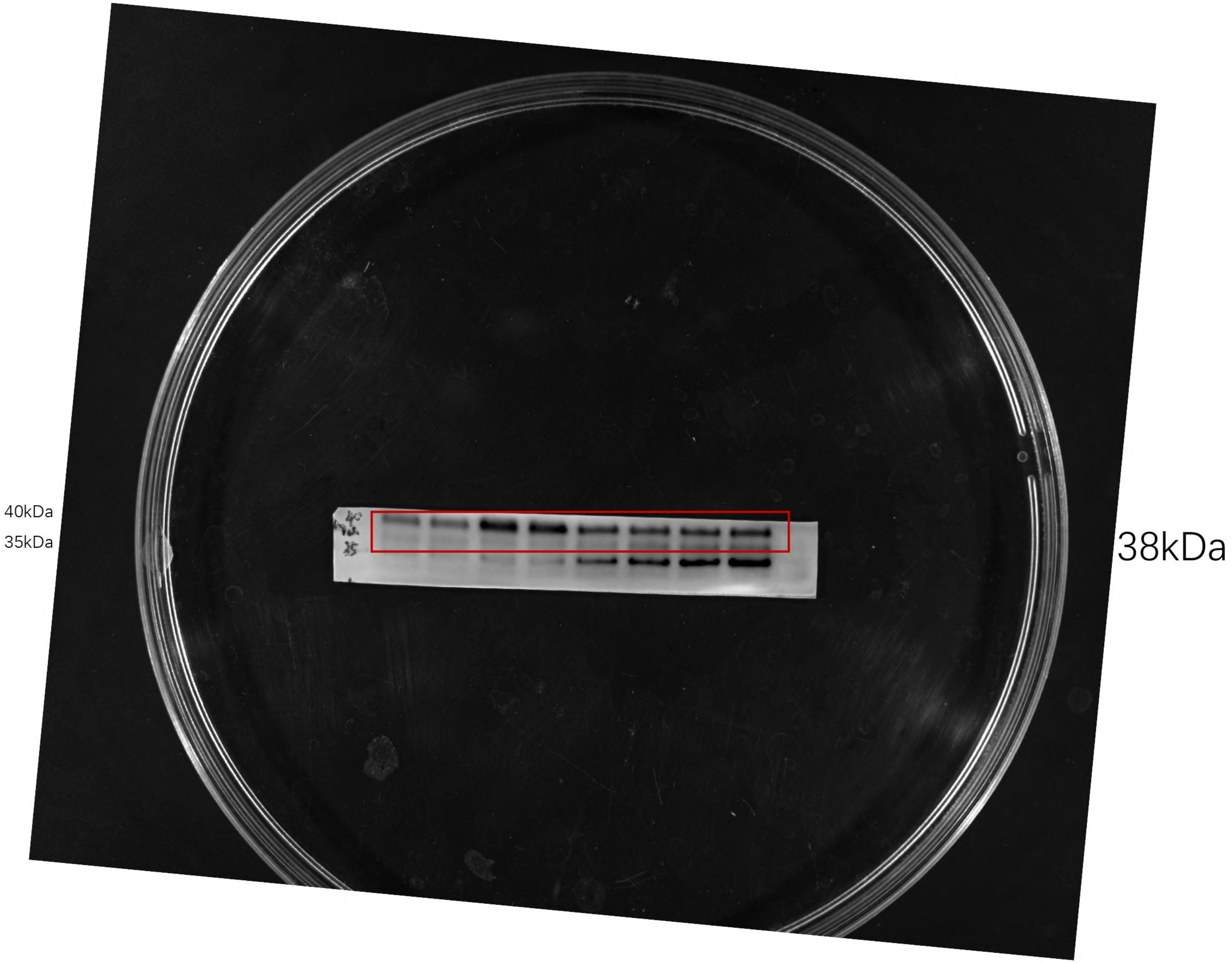

Source Fig.4C GAPDH

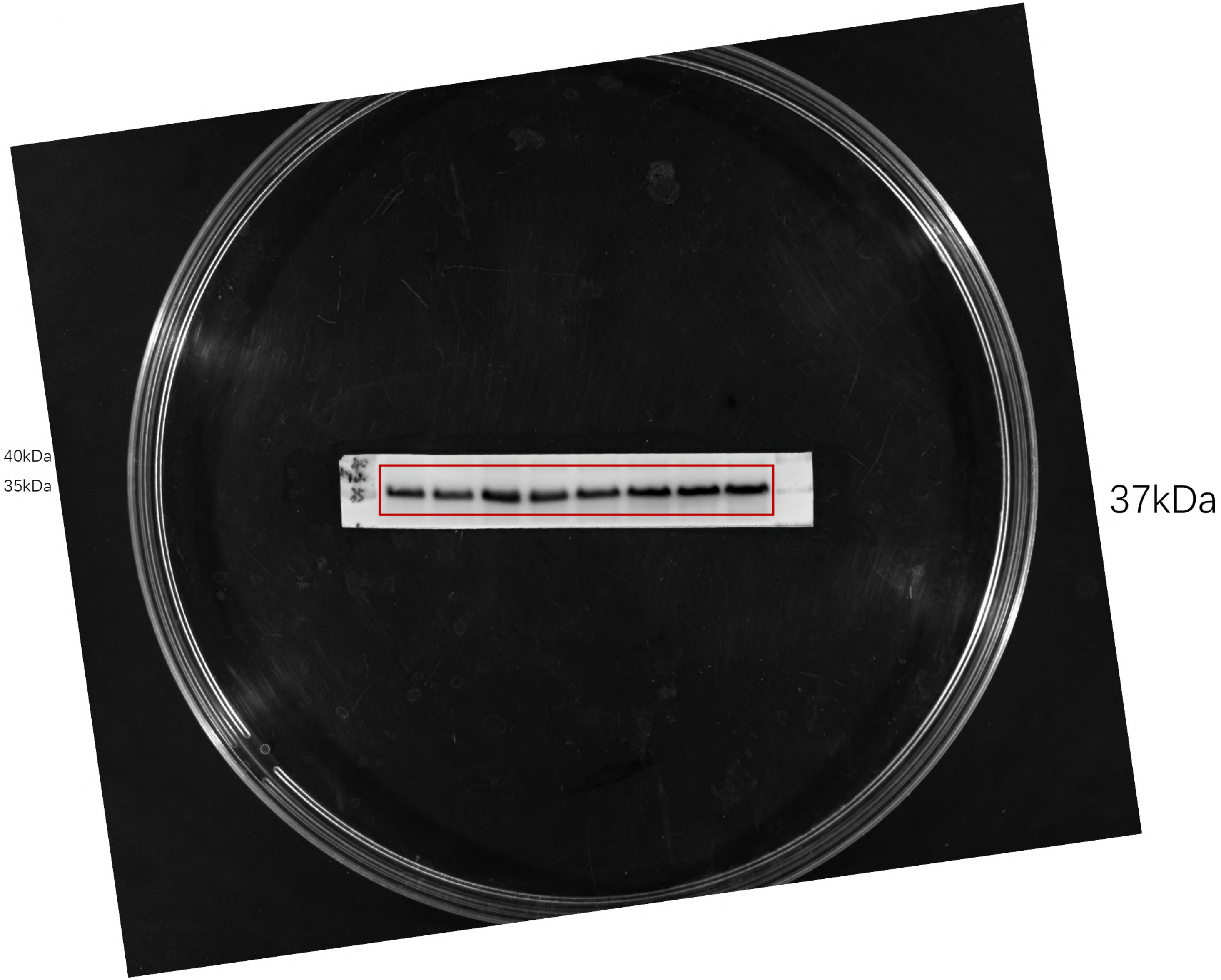

Source Fig.4C p38

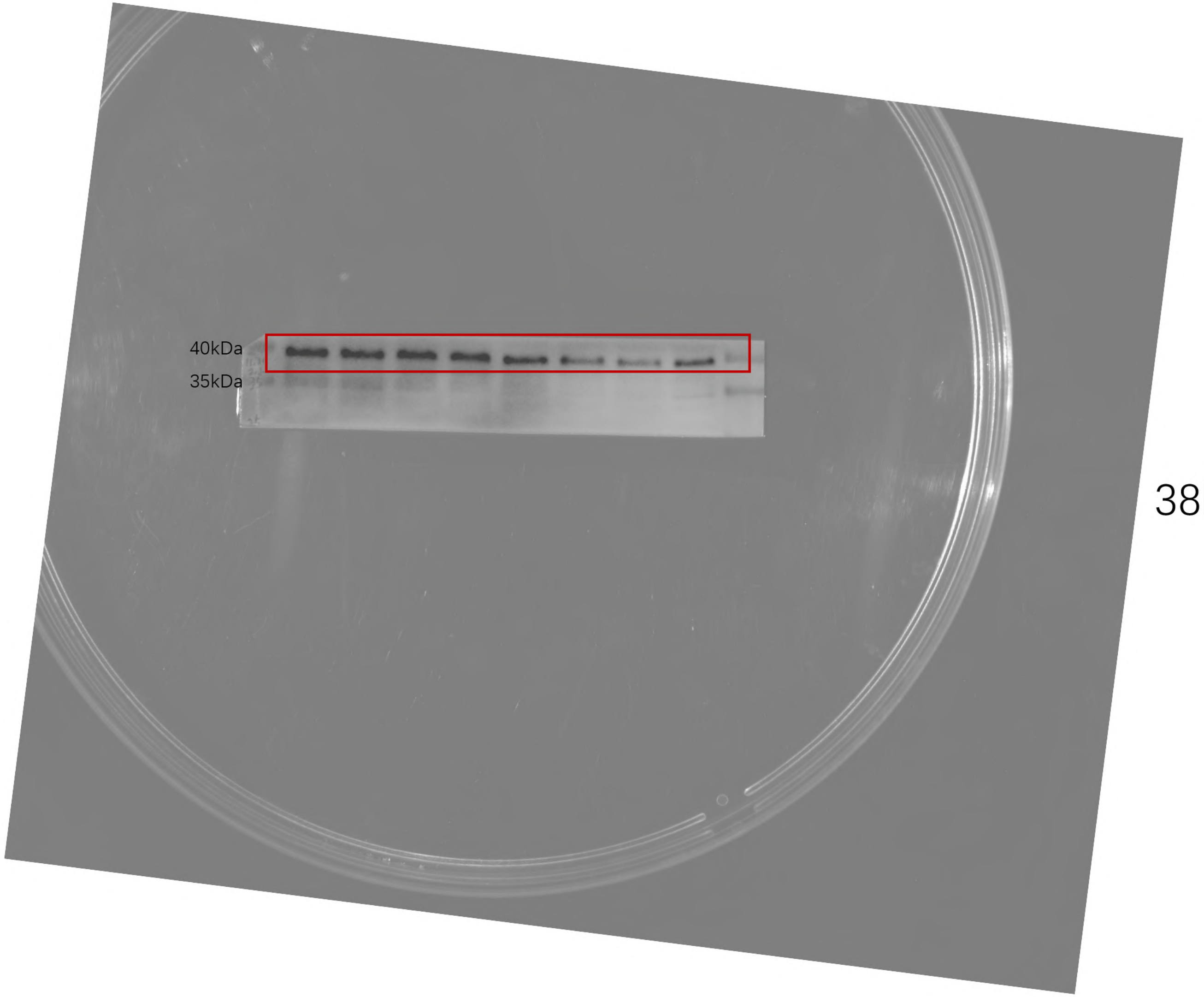

38kDa

Source Fig.4C GAPDH

40kDa
35kDa

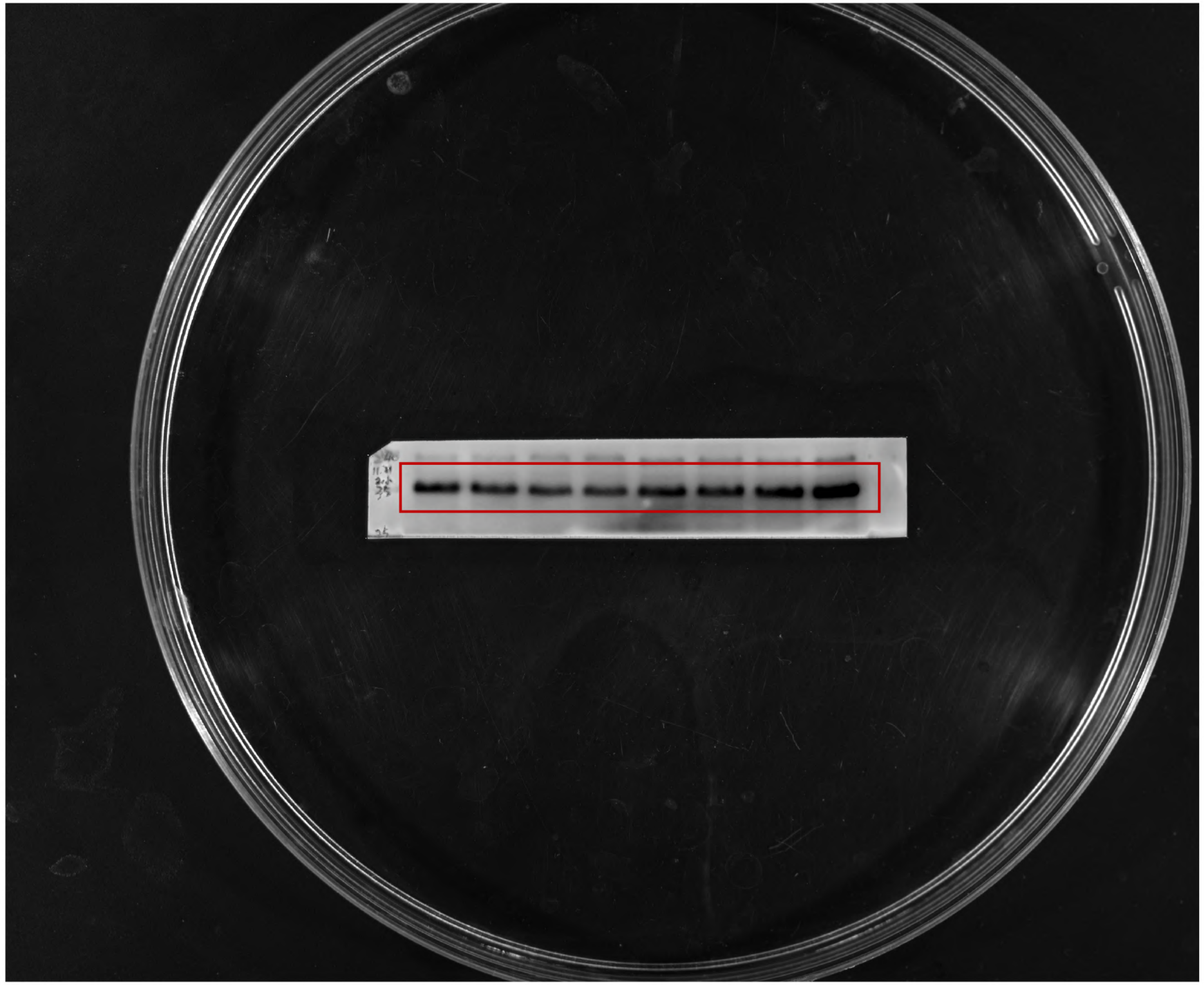

37kDa

Source Fig.4C p-TRAF2

50kDa

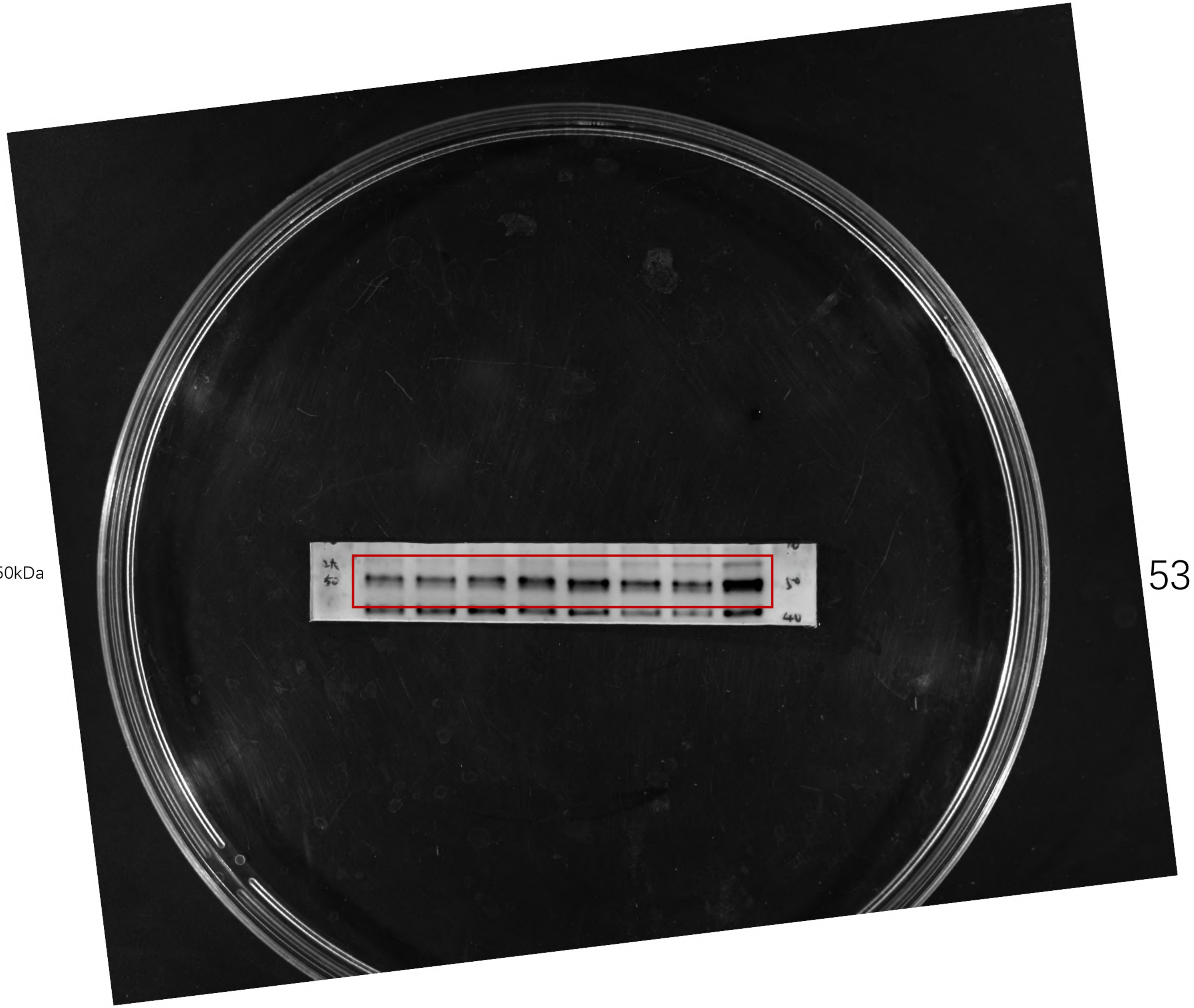

53kDa

Source Fig.4C TRAF2

50kDa

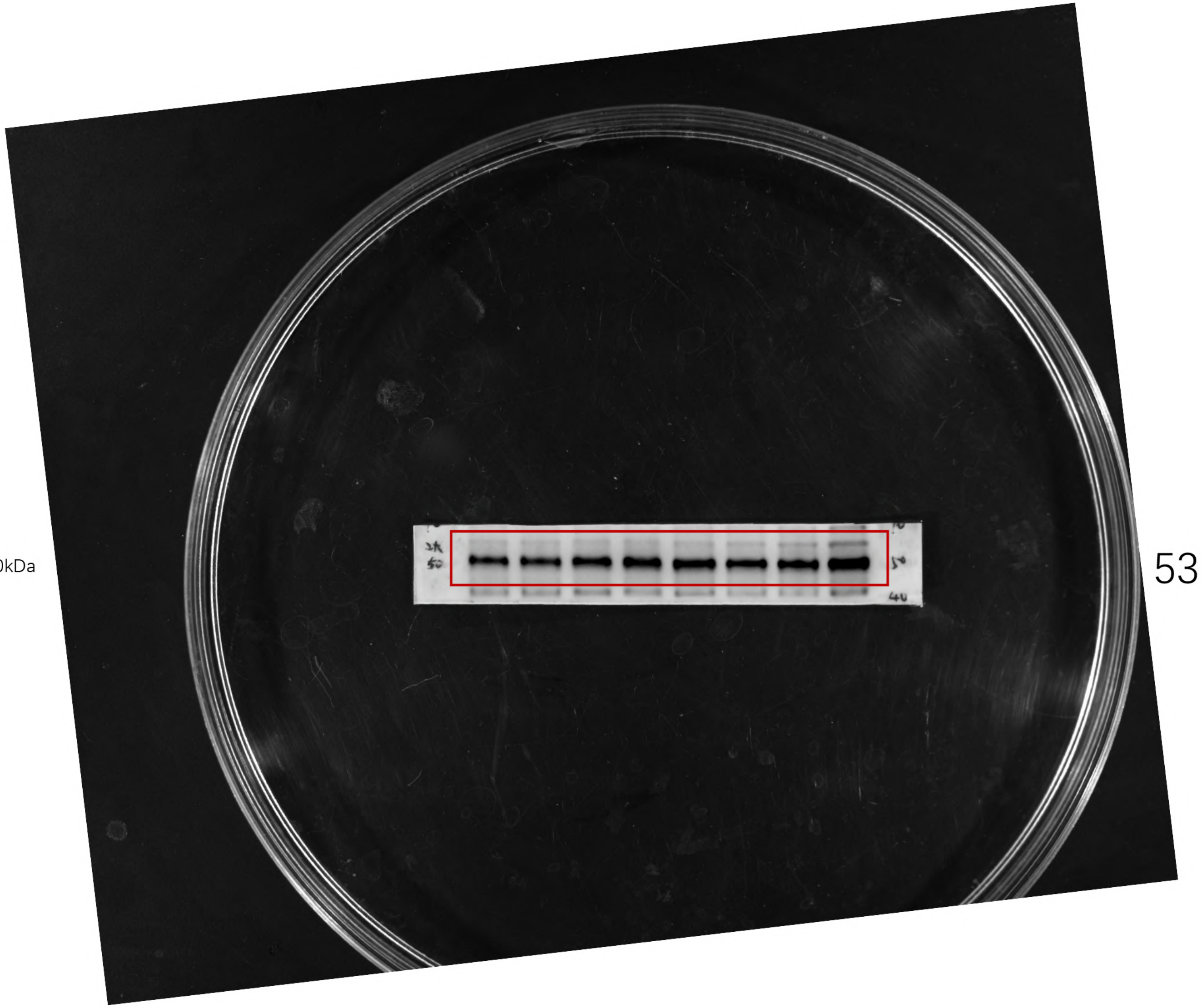

53kDa

Source Fig.4C Fn14

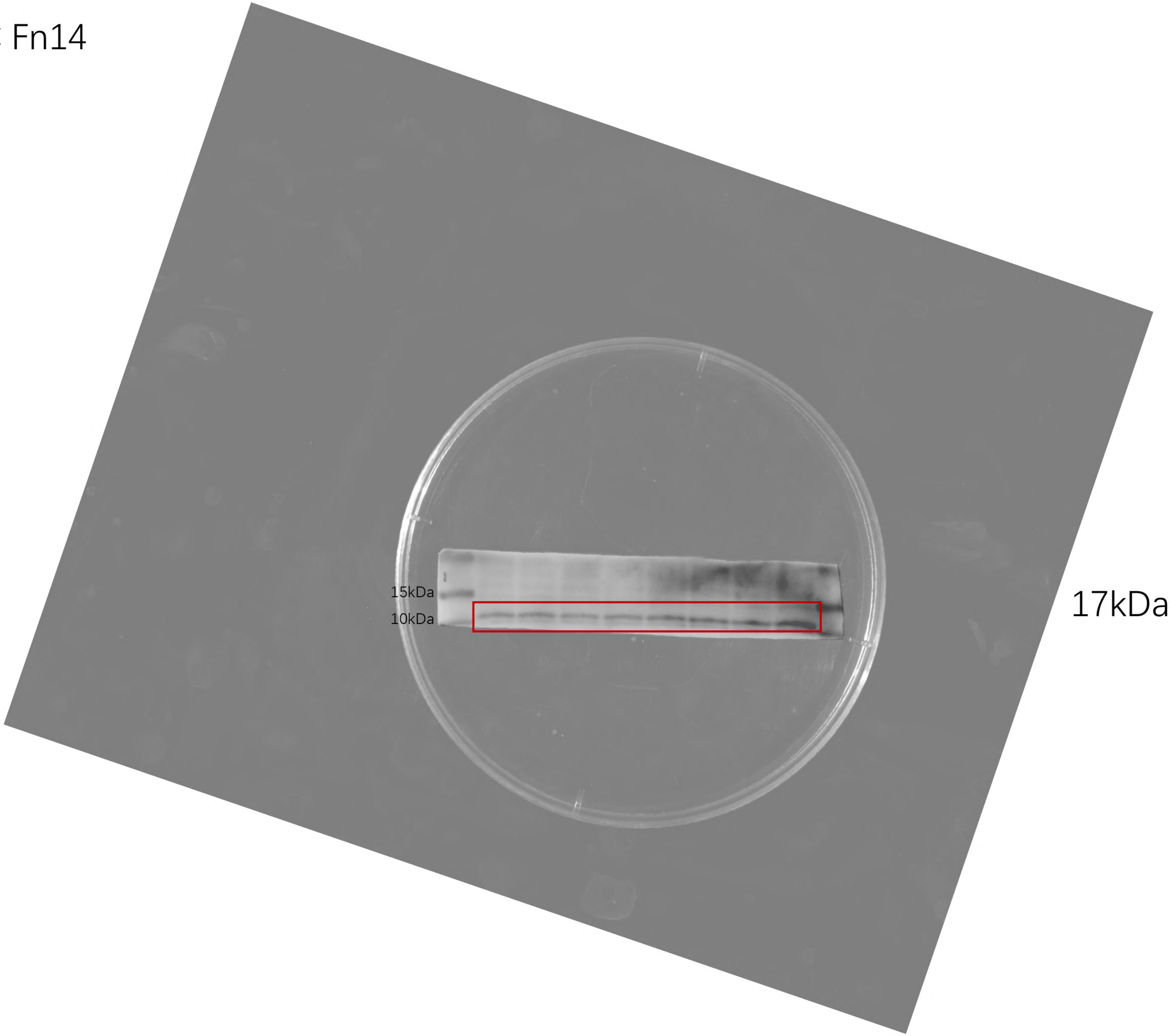

17kDa

Source Fig.4C GAPDH

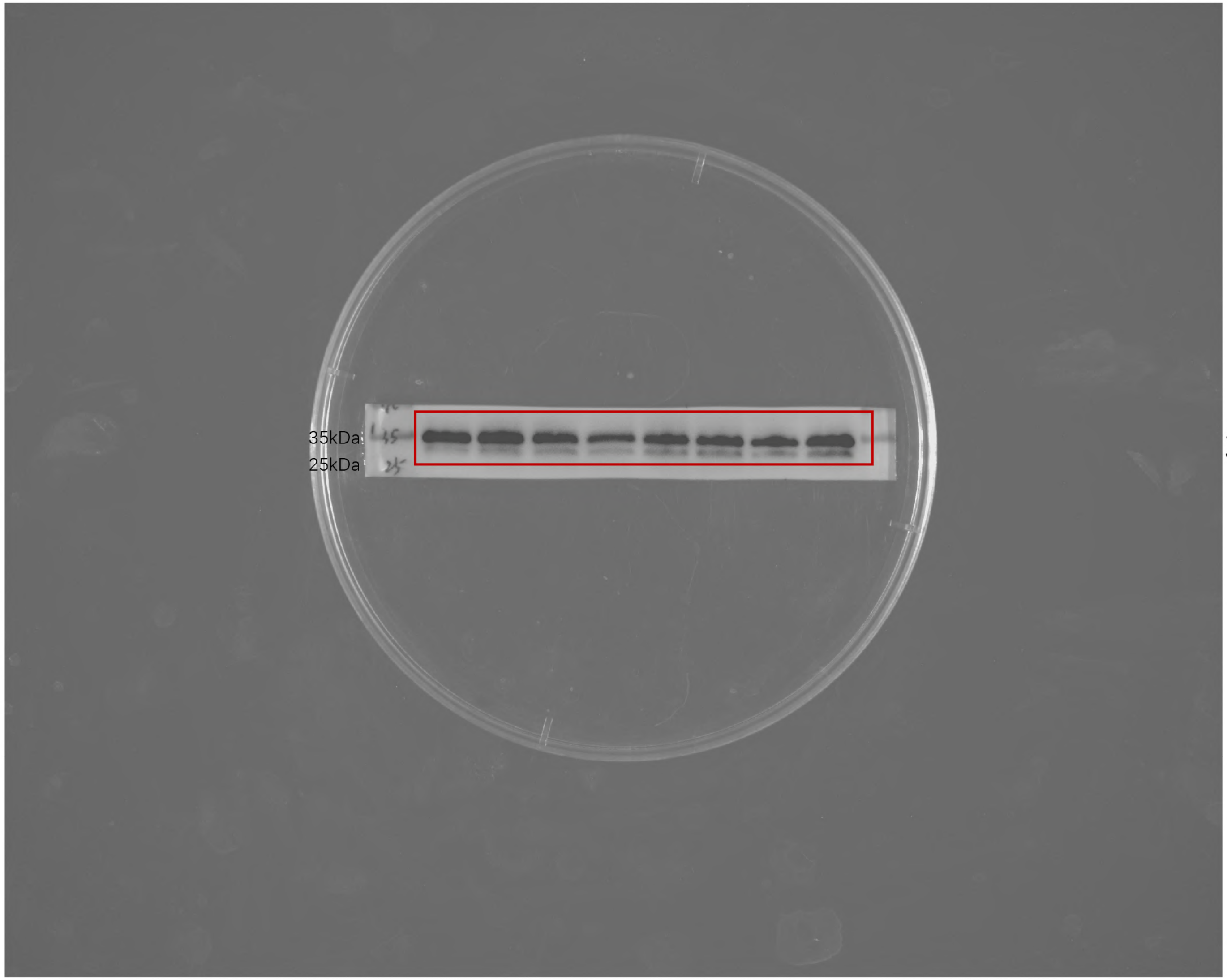

37kDa

Source Fig.5G LCN2

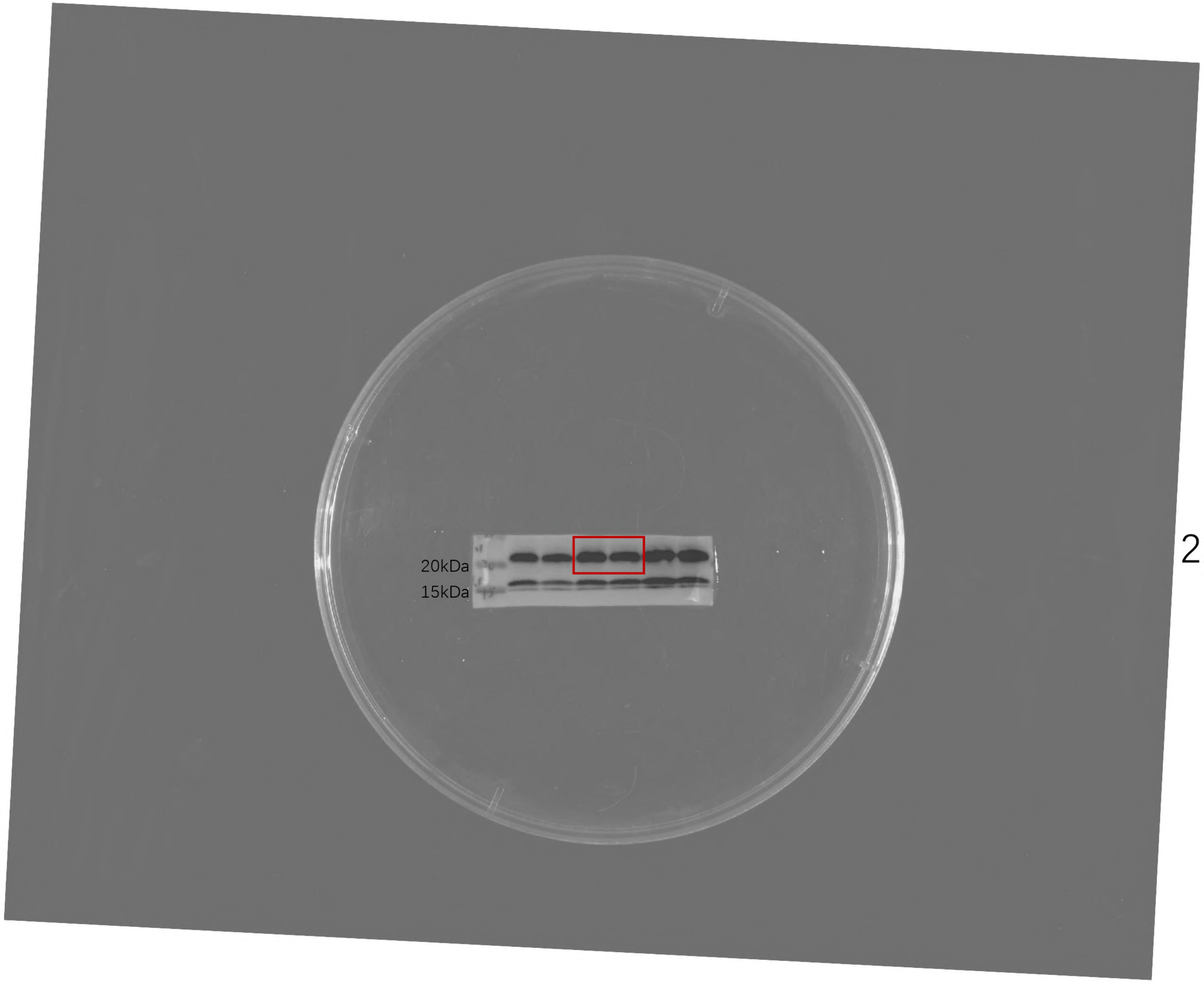

21kDa

Source Fig.5G GAPDH

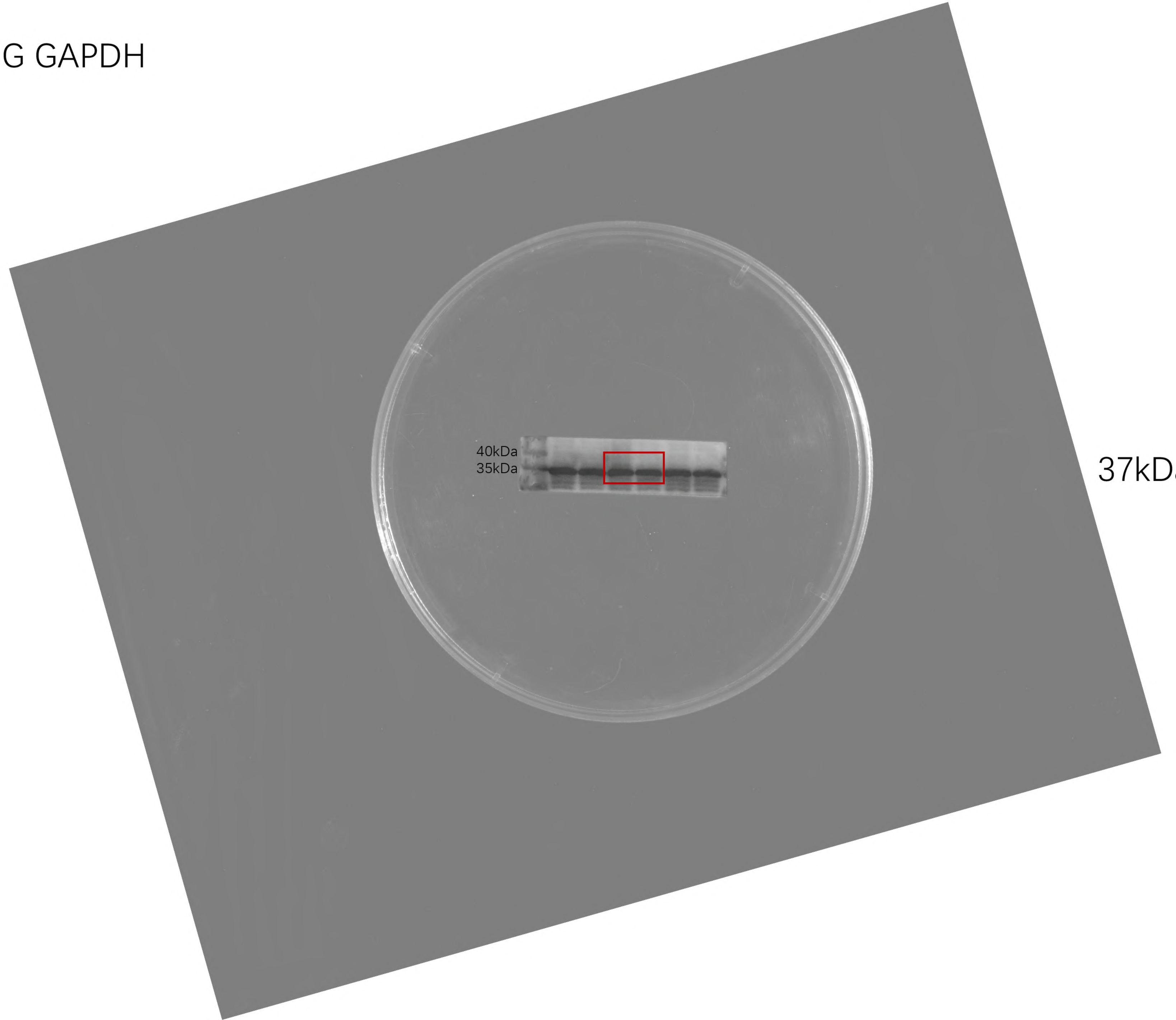

37kDa

Source Fig.5G LCN2

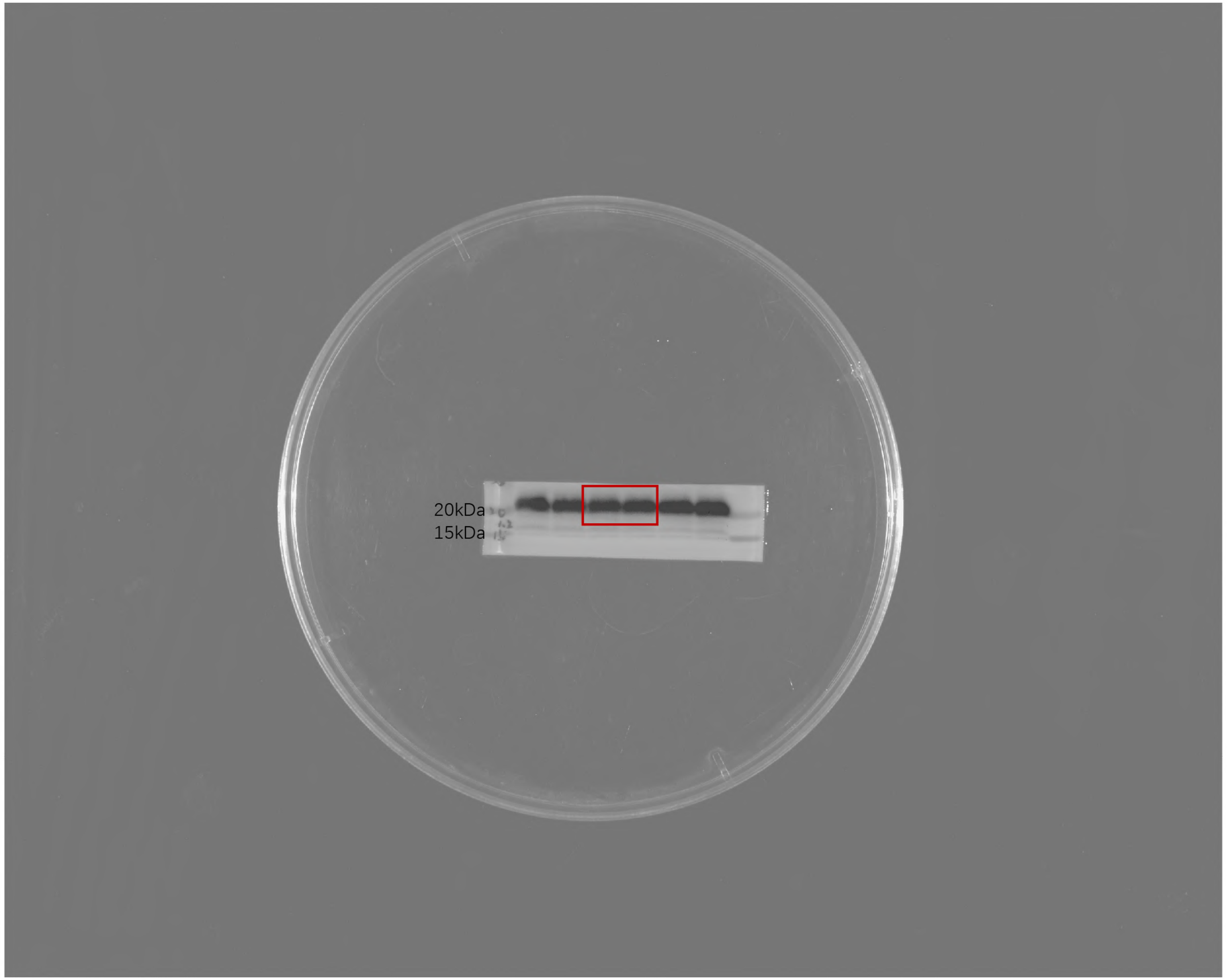

21kDa

Source Fig.5G GAPDH

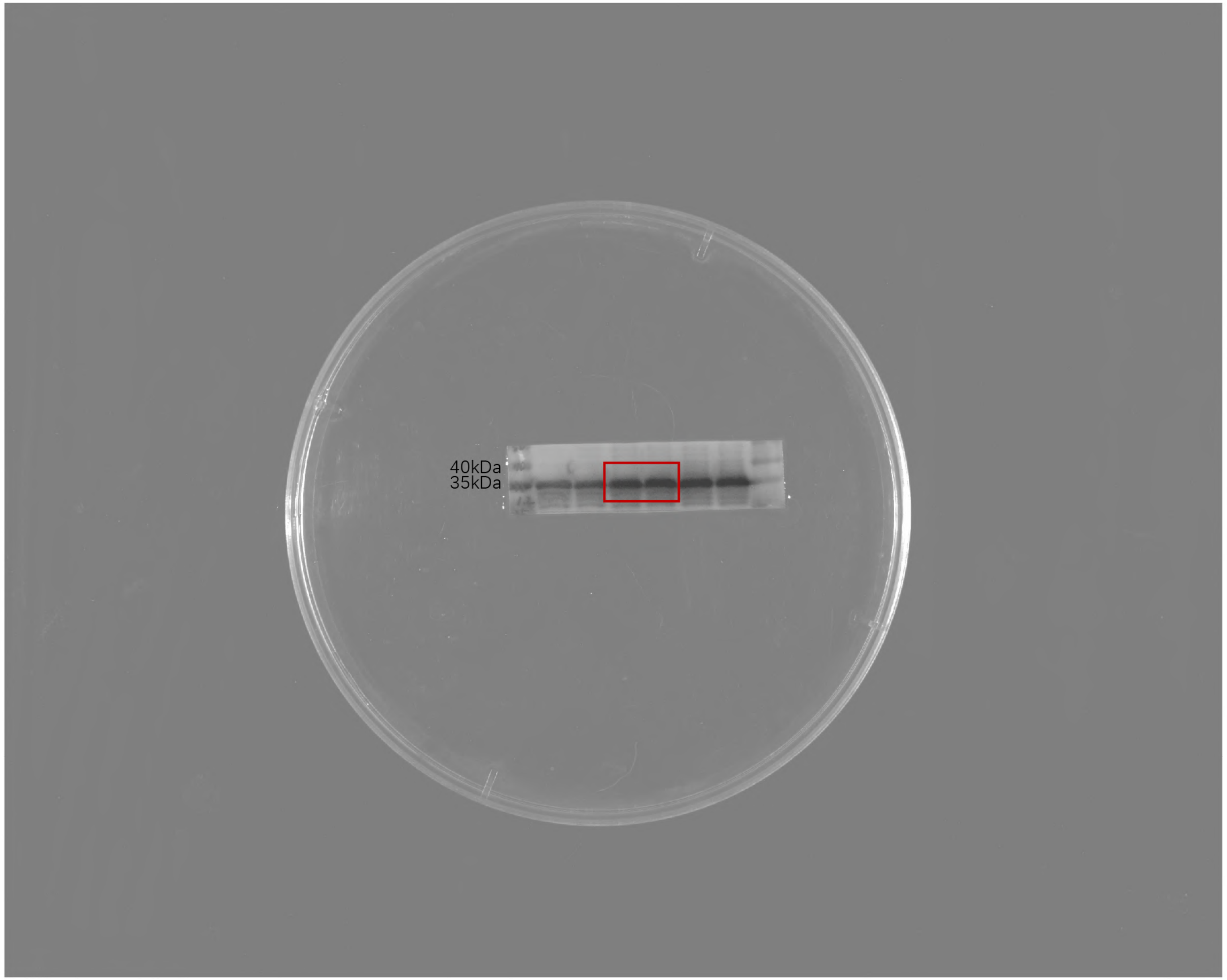

37kDa

Source Fig.5G 24P3R

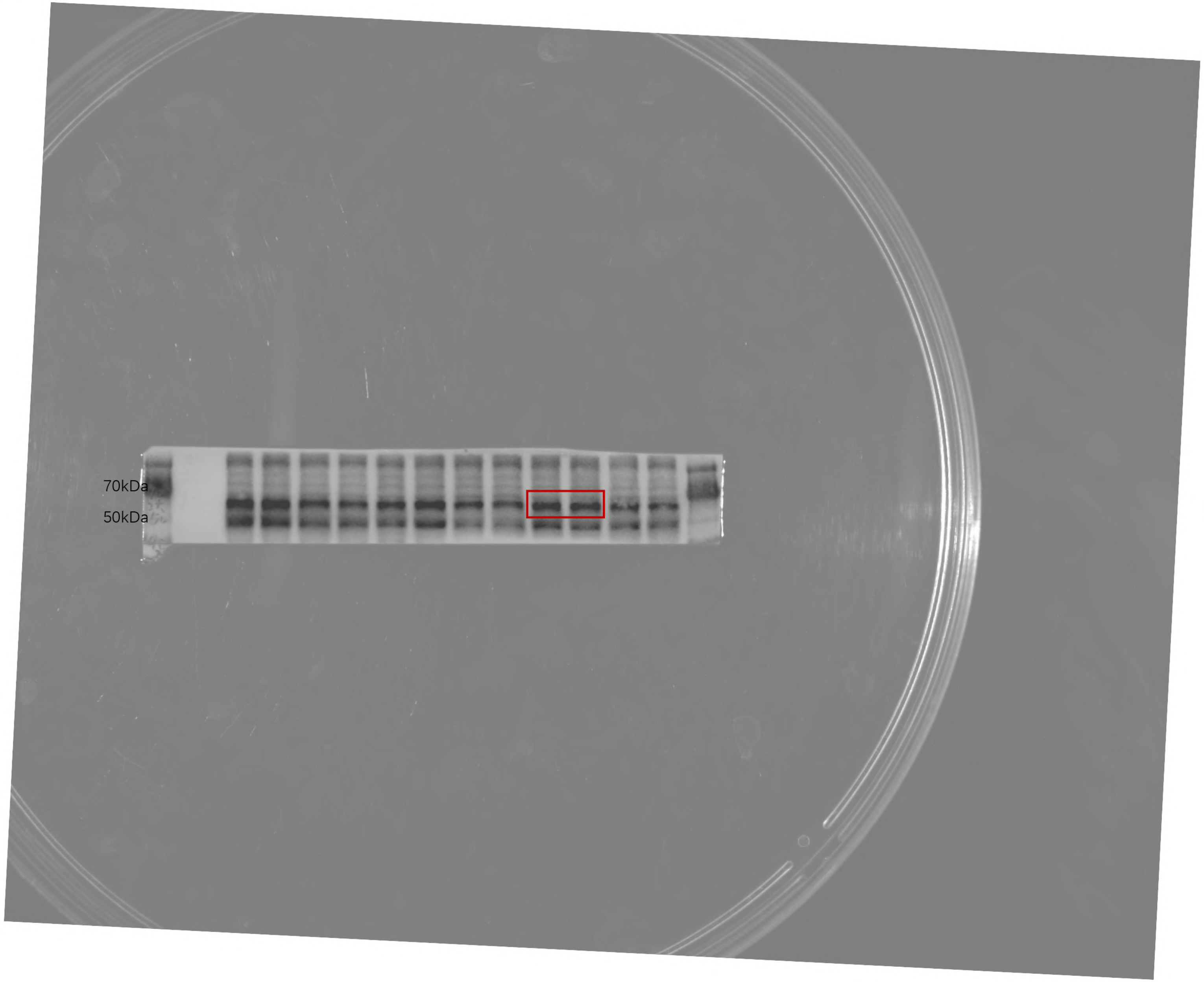

66kDa

Source Fig.5G GAPDH

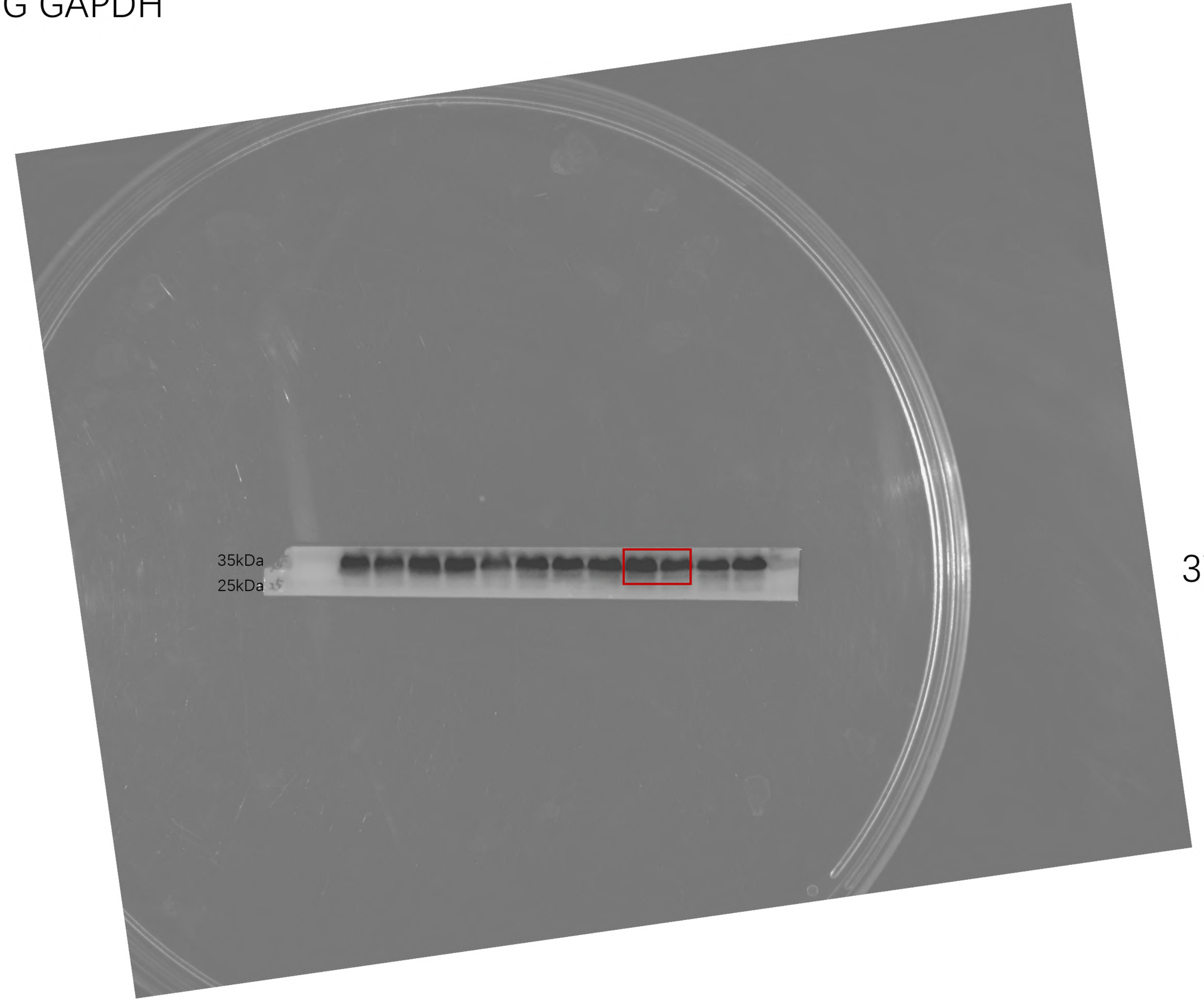

37kDa

Source Fig.5G 24P3R

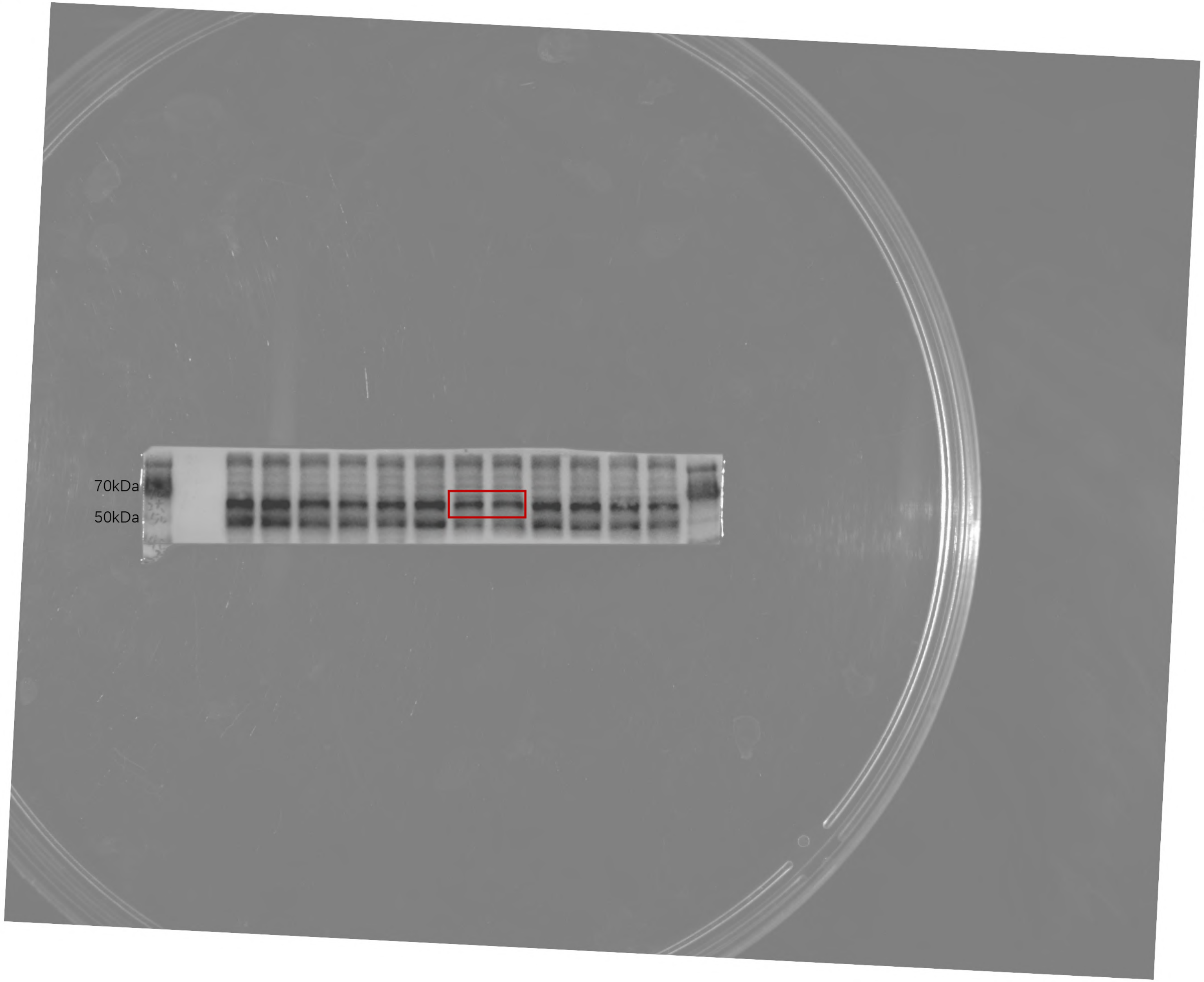

66kDa

Source Fig.5G GAPDH

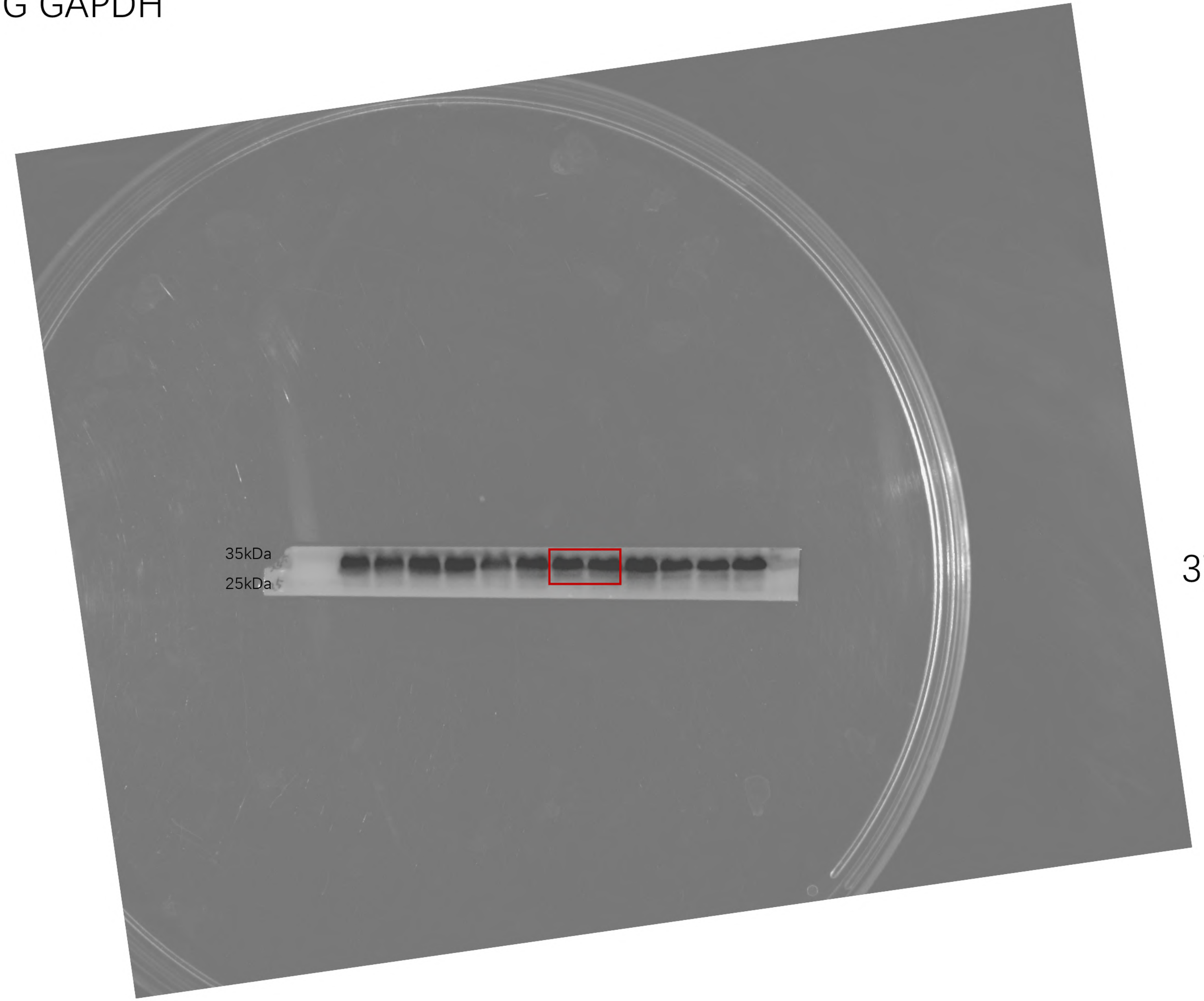

37kDa

Source Fig.5H MMP9

100kDa
70kDa

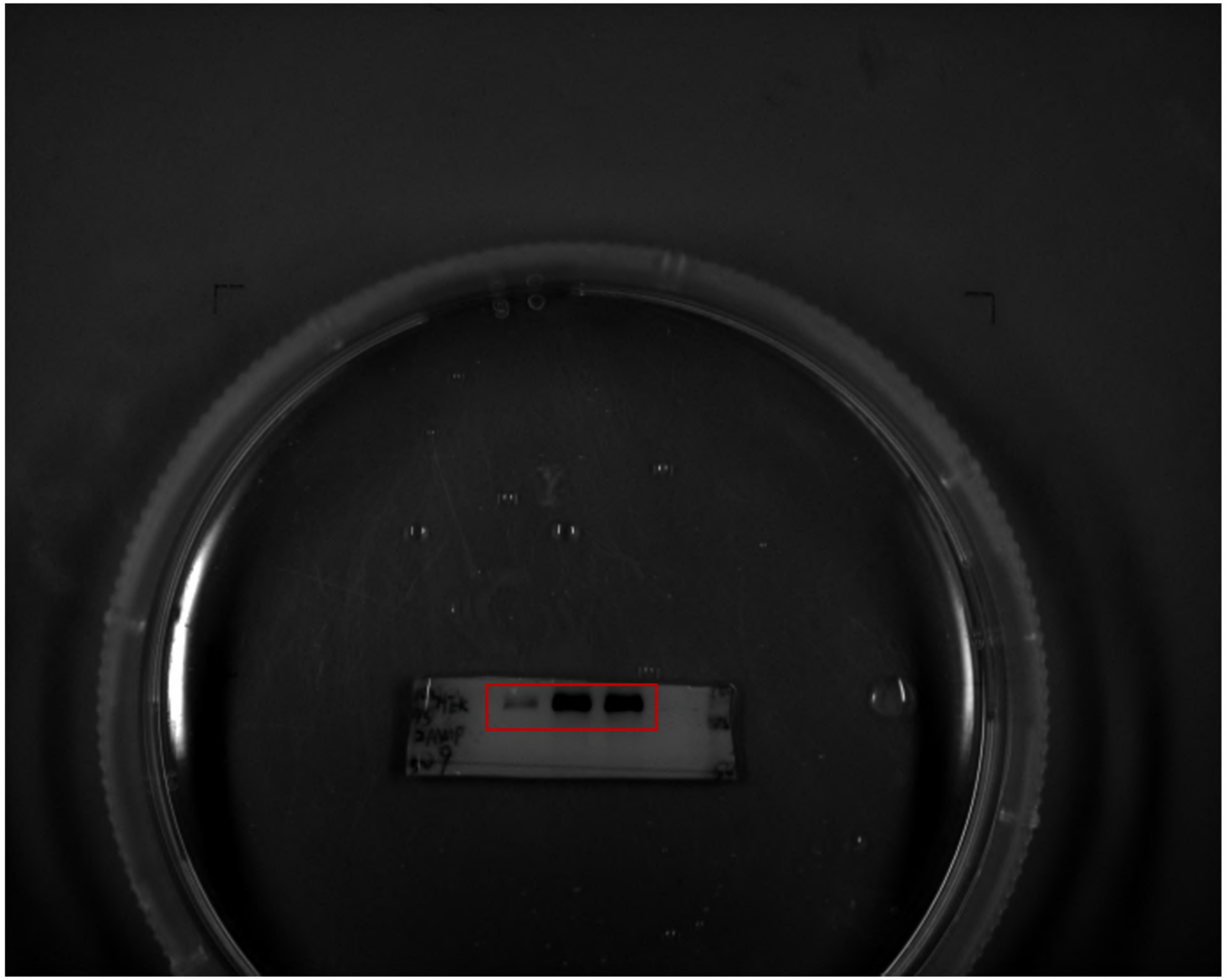

92kDa
84kDa

Source Fig.5H B-Actin

37kDa

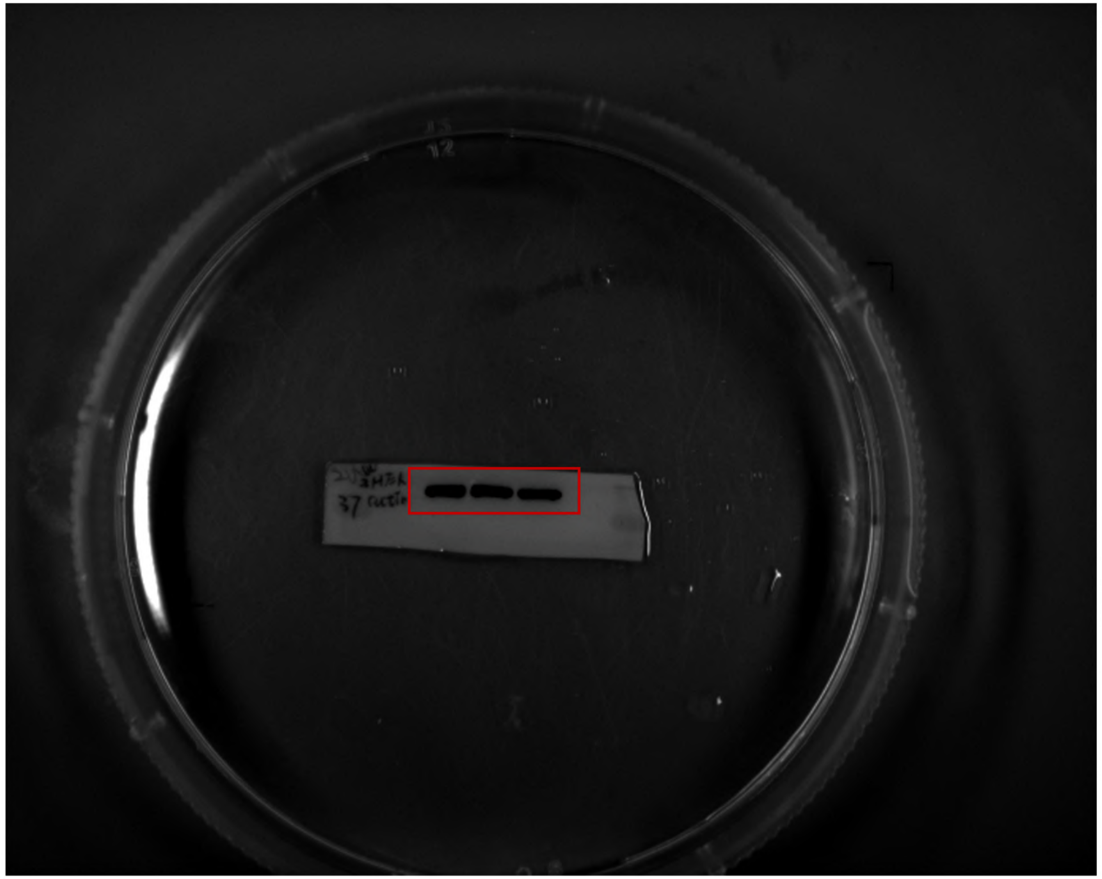

45kDa

Source Fig.5H TRAF2

70kDa
50kDa

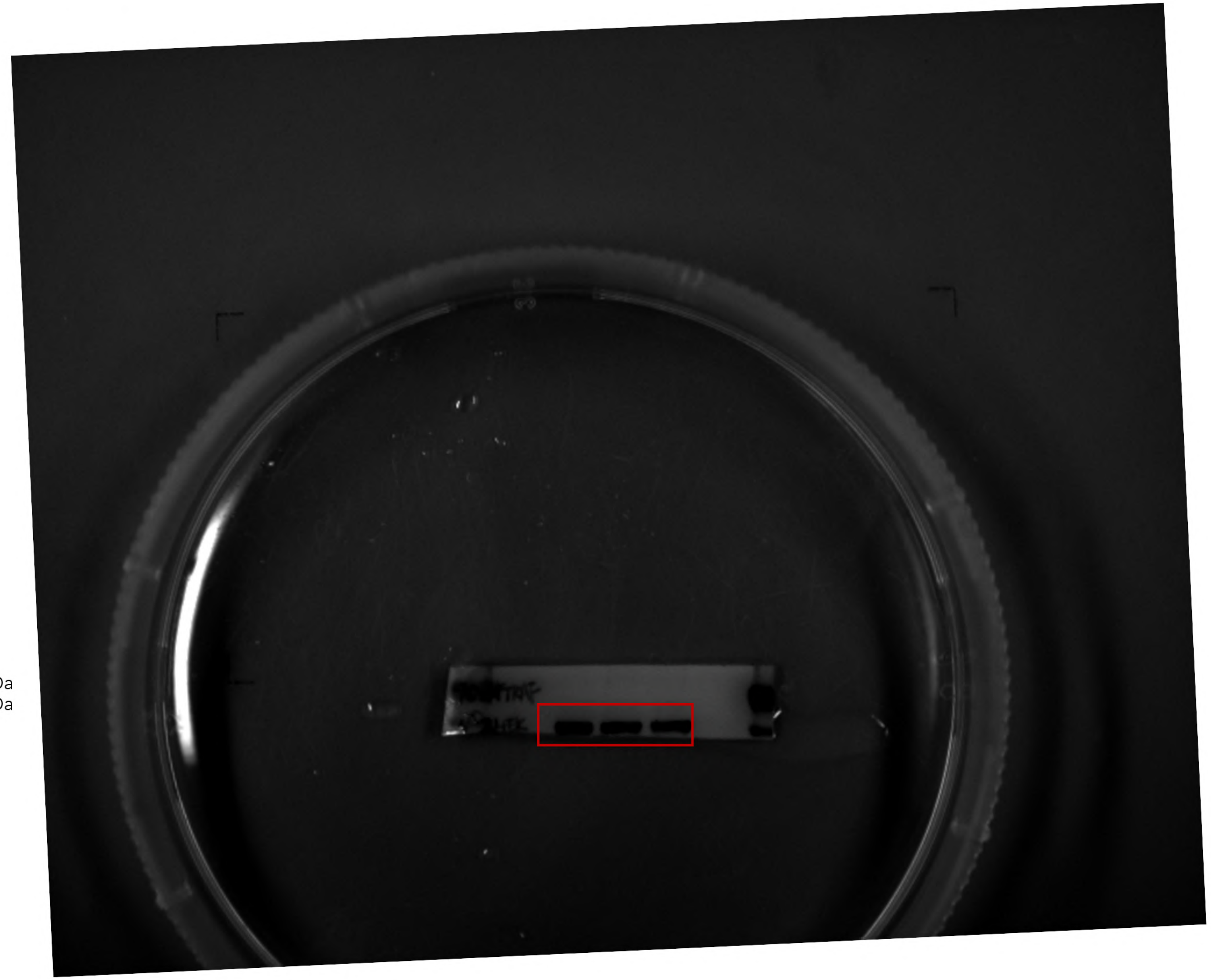

53kDa

Source Fig.5H B-Actin

40kDa

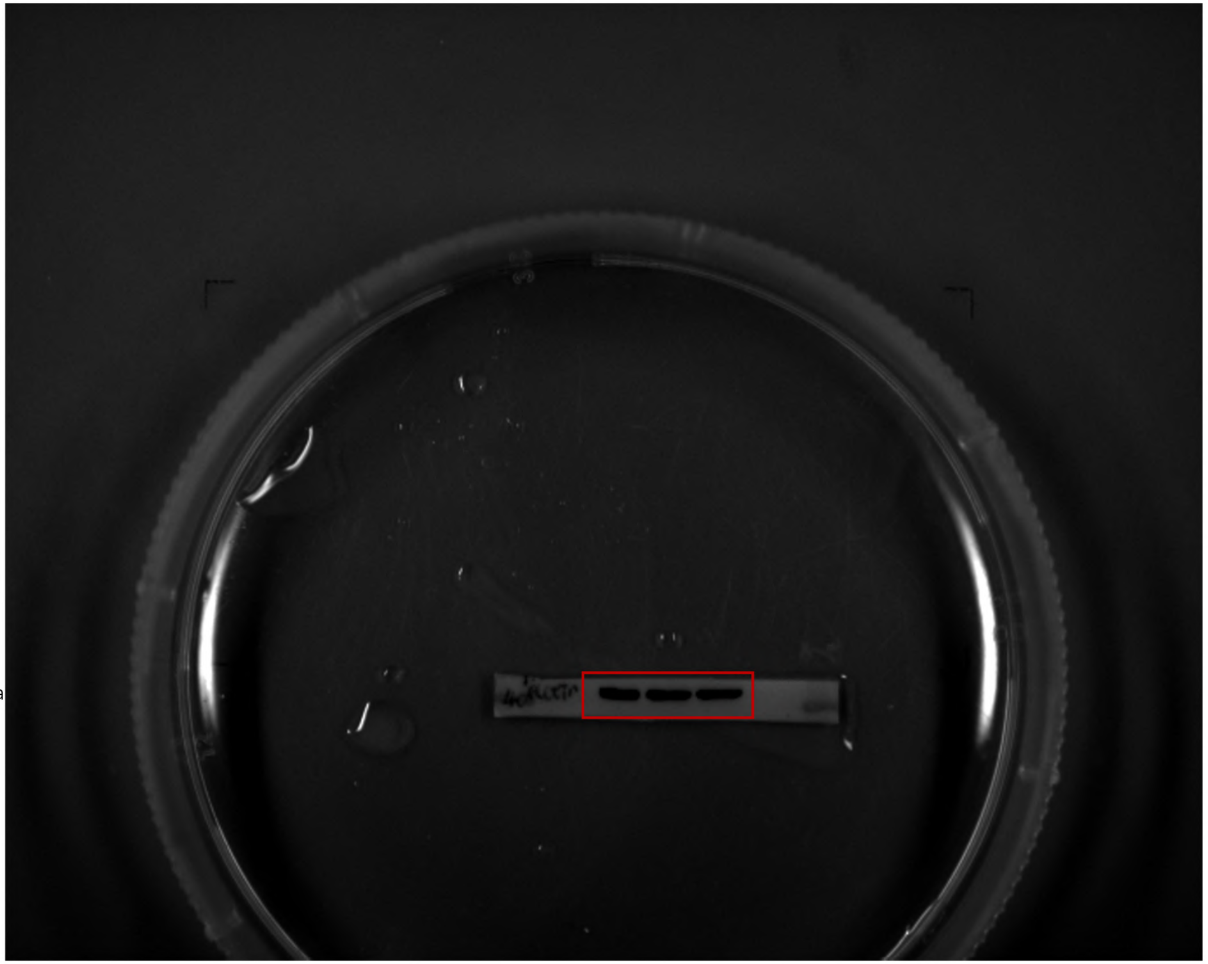

45kDa

Source Fig.6C p-ERK1/2

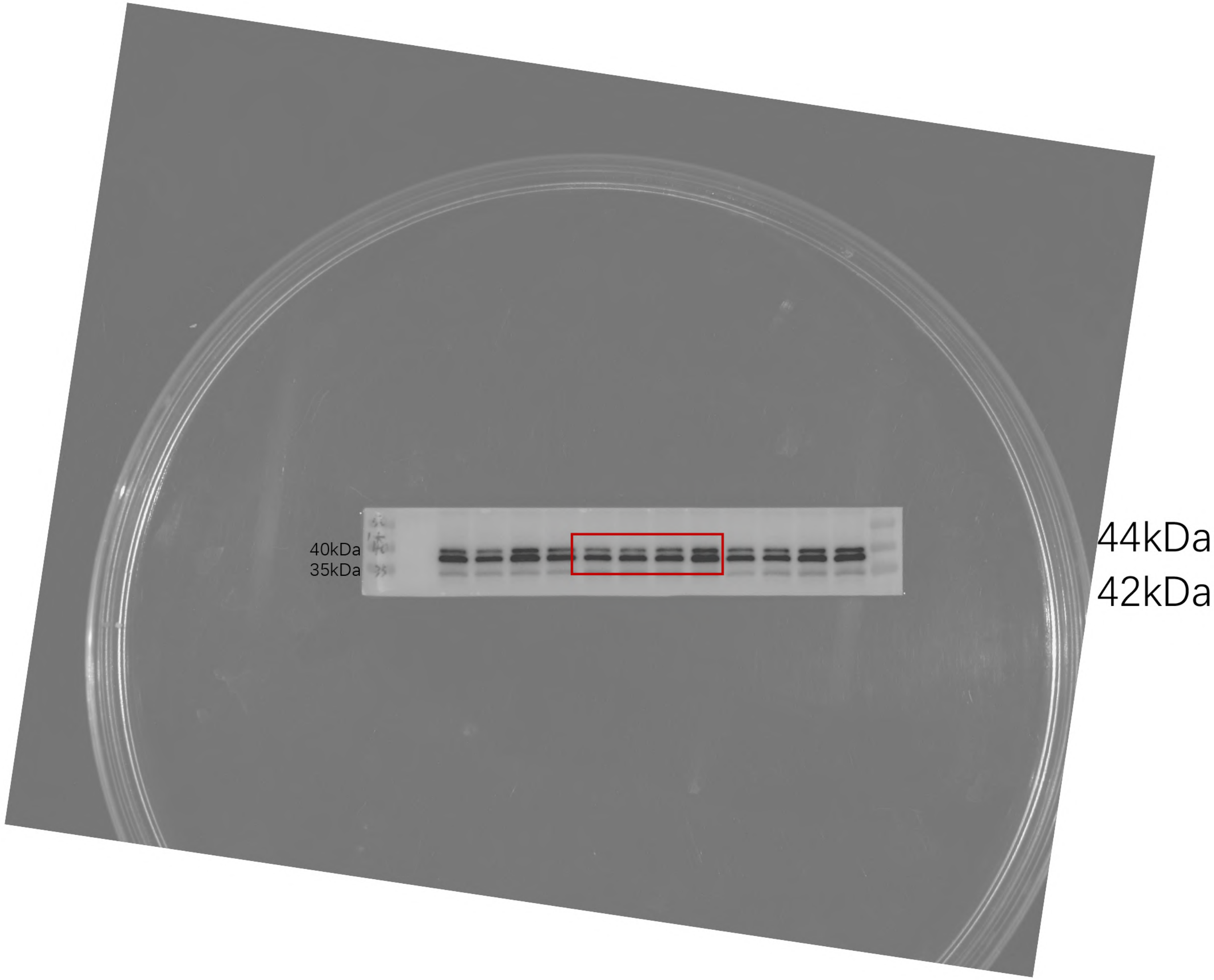

Source Fig.6C GAPDH

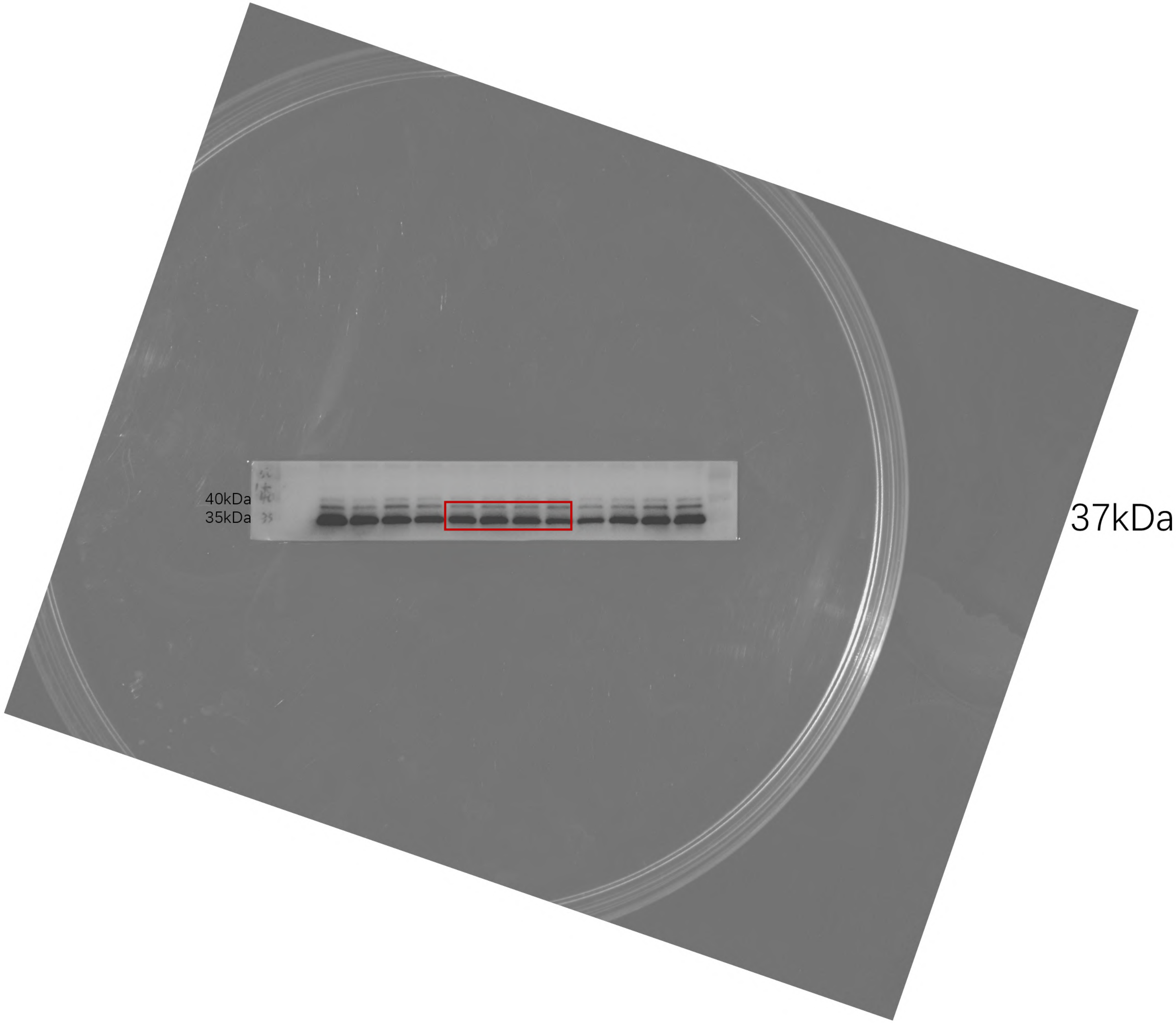

Source Fig.6C ERK1/2

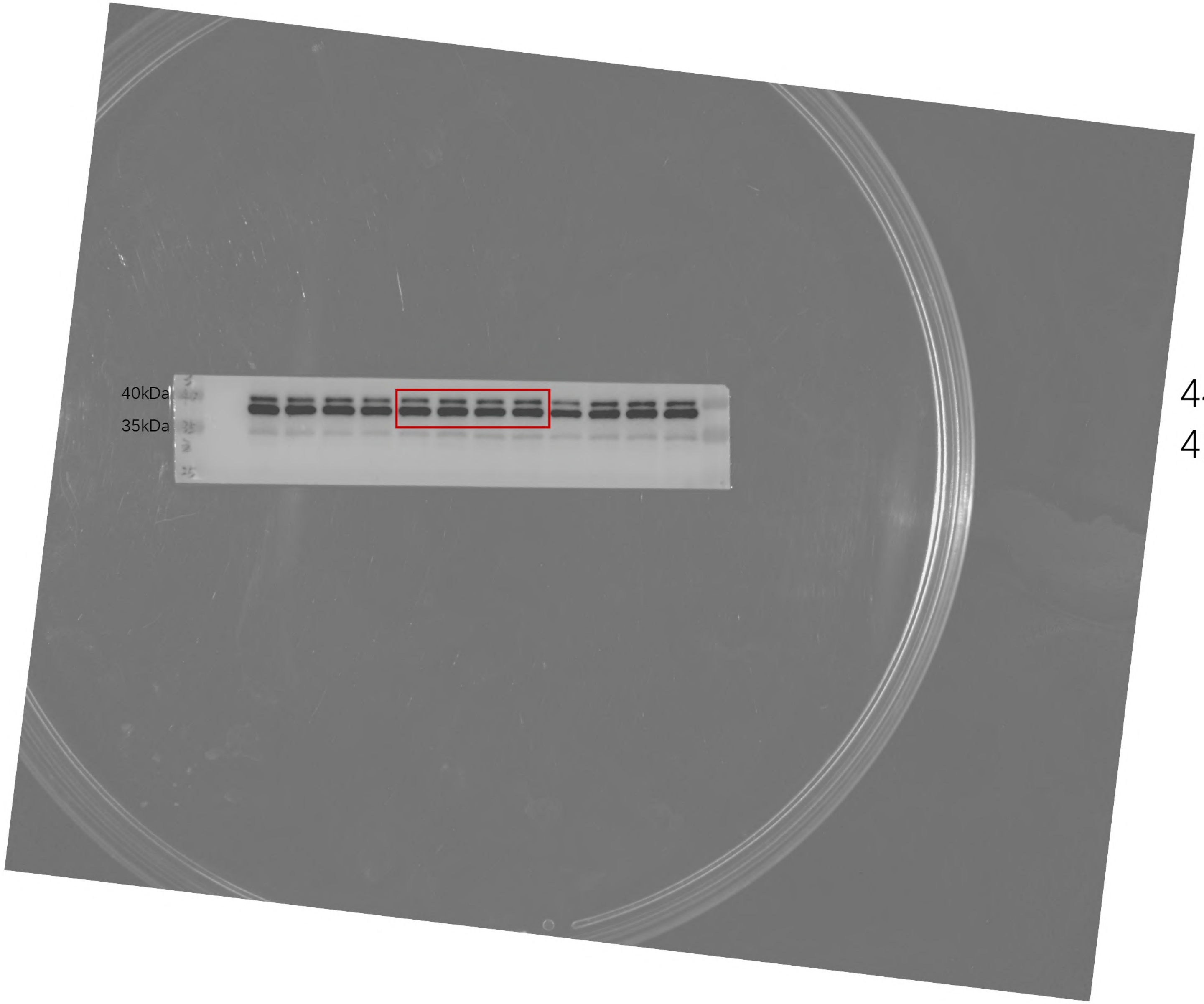

44kDa
42kDa

Source Fig.6C GAPDH

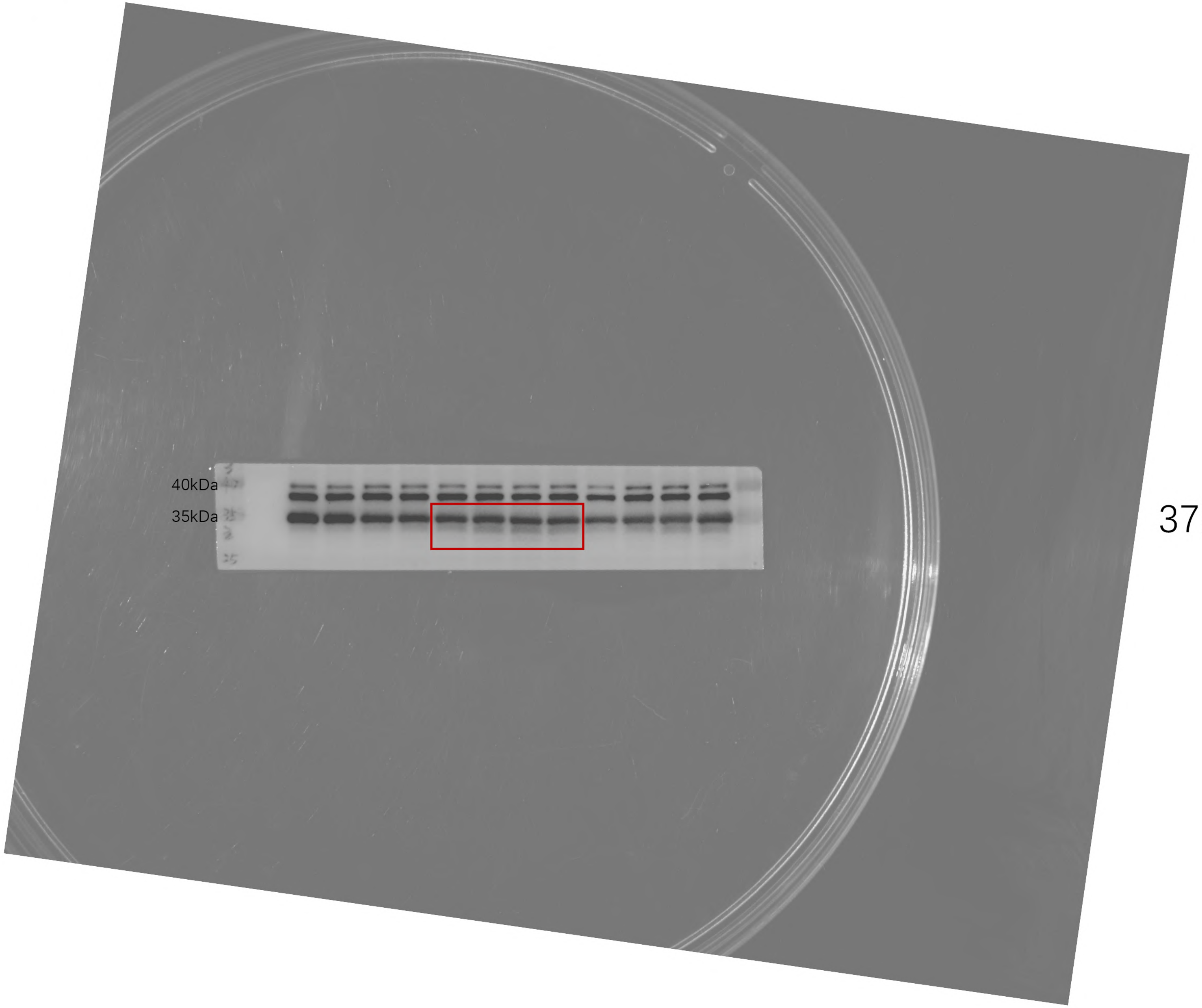

37kDa

Source Fig.6C p-JNK

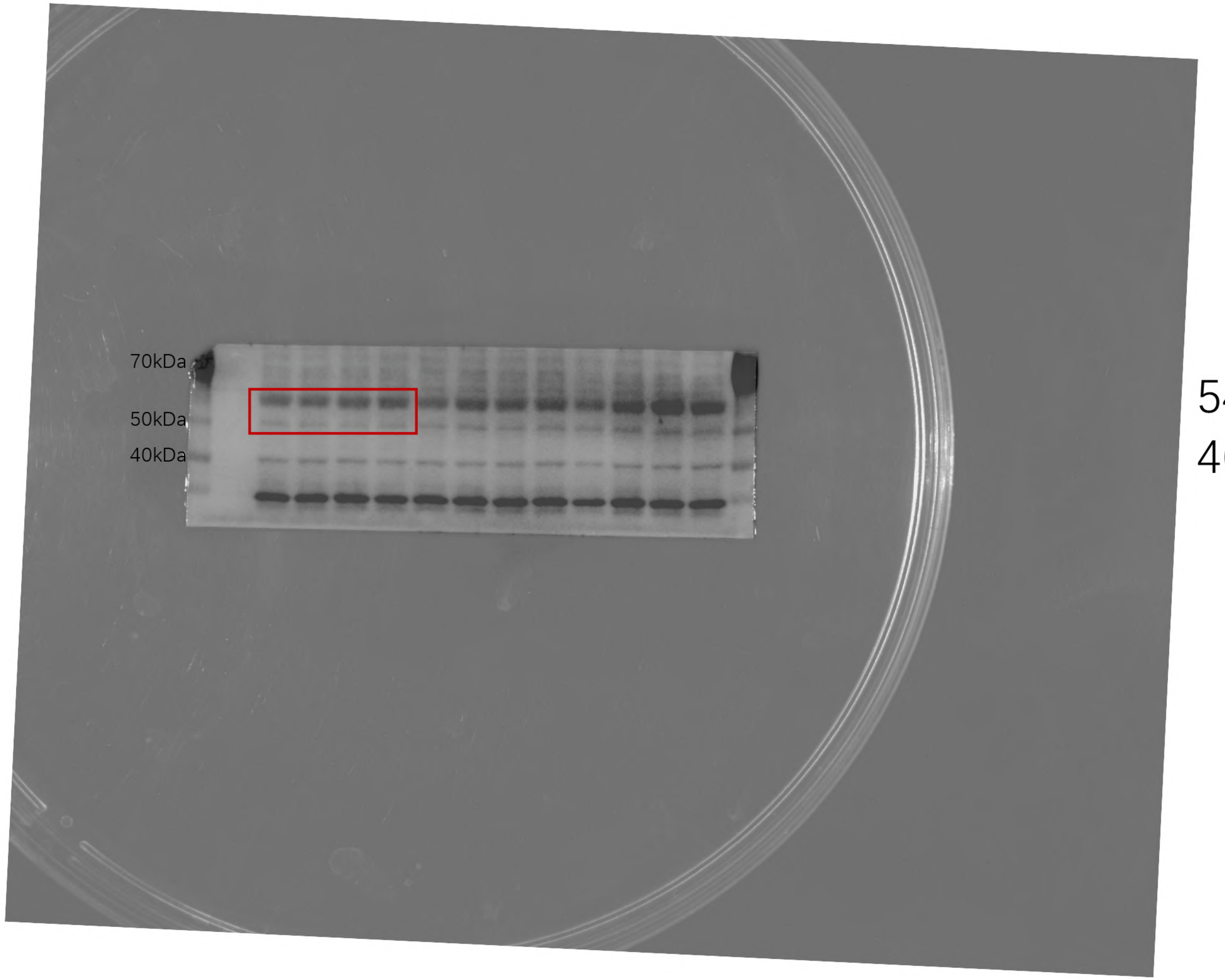

54kDa
46kDa

Source Fig.6C GAPDH

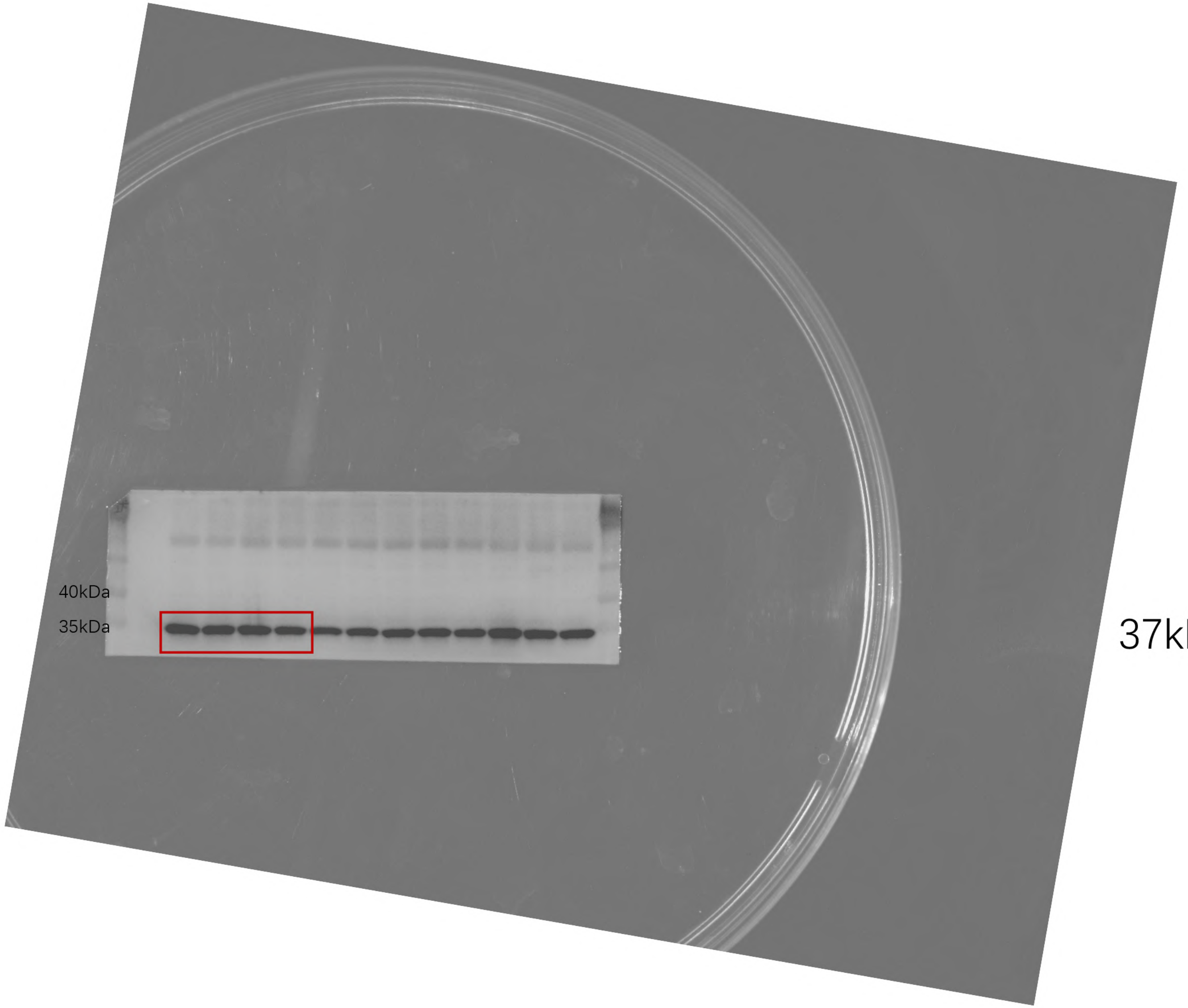

37kDa

Source Fig.6C JNK

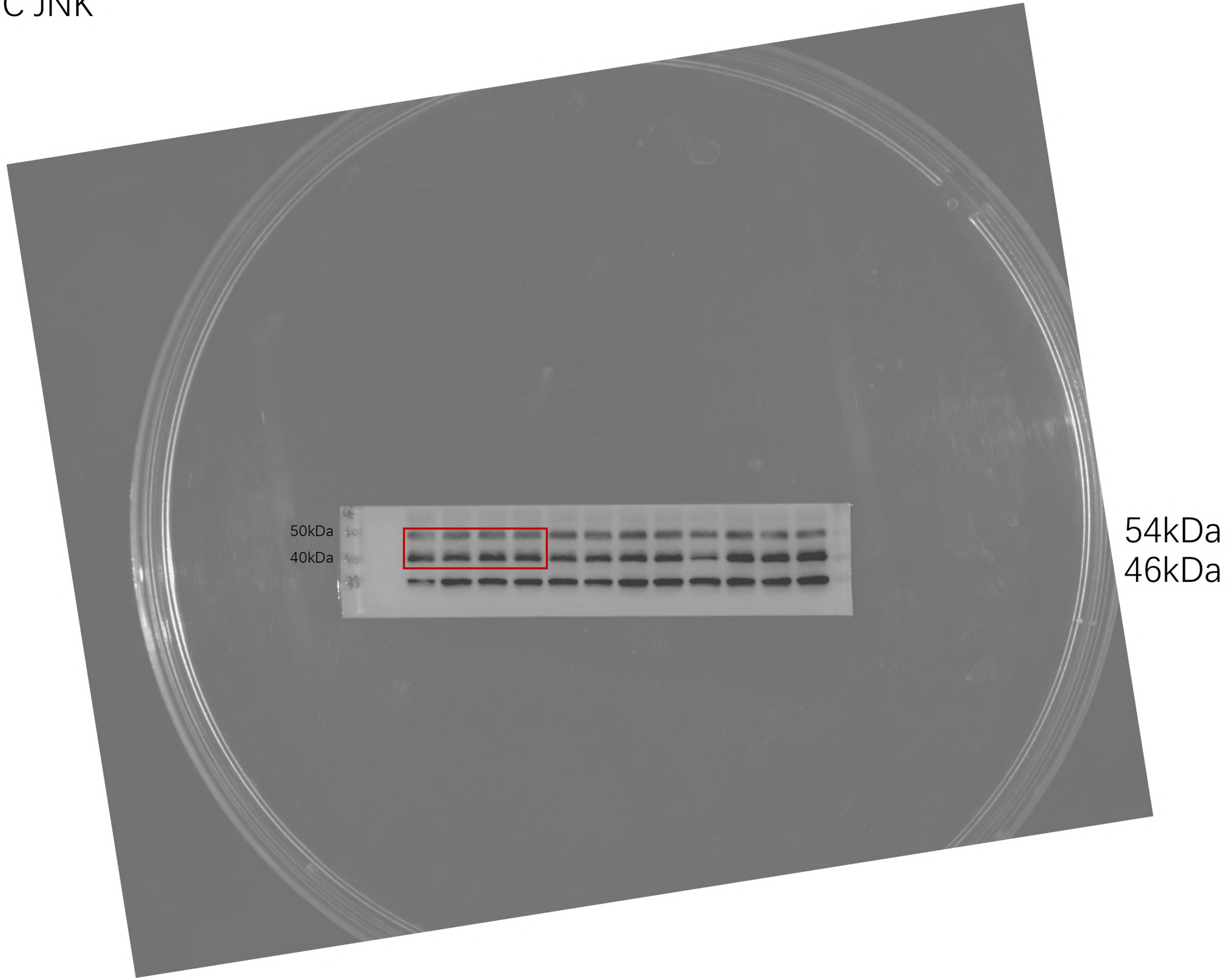

Source Fig.6C GAPDH

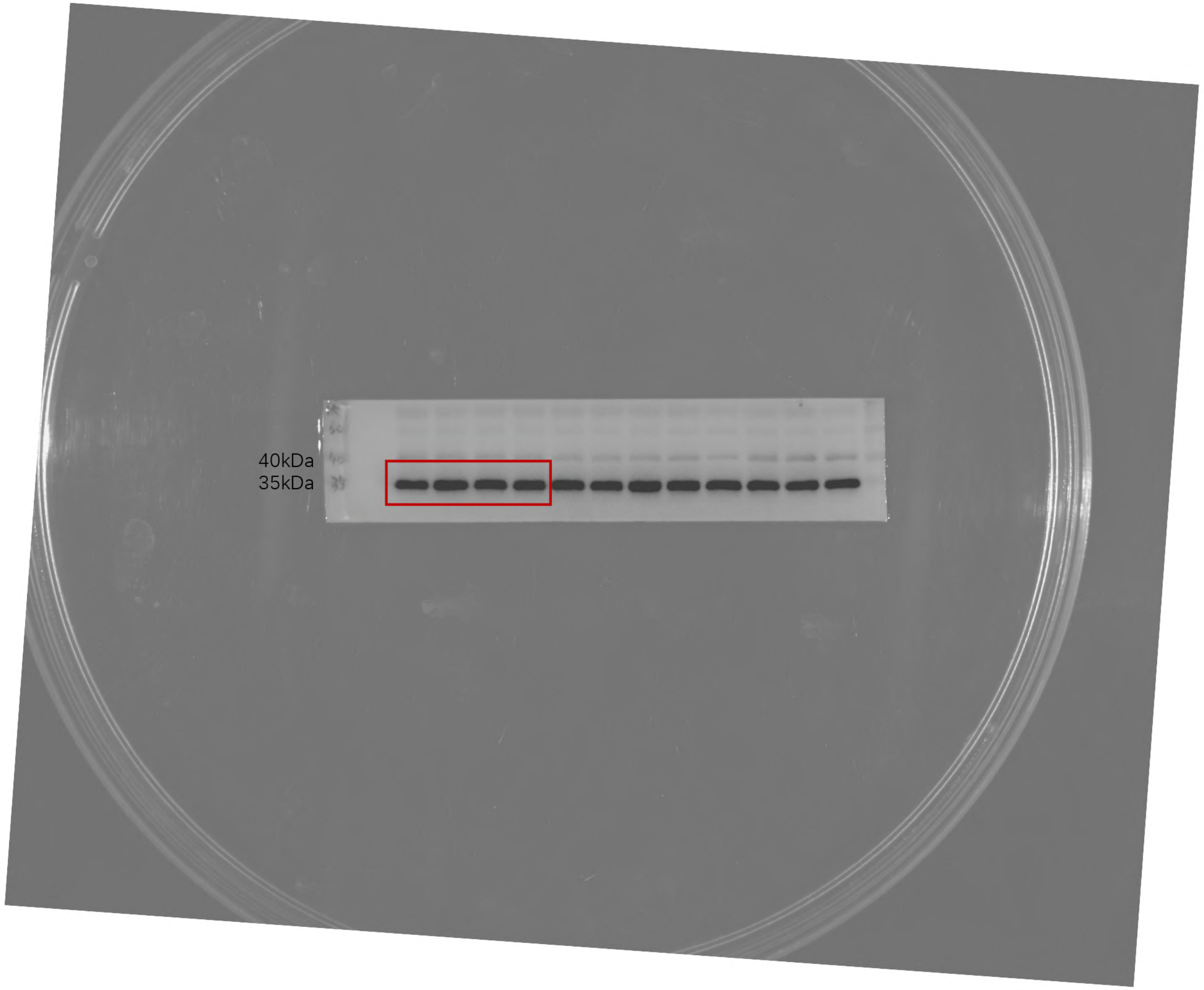

92kDa
84kDa

Source Fig.6C p-p38

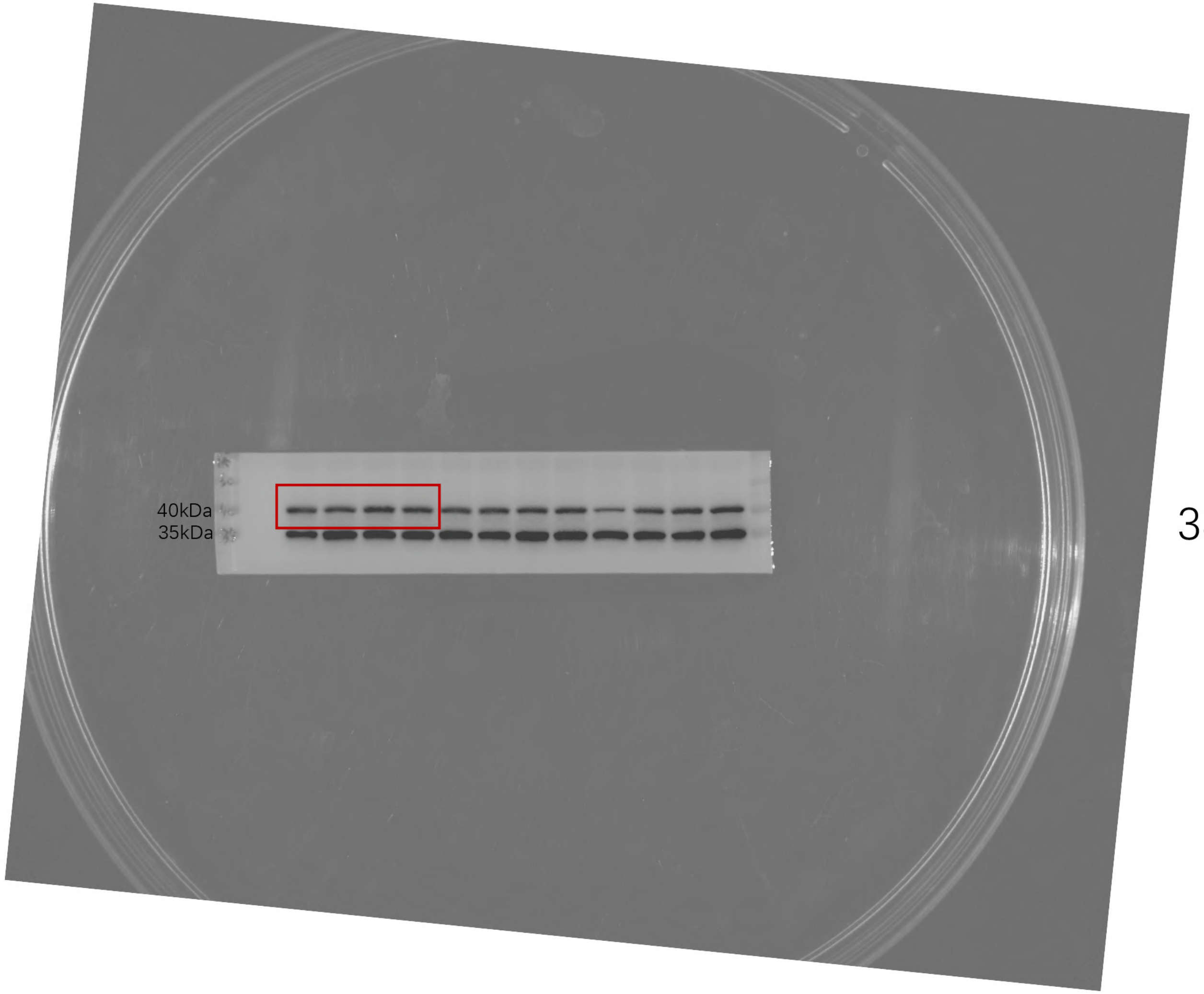

38kDa

Source Fig.6C p38

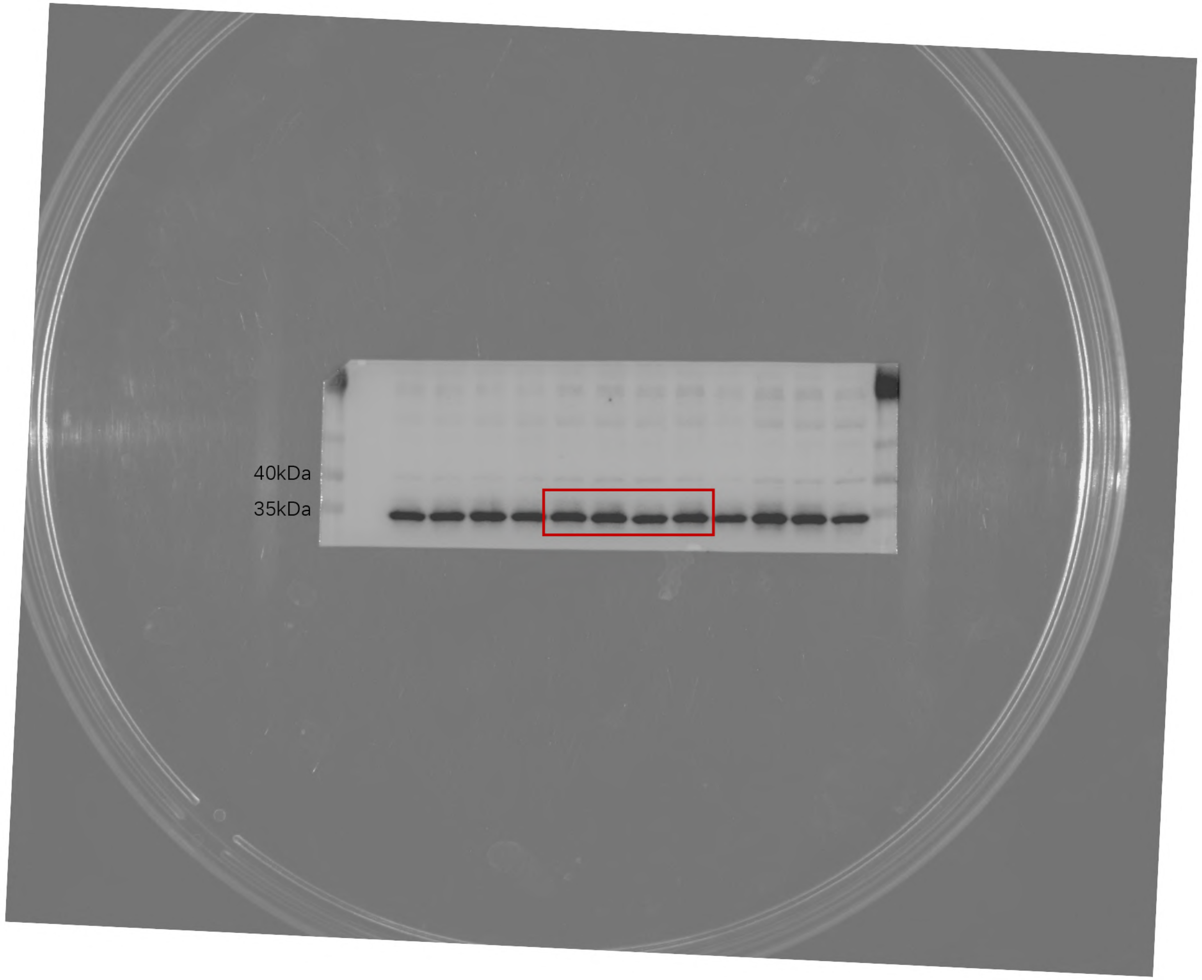

38kDa

Source Fig.6C p-TRAF2

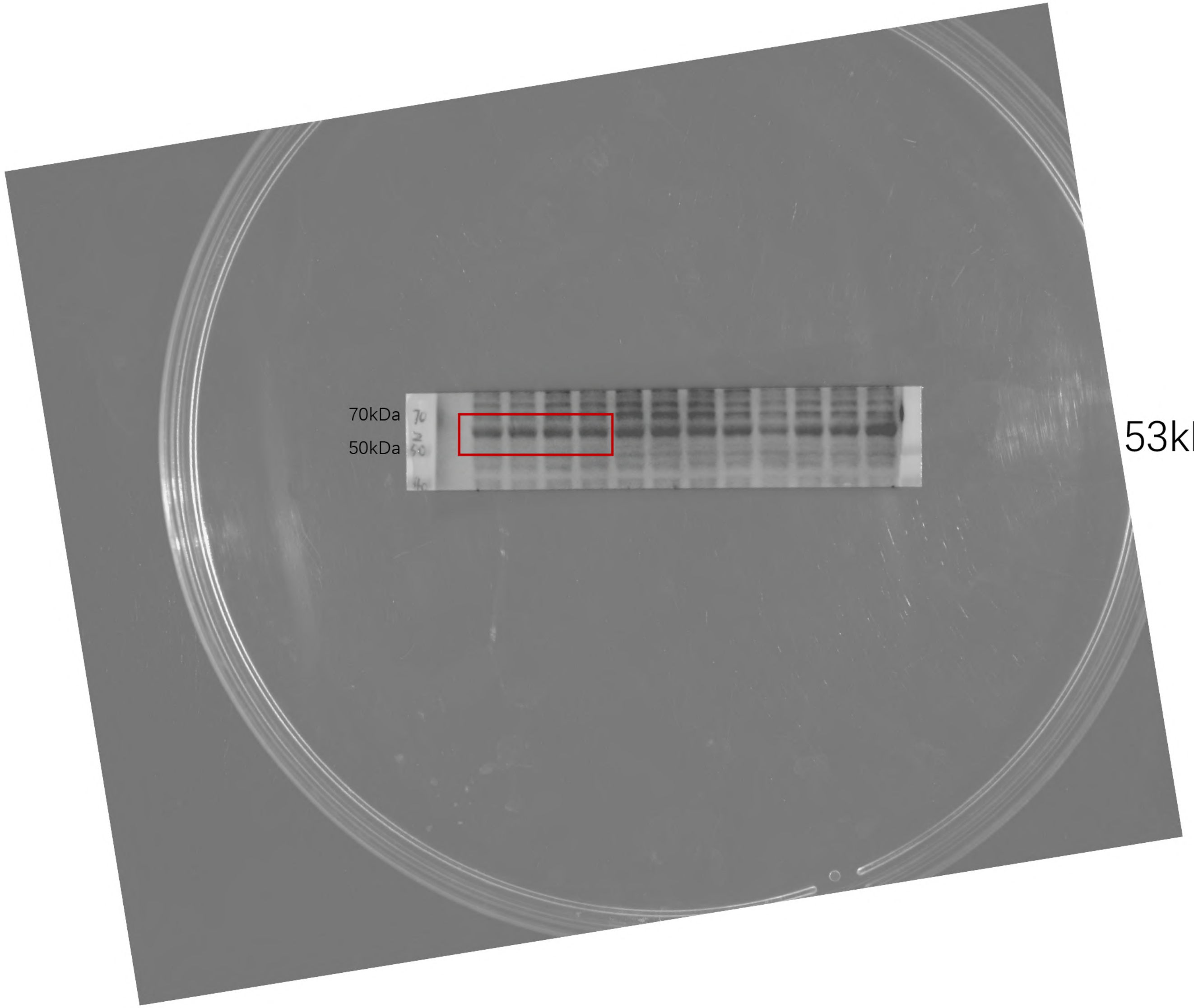

Source Fig.6C GAPDH

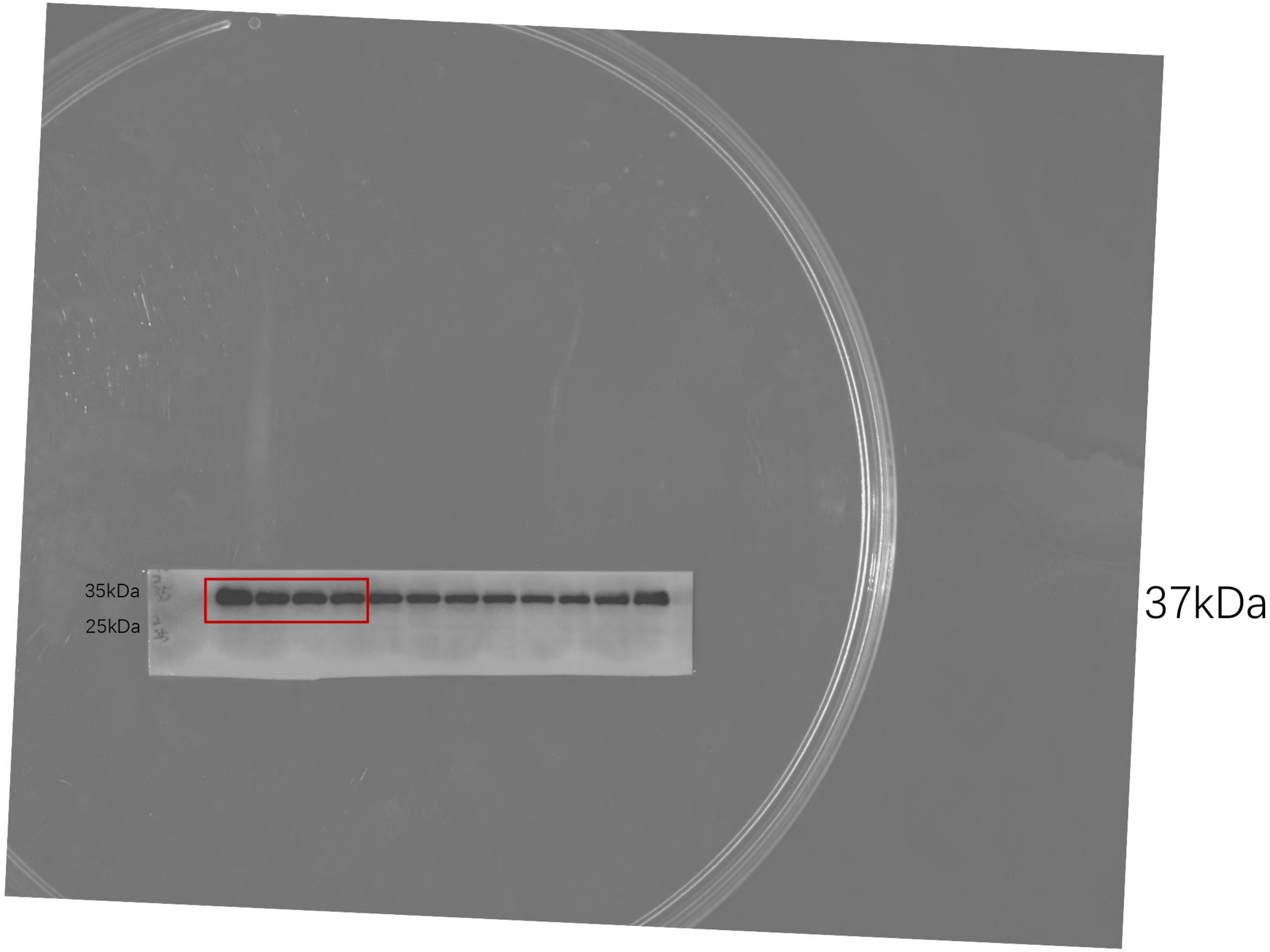

Source Fig.6C TRAF2

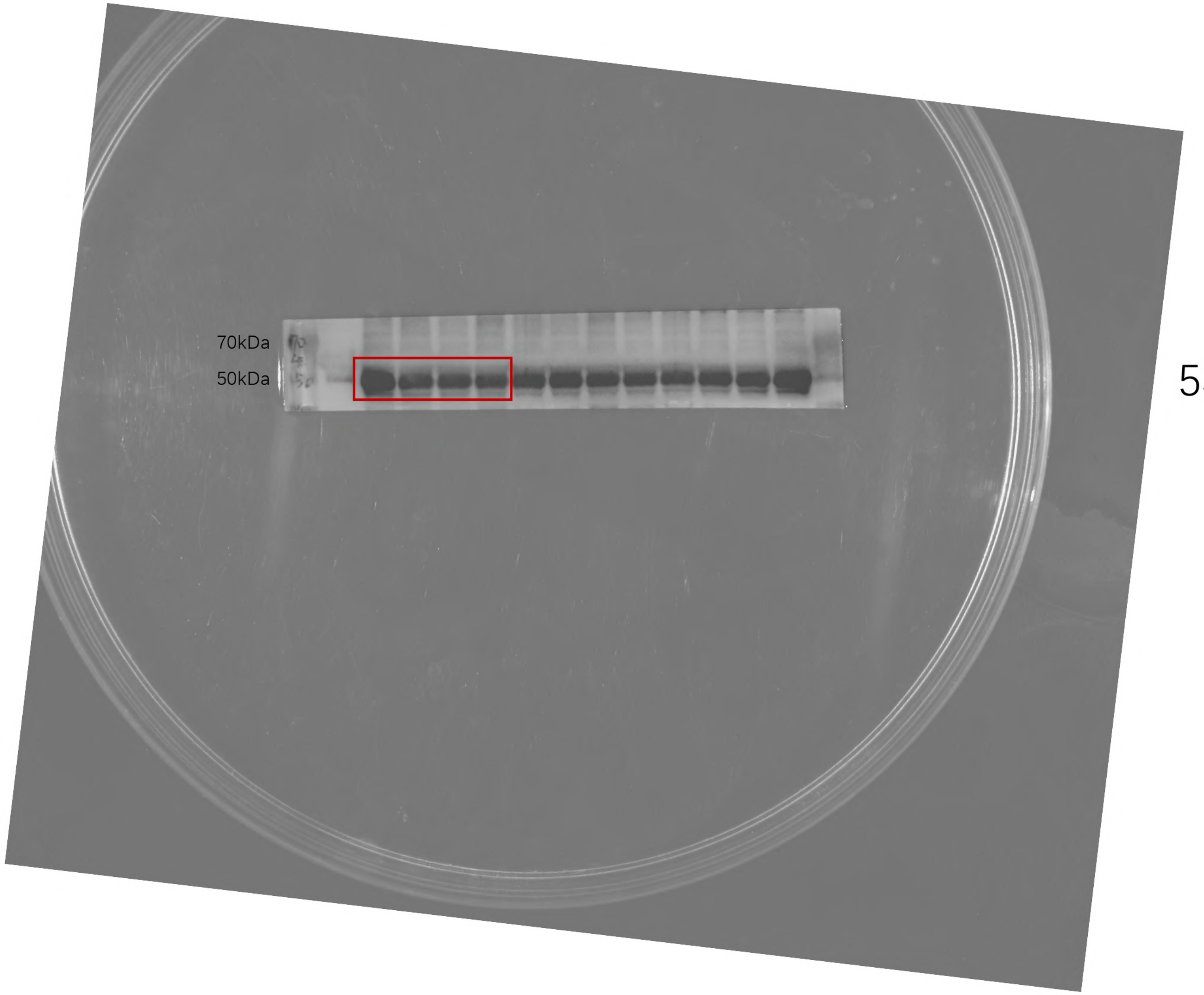

53kDa

Source Fig.6C GAPDH

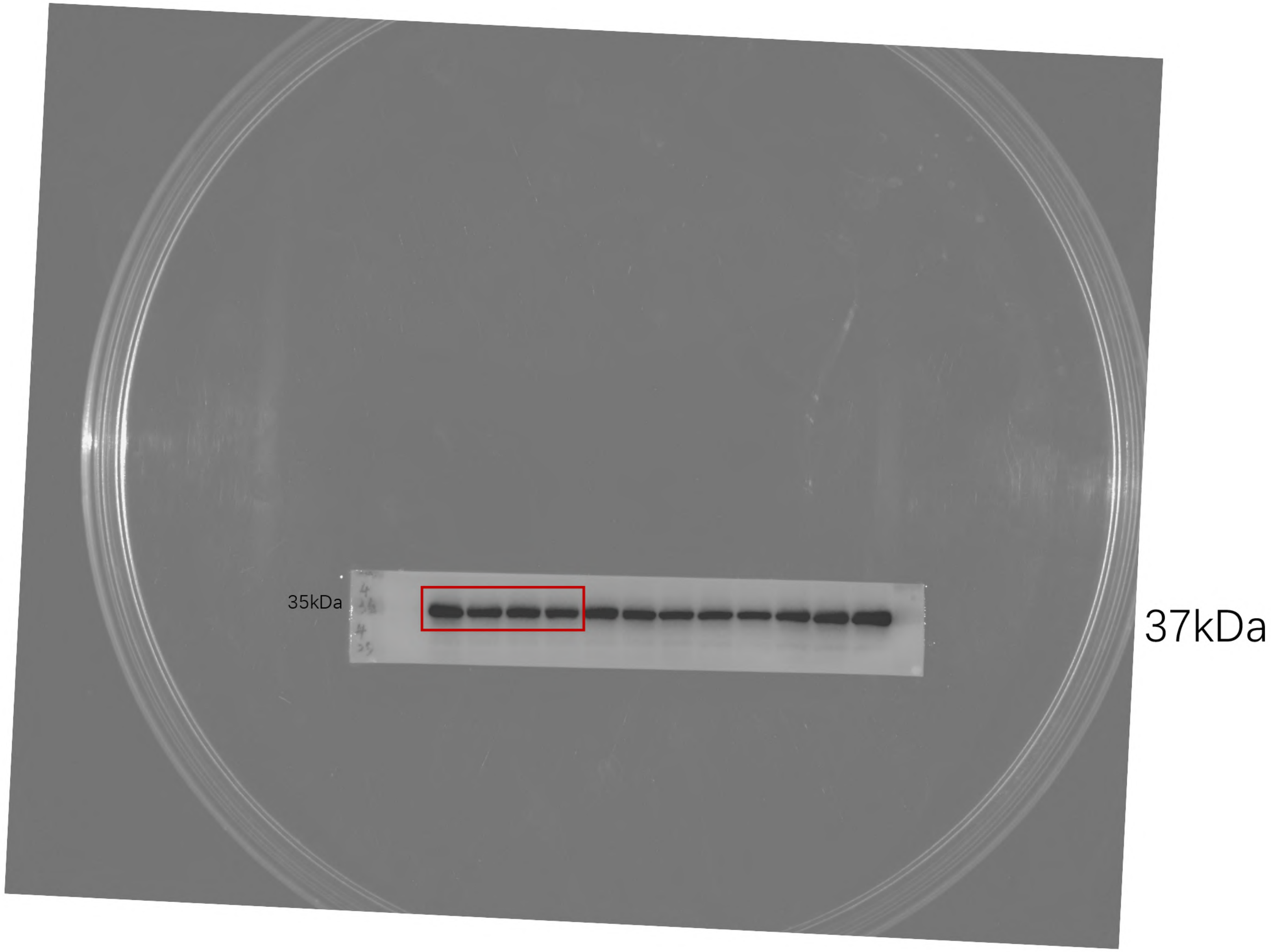

Source Fig.6C Fn14

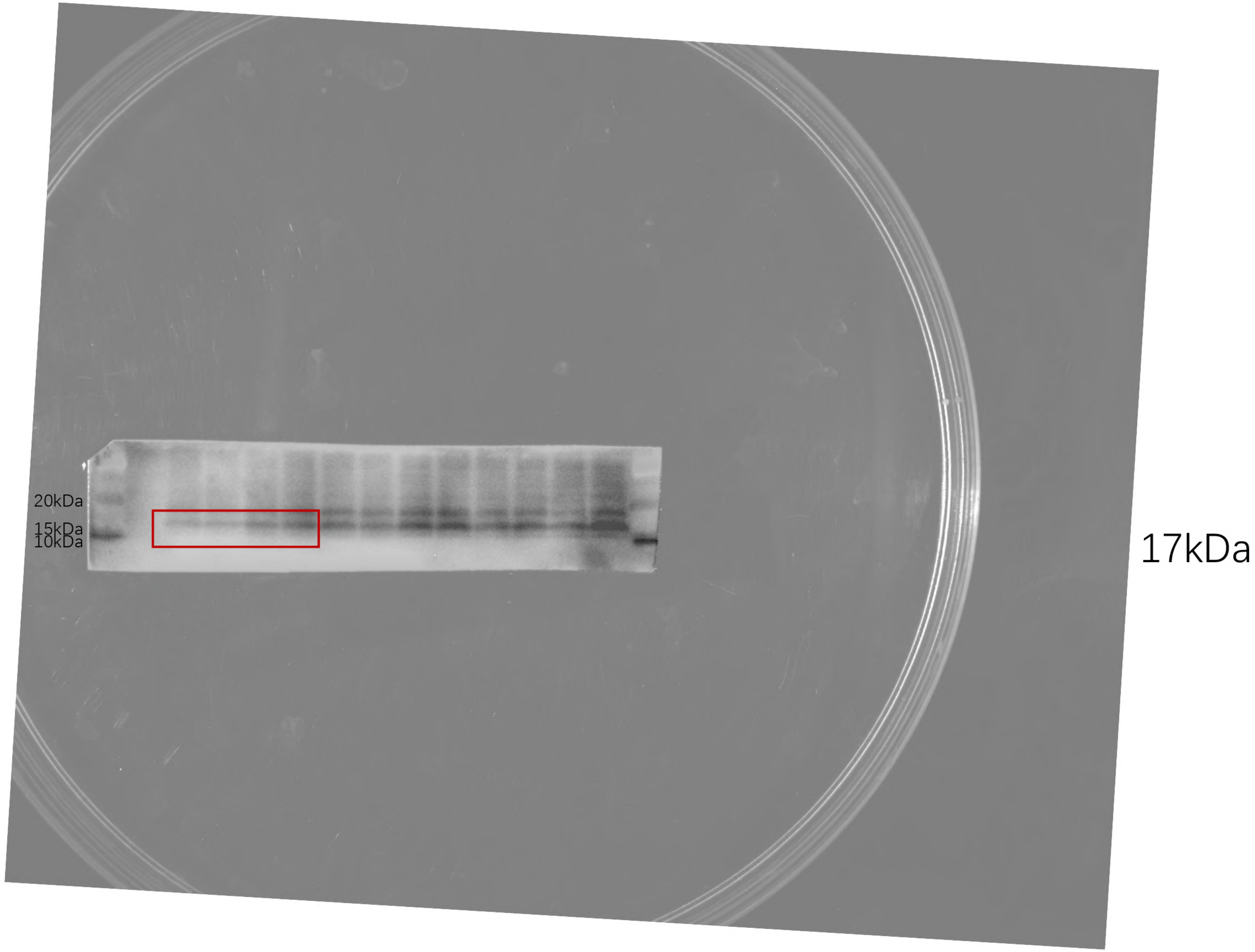

Source Fig.6C GAPDH

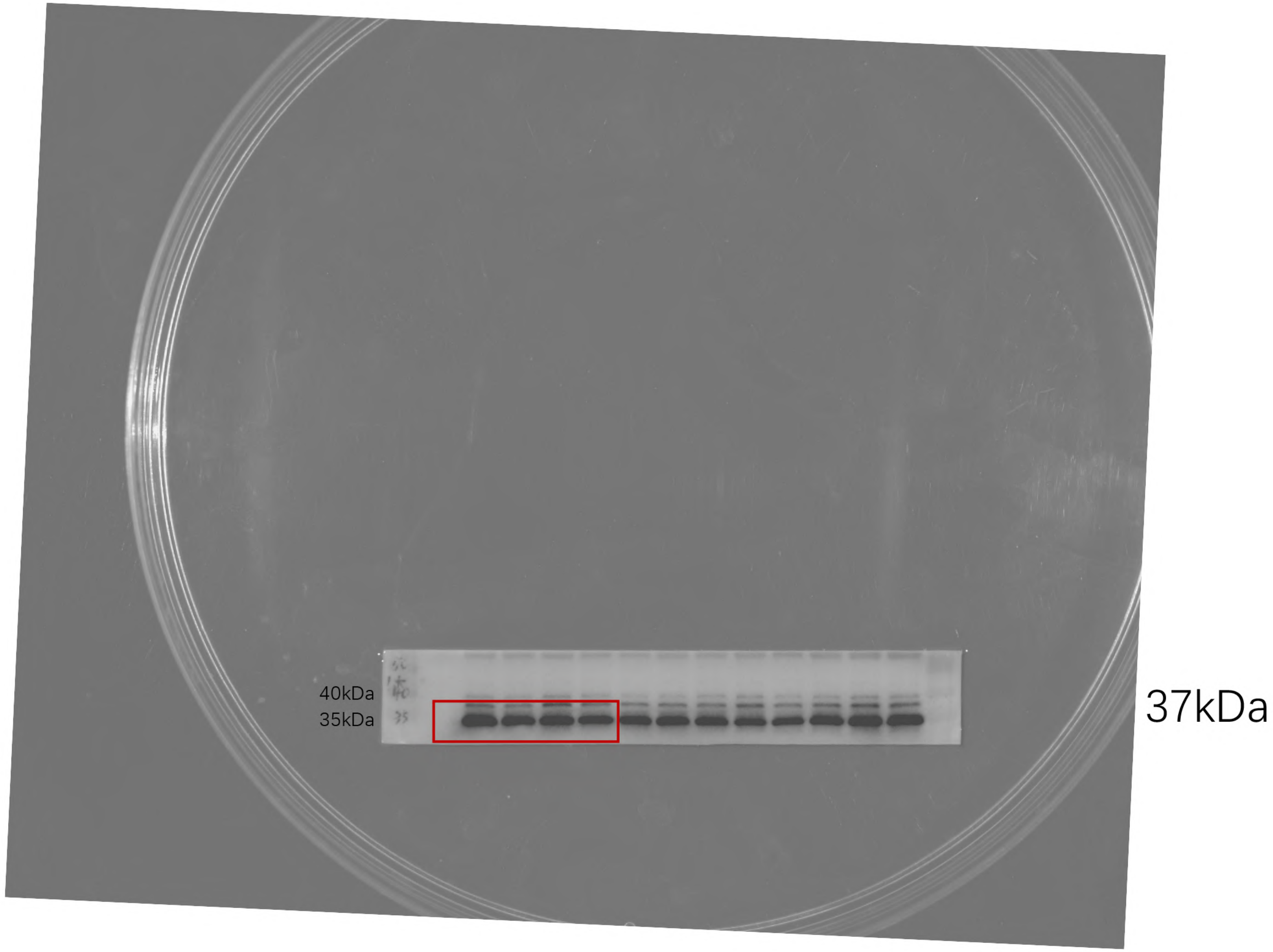

Source Fig.6D Fn14

20kDa

15kDa

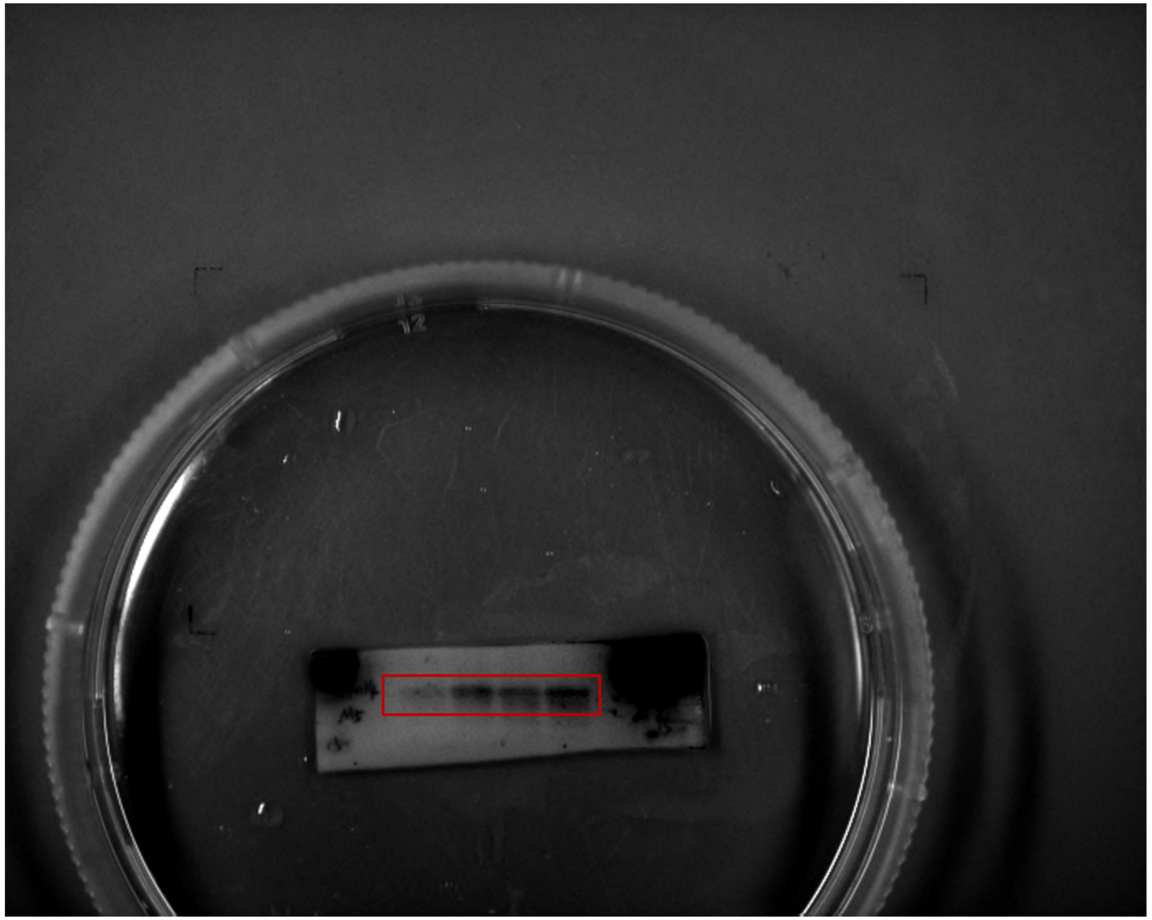

17kDa

Source Fig.6D b-actin

40kDa
37kDa

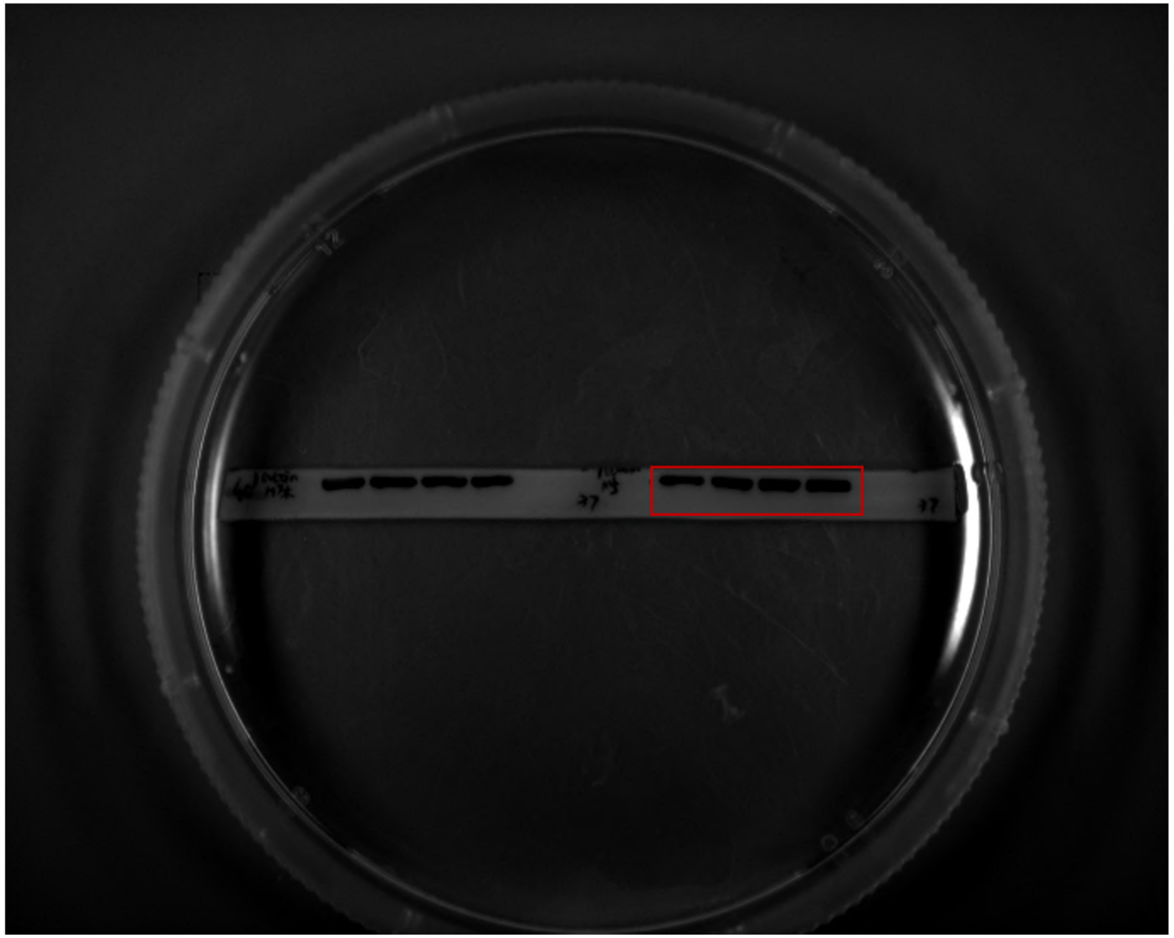

45kDa

Source Fig.6D p-ERK1/2

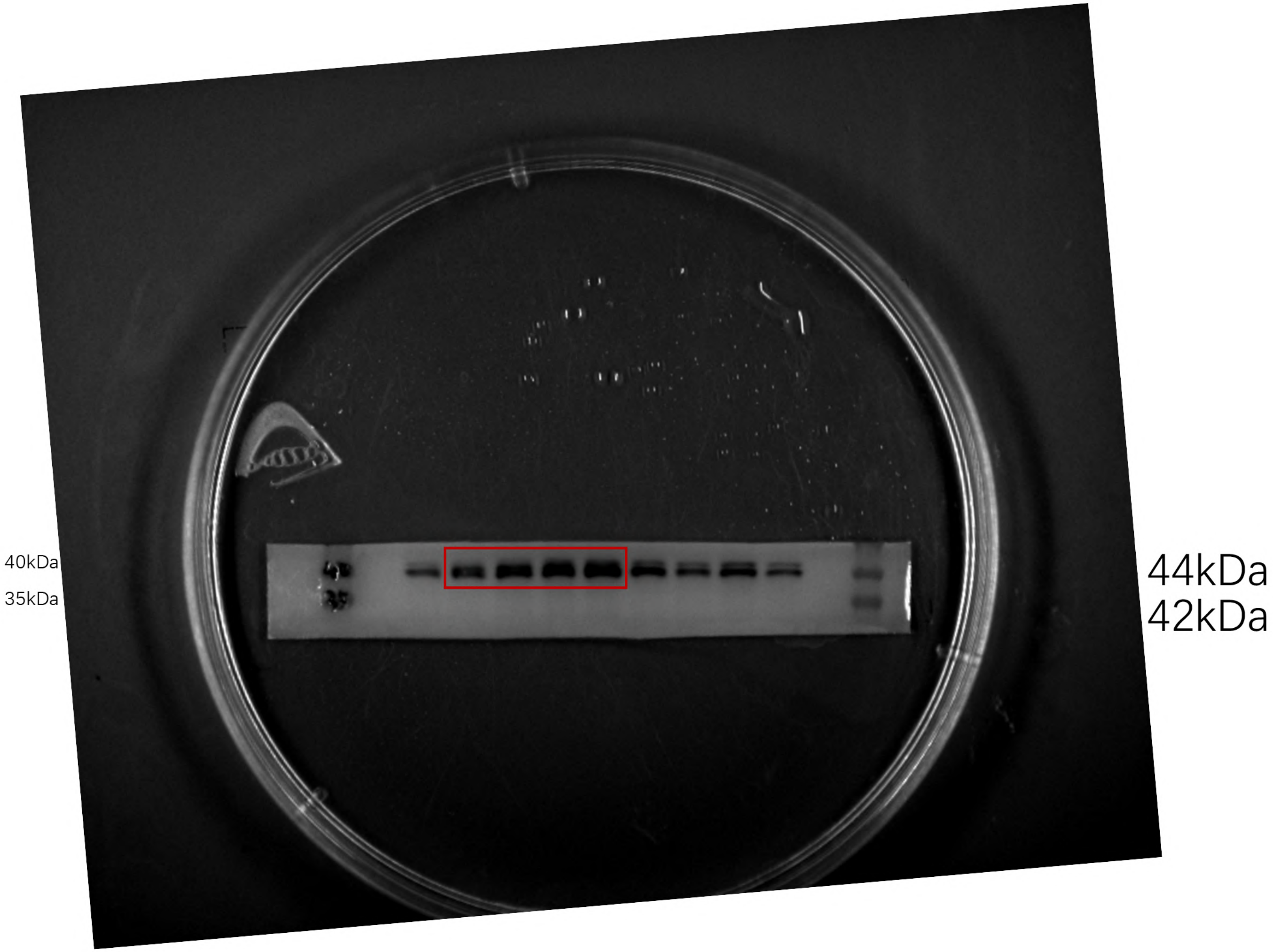

Source Fig.6D ERK1/2

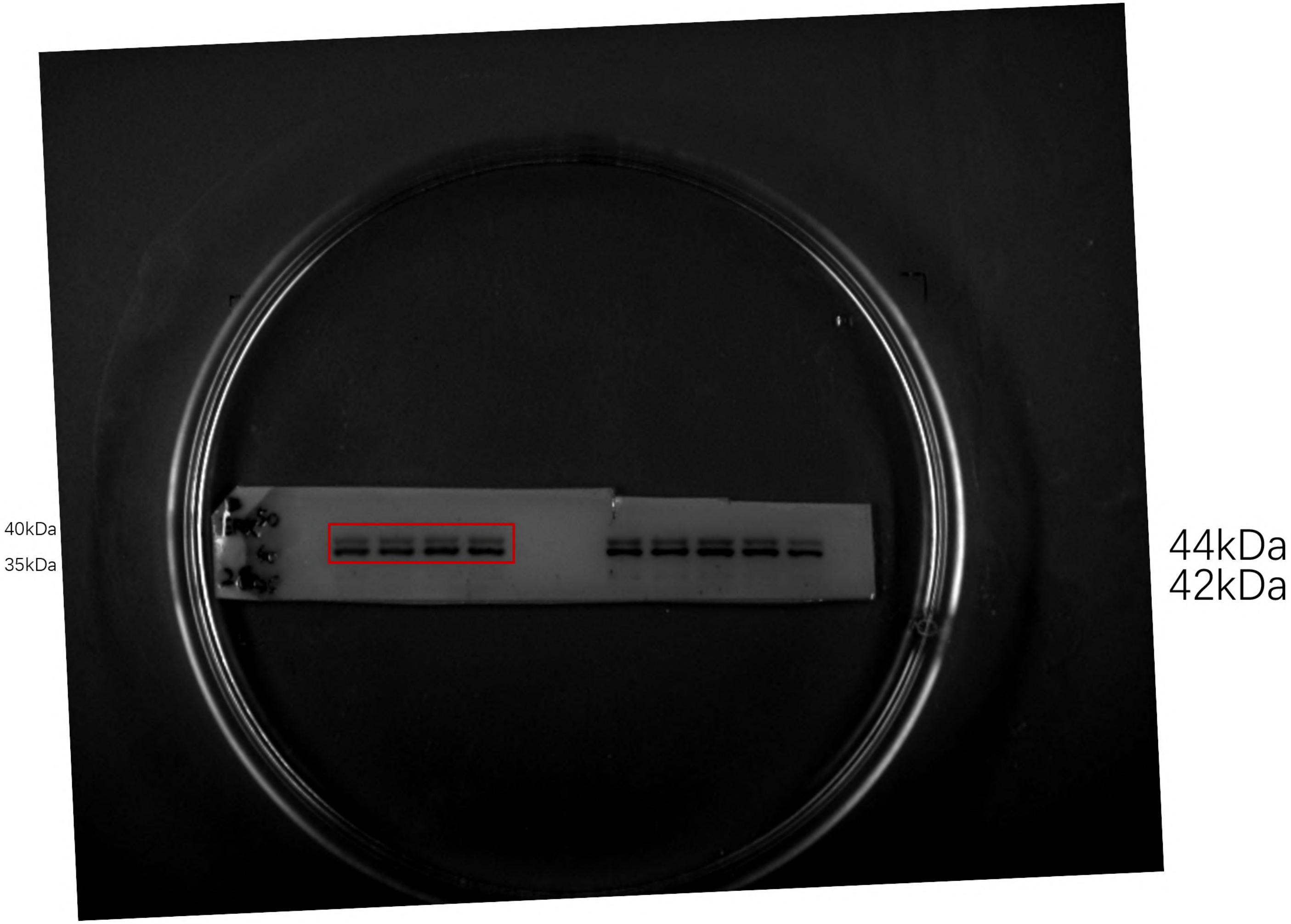

Source Fig.6D p-JNK

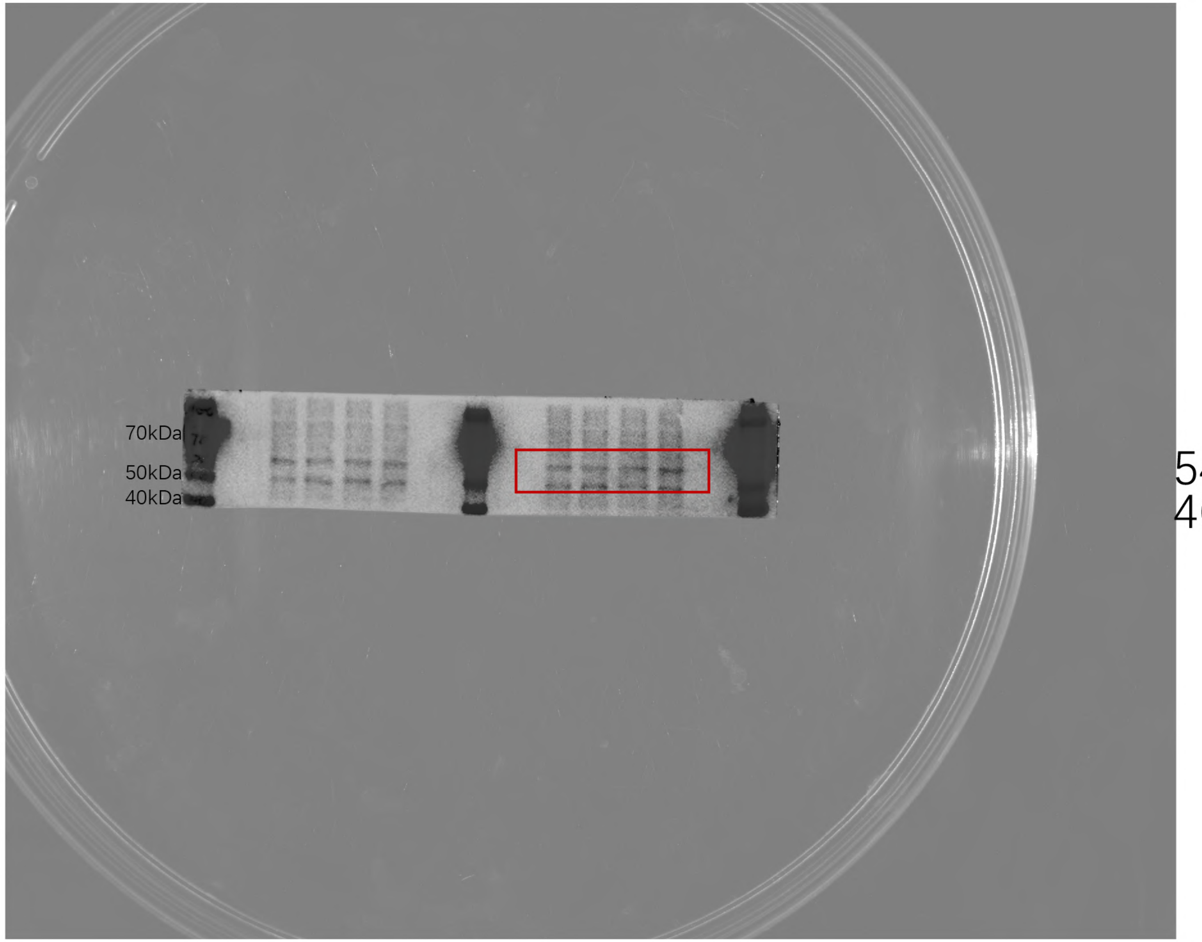

54kDa
46kDa

Source Fig.6D JNK

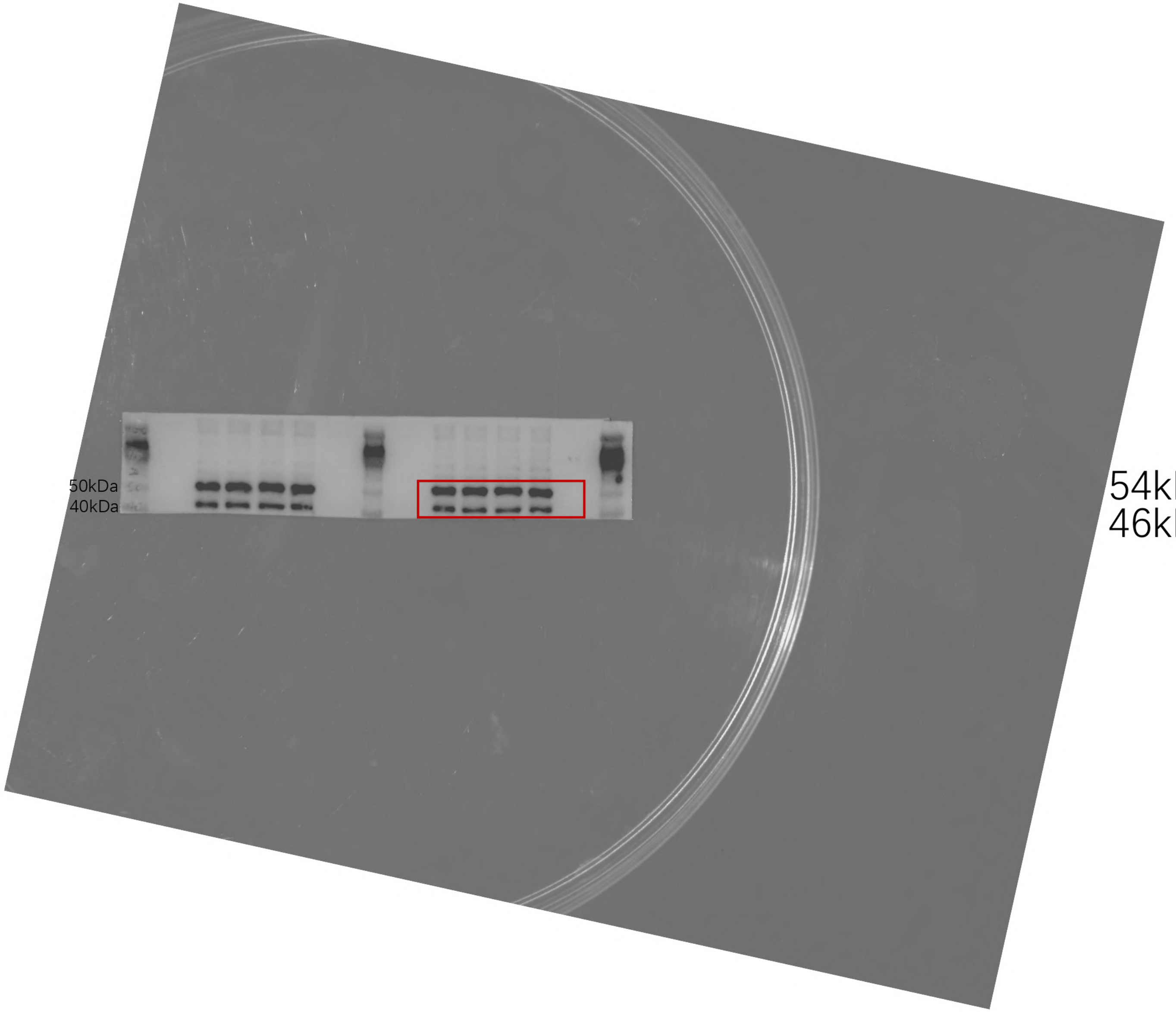

54kDa
46kDa

Source Fig.6D p-p38

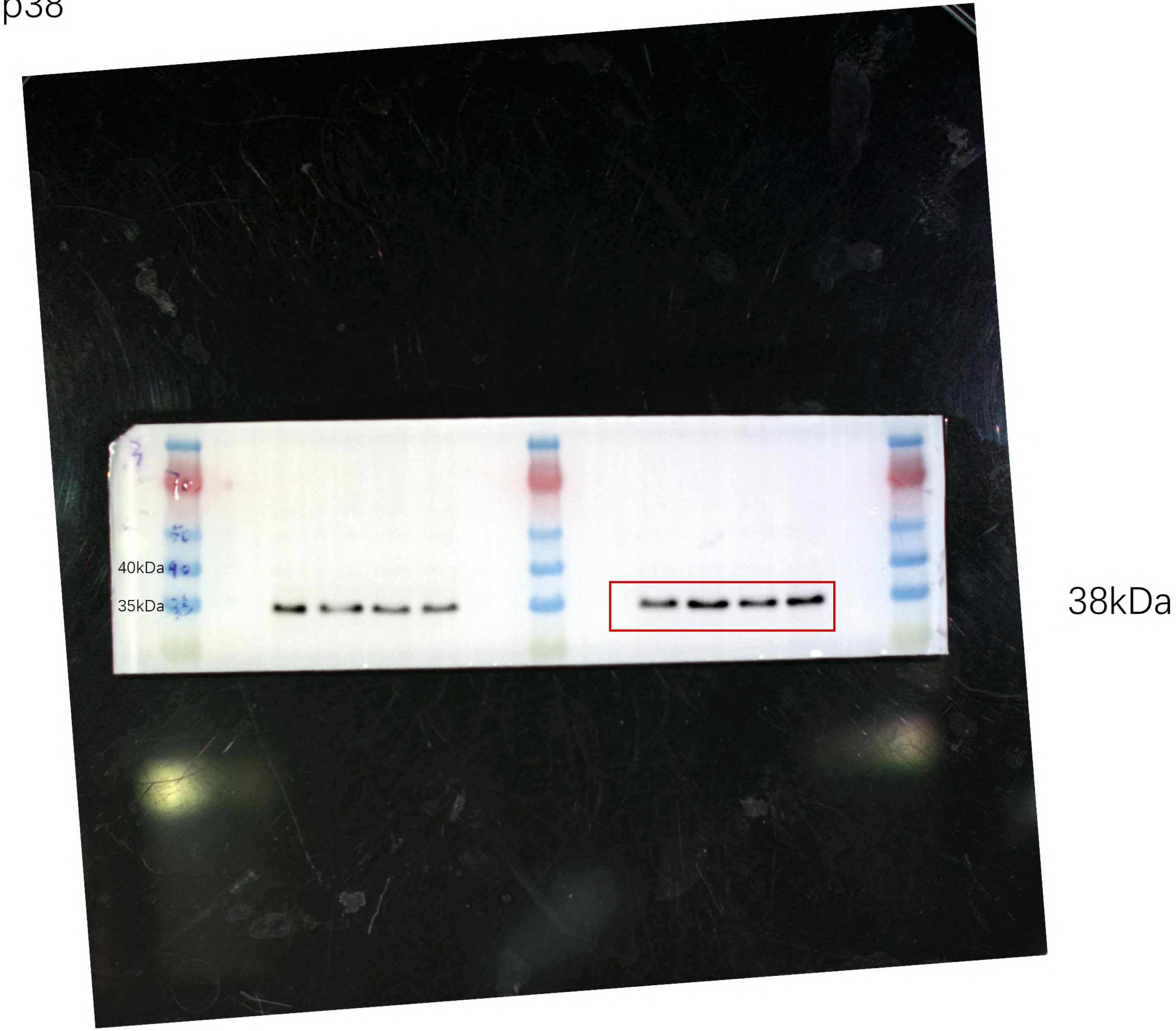

Source Fig.6D GAPDH

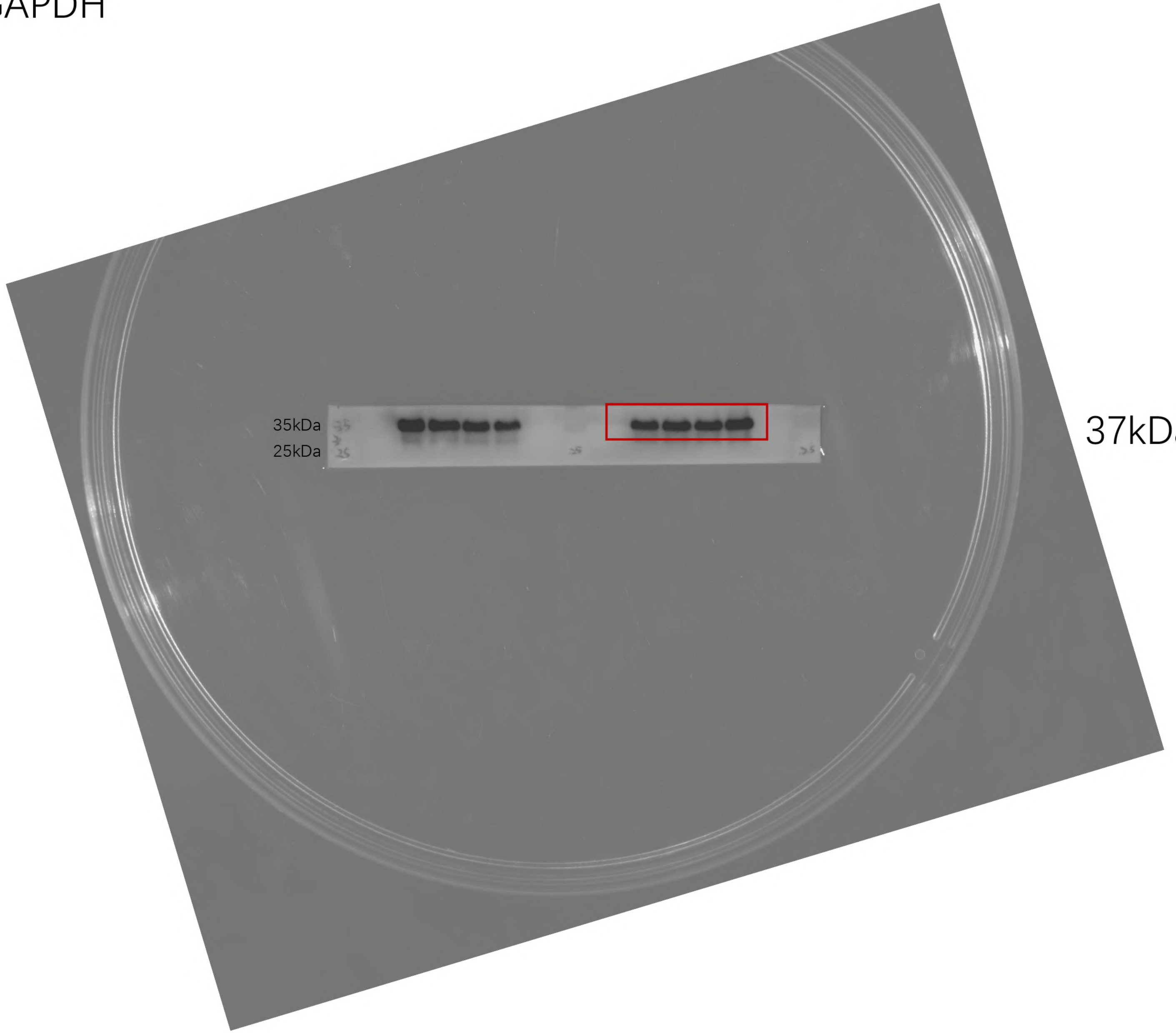

Source Fig.6D p38

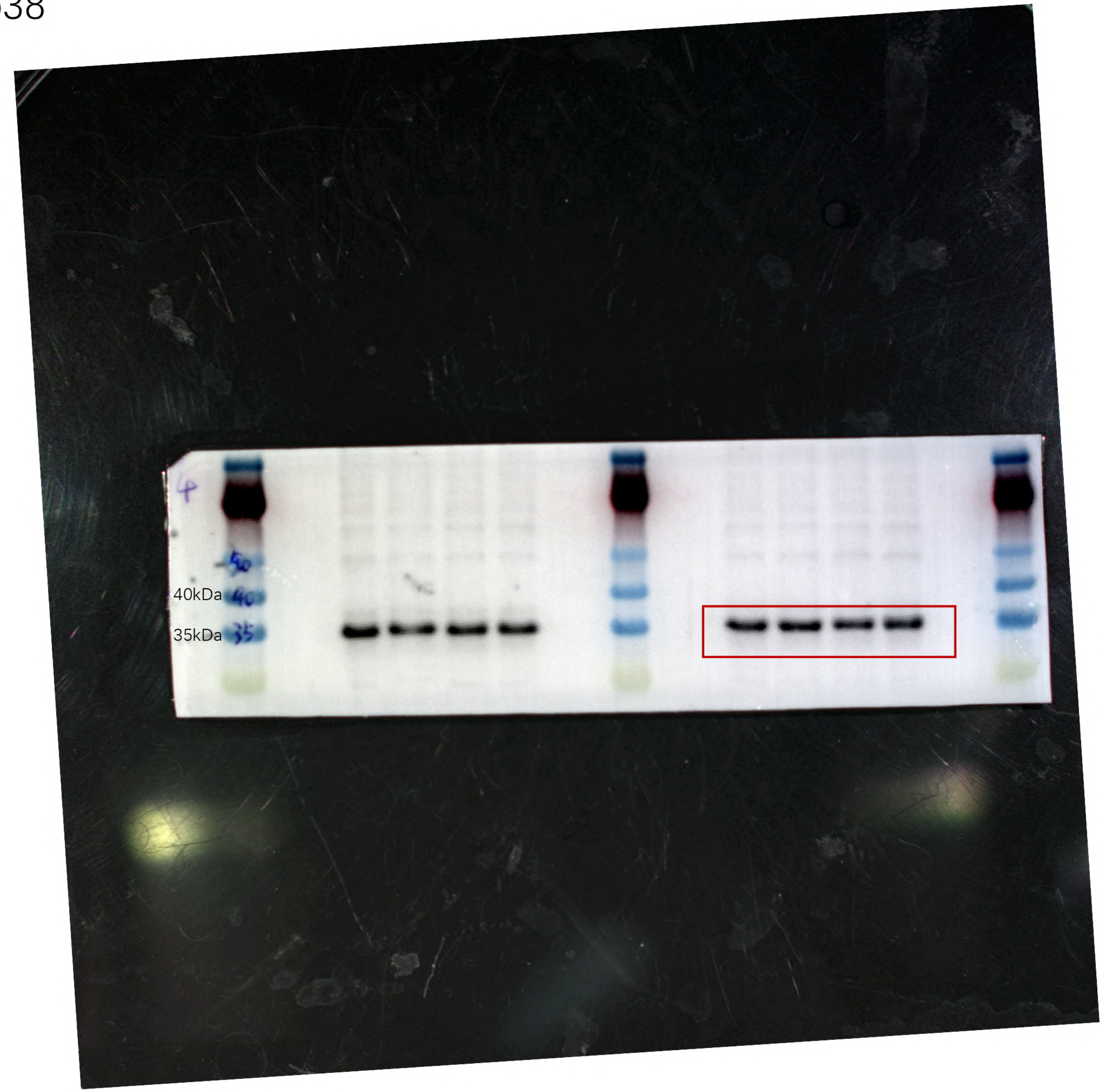

Source Fig.6D GAPDH

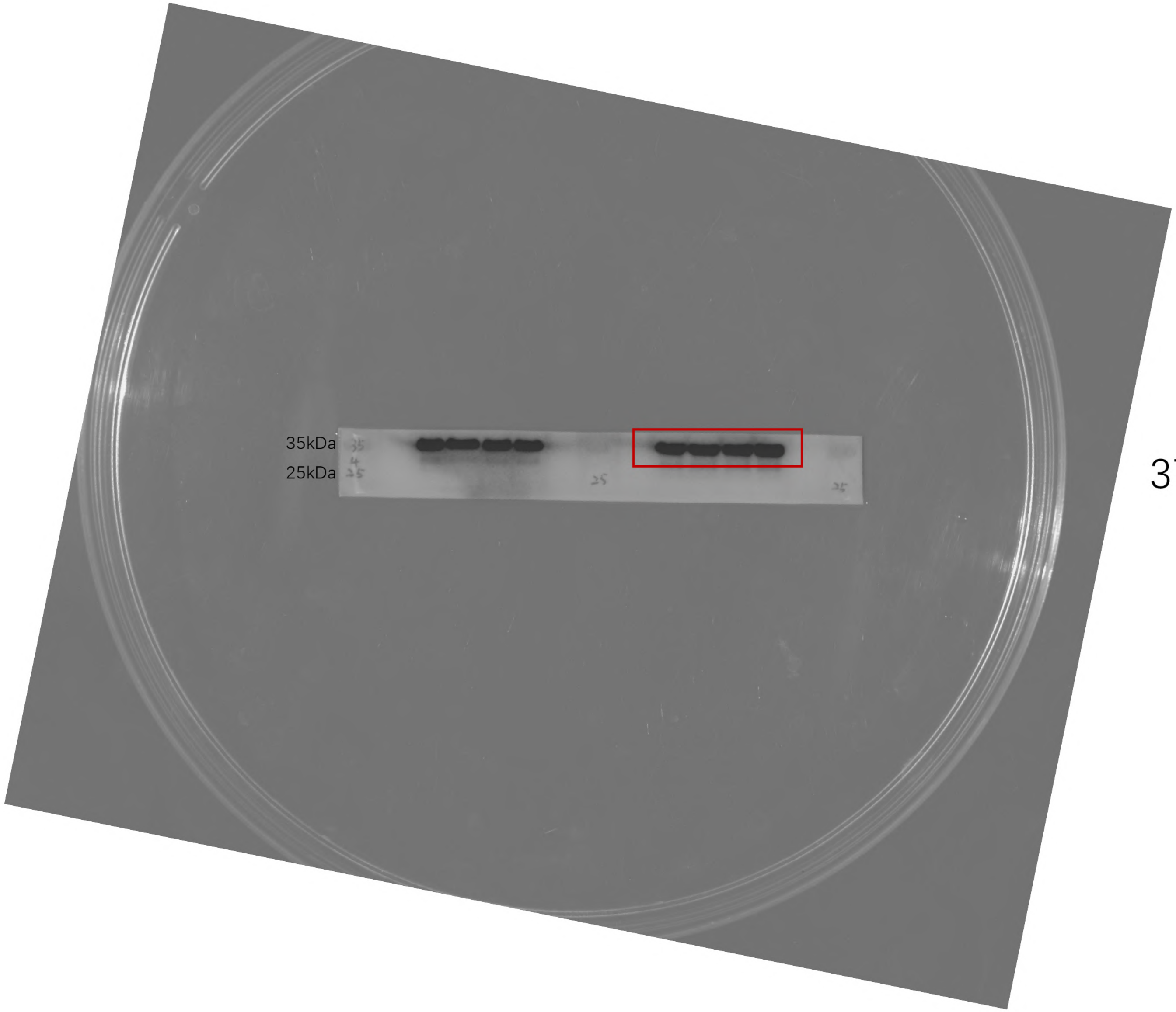

37kDa

Source Fig.6D p-TRAF2

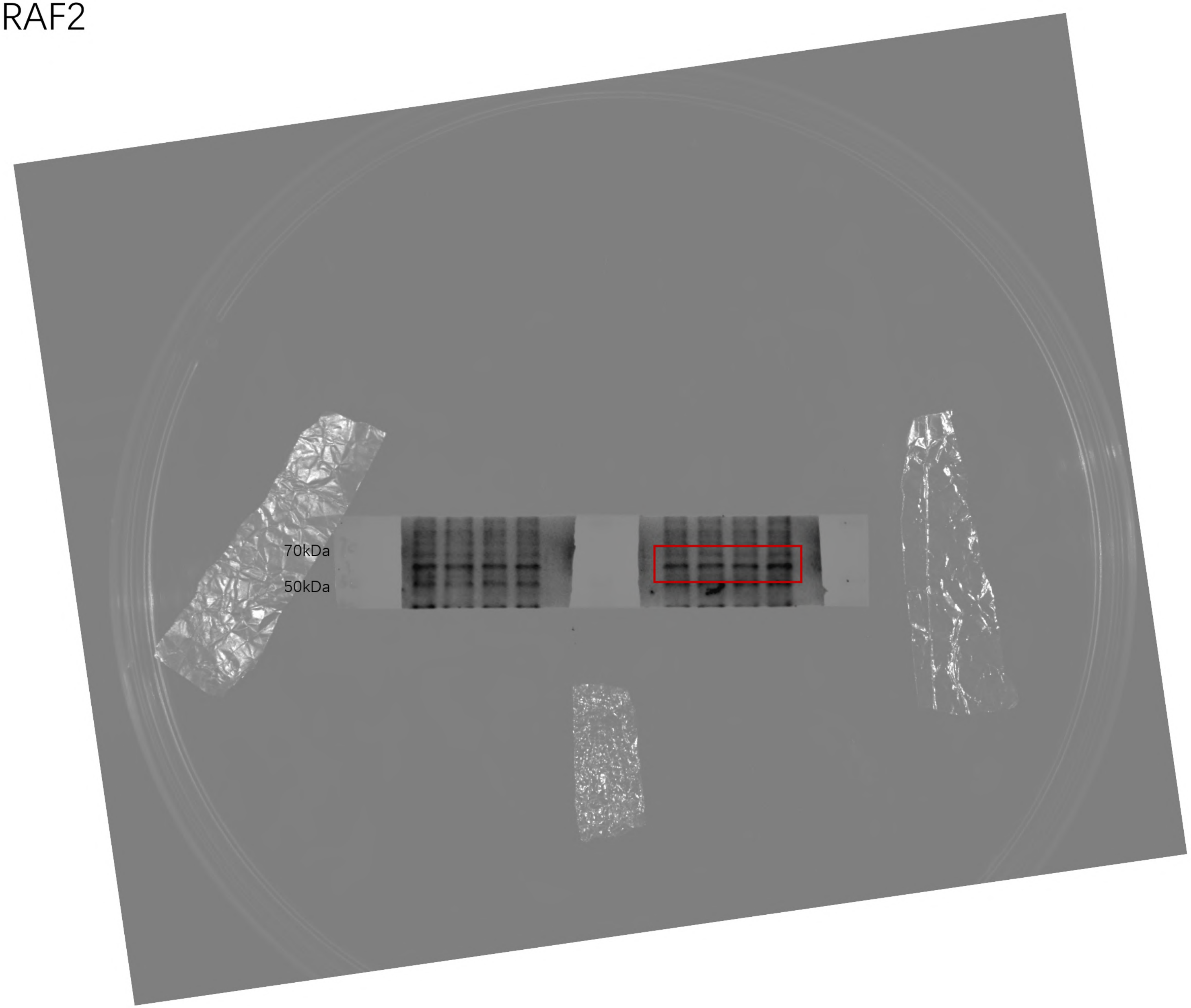

53kDa

Source Fig.6D TRAF2

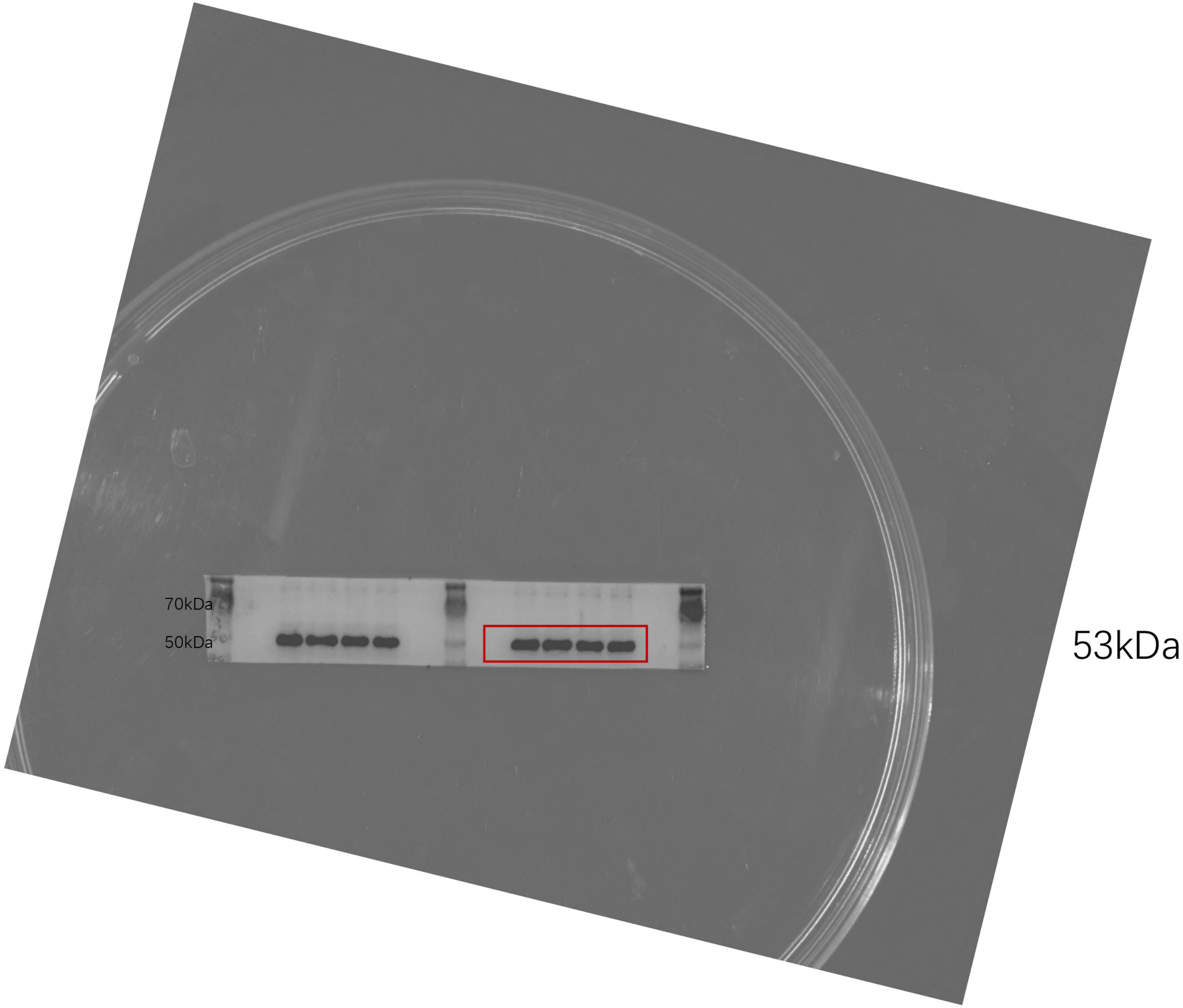

Source Fig.6D 24P3R

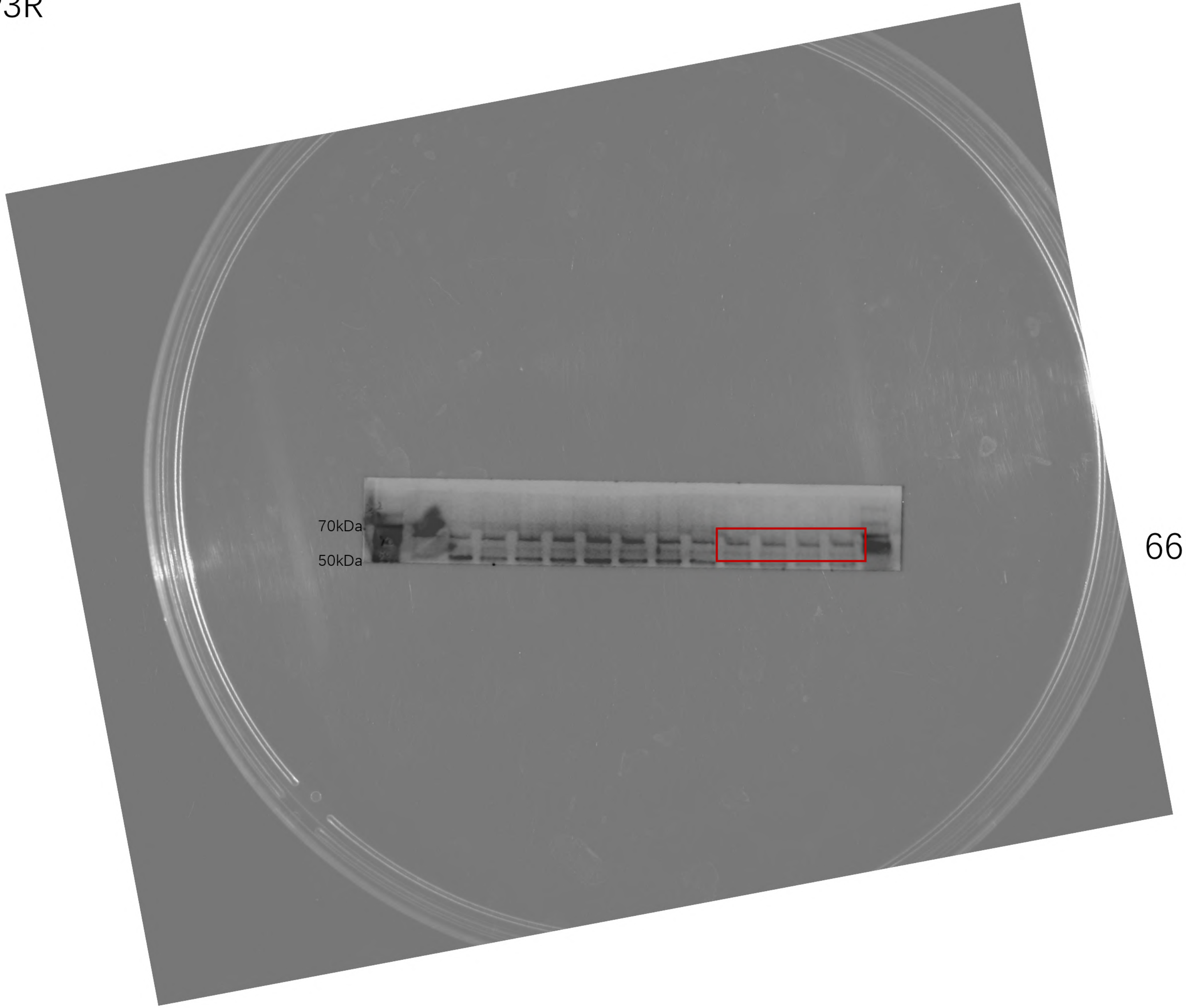

66kDa

Source Fig.6D GAPDH

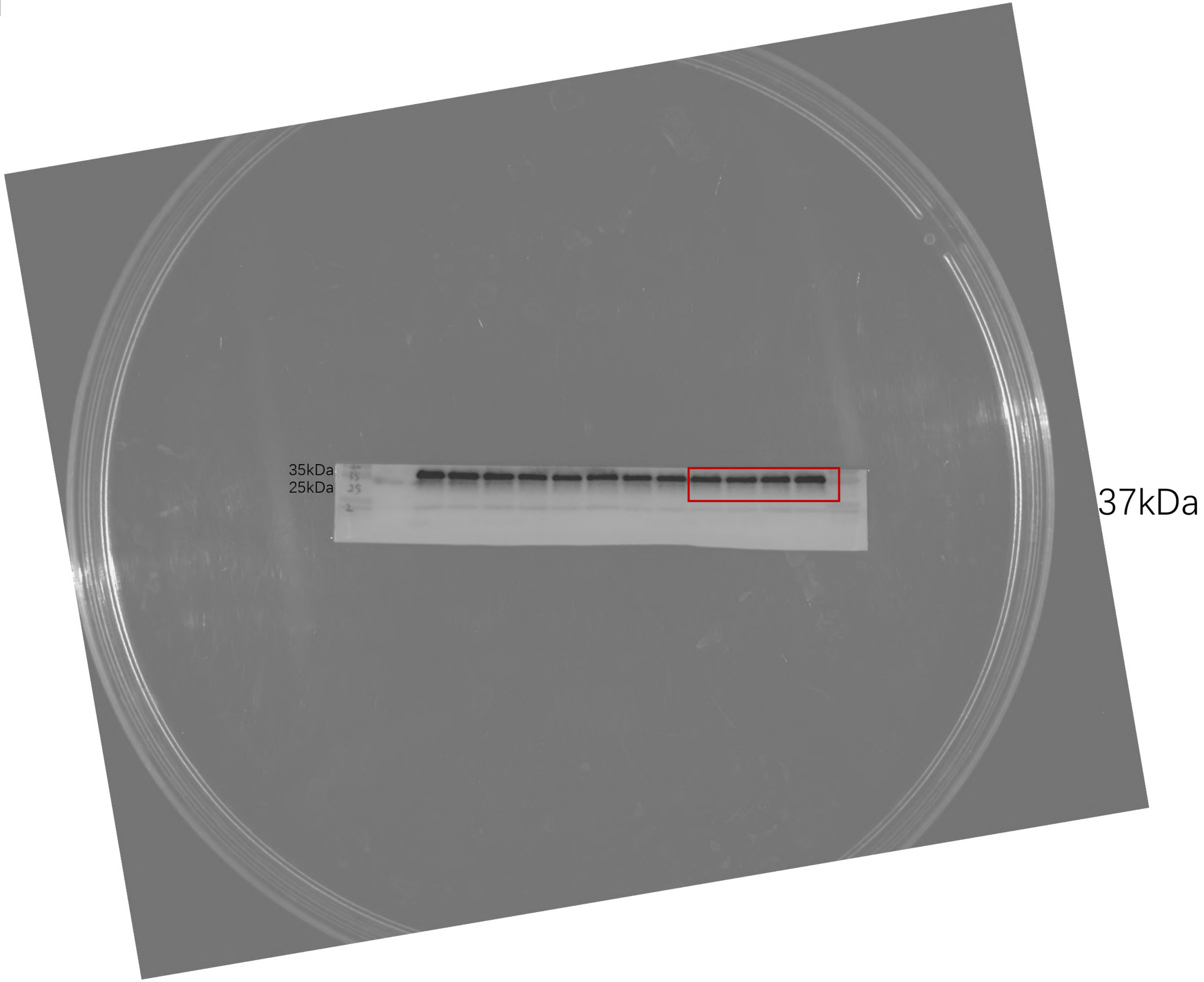

Source Fig.7A Fn14

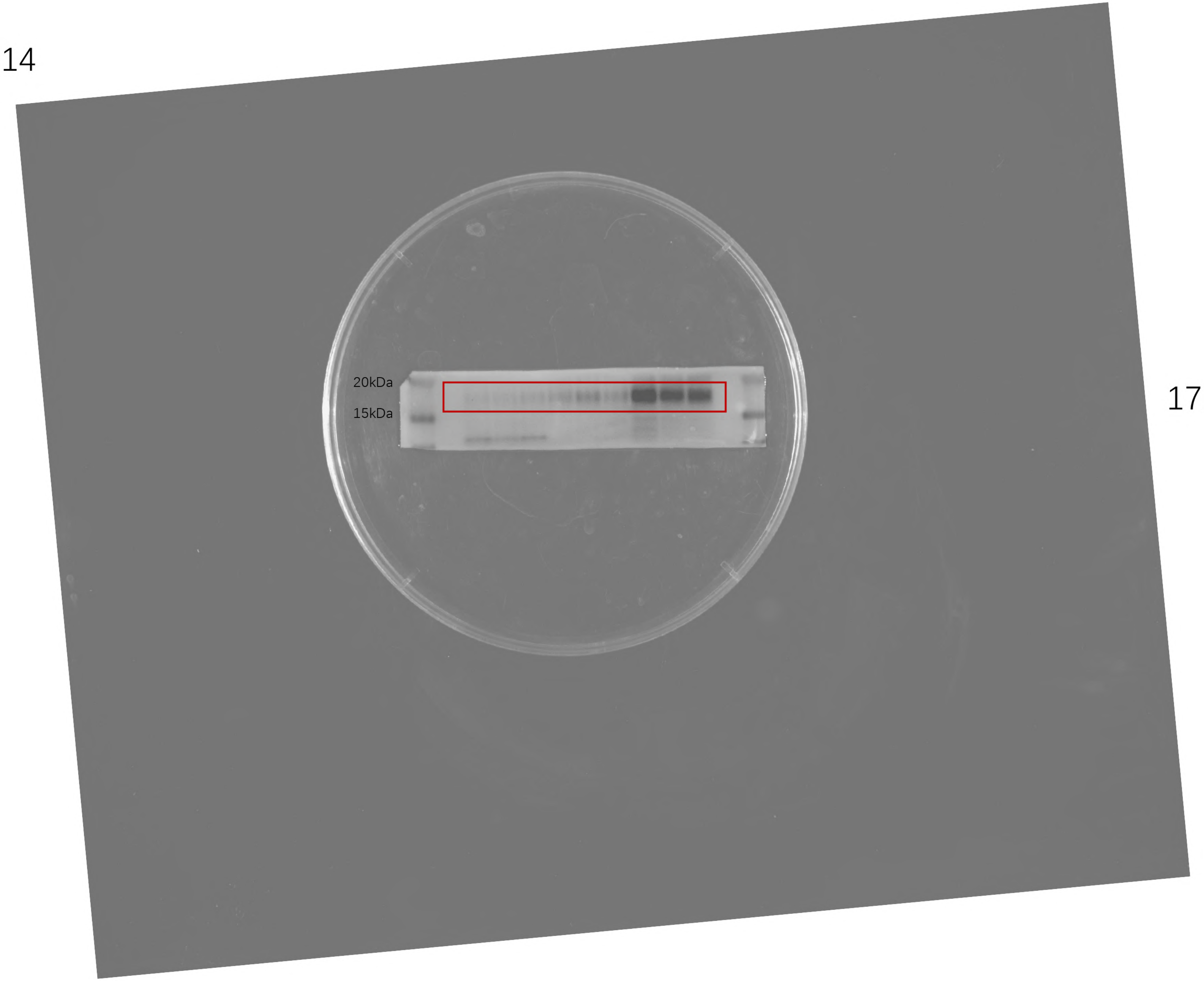

17kDa

Source Fig.7A GAPDH

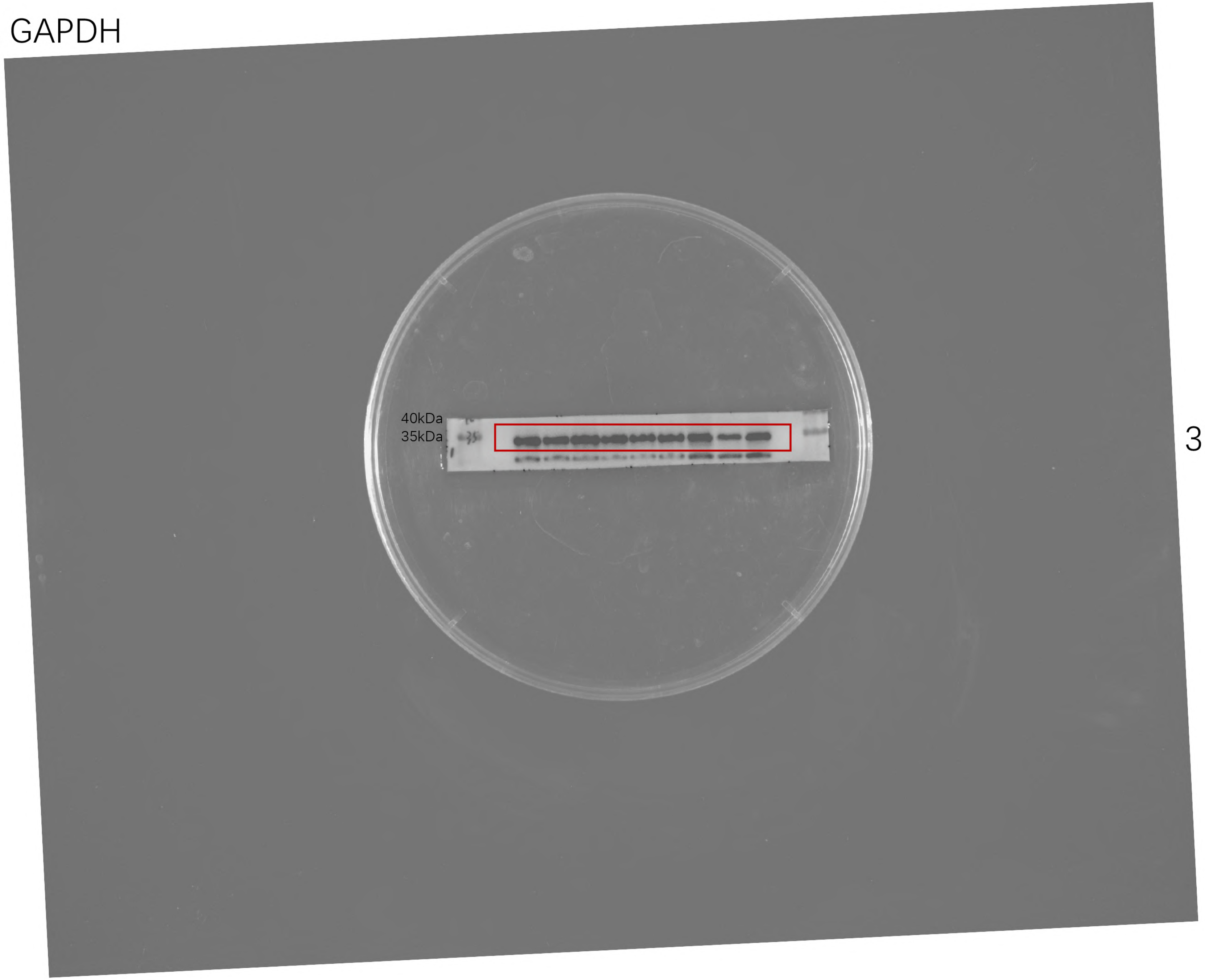

37kDa

Source Fig.7A p-ERK1/2

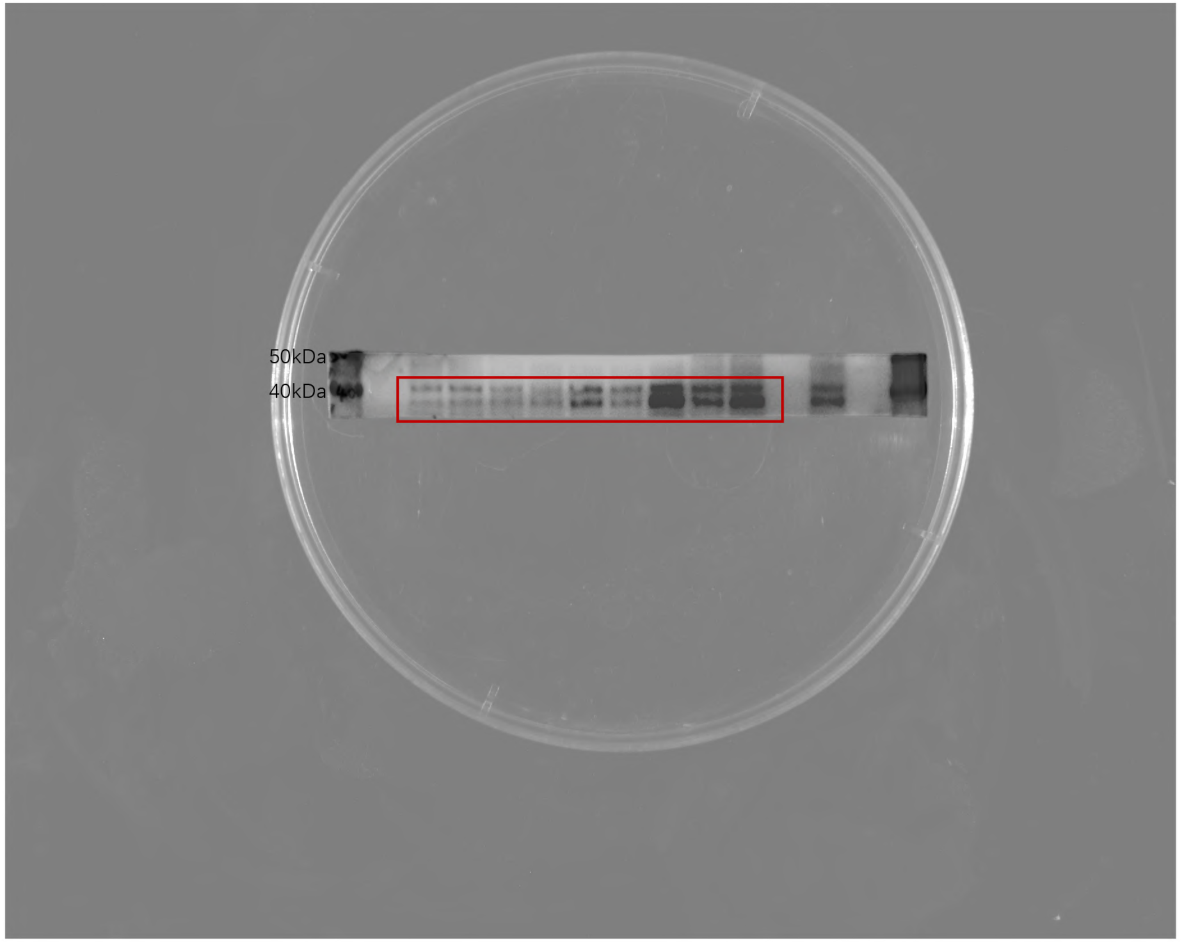

44kDa
42kDa

Source Fig.7A ERK1/2

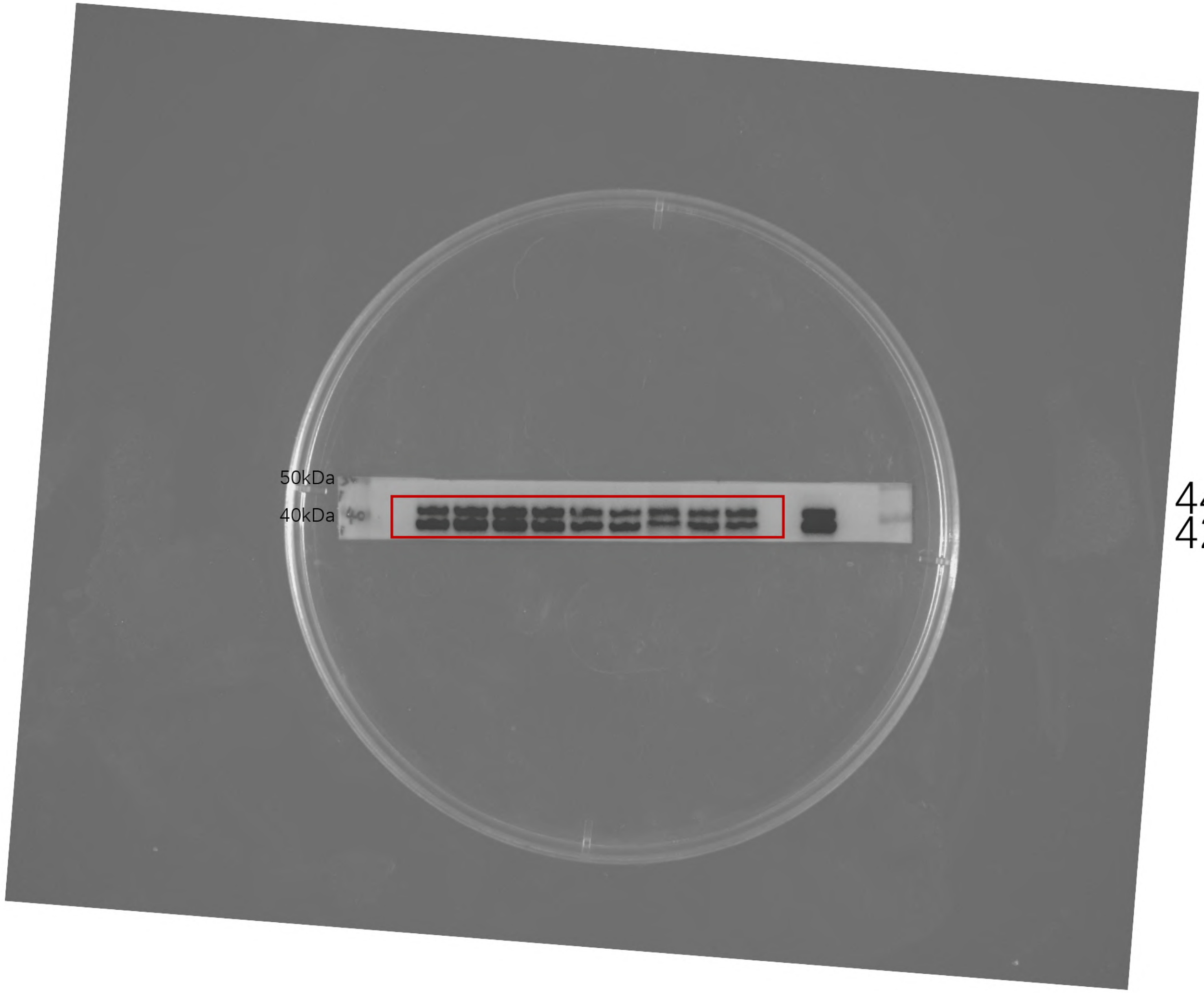

Source Fig.7A GAPDH

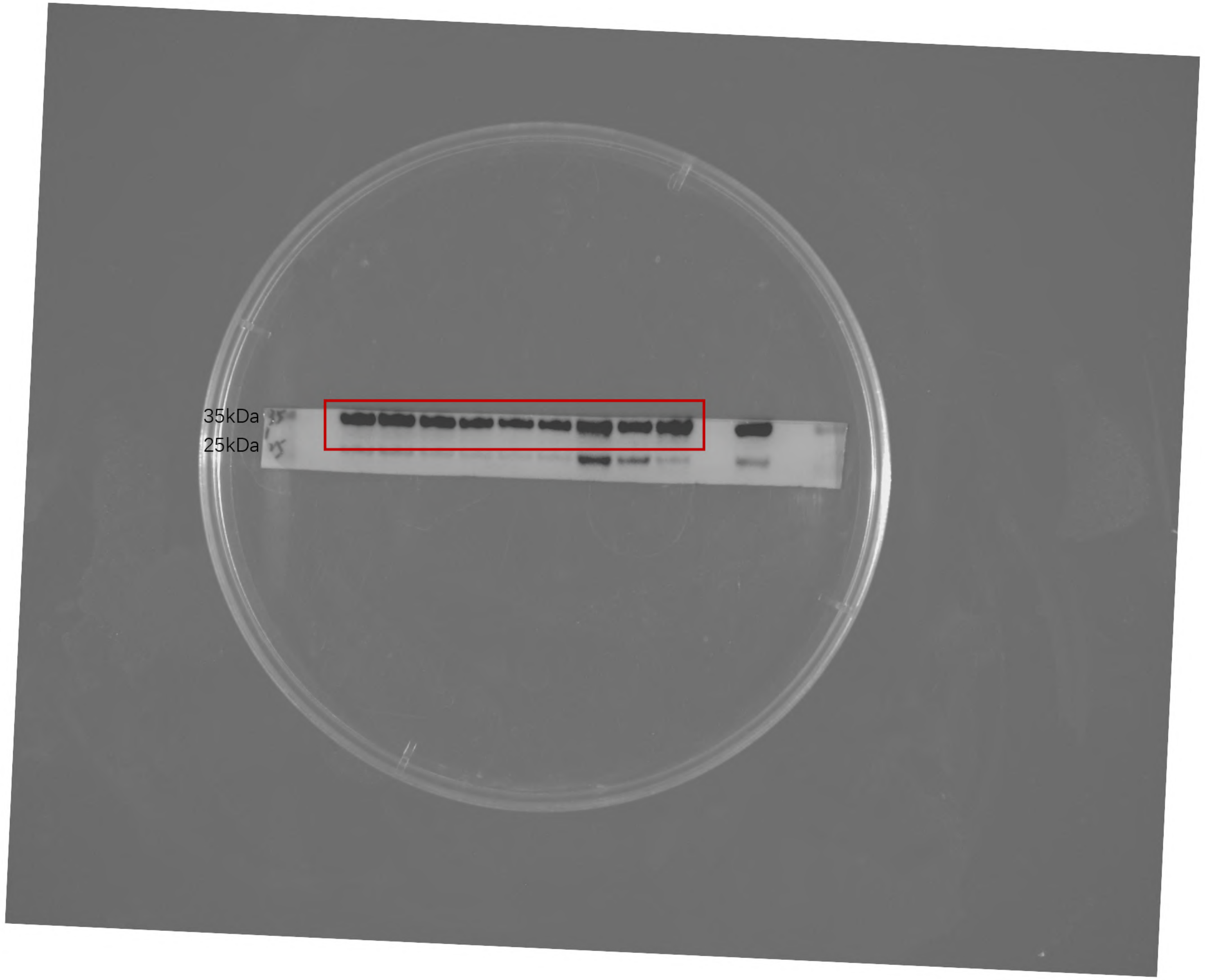

37kDa

Source Fig.7F LCN2

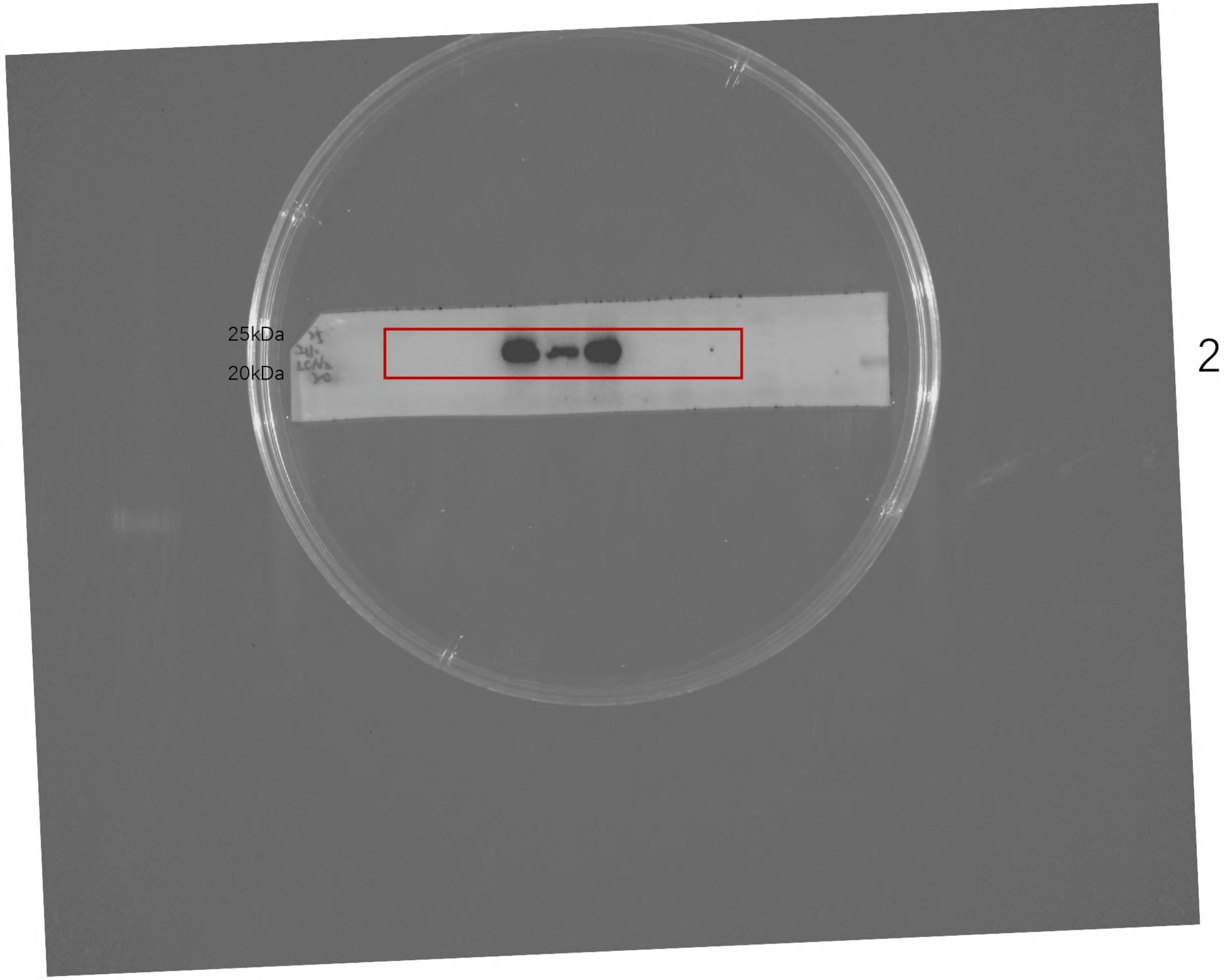

21kDa

Source Fig.7F KRT1

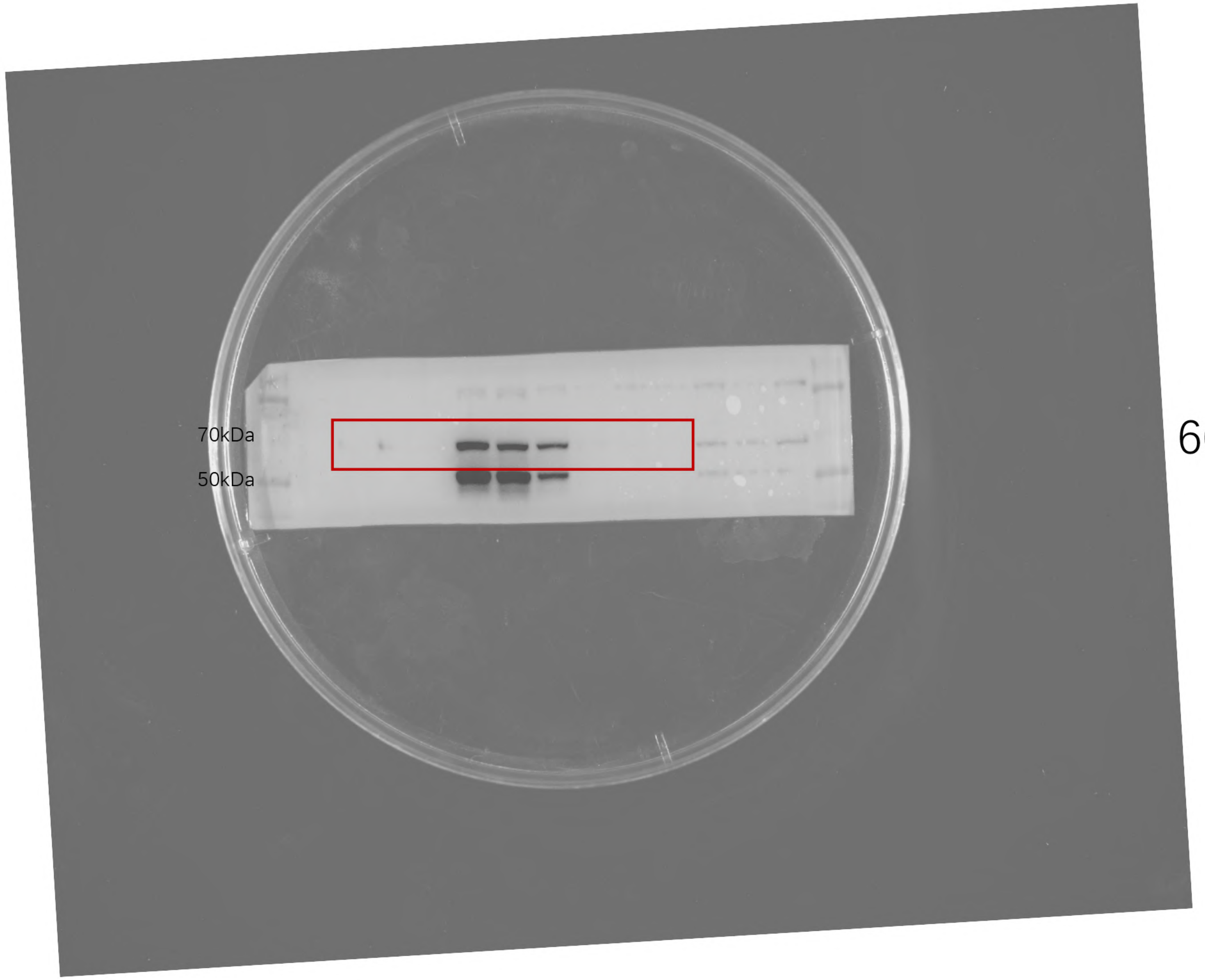

Source Fig.7F KRT5

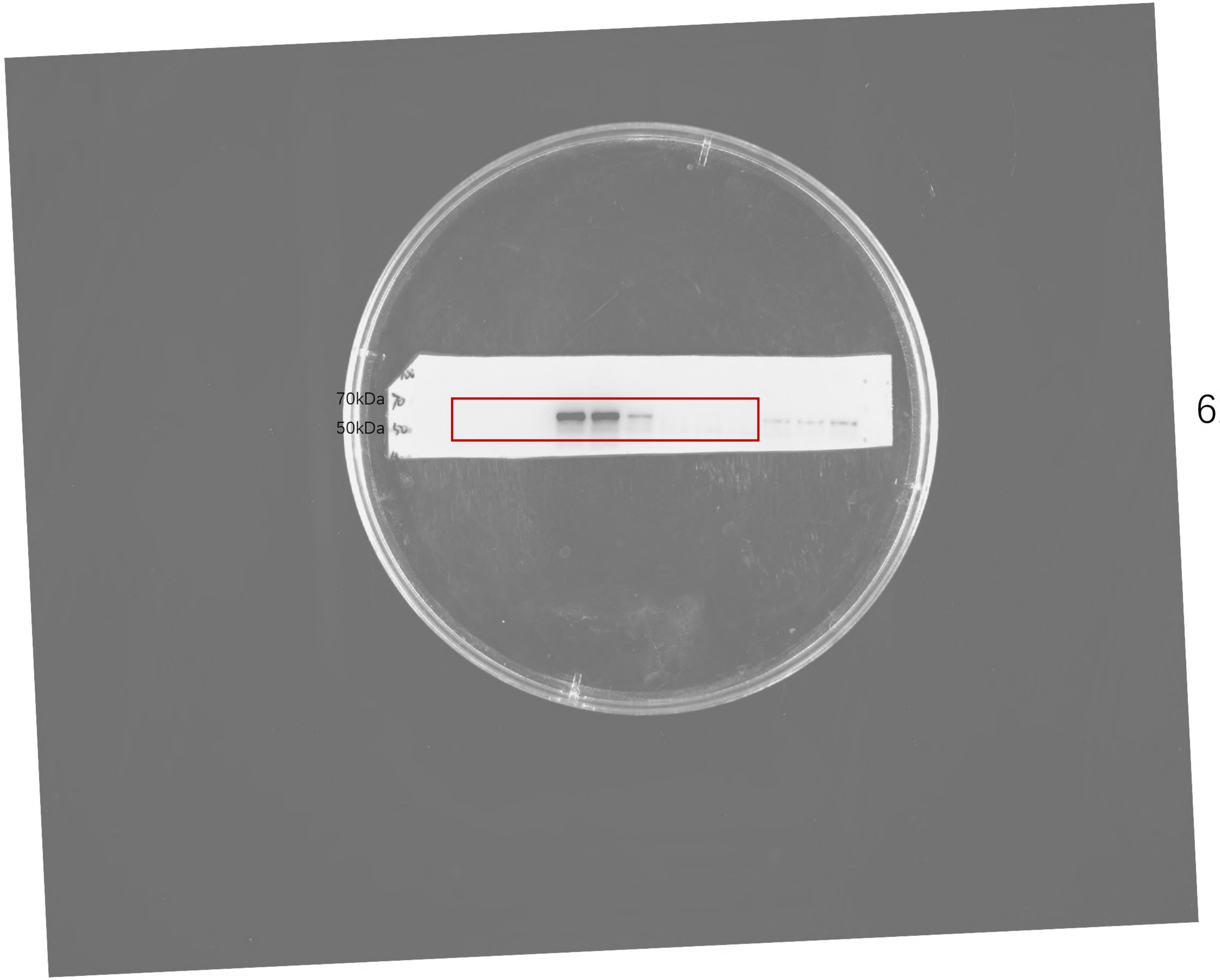

62kDa

Source Fig.7F KRT10

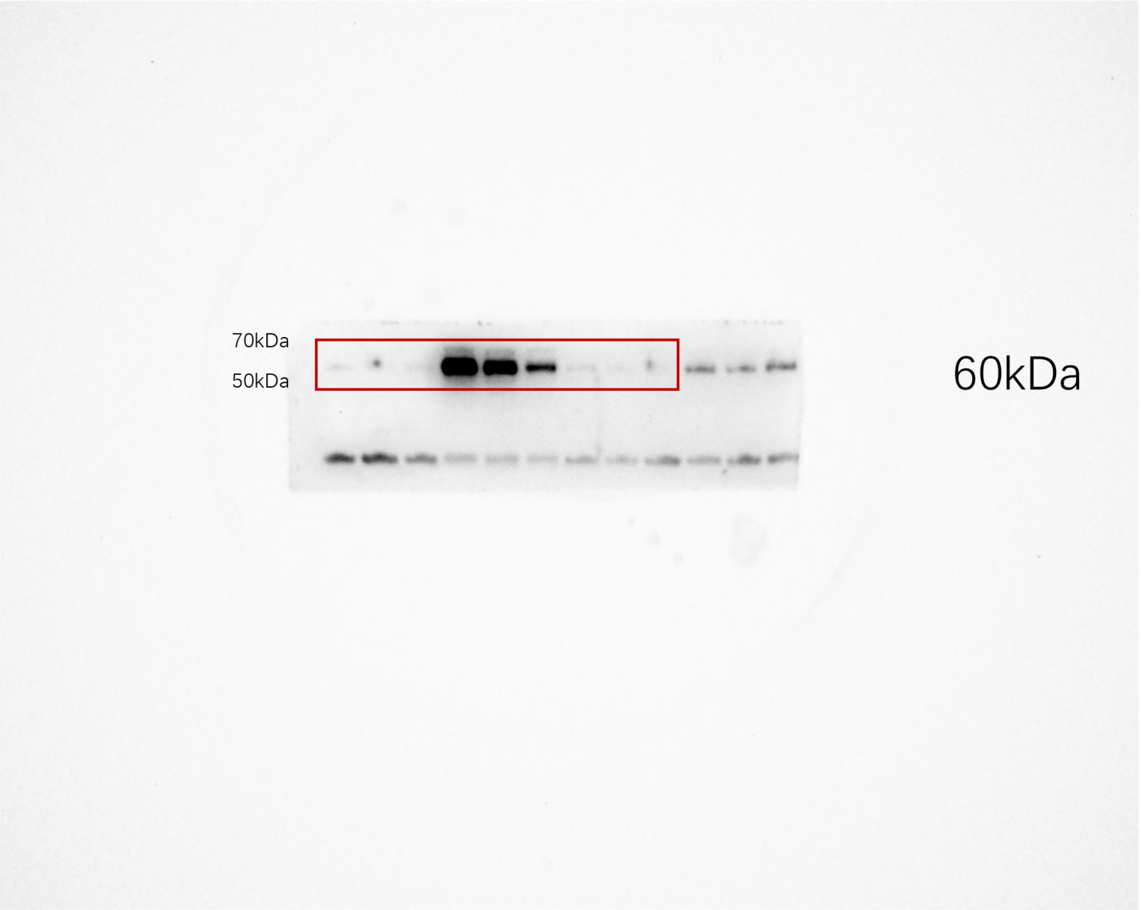

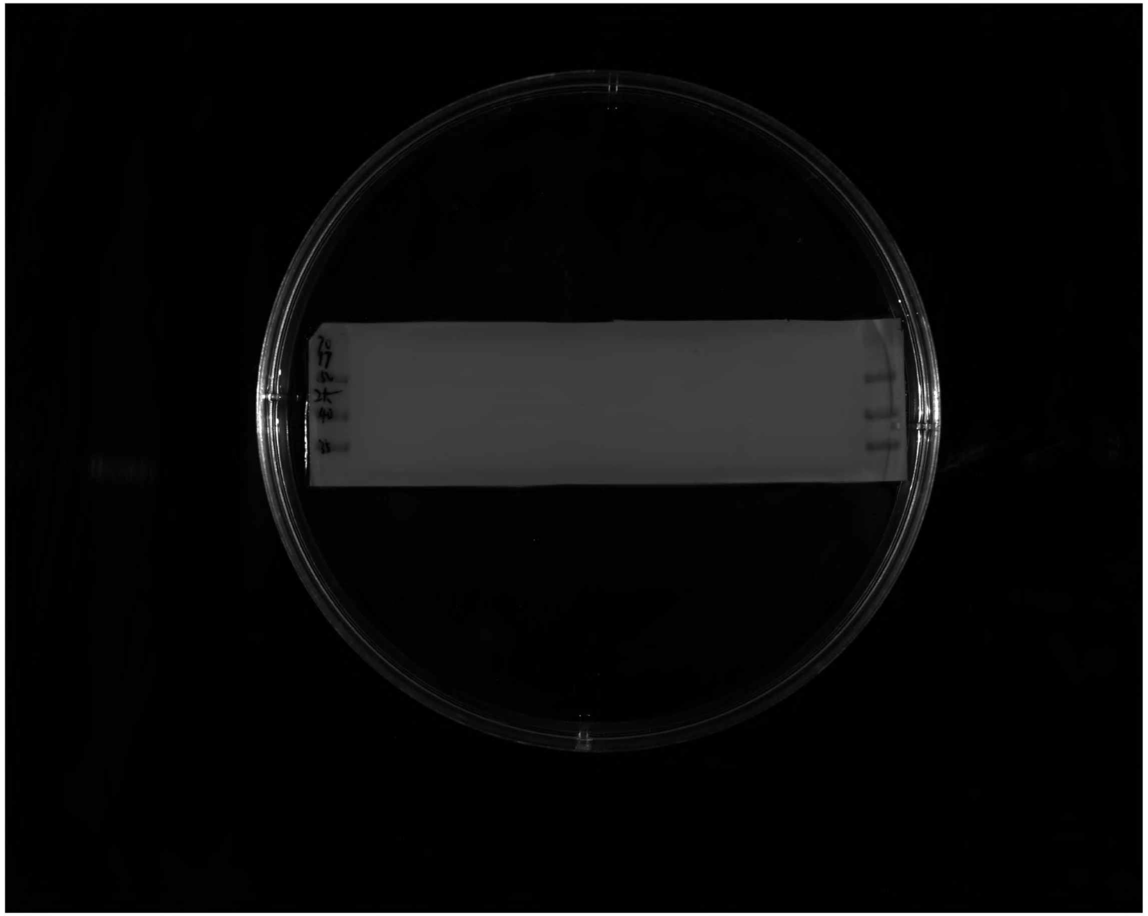

Source Fig.7F KRT14

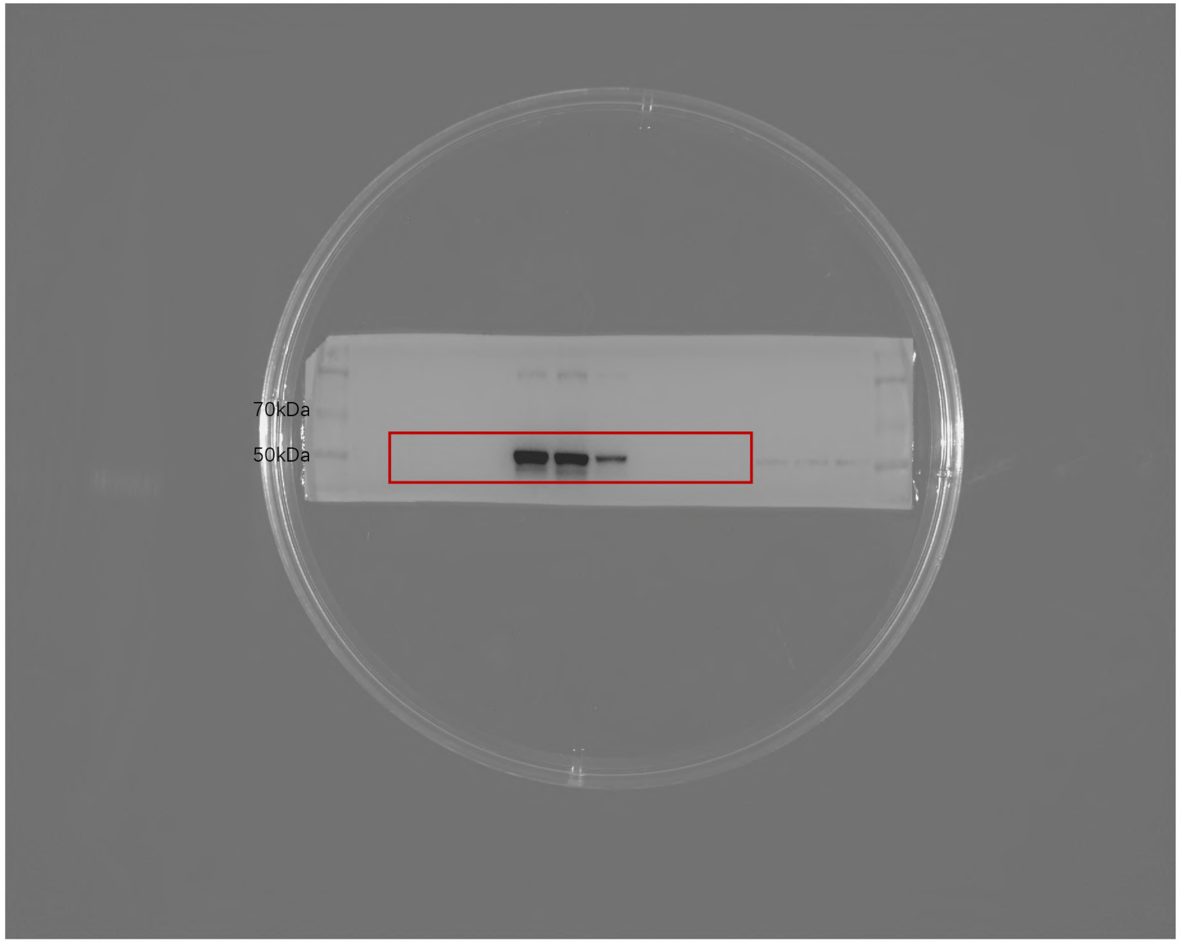

53kDa

Source Fig.7F KRT17

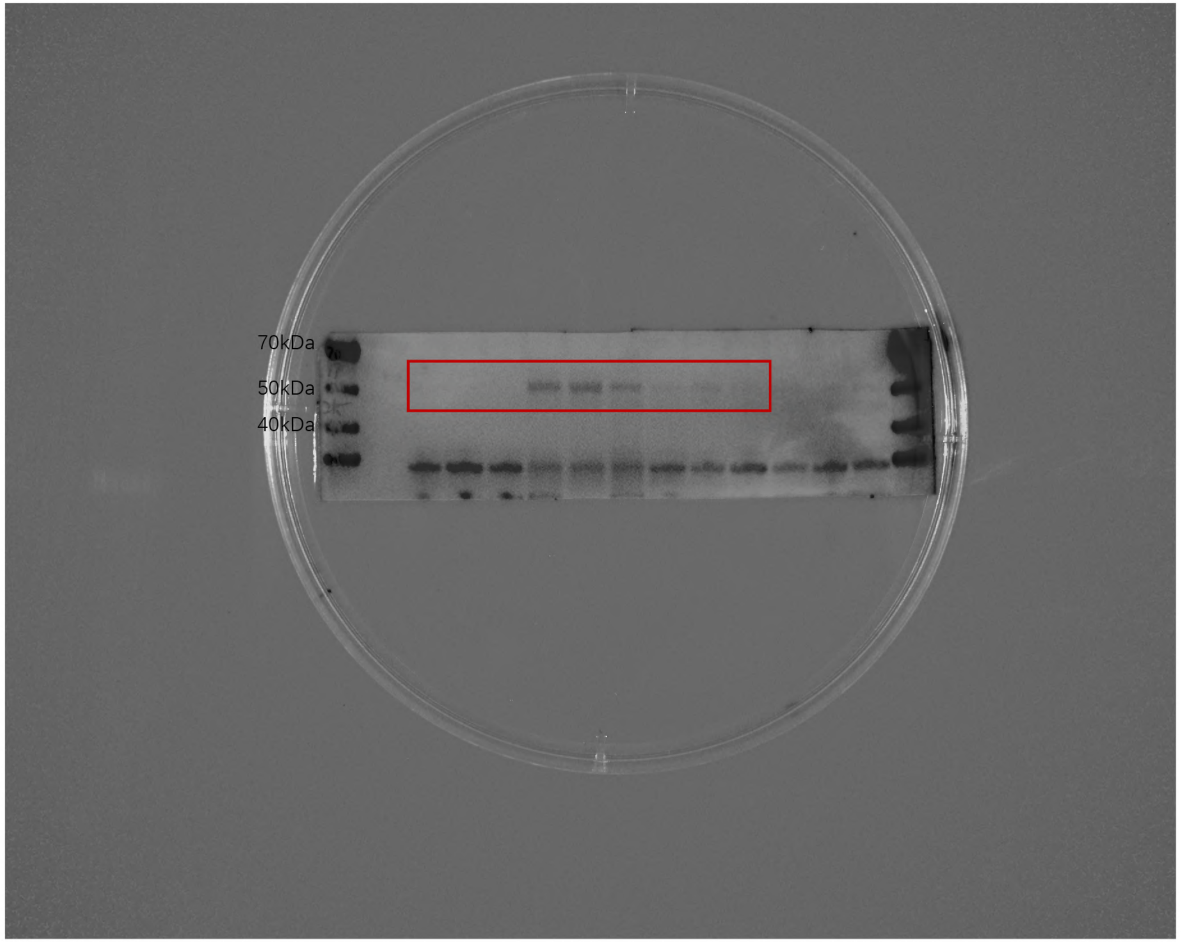

48kDa

Source Fig.7F Loricrin

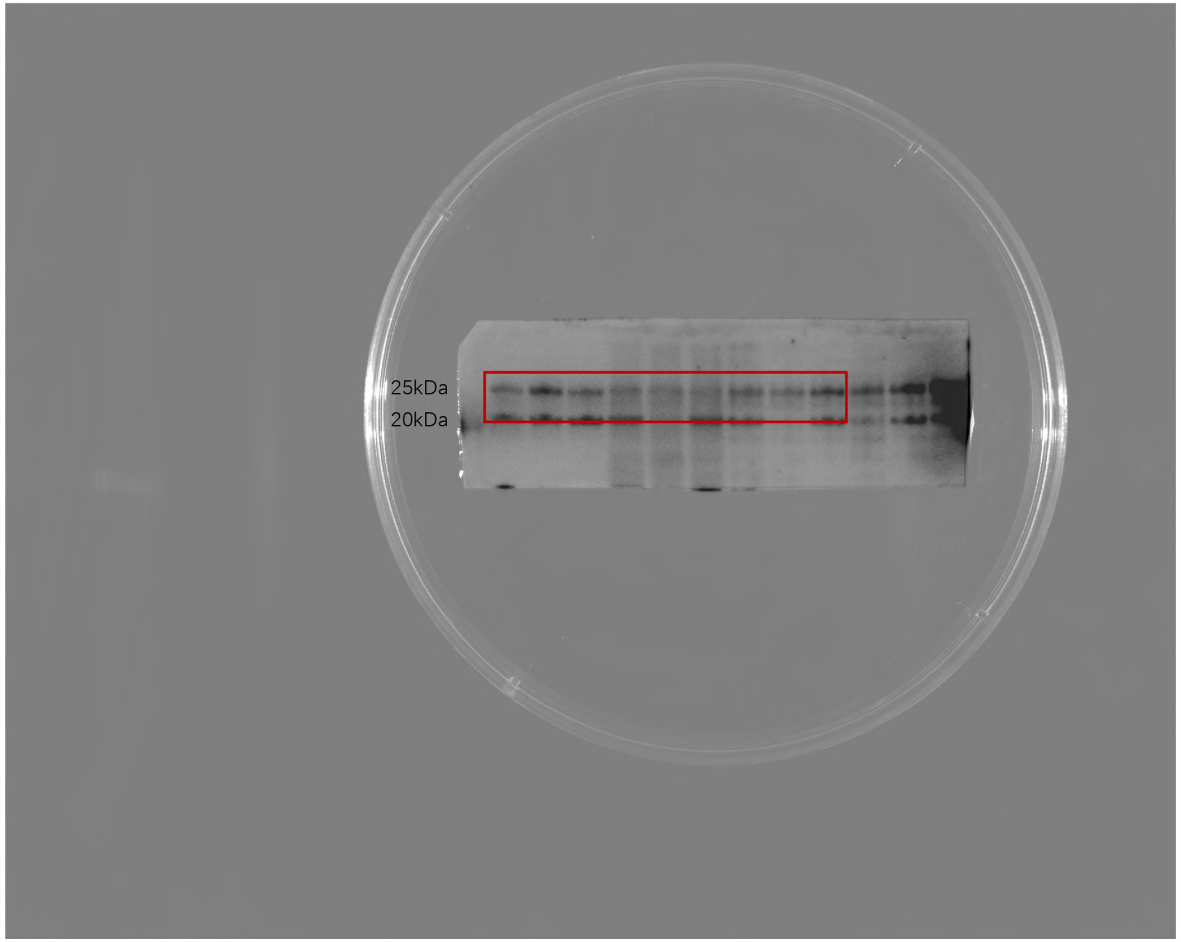

26kDa

Source Fig.7F Fn14

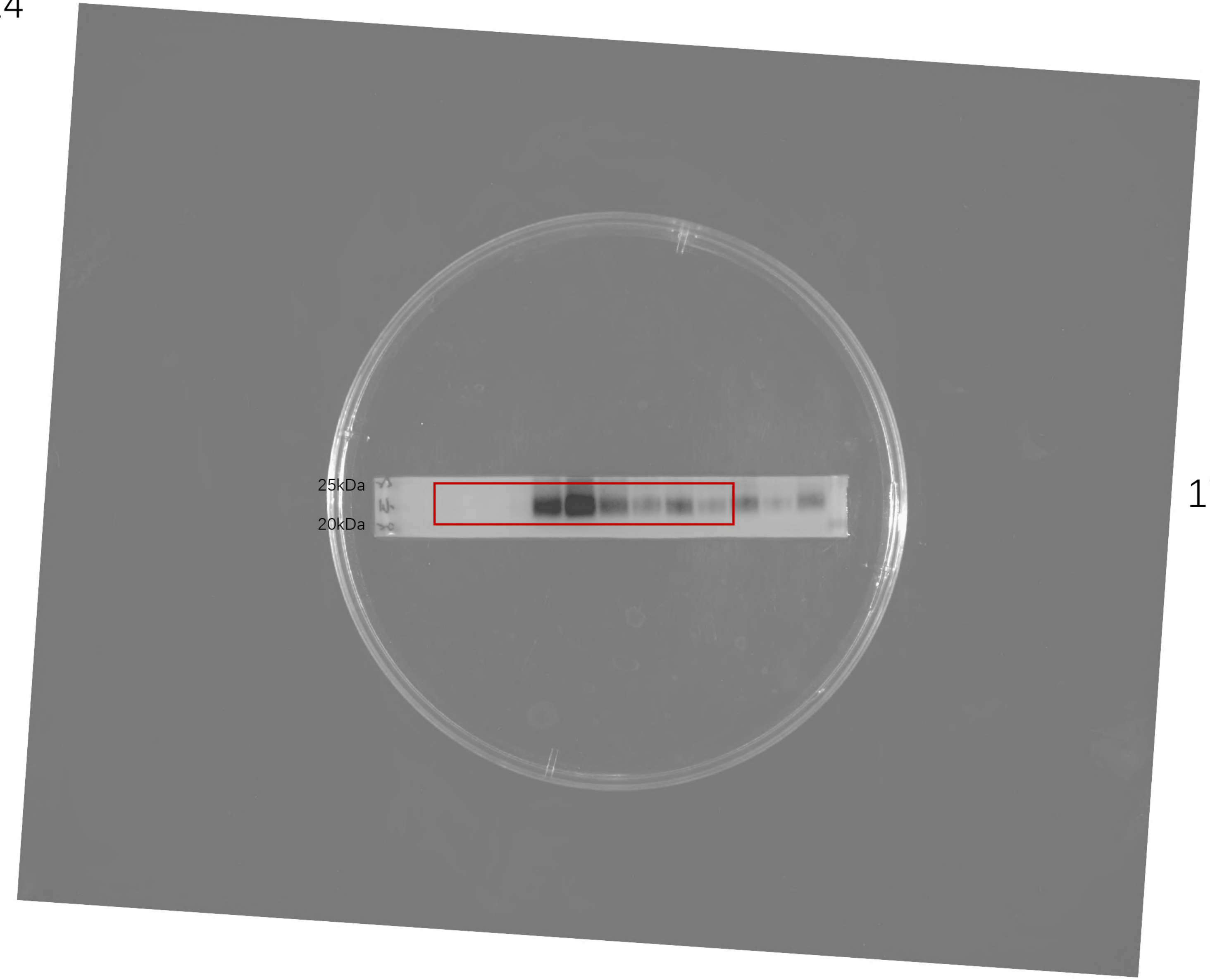

17kDa

Source Fig.7F GAPDH

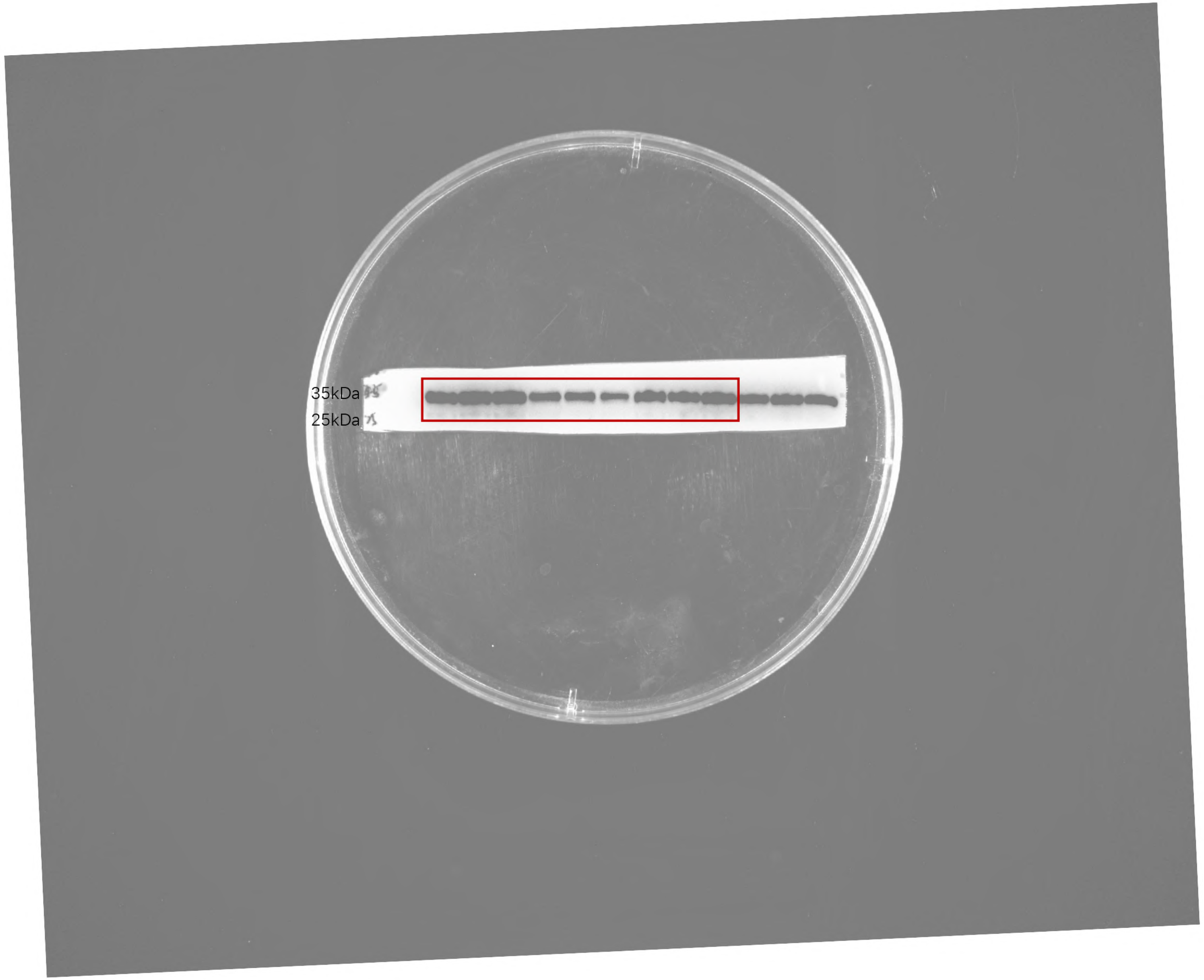

37kDa

Source Fig.7F KRT6

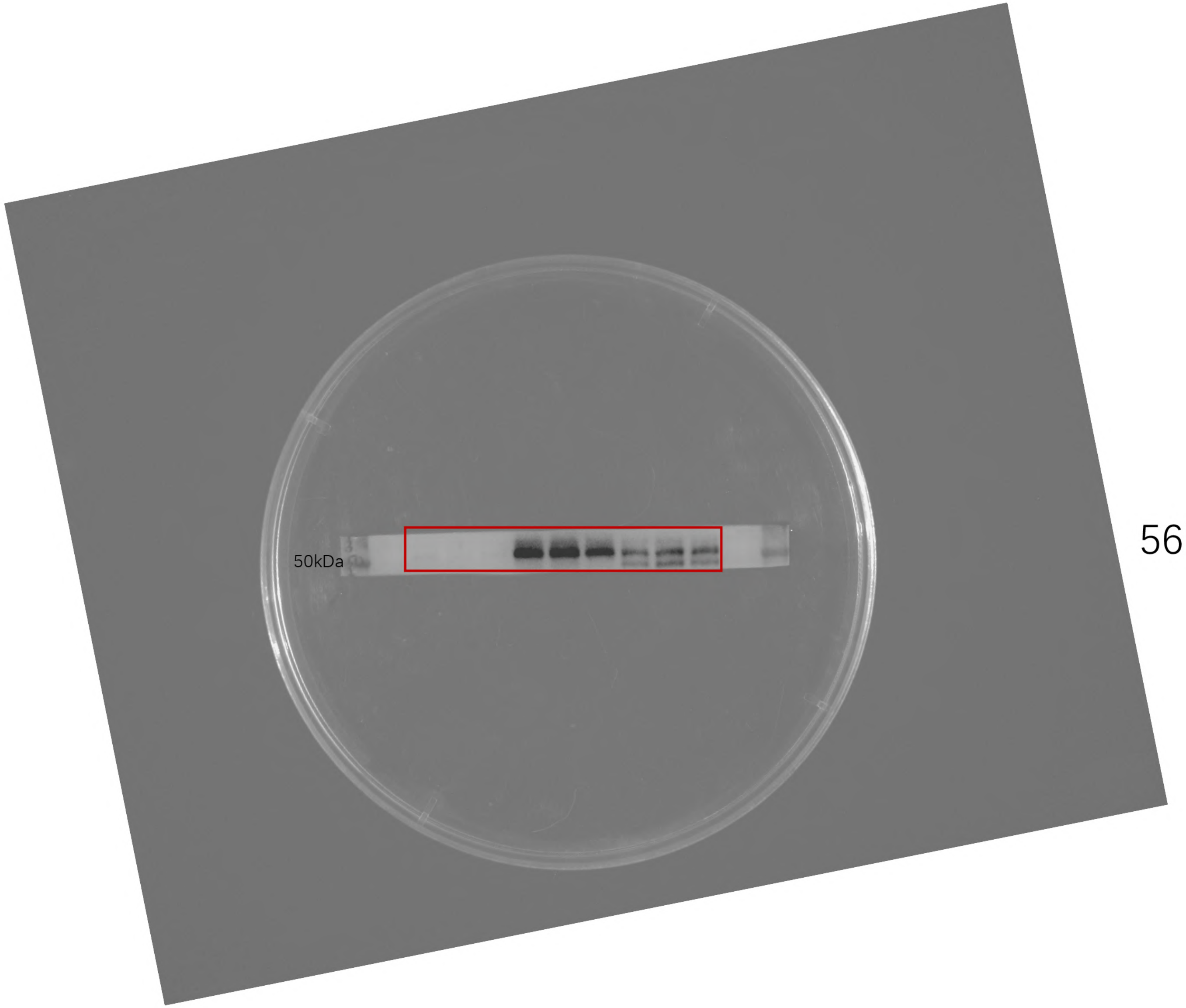

56kDa

Source Fig.7F KRT16

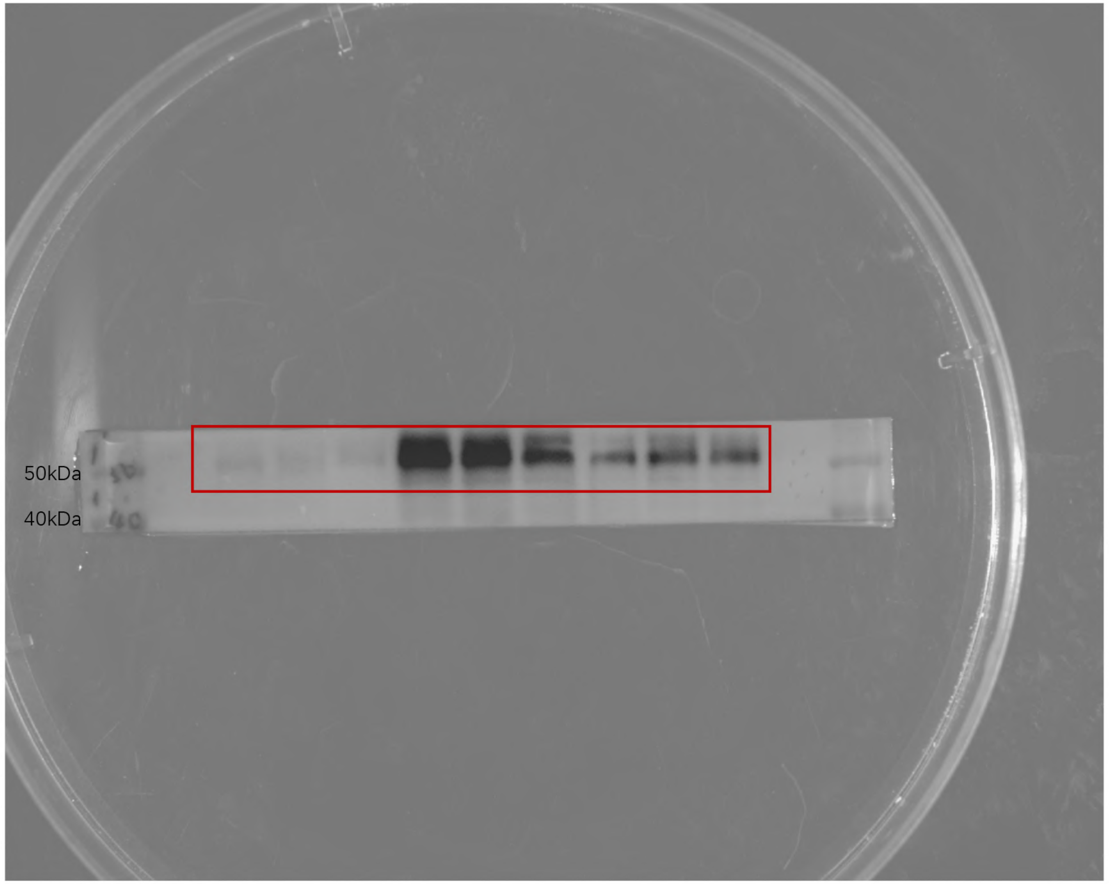

48kDa

Source Fig.7F CXCL10

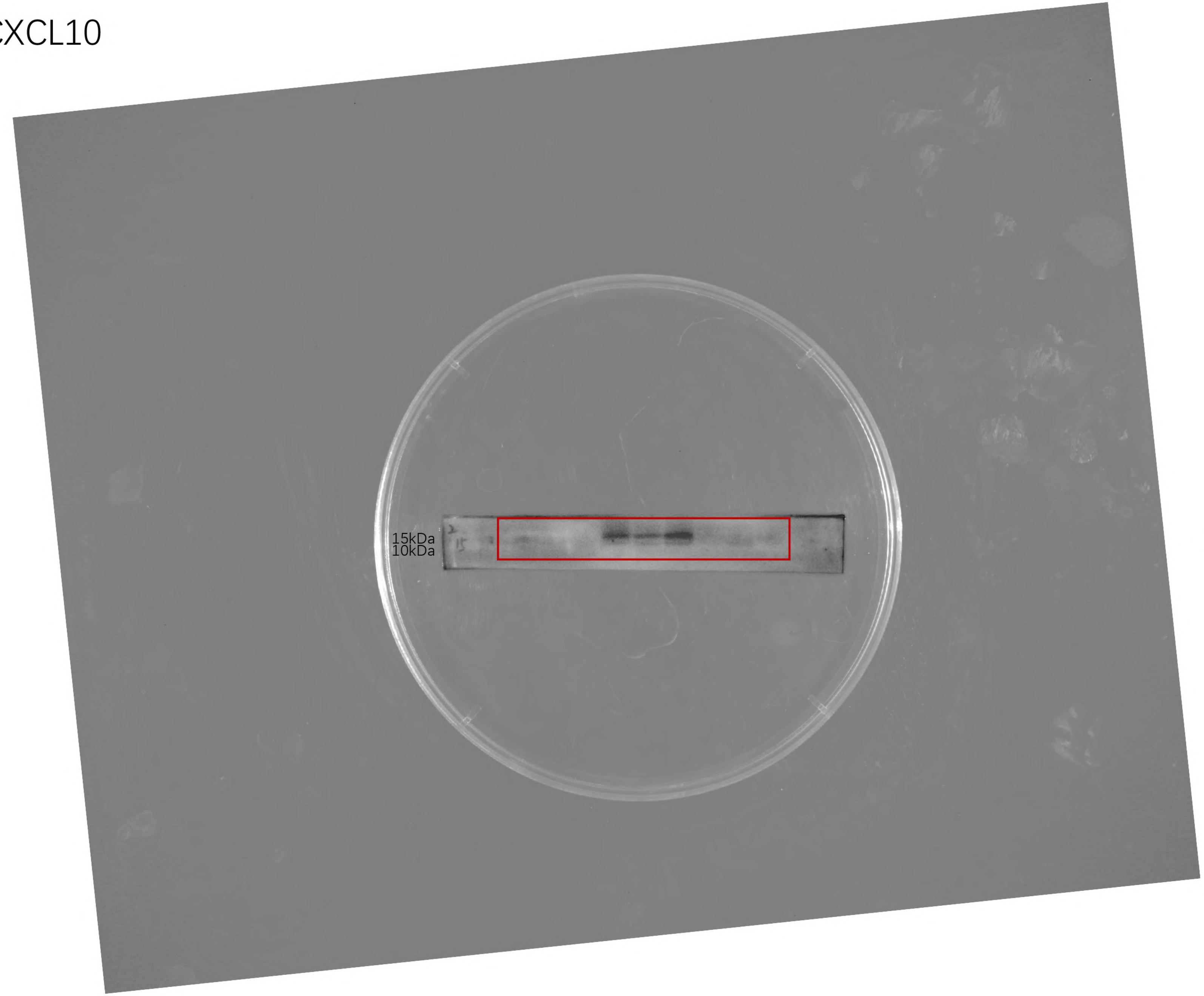

10kDa

Source Fig.7F p-ERK1/2

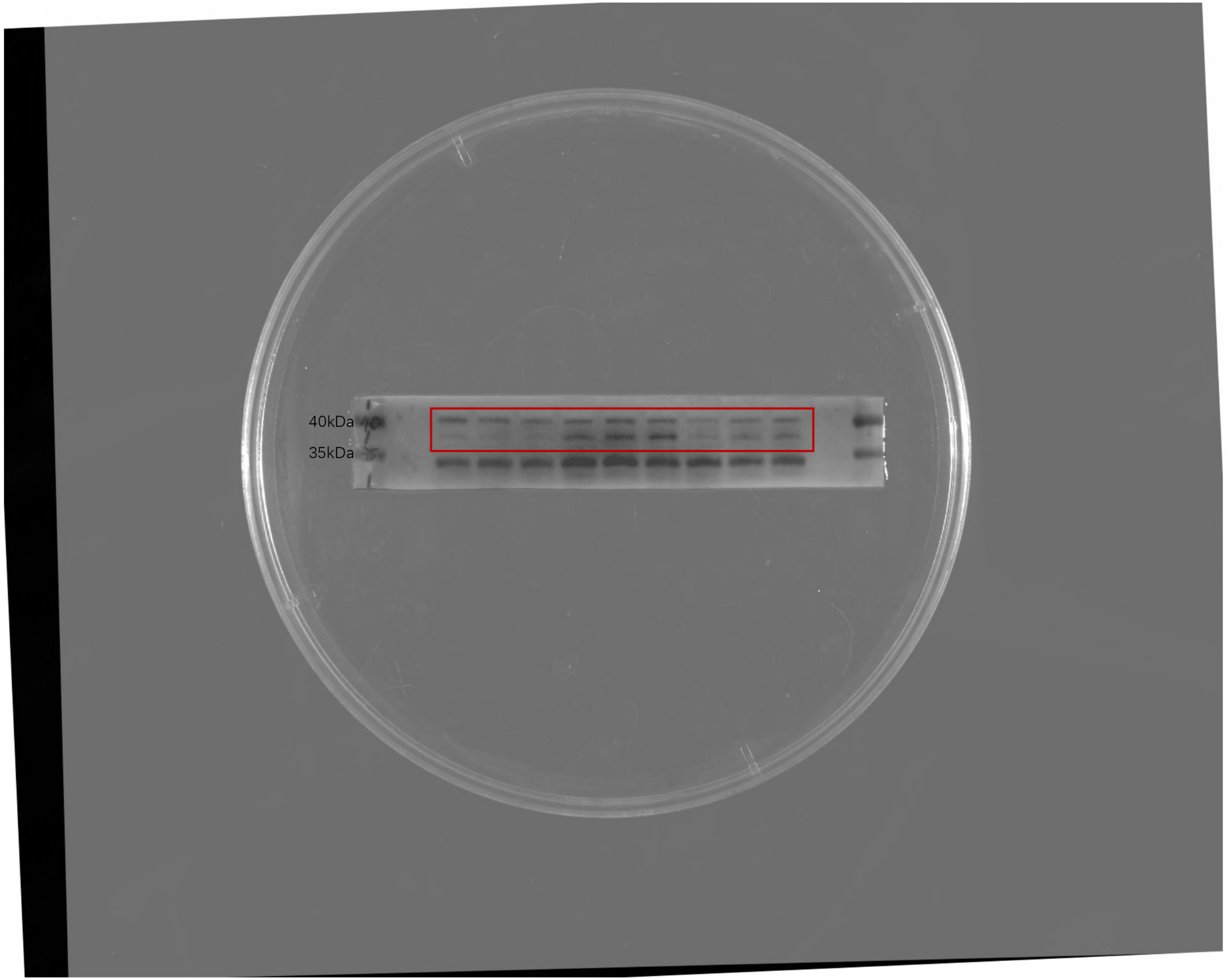

44kDa
42kDa

Source Fig.7F ERK1/2

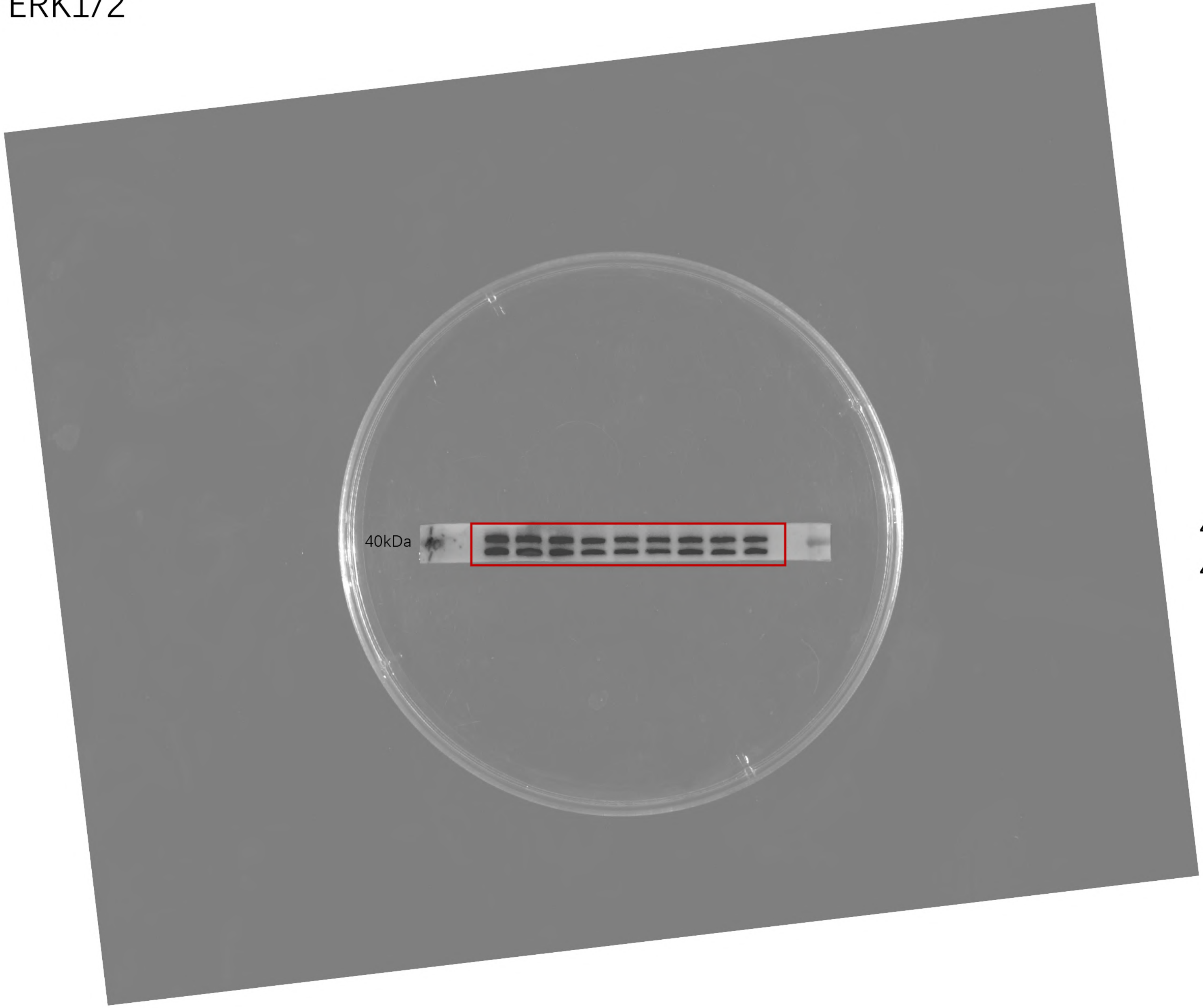

44kDa
42kDa

Source Fig.7F GAPDH

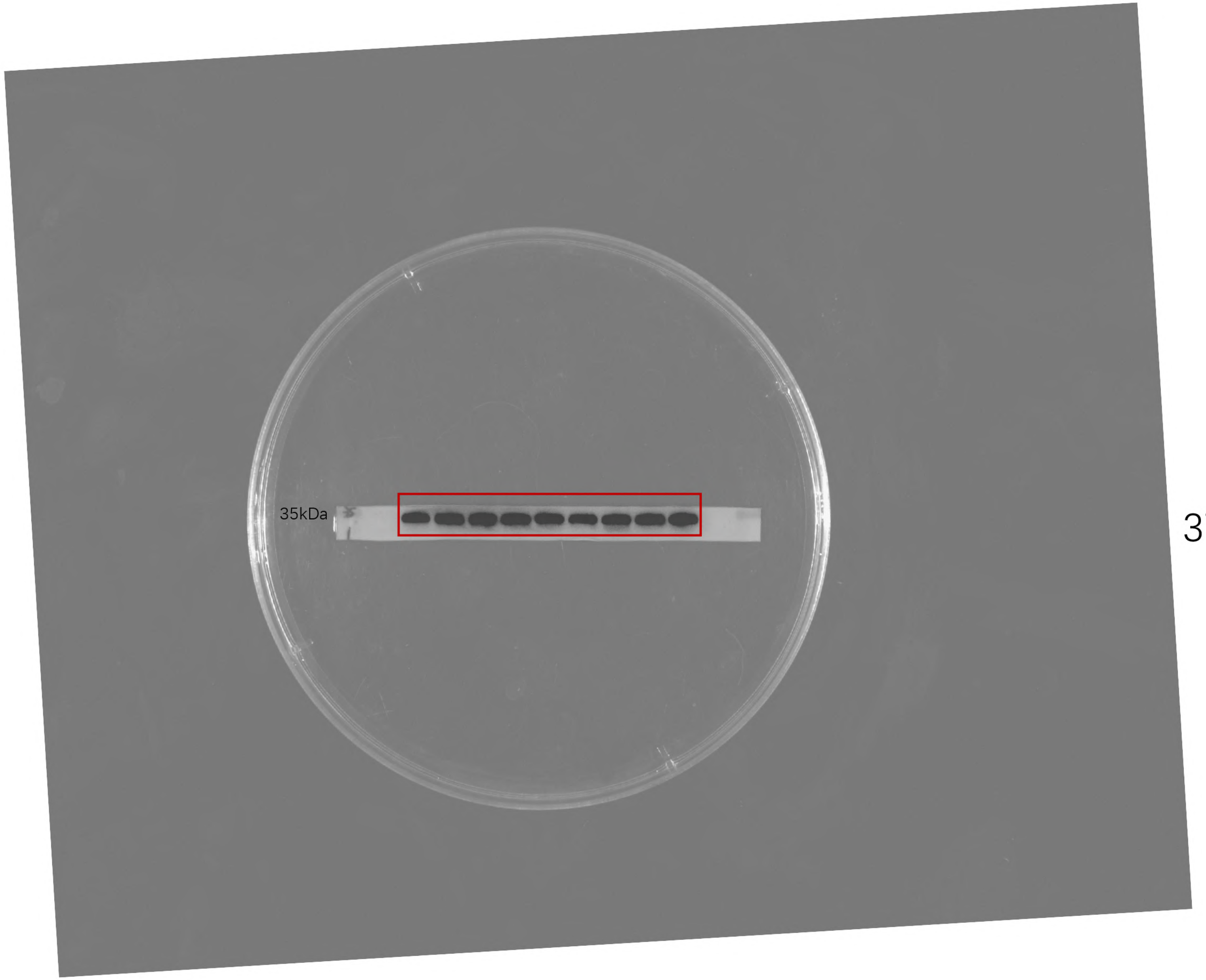

37kDa

Source Fig.7F p-p38

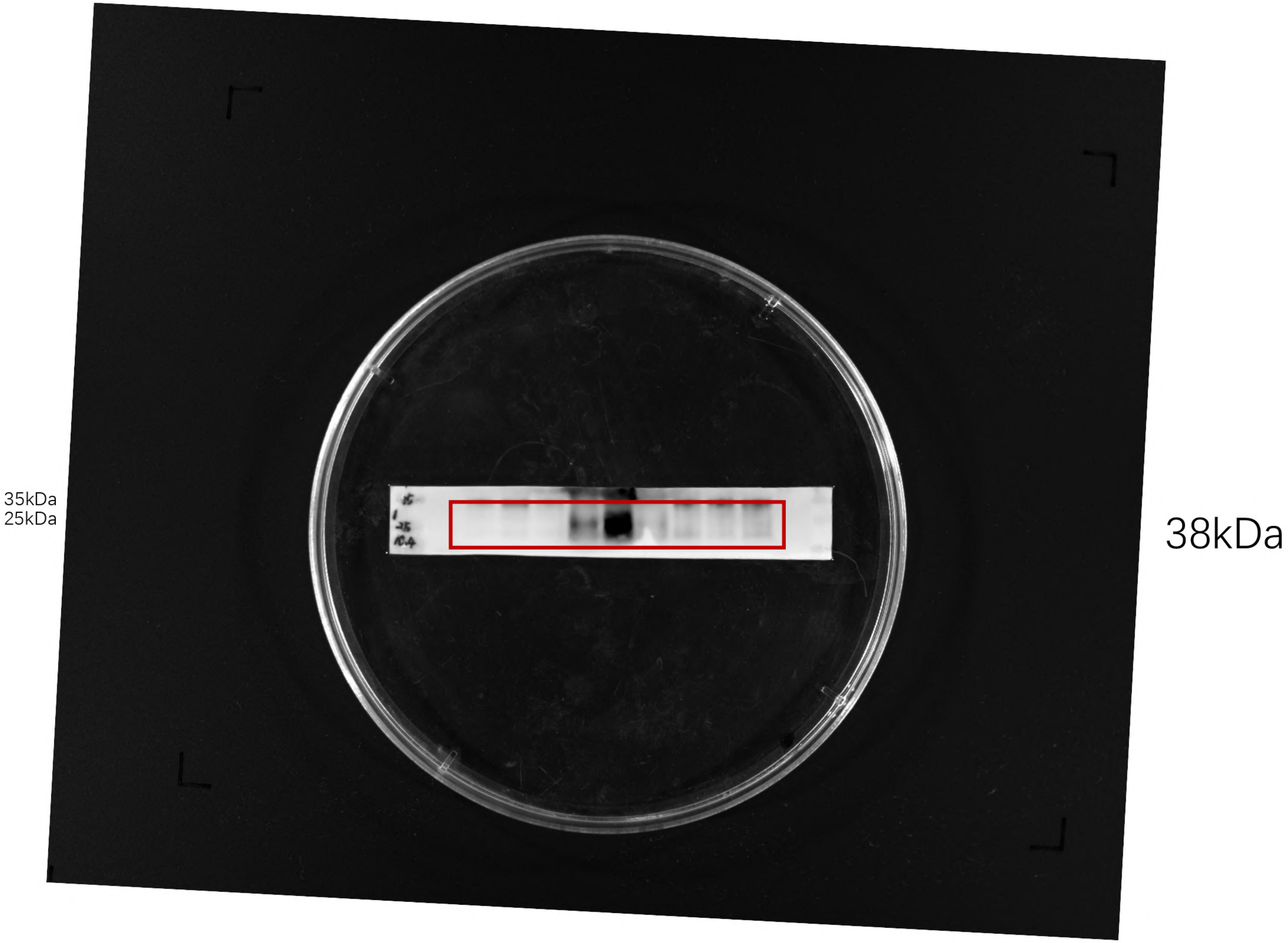

Source Fig.7F p38

35kDa
25kDa

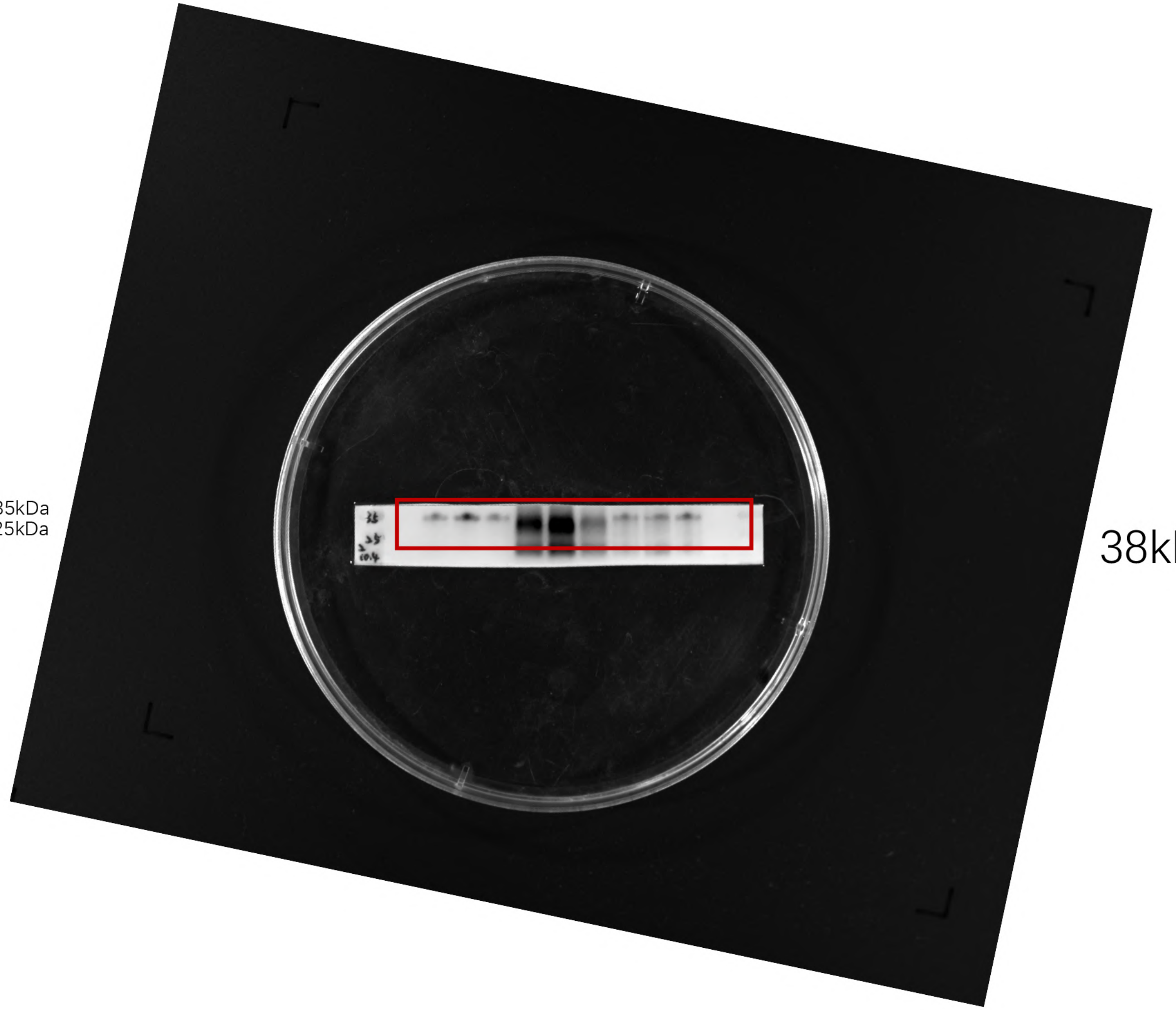

38kDa

Source Fig.7F p-JNK

70kDa
50kDa

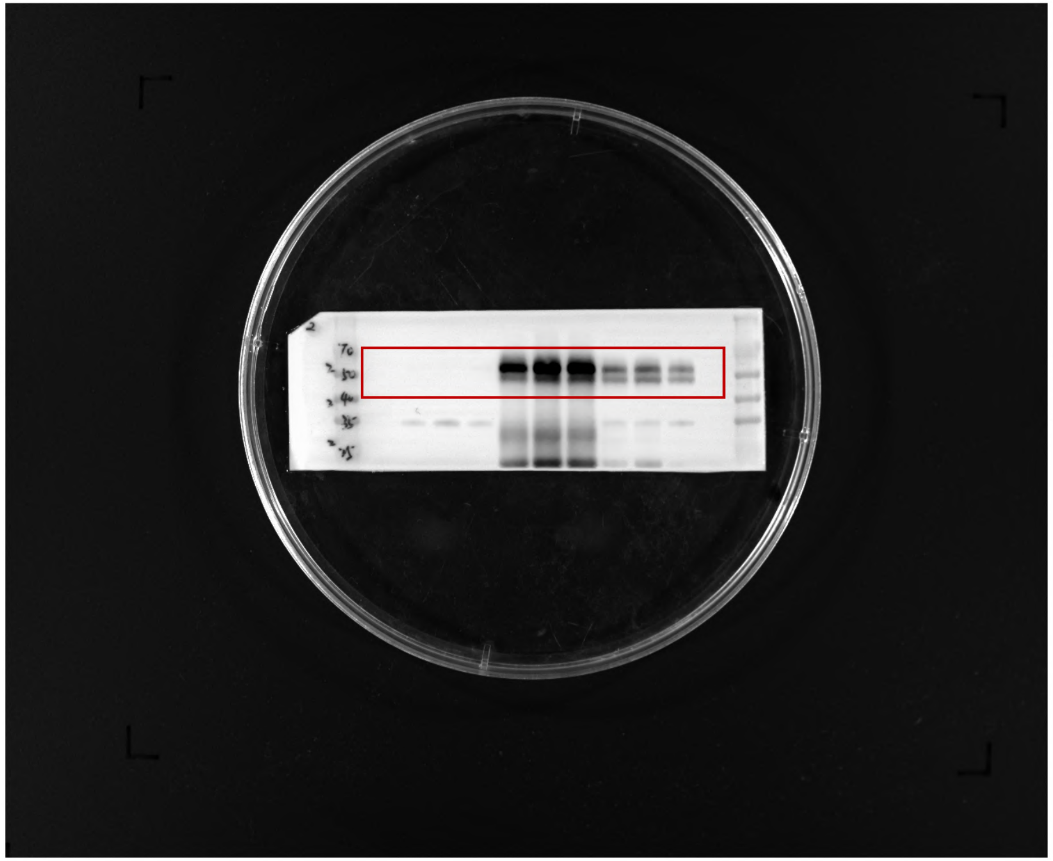

54kDa
46kDa

Source Fig.7F JNK

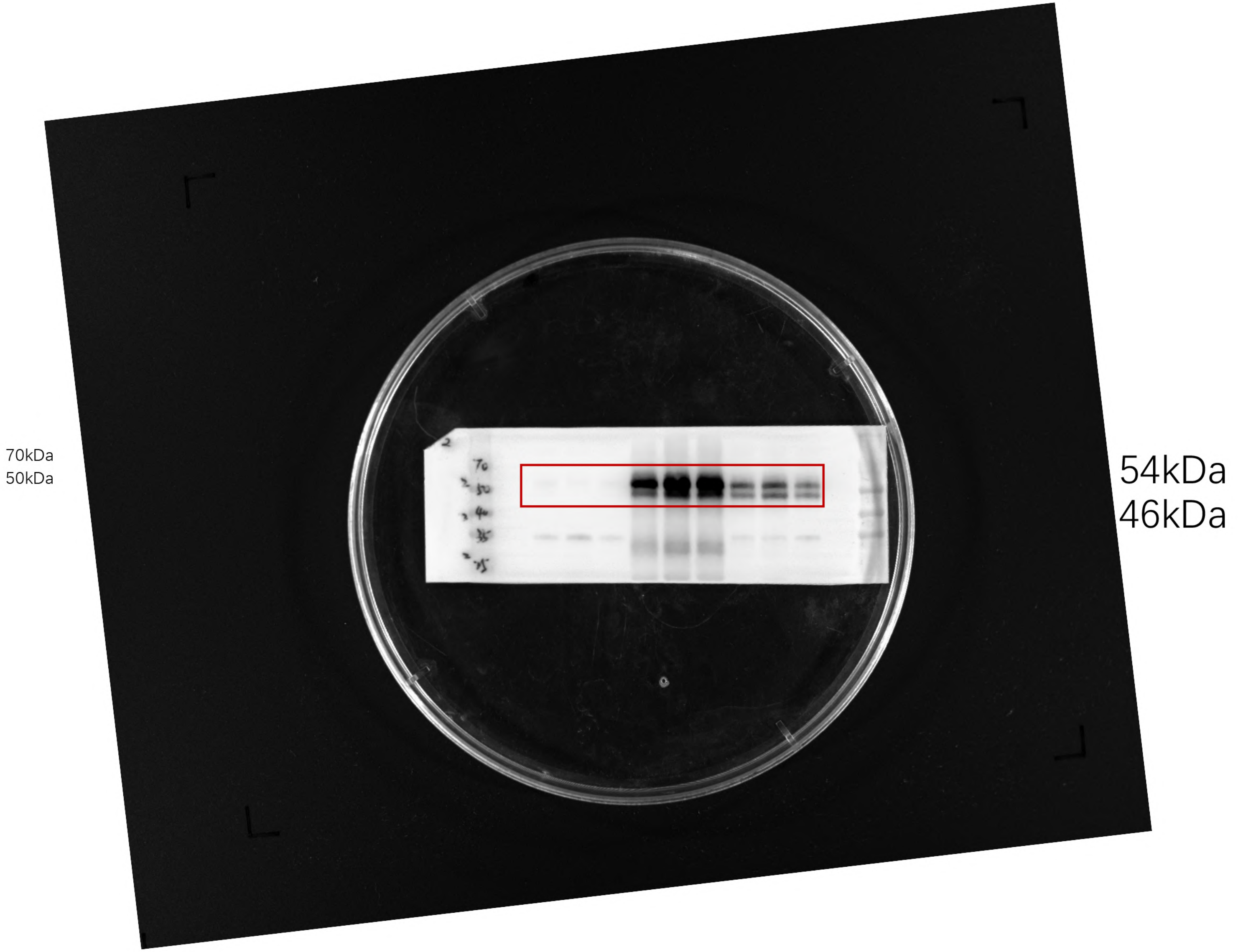

Source Fig.8A KRT6

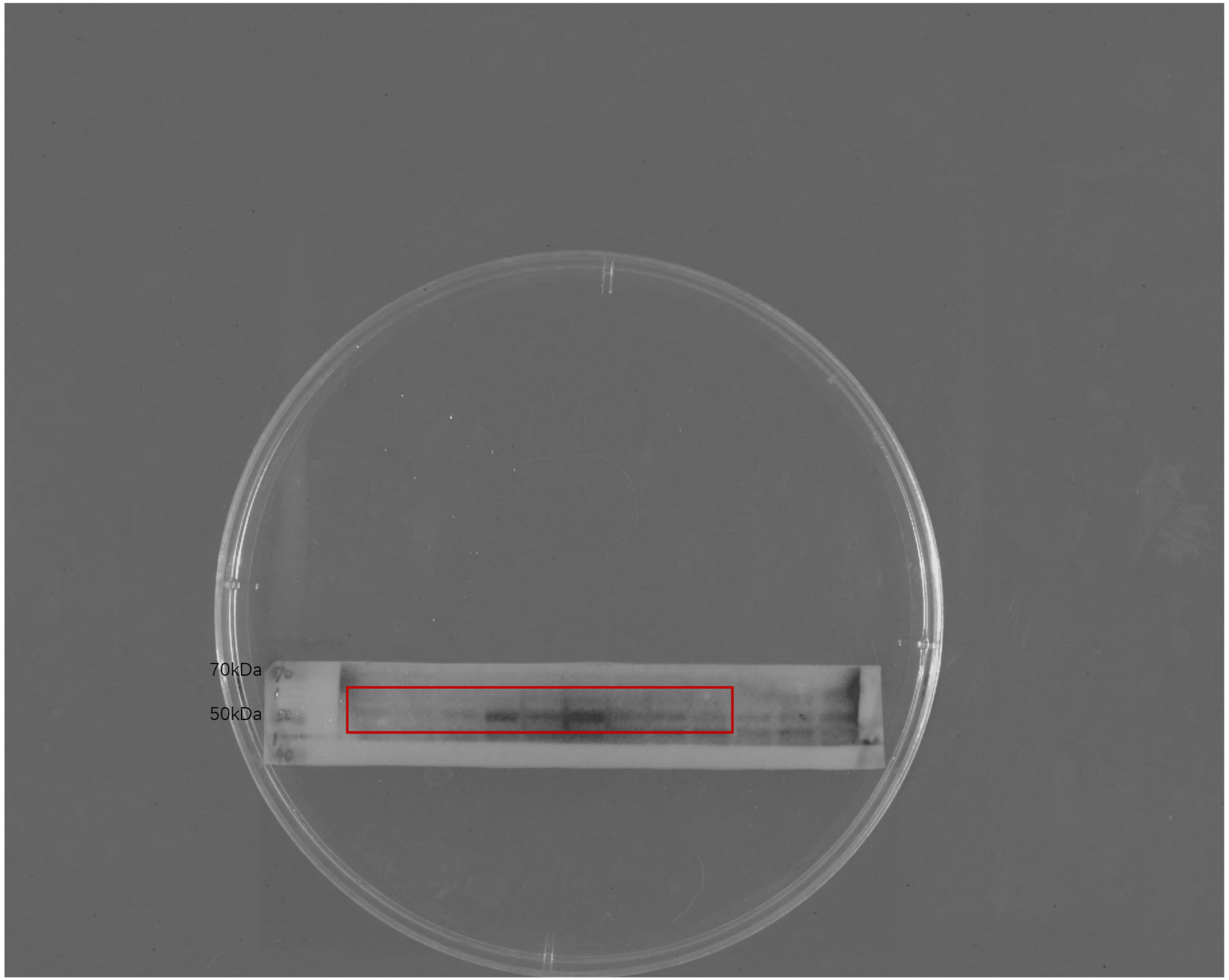

56kDa

Source Fig.8A GAPDH

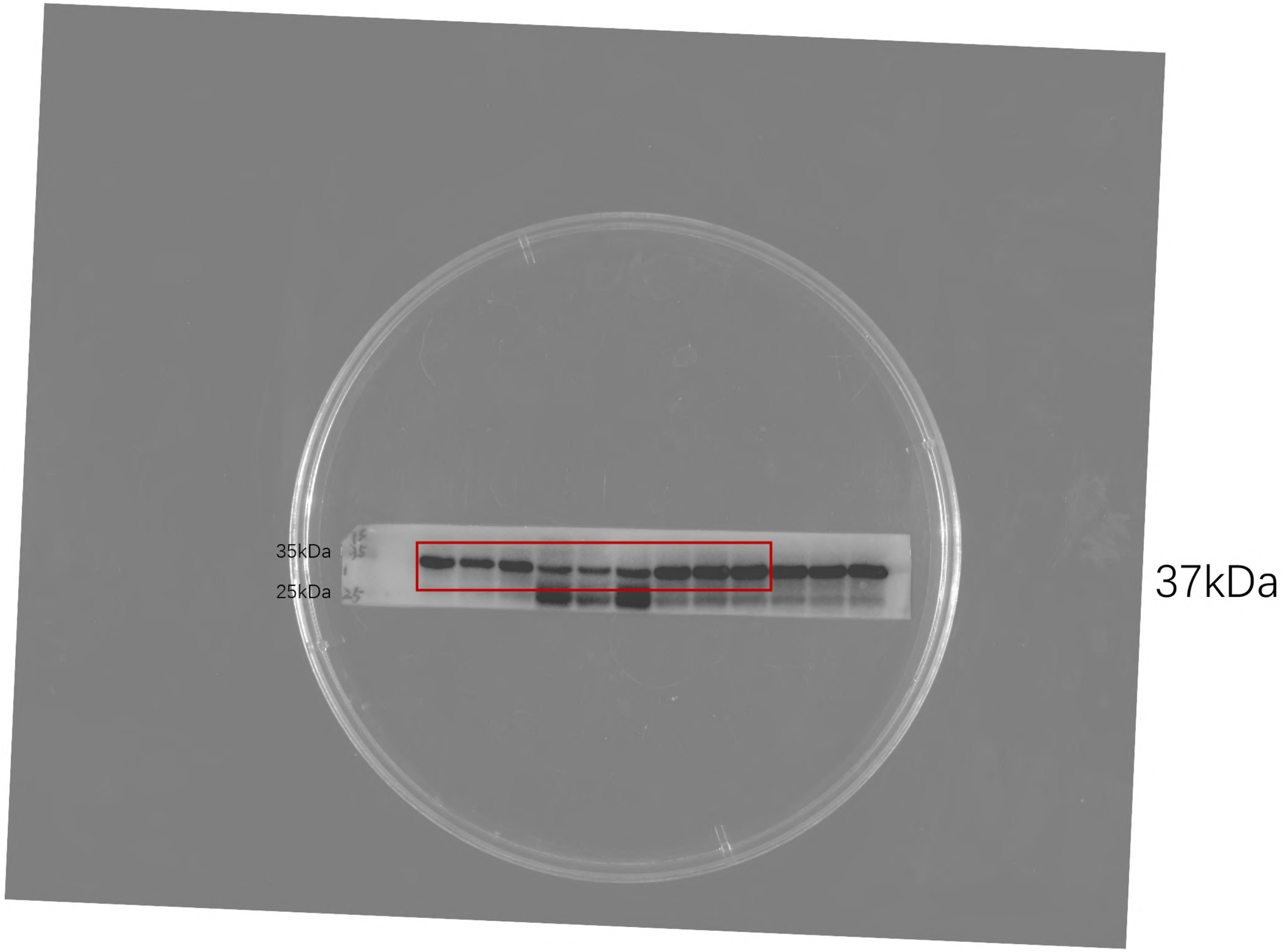

Source Fig.8 KRT16

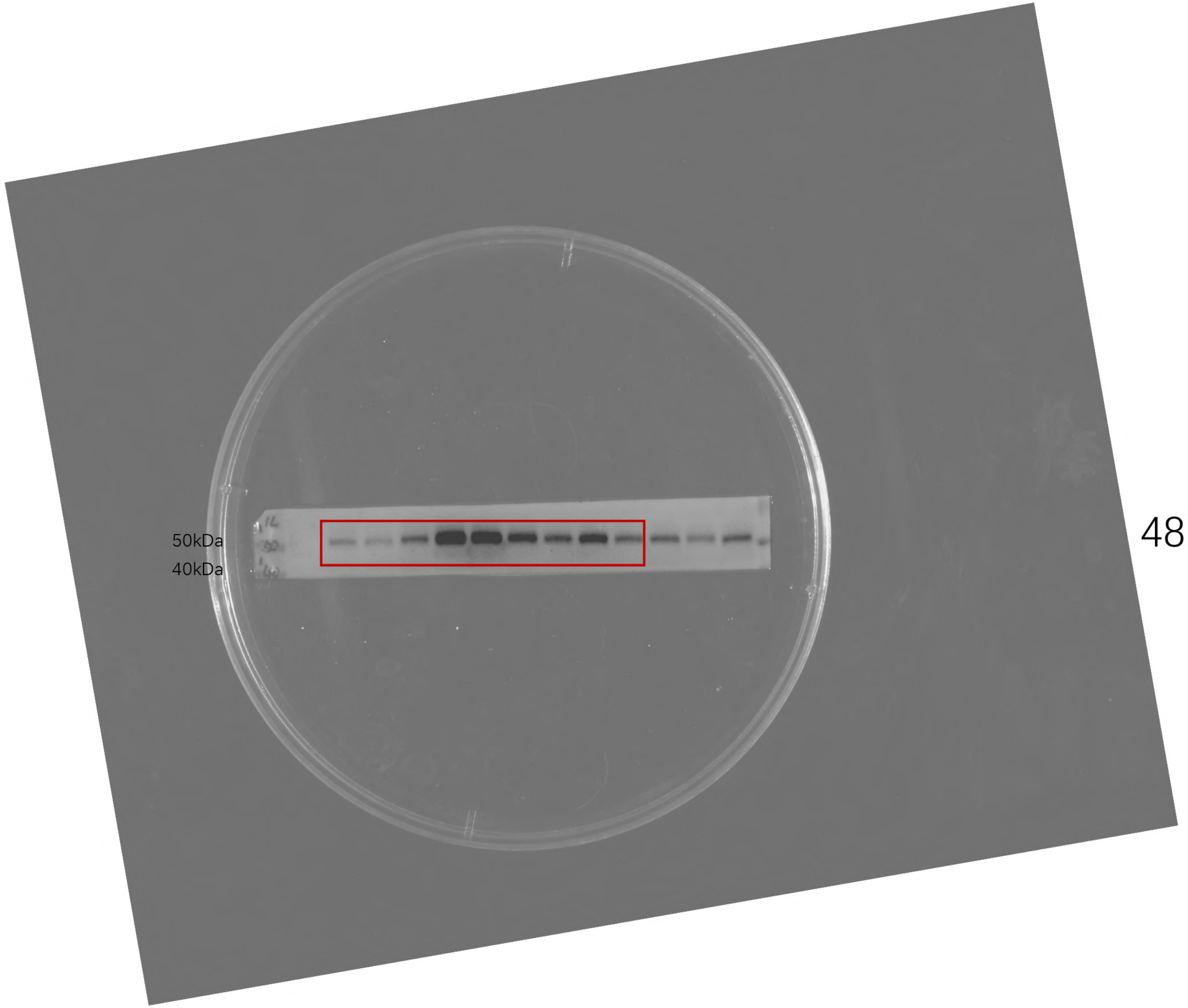

Source Fig.8A LCN2

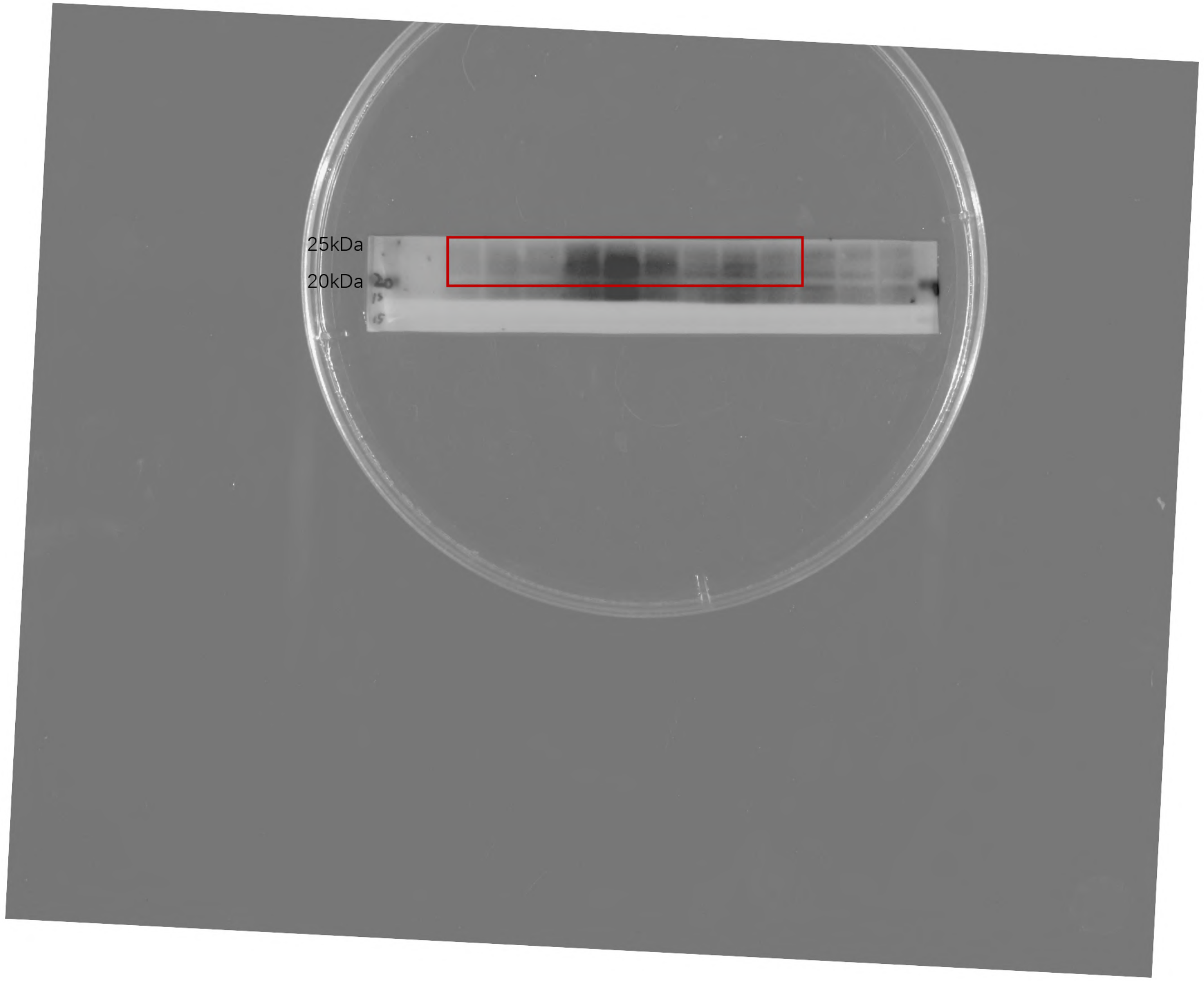

21kDa

Source Fig.8A GAPDH

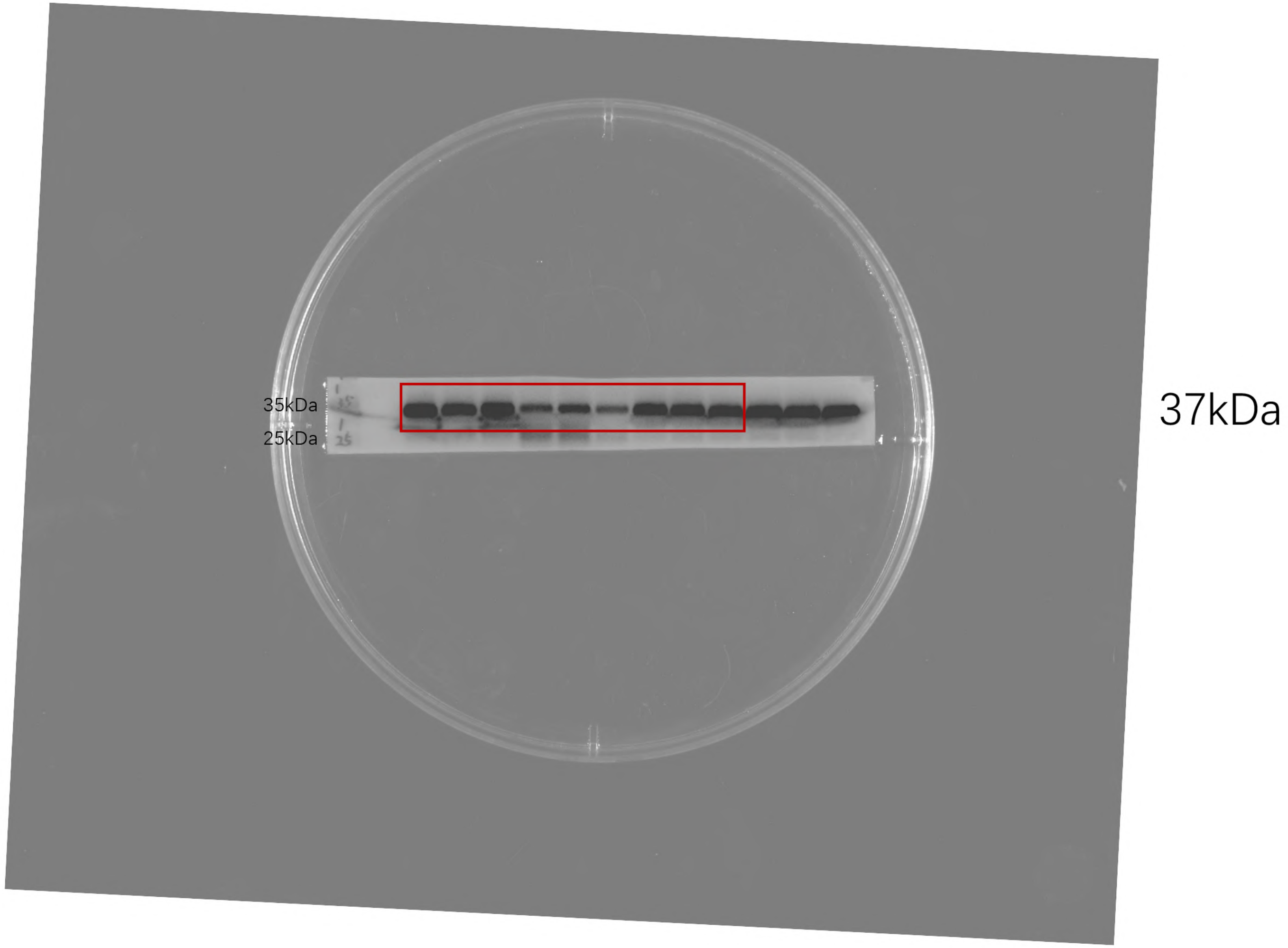

Source Fig.8C p-ERK1/2

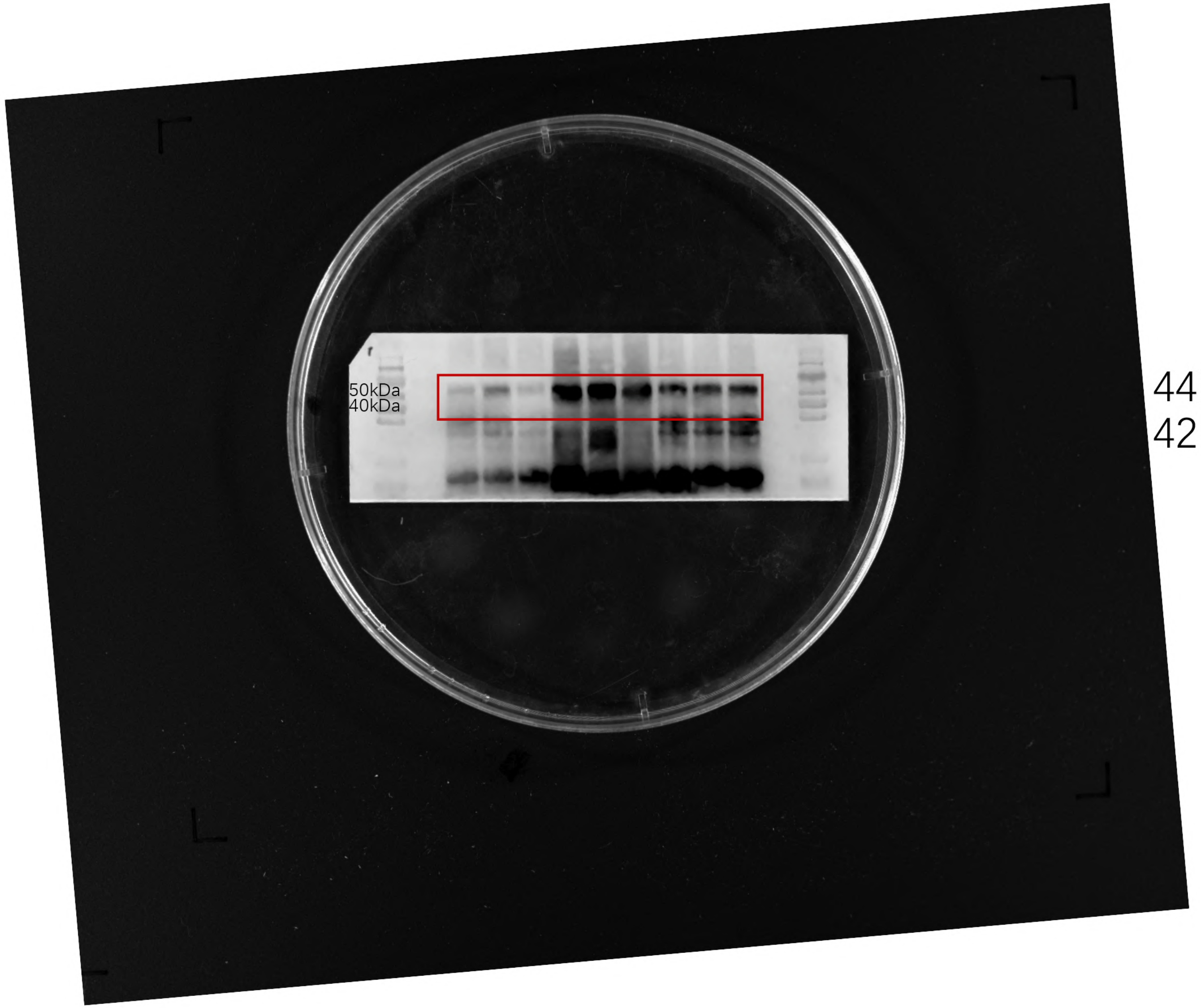

44kDa
42kDa

Source Fig.8C ERK1/2

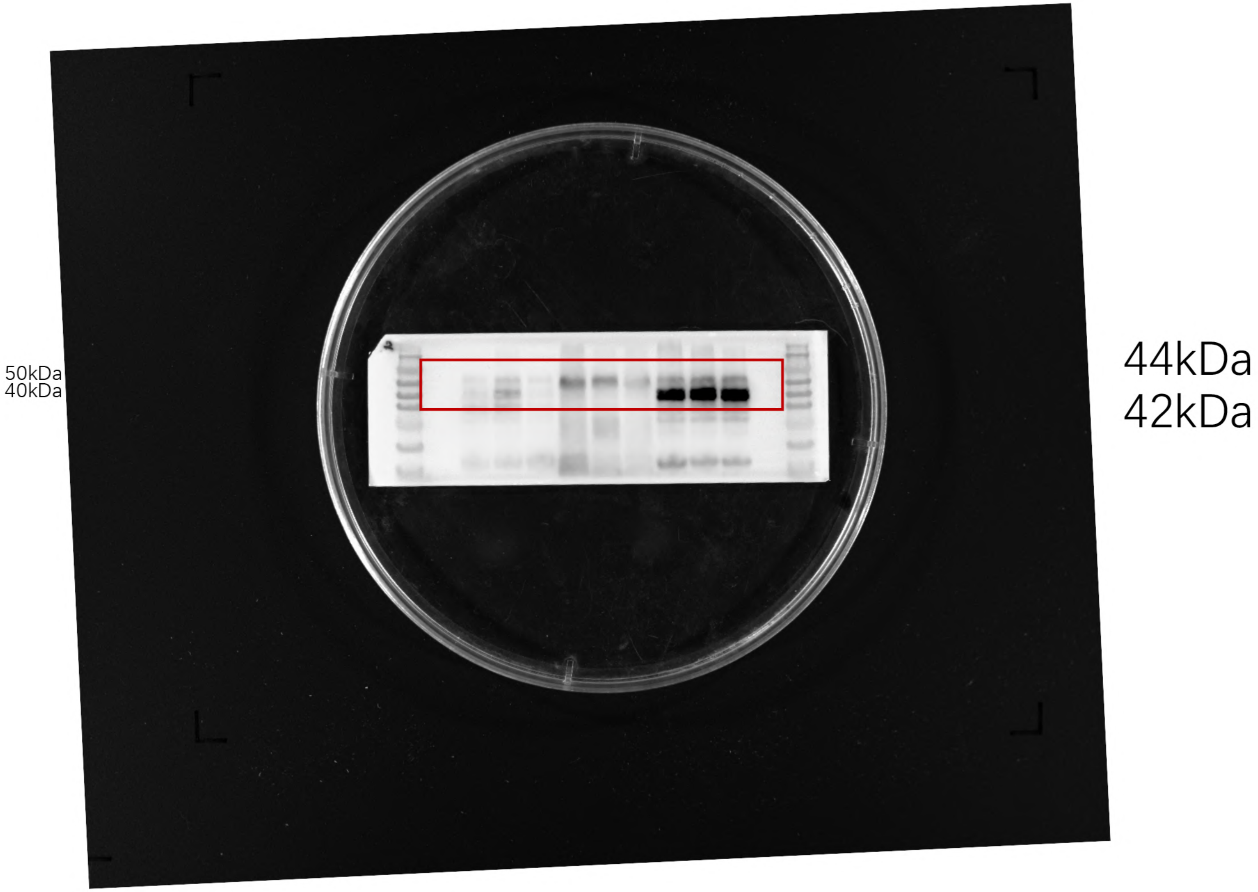

Source Fig.8C p-JNK

70kDa
50kDa

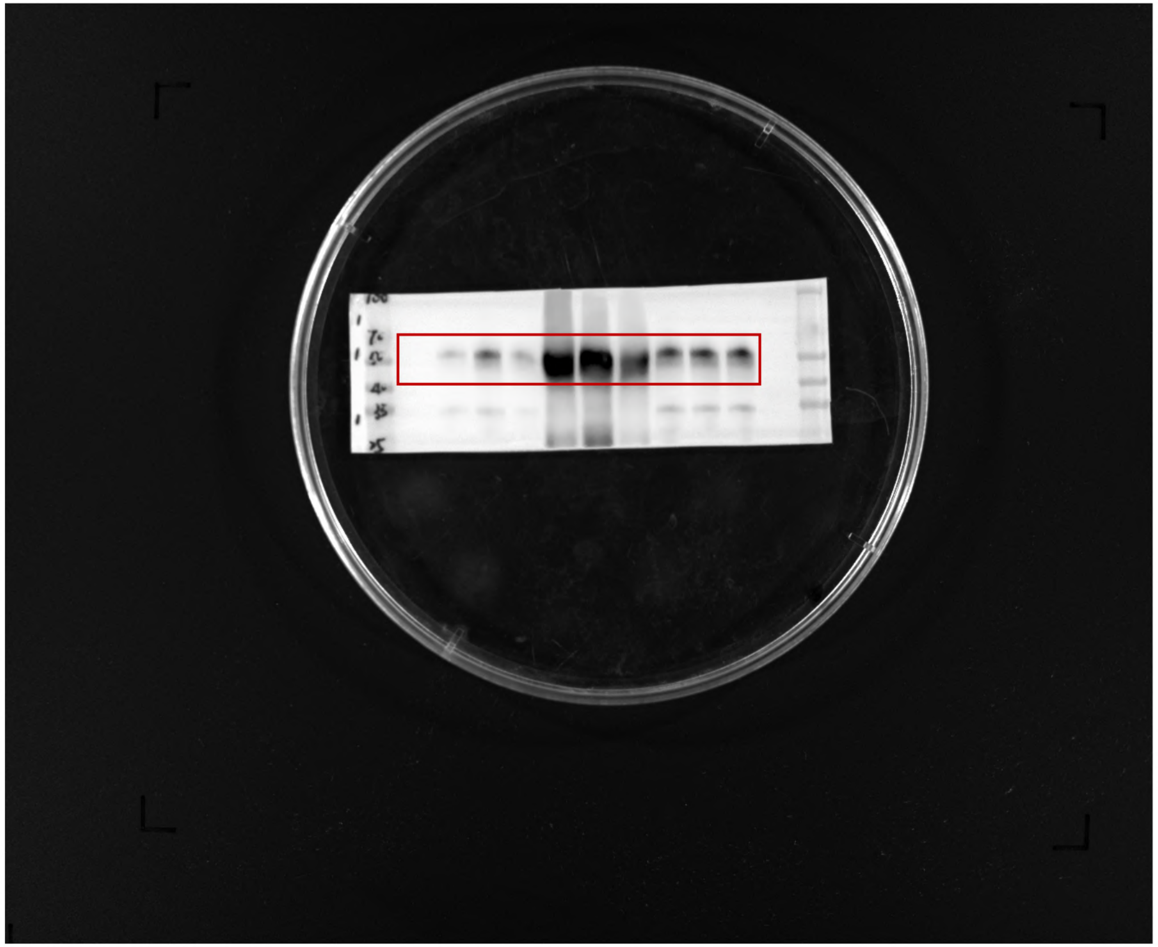

54kDa
46kDa

Source Fig.8C JNK

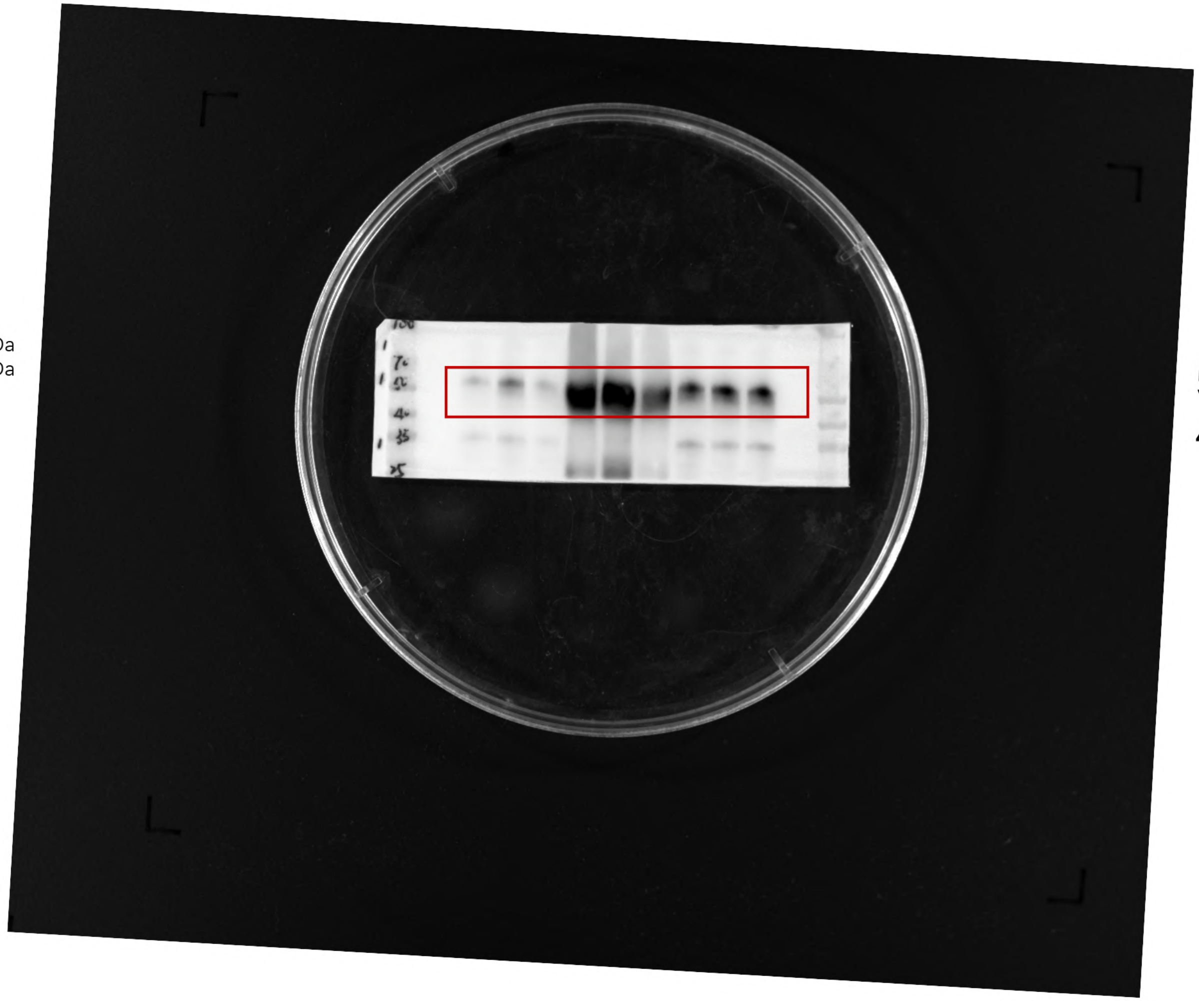

Source Fig.8C p-p38

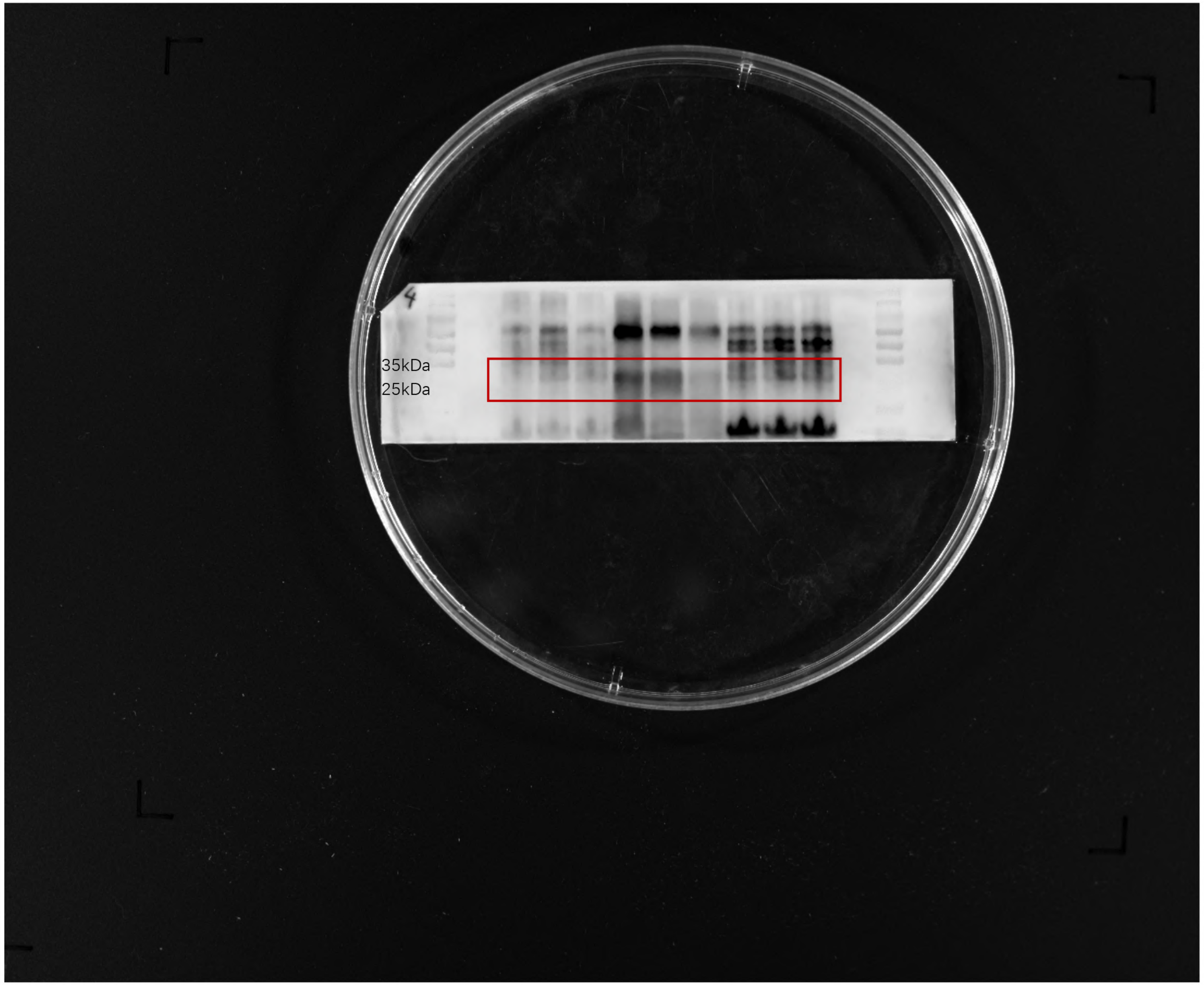

38kDa

Source Fig.8C p38

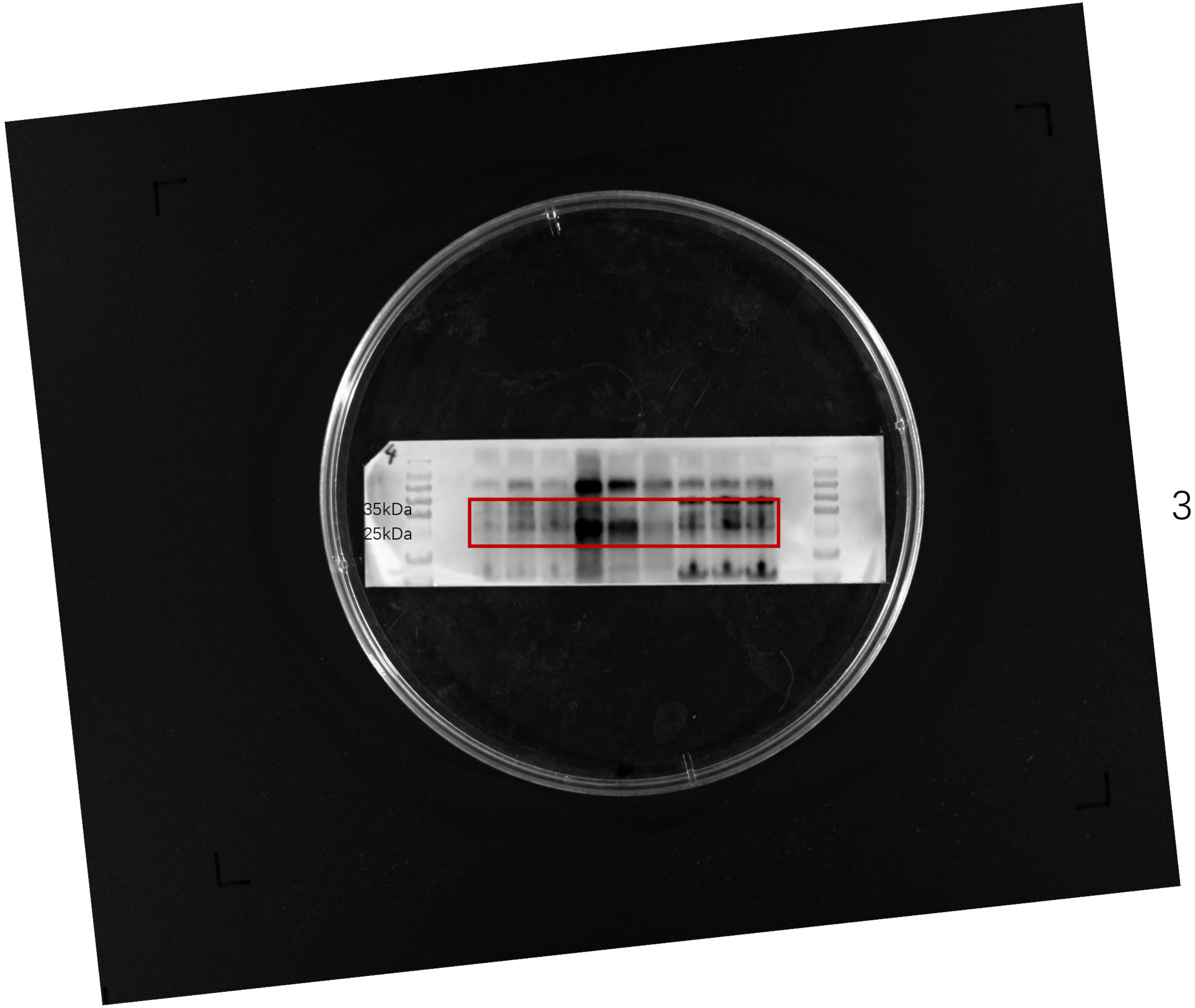

38kDa

Source Fig.8G KRT5

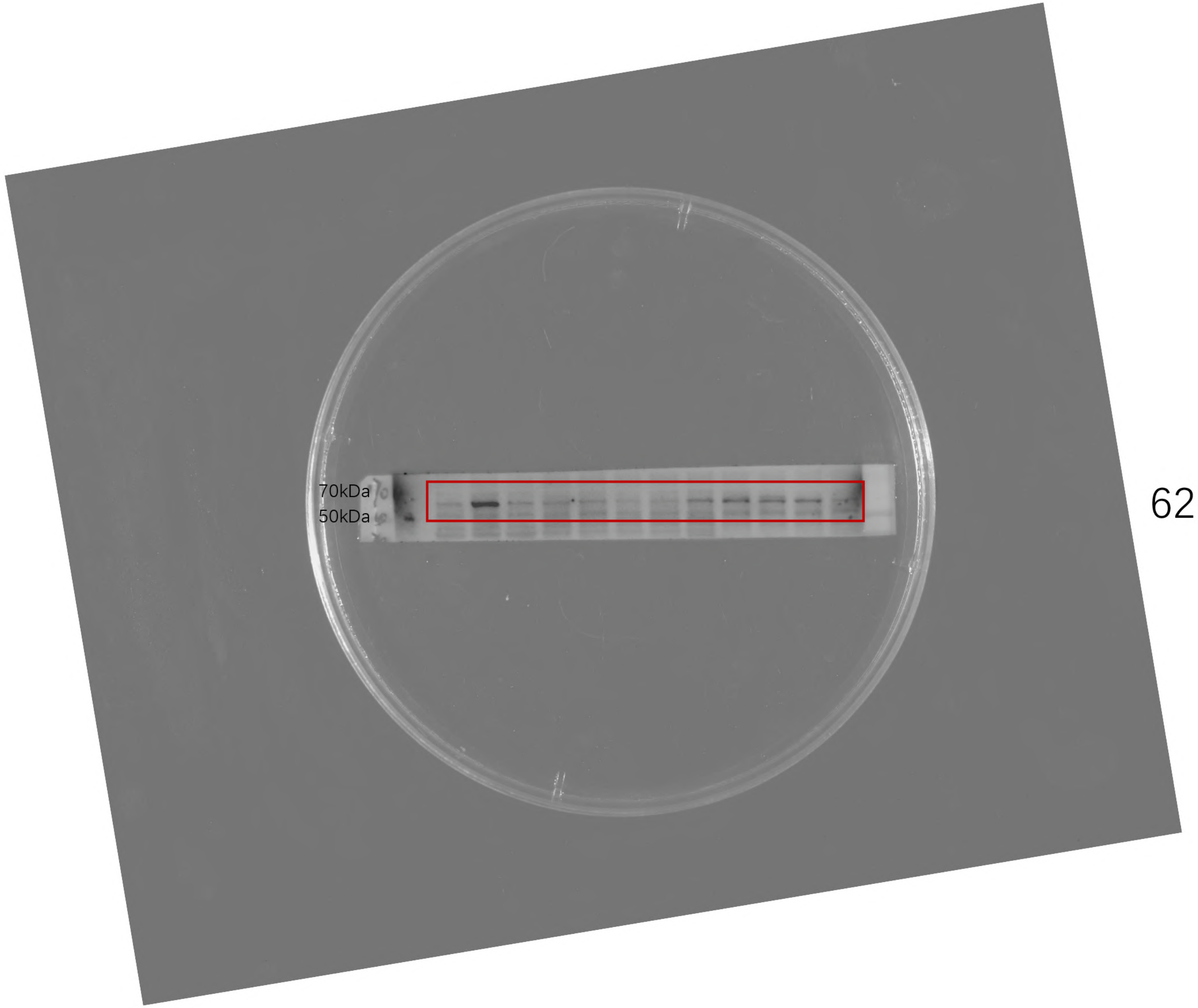

62kDa

Source Fig.8G GAPDH

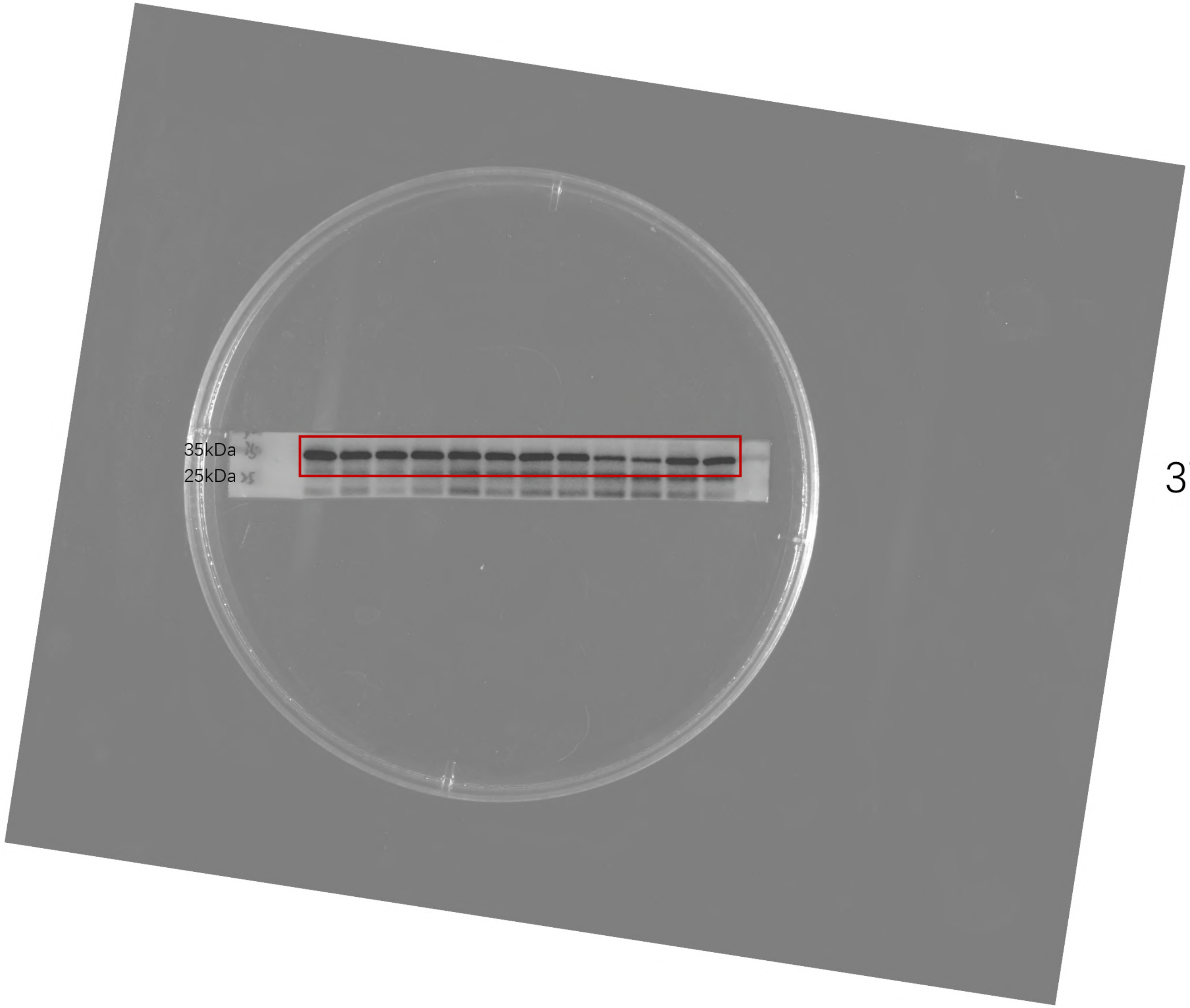

37kDa

Source Fig.8G KRT6

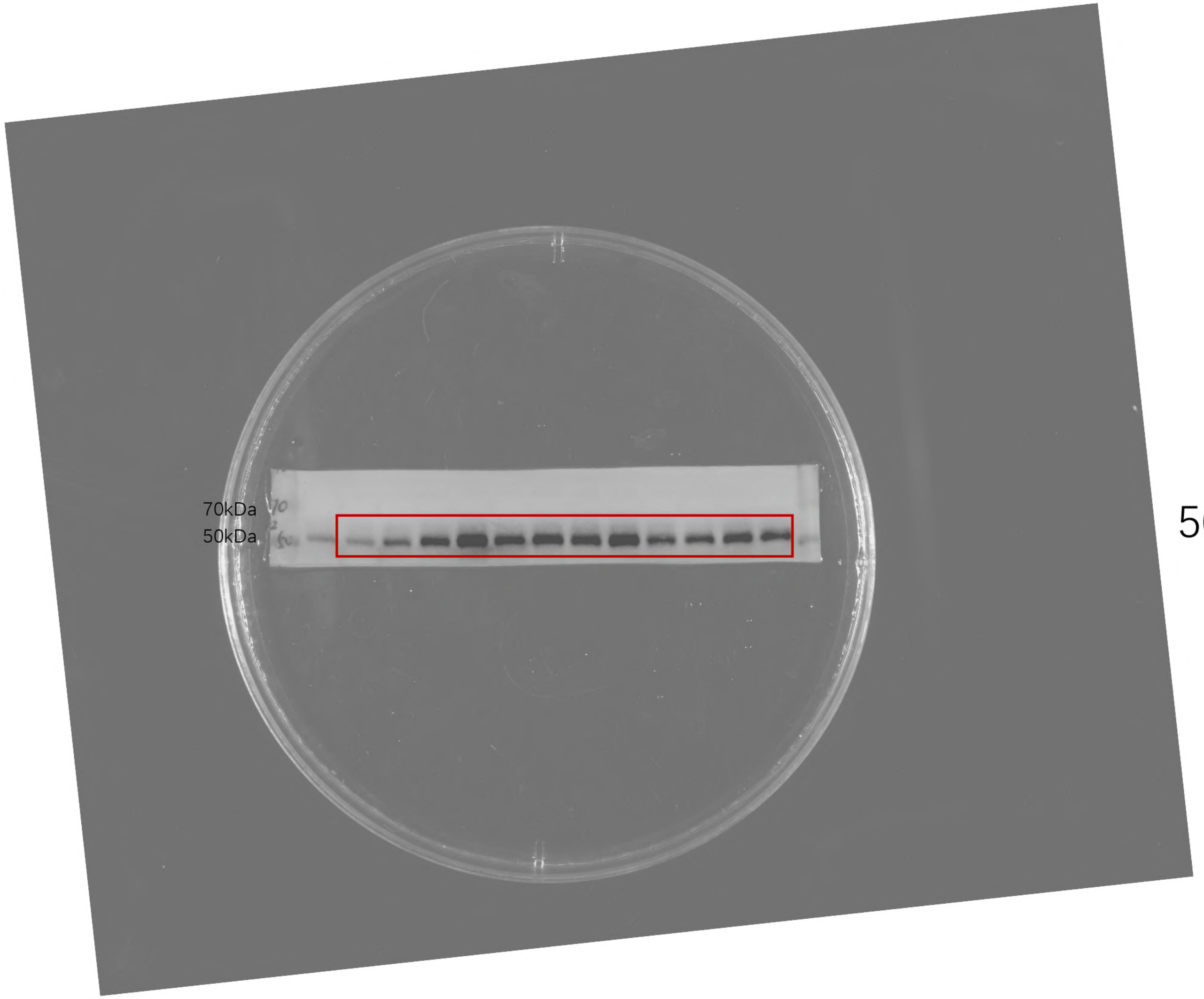

Source Fig.8G GAPDH

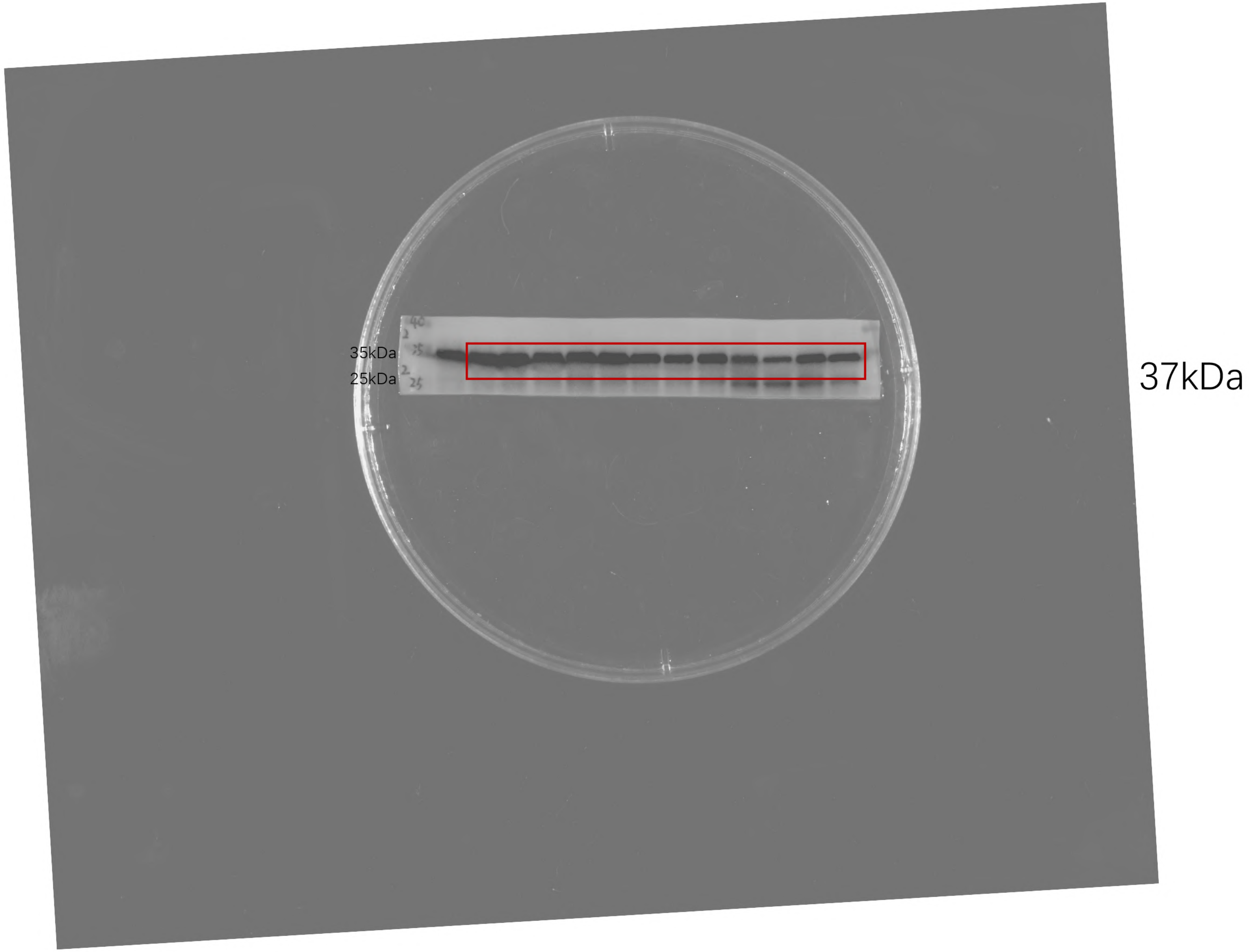

Source Fig.8G KRT14

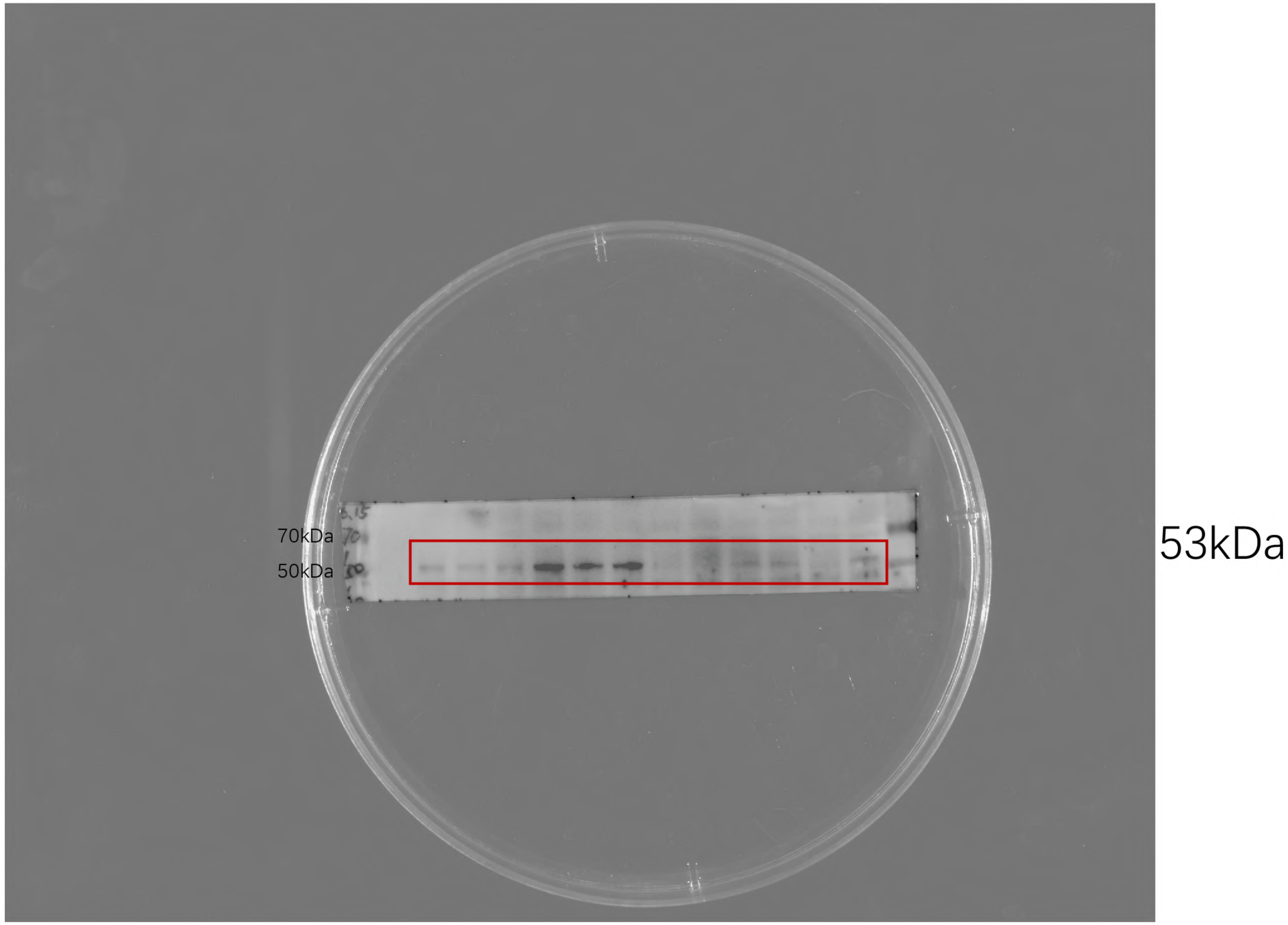

Source Fig.8G GAPDH

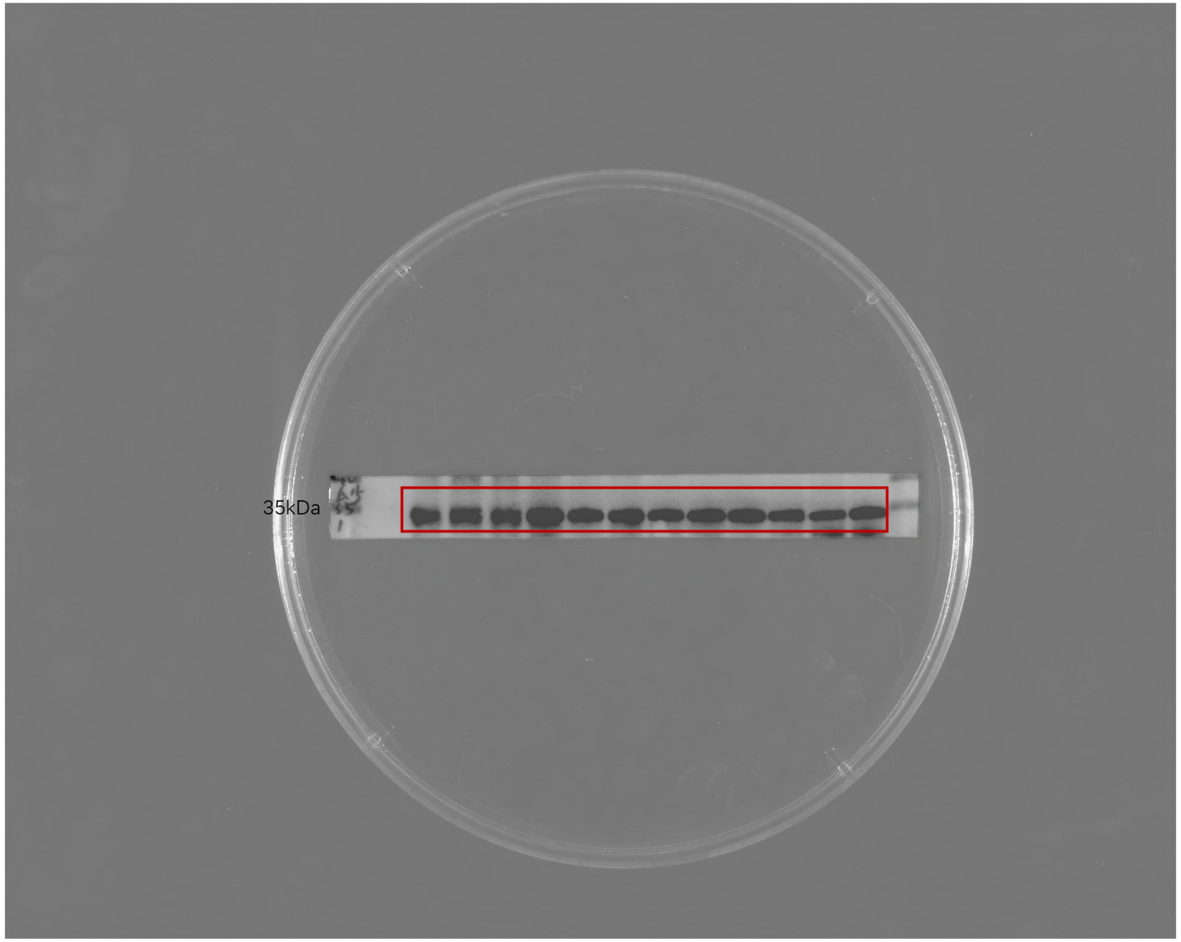

37kDa

Source Fig.8G KRT16

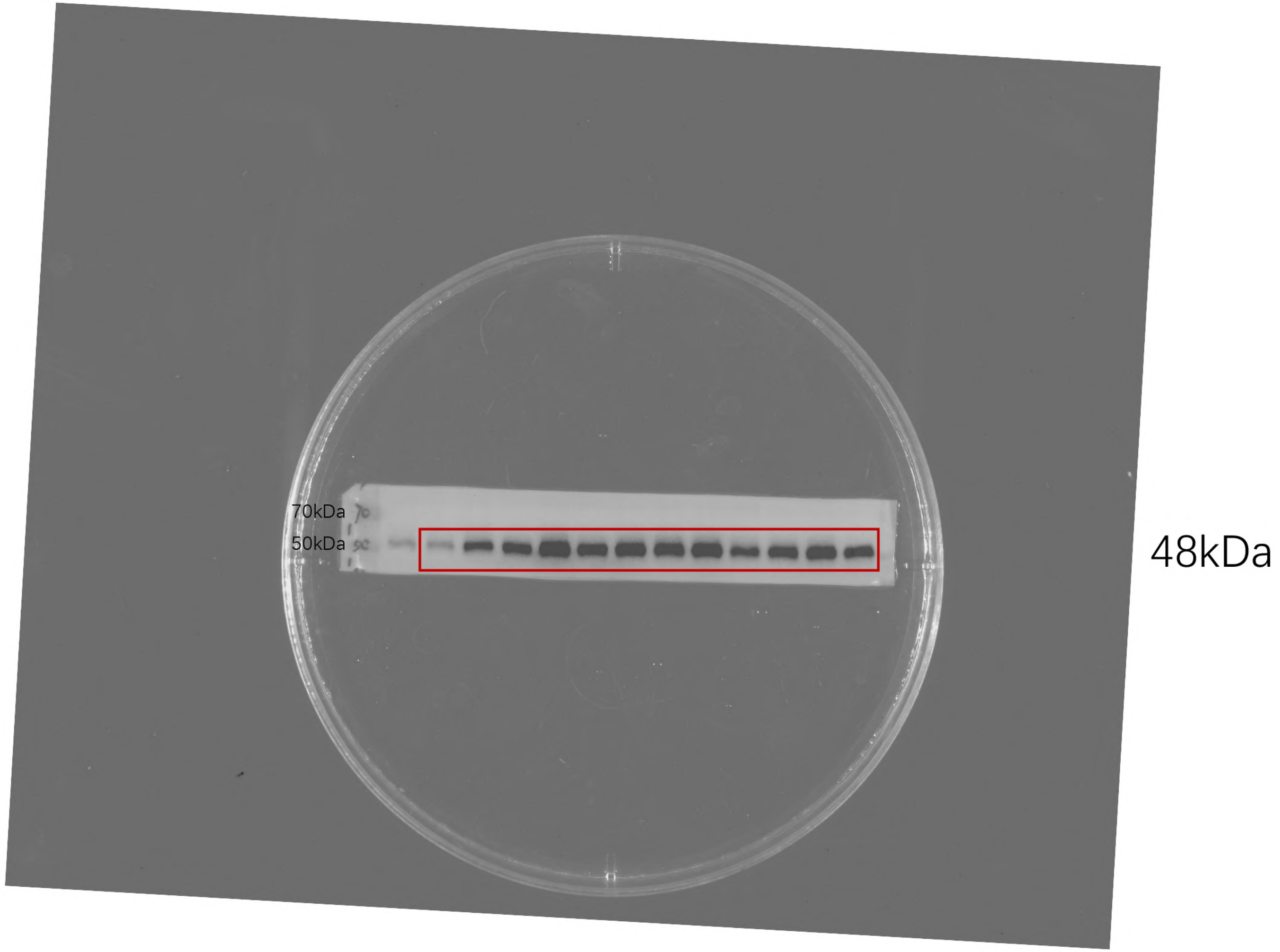

Source Fig.8G GAPDH

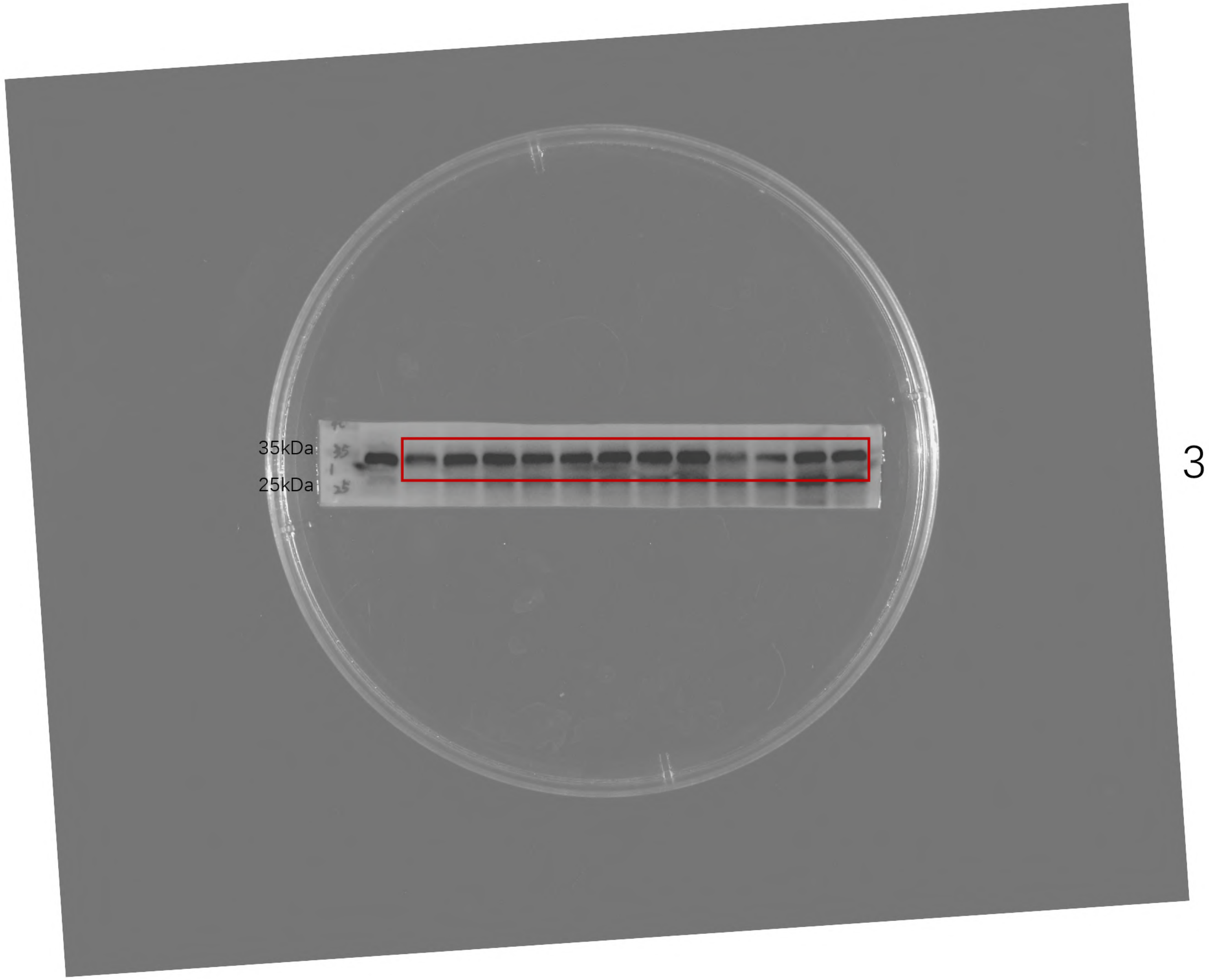

37kDa

Source Fig.S8A KRT5

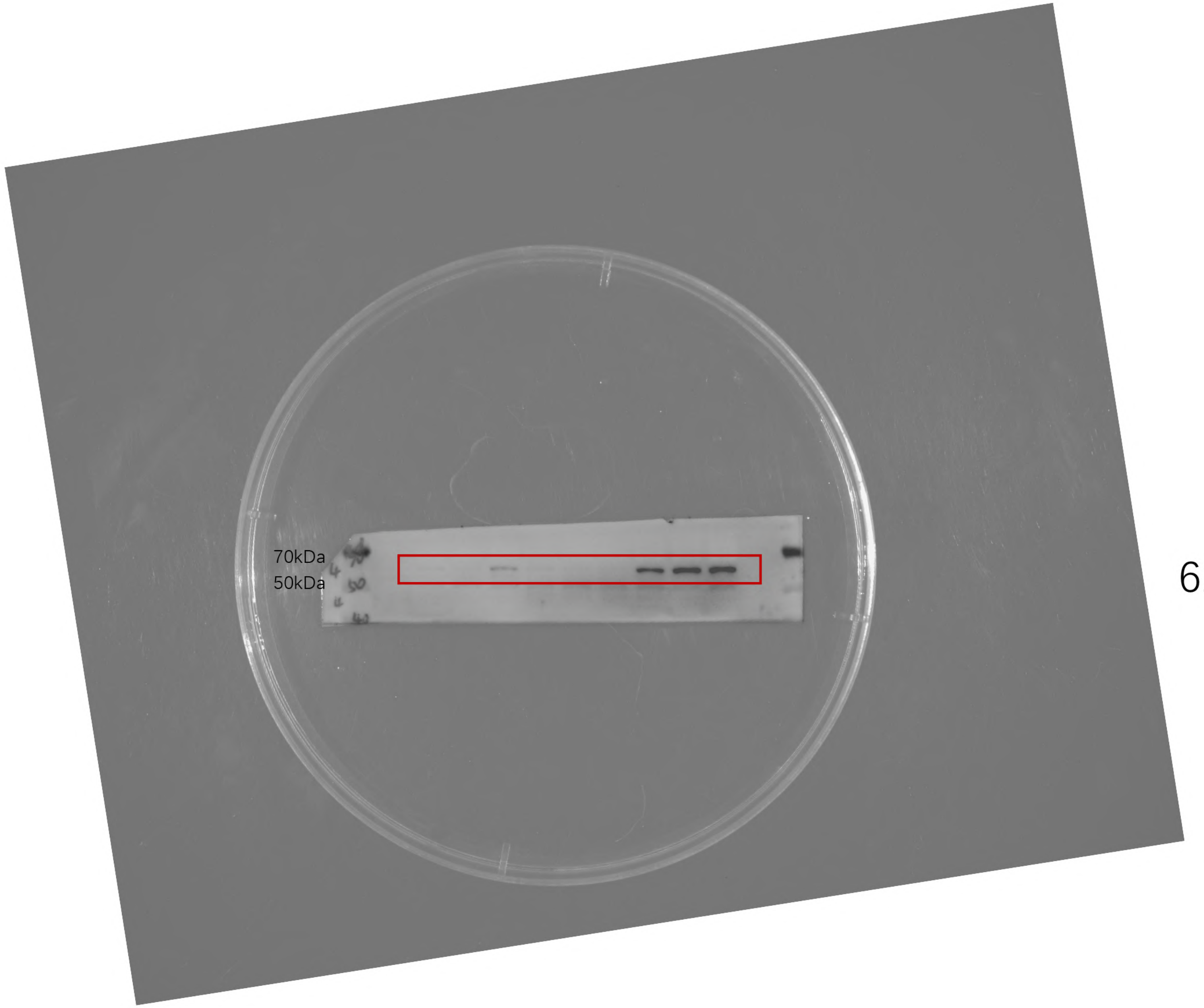

62kDa

Source Fig.S8A KRT6

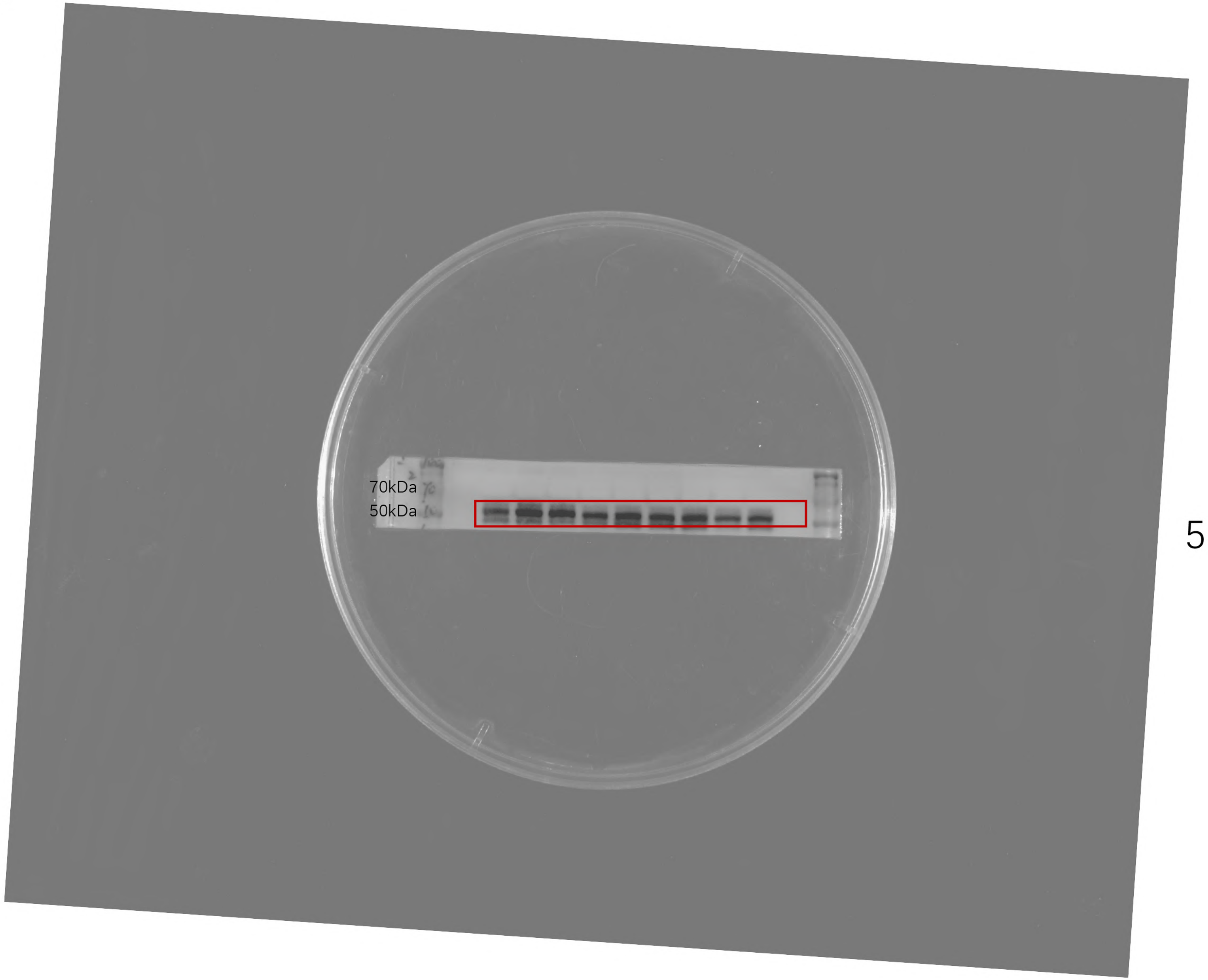

56kDa

Source Fig.S8A KRT14

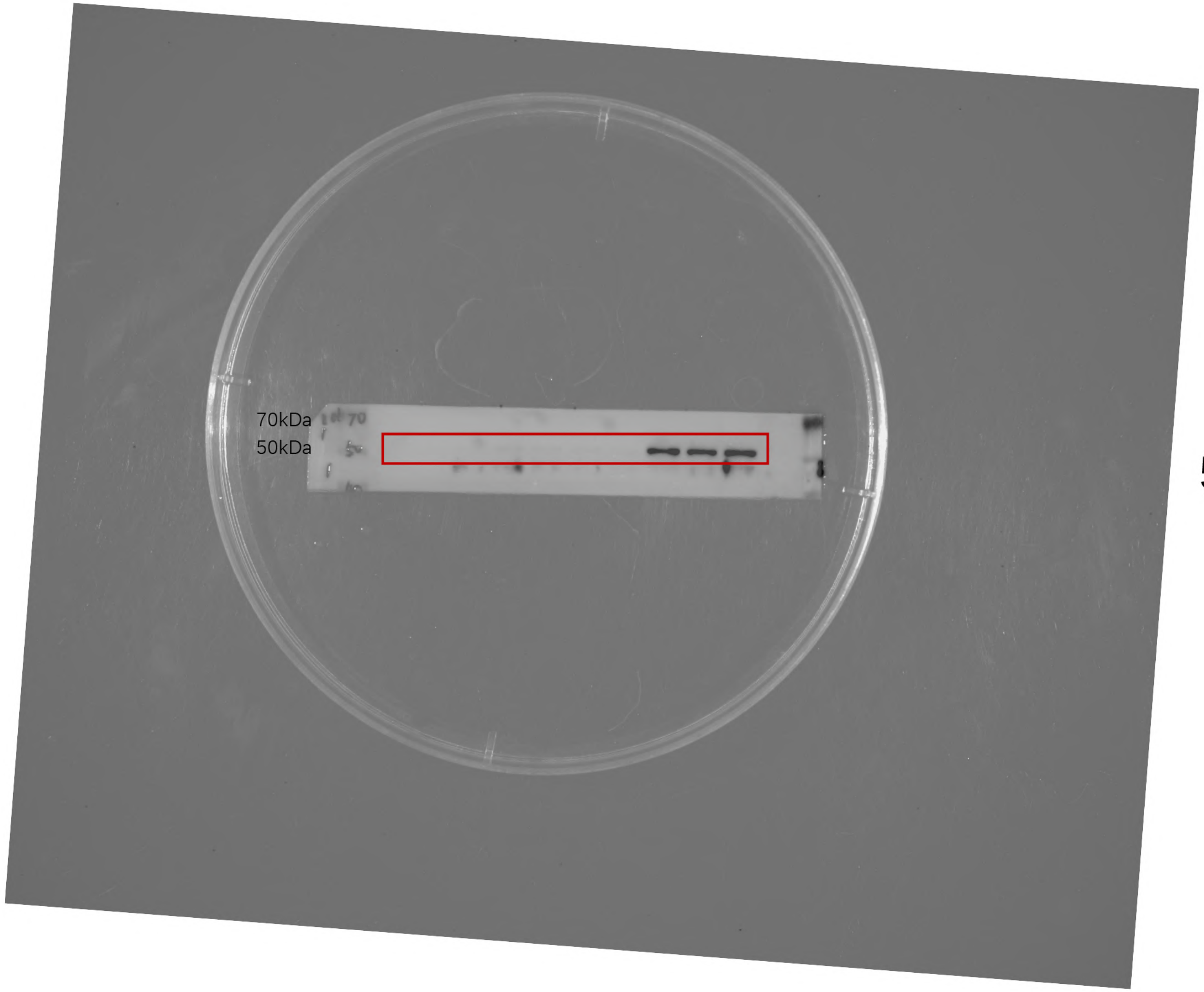

53kDa

Source Fig.S8A KRT16

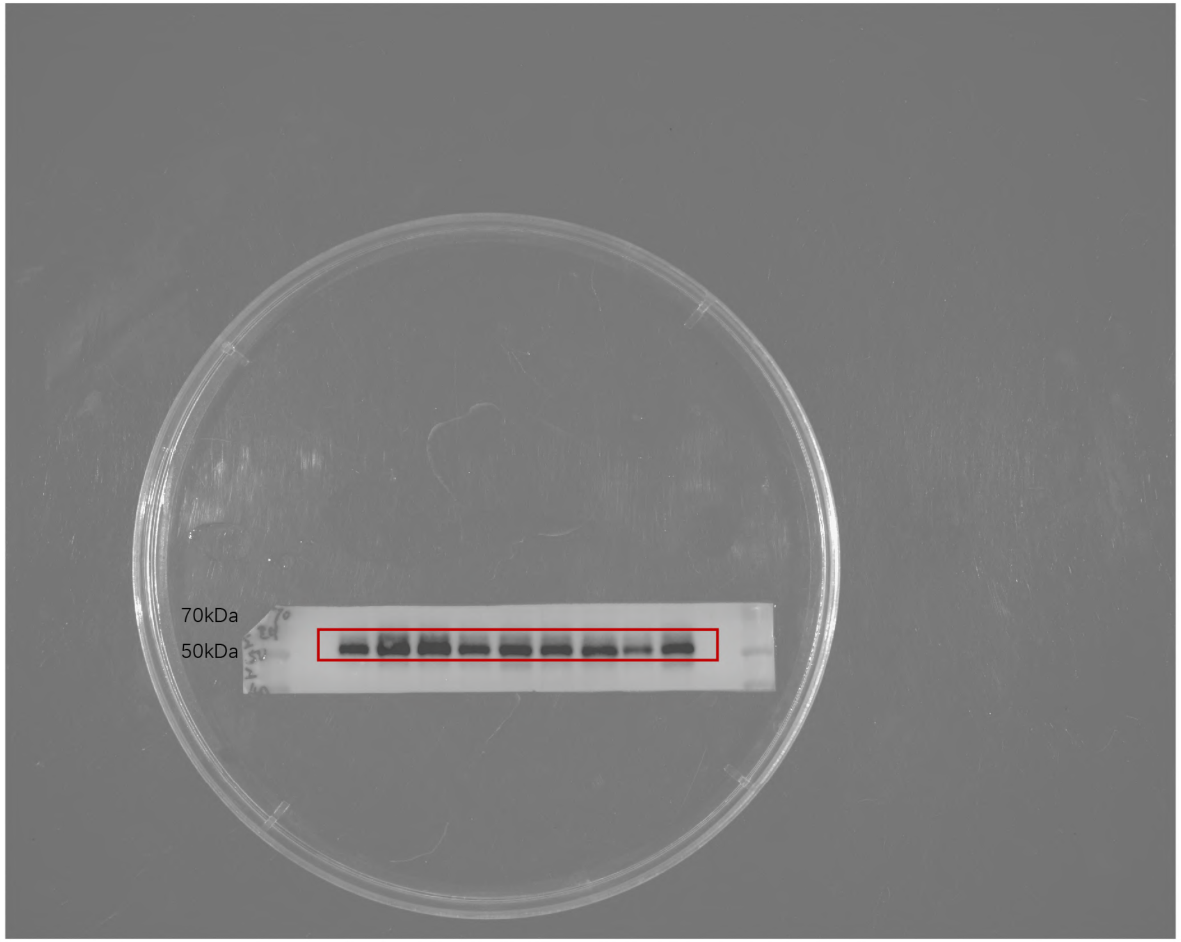

48kDa

Source Fig.S8A KRT17

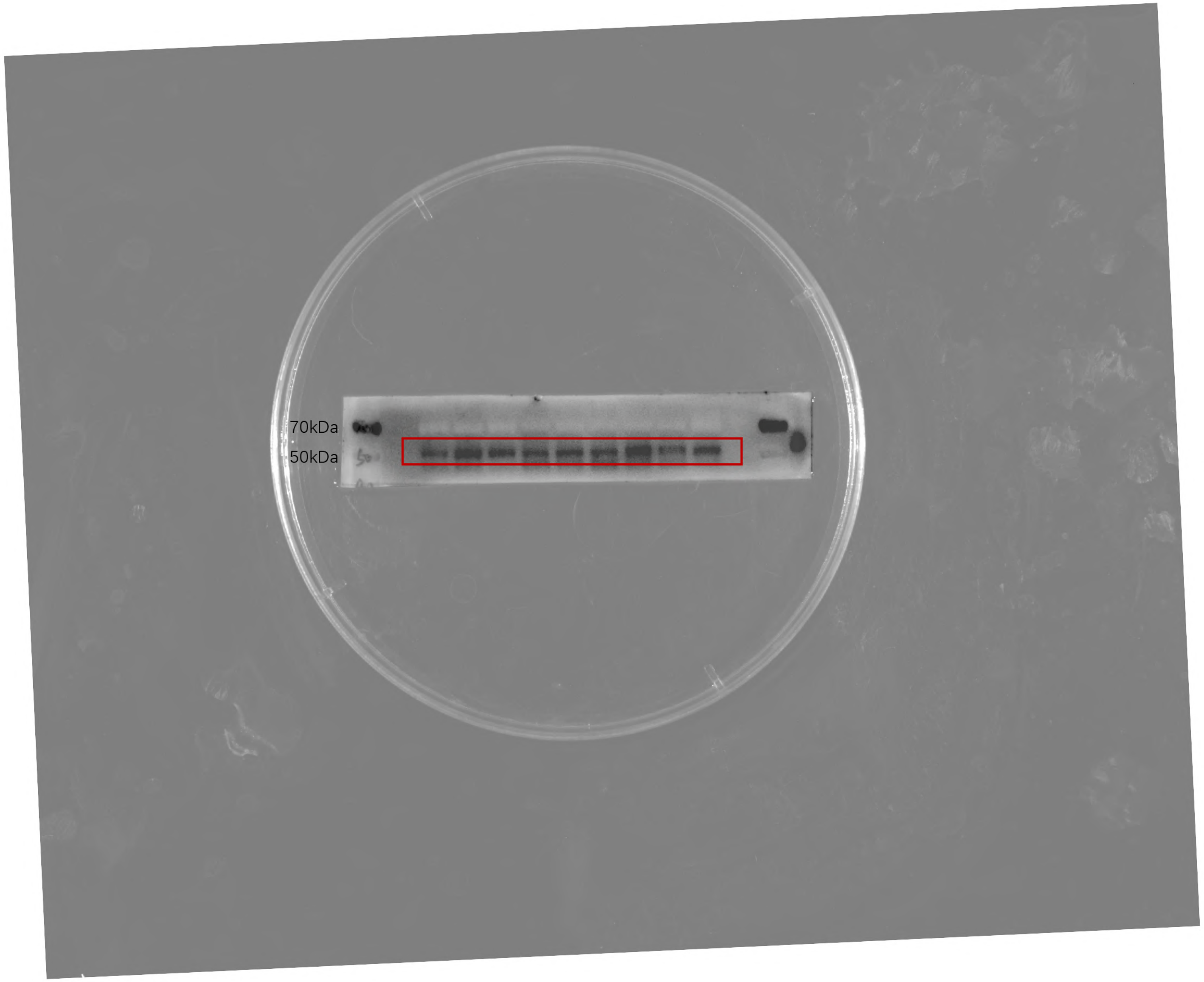

48kDa

Source Fig.S8A Fn14

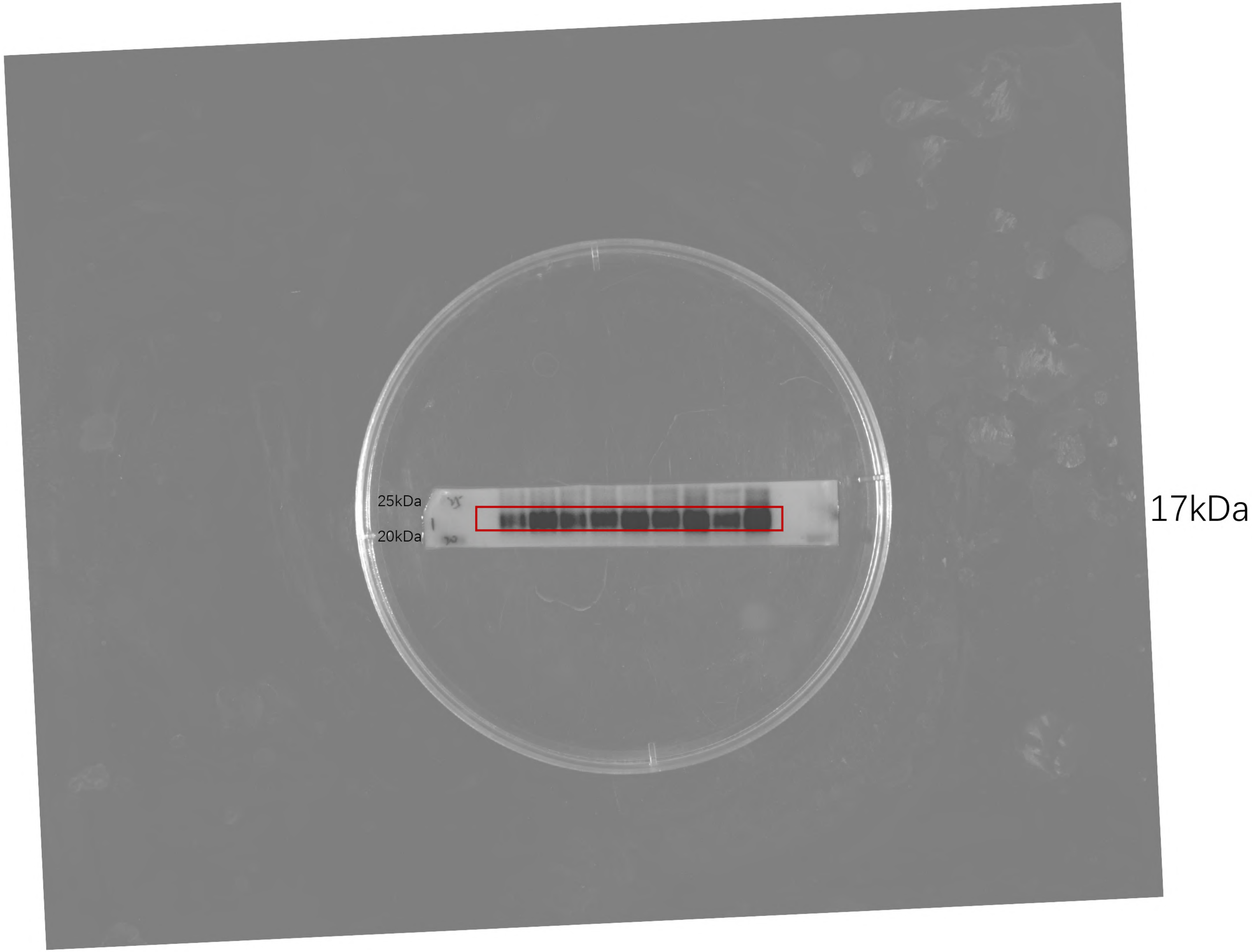

Source Fig.S8A CXCL10

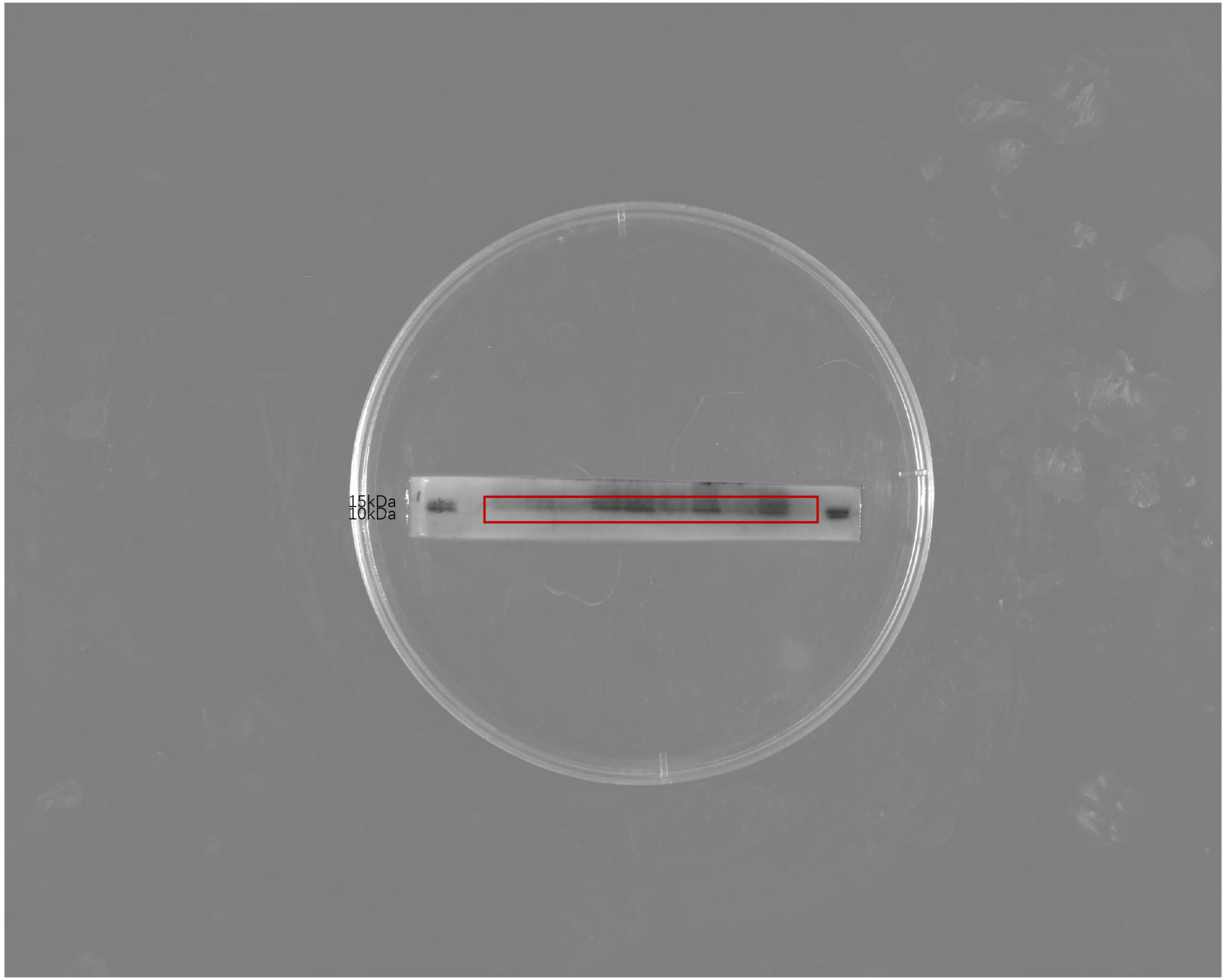

10kDa

Source Fig.S8A GAPDH

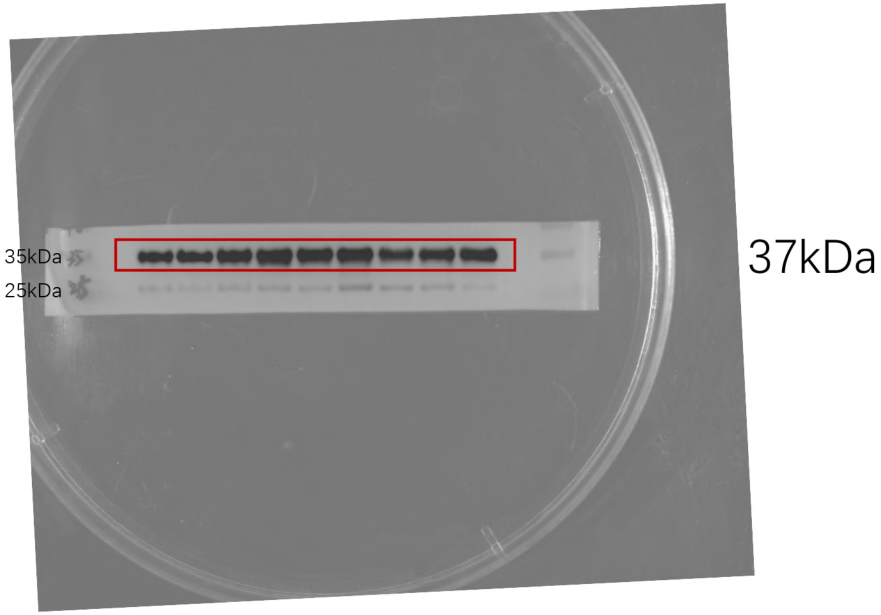

Source Fig.S11 LCN2

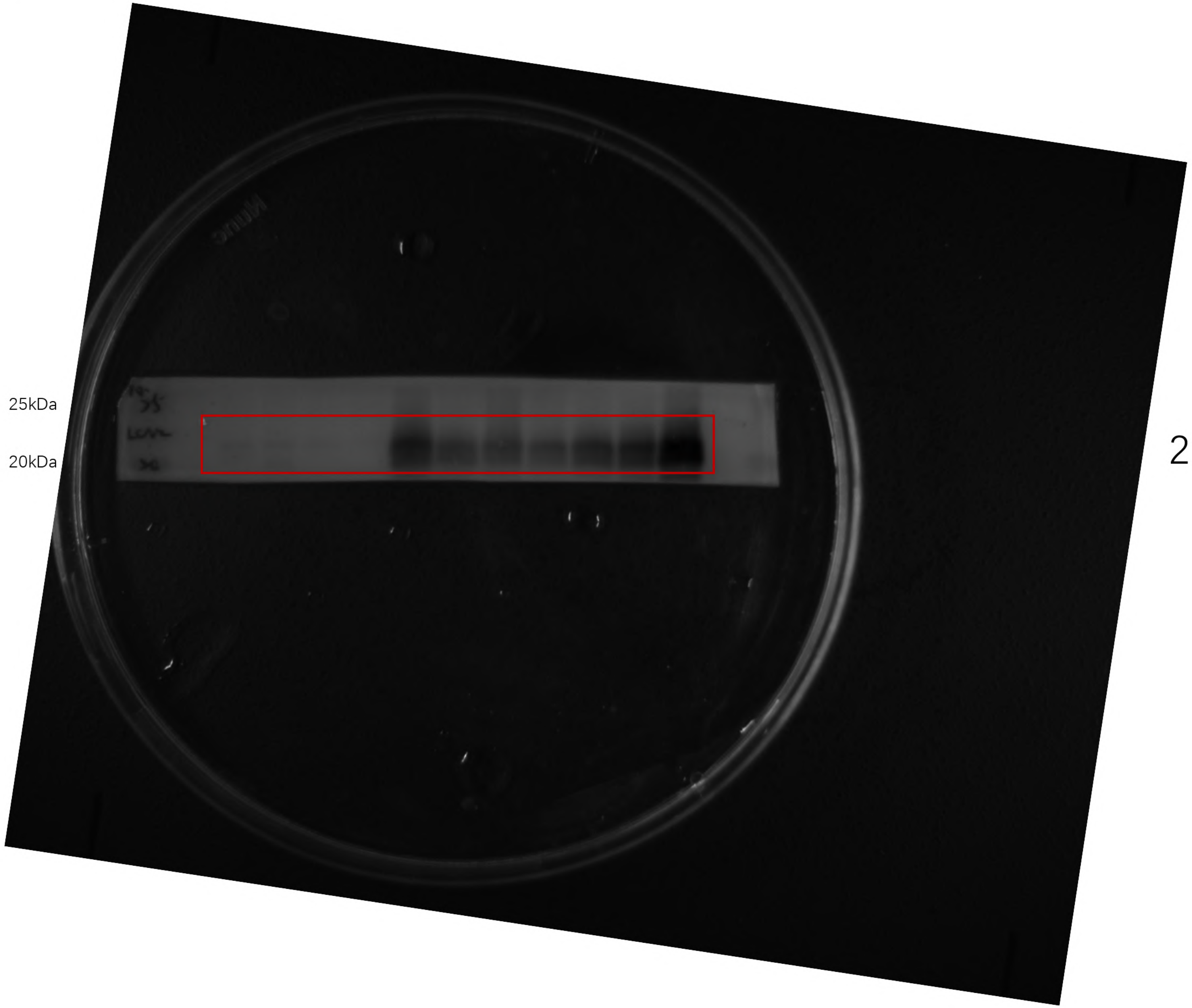

Source Fig.S11 GAPDH

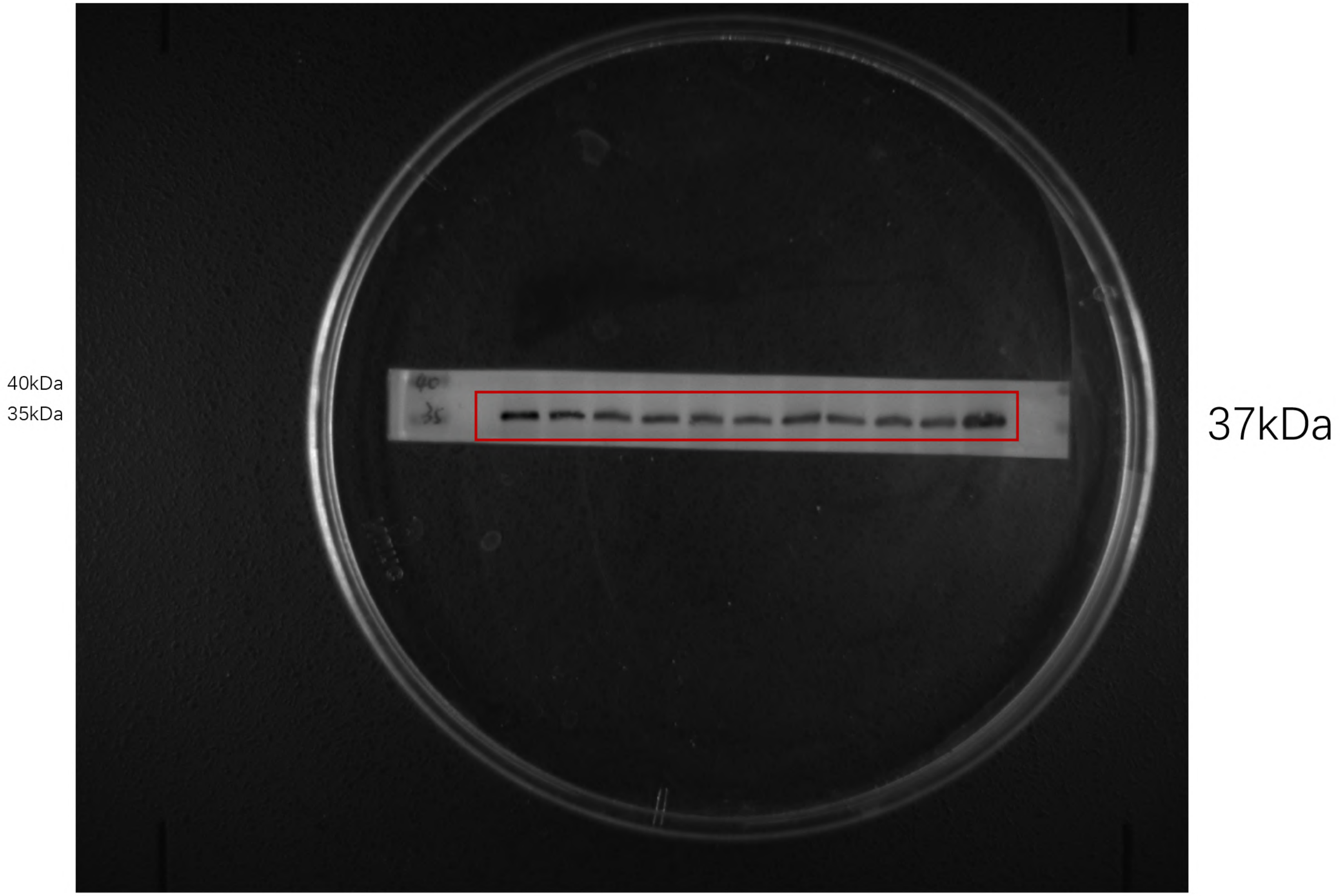

Supplement: Supplementary file 4 — Unprocessed images [file 41423_2025_1292_MOESM4_ESM.pdf]
